# Designing influenza virus-derived cell-penetrating peptides for antigen delivery: Integrating uptake efficiency, safety, and receptor targeting

**DOI:** 10.1371/journal.pone.0338028

**Published:** 2025-12-09

**Authors:** Sanaz Sadeh, Amir Ghaemi, Nazila Arbab Soleimani, Majid Moghbeli, Golnaz BahramAli

**Affiliations:** 1 Department of Microbiology, Damghan Branch, Islamic Azad University, Damghan, Iran; 2 Department of Influenza and other Respiratory Viruses, Pasteur Institute of Iran, Tehran, Iran; 3 Hepatitis and AIDS Department, Pasteur Institute of Iran, Tehran, Iran; Albert Einstein College of Medicine, UNITED STATES OF AMERICA

## Abstract

Successful antigen delivery is of paramount importance for successful vaccination. Cell-penetrating peptides (CPPs) offer a highly effective means of delivering antigens, nucleic acids, and other drug compounds to cells. However, their mechanisms of action remain poorly understood and require further investigation. This study sought to identify novel CPPs within the influenza virus proteome using computational screening methods for vaccine and antigen delivery. CPP candidates were screened from major influenza proteins using CellPPD, C2Pred, and PreTP-EL. Their efficiencies in uptake, physicochemical properties, and safety profiles were assessed using MLCPP, ProtParam, IEDB, ToxinPred, and AllerTop. Structural properties were assessed using AlphaFold, and binding interactions with the lung-targeting sialic acid analog LSTc were investigated using molecular docking and molecular dynamics simulations. Of the CPPs discovered, PB1-derived peptides, especially PB1−1 (RGDTQIQTRR), exhibit high membrane permeability and strong affinity for sialic acid receptors, along with low predicted toxicity and promising intracellular delivery capacity. PB1−1 forms a stable complex with LSTc, which pointed towards its potential for receptor-mediated lung targeting. The identified influenza-derived CPPs have strong therapeutic potential owing to their high predicted uptake efficiency, good safety profiles, and capacity for binding to lung-specific sialic acid receptors, suggesting their suitability for targeted vaccine or antigen delivery. These peptides take advantage of viral-mimetic entry pathways, including clathrin/caveolae-mediated endocytosis and direct membrane permeation, to efficiently deliver therapeutic cargo into cells. Cumulatively, our results suggest that influenza-derived CPPs, particularly PB1−1, may be suitable candidates for respiratory therapy and vaccine delivery. However, given the purely computational scope, the results should be considered hypothesis-generating and require experimental validation in vitro and in vivo.

## 1. Introduction

The development of vaccines is a major step toward the elimination of various diseases. Successful vaccination depends on long-lasting immune responses and protection against infections. This technique requires an accurate and effective antigen delivery to stimulate the immune system. Different methods are used to transport cargo across barriers [1].

CPPs, also known as protein transduction domains (PTDs) or Trojan peptides, are powerful biological nanocarriers that can overcome natural barriers. They can cross biological membranes and deliver membrane-impermeable compounds into the cells [2]. These short, positively charged peptides contain 5–30 amino acids. Through endocytosis, CPPs can transfer bioactive compounds to different cell types [3]. Recently, CPPs such as penetratin and transportan have been found to carry bioactive molecules and therapeutics such as proteins, peptides, and oligonucleotides in vitro and in vivo [3].

Many CPPs originate from viruses, which can penetrate cell membranes and distribute substances. They are frequently derived from viral proteins that naturally support infection, such as the HIV-1 Trans-Activator of Transcription (TAT) protein, one of the most studied viral CPPs [4]. It has been reported that TAT enhances cytoplasmic molecule absorption [2]. There are other transducing proteins than HIV-1 TAT, such as HSV-1 VP22 and HPV L2 [5]. CPPs are promising agents for the delivery of therapeutic molecules to cells. However, their membrane translocation process is poorly understood and they are yet to be clinically applied. Discovering new and, more effective CPPs with higher bioavailability and fewer negative effects for safe gene delivery and therapeutic applications can be challenging [6].

The traditional high-throughput laboratory screening of novel CPPs requires time and effort. However, prior to laboratory studies, in silico prediction approaches are more efficient and cost-effective. These methods have been shown to accelerate CPP-based research [7]. CPPs can be predicted using molecular docking, neural networks, and support vector machines (SVM). These computational methods have improved the efficiency of lab-based methods [8–10].

The influenza A virus genome is comprised of eight segments of negative-sense single-stranded viral RNA (vRNA). These segments encode ten viral proteins, including the structural components hemagglutinin (HA), neuraminidase (NA), matrix proteins (M1 and M2), and nucleoprotein. Additionally, the genome encodes non-structural proteins, such as NS1, which enable the virus to evade the host immune system and replicate. PA, PB1, and PB2 form a viral RNA polymerase complex that is essential for RNA transcription and replication.

Although various influenza virus proteins have been investigated, there are no comprehensive reports on cell-penetrating peptides (CPPs) derived from all influenza proteins. Some studies suggest that peptides located in the hemagglutinin cleavage site (pHACS) of highly pathogenic influenza A viruses, notably subtypes H5 and H7, facilitate host cell adhesion and membrane fusion [11]. The multibasic cleavage site found in these highly pathogenic viruses is cleaved by the widely expressed furin-like proteases. As a result, these viruses exhibit higher pathogenicity and can infect multiple tissues owing to their more efficient cleavage [11].

Given the strong transmissibility and infectivity of influenza virus, we employed in silico approaches to discover novel CPPs within its proteins. These CPPs may enhance gene and vaccine delivery and offer potential improvements for the treatment of a broad range of diseases.

## Materials and methods

### 1.1. Identification of potential CPPs

This study aimed to identify and describe Influenza A virus protein-derived cell-penetrating peptides (CPPs) using a combination of bioinformatics tools to screen the influenza A virus (H1N1 subtype) proteome for CPP and non-CPP candidates. This study was performed entirely using in-silico analyses and did not involve human participants, animal subjects, or biological specimens. Therefore, no ethics approval or consent was required.

We used the reference genome of the Influenza A virus strain A/Puerto Rico/8/1934 (H1N1), NCBI Taxon ID 211044, which is a common virus used in virological studies because of its well-characterized genetic and antigenic properties. The PR8 genome is composed of eight single-stranded RNA segments of approximately 13,500 nucleotides [12].

Sequences of the hemagglutinin (HA) protein (GenBank ID: NP_040980.1), neuraminidase (NA) protein (GenBank ID: NP_040981.1), nucleoprotein (NP) protein (GenBank ID: NP_040982.1), nonstructural protein 1 (NS1) (GenBank ID: NP_040984.1), nonstructural protein 2 (NS2-NEP) (GenBank ID: NP_040983.1), polymerase PA (PA) protein (GenBank ID: NP_040986.1), polymerase PB1 (PB1) protein (GenBank ID: NP_040985.1), polymerase PB2 (PB2) protein (GenBank ID: NP_040987.1), matrix protein 1 (M1) protein (GenBank ID: NP_040978.1), and matrix protein 2 (M2) protein (GenBank ID: NP_040979.2).

We first analyzed the sequences using the CellPPD web server (https://webs.iiitd.edu.in/raghava/cellppd/index.html), which employs a support vector machine (SVM) model to predict potential cell-penetrating peptides (CPPs). SVM scores were then assigned to the identified CPP candidates to reflect their likelihood of cellular entry [10,13,14]. The CellPPD server retrieved these sequences by using an SVM prediction threshold of 0.0. The identified peptides were further evaluated using the C2Pred web server (http://lin-group.cn/server/C2Pred) [14]. This server uses machine learning techniques to evaluate the probability of CPP based on peptide features with a classification threshold of 0.5. Peptides scoring below 0.5 were classified as non-CPPs, and those above as CPPs. This tool offers a prediction accuracy of 83.6%.

We also used the PreTP-EL web server to further evaluate the transport properties of the predicted CPPs by adding an additional validation layer. This server distinguishes between CPPs and non-CPPs [15]. According to the results obtained in this study, the server used a threshold score of 0.5 to classify CPPs. Peptides with a score greater than 0.5 were classified as non-CPPs, while those with a score less than 0.5 were classified as CPPs.

### 1.2. CPP uptake efficiency

#### CPP uptake efficiency.

was used to assess the uptake efficiency of the CPPs identified in the previous step using the MLCPP web server (http://www.thegleelab.org/MLCPP/). For this analysis, all CPPs detected using the CellPPD web server were submitted to MLCPP for evaluation. MLCPP improves CPP identification by using a two-layer prediction framework. In the initial layer, peptides are categorized as CPPs or non-CPPs using machine learning algorithms. Once classified, the second layer estimates the uptake efficiency of each peptide, categorizing them as either high or low uptake based on their predicted ability to penetrate cell membranes. This method provides insights into peptide characteristics and their potential applications in gene delivery [16].

### 1.3. Physicochemical properties of CPPs

Understanding the physicochemical properties of CPPs is crucial for evaluating their efficacies. Molecular weight (MW), net charge, theoretical pI, amphipathicity, hydropathicity, hydrophobicity, hydrophilicity, steric hindrance, net hydrogen, and side chain volume are some of the properties calculated using computational tools. The CellPPD web server (https://webs.iiitd.edu.in/raghava/cellppd/index.html) was used for analysis.

### 1.4. Assessment of membrane-binding potential of CPPs

We employed the Boman index to assess the likelihood of a peptide interacting with the membrane. This computational method determines the binding affinity of a peptide by adding the solubility values of all the amino acids in the peptide sequence. An elevated Boman index indicates an increased likelihood of interaction with membranes or proteins. This method is useful because it considers both total hydrophobicity and the potential for non-specific binding—two key factors in determining peptide–membrane interactions. We computed the Boman index using the APD3 web server (http://aps.unmc.edu/AP/prediction/prediction_main.php) [17].

The TMHMM web server (http://www.cbs.dtu.dk/services/TMHMM/) was used to predict the quantity and positioning of transmembrane helices within a protein. This tool applies a Hidden Markov Model trained on known protein sequences to identify the amino acid probability profiles associated with transmembrane regions. This model was trained on known protein sequences to examine the properties of transmembrane helices, such as how likely they were to contain hydrophobic residues. TMHMM also predicts the spanning or interaction of these peptides with the plasma membrane. This step is necessary to understand how CPPs are located within the membrane or interact with its parts, which could affect the movement of therapeutic molecules across cellular membranes [18].

### 1.5. Immunogenicity assessment of CPPs using the IEDB immunogenicity predictor

Immunogenicity assessment is a major consideration in the development of CPPs for therapeutic purposes.As unwanted immune responses can reduce peptide efficacy, minimizing immunogenicity at the design stage is essential. We used a bioinformatics tool, the IEDB Immunogenicity Predictor, to predict the immunogenicity of CPPs, as it provides the probability of peptide presentation by a major histocompatibility complex. The IEDB tool employs experimentally validated algorithms to predict peptide binding to various MHC alleles [13,19]. If a predicted peptide that is thought to have a strong affinity for a certain MHC allele is given repeatedly, T cells may be able to recognize it and react to an immunogen containing this peptide. In this study, by incorporating immunogenicity prediction into our analysis, we identified low immunogenic potential CPPs, a criterion supporting the safety and efficacy of these peptides as therapeutic agents [13].

### 1.6. Toxicity and allergenicity

We evaluated the predicted peptides using specialized web-based tools to assess the toxicity and allergenicity of the CPPs. The ToxinPred web server (https://webs.iiitd.edu.in/raghava/toxinpred/algo.php) was used to predict the toxicity. This server checks the toxic potential of the peptides based on their amino acid sequences. To sort each query sequence, the method uses a peptide database and machine-learning algorithms to assess toxicity [20].

Allergy prediction was conducted using the following web servers: (https://www.ddg-pharmfac.net/allerTOP/) and (http://ddg-pharmfac.net/allergenFP/). AllerTop utilizes an alignment-free method to predict the allergenicity of peptides by integrating amino acid properties with the ACC transformation [21]. AllergenFP refers to FingerPrint, a descriptor-based approach for predicting allergenicity. The process involves converting the peptide sequence into a binary string based on distinct characteristics, followed by comparison with a database of known allergens [18].

### 1.7. Half-life prediction

The half-life and stabilities of these peptides were estimated using specialized tools capable of providing valuable predictions. The ProtParam tool, accessible at (https://web.expasy.org/protparam), was used to evaluate the theoretical half-life of the peptides in *E. coli* and mammalian cells, as well as the instability index of the peptides.

#### Evaluation of helix properties.

Examination of the structural characteristics of these CPPs employed a Schiffer-Edmundson wheel model to graphically represent the amphipathic properties of alpha helices in peptides through a helical wheel diagram. A specialized web server, Heliquest, accessible at https://heliquest.ipmc.cnrs.fr/cgi-bin/ComputParams.py, was utilized for the generation of these diagrams. The Schiffer-Edmundson wheel model illustrates, in two dimensions, a circular projection of an alpha helix, providing an approximation of the amino acid residue positioning within a helical structure. This method effectively analyzed the distribution of hydrophobic and hydrophilic residues within the helix. Heliquest provides a graphical representation of the helix and conducts various calculations, including determination of the hydrophobic moment μH, which characterizes the amphipathic properties of the peptide. Amphipathic describes the presence of both hydrophobic and hydrophilic regions, which are crucial for peptide interactions with the lipid bilayers. An increased hydrophobic moment indicates a greater segregation of hydrophobic or hydrophilic residues, which is typically associated with the capacity of the peptide to penetrate cells through translocation across membranes [22].

#### 3D modeling of CPP.

Recently, significant advancements have been made in deep learning for peptide structure prediction. AlphaFold) https://deepmind.google/technologies/alphafold/ (exemplifies a leading tool for the accurate, high-resolution modeling of peptide structures [23, 24]. AlphaFold, a deep learning algorithm that uses specialized neural networks and is based on the coevolutionary information of proteins and peptides with multiple sequence alignments (MSA), has been developed [25]. Accordingly, we utilized AlphaFold to predict the structure of peptides and proteins [26]. Therefore, the amino acid sequence of each CPP was exposed to the Alphafold server for the 3D structure prediction. Several 3D models were generated for each CPP using AlphaFold. Alphafold is known to be the best for CASP 15. AlphaFold outputs the pLDDT value and a visual inspection of the predicted 3D structure for each CPPs. The pLDDT plot provides an insight into the feasibility of CPP in stable structures. Higher pLDDT scores reflect greater confidence in the predicted model. The resulting 3D structures highlighted distinct amphipathic alpha-helices, and a key motif emerged as being particularly important for how these peptides interact with lipid bilayers, which are often used as a stand-in for cell membranes.

#### CPP 3d model refinement.

GalaxyRefine (http://galaxy.seoklab.org/) server was employed to rebuild the side chains, and side-chain repacking and relaxation 3D structures for each CPP to enhance the global and local structure quality. This server is known as the top-scoring algorithm in CASP 15. galaxyRefine output refined structure, which can be evaluated in terms of its different refinement parameters, such as GDT-HA (high accuracy version of GDT), Root Mean Square Deviation (RMSD), MolProbity, and clash score [27].

#### CPP 3D model validation.

Finally, we evaluated the top refined model from the galaxy output and subjected it to the ProSA-Web server (https://prosa.services.came. sbg. ac. at/ prosa. php) To assess the accuracy and potential errors of the predicted 3D structure before molecular docking and dynamic simulations [28,29]. The server computes the Z-score, which indicates the quality statistics of the 3D predicted model. Negative Z-scores represent a well-folded, high-quality protein structure.

### 1.8. Docking preparation

To achieve this, we selected LSTc, a well-studied sialooligosaccharide recognized for its strong binding affinity to HA, as a ligand because it effectively mimics the natural HA receptor. HA plays a major role in the attachment of viruses to host cells through the binding of sialic acid moieties of host cells [30]. This binding supports entry into the host cell mainly through the clathrin-mediated endocytosis pathway, although other pathways such as macropinocytosis cannot be completely excluded [31]. The LSTc structure was obtained from PDB code 8FAW and prepared using the HADDOCK protocol for high-accuracy docking. To this end, we systematically used the performance of two standard docking programs, HADDOCK and HDOCK, to model and cluster the CPPs LSTc complex. Both are information-driven docking methods [32,33]. The lowest HADDOCK and HDOCK scores of CPPs/ LSTc clusters were subjected to further analysis and molecular dynamic behavior assessment. We set the unambiguous restraint energy constants to 1 during the first iteration and 0 during the last iteration, whereas we set the ambiguous ones to 0 during the last as proposed in the HADDOCK. In the scoring parameters, Evdw_1 is equal to 1.0, and Eelec_3 is 0.1. The best scoring complex of CPPs/LSTc was subjected to PRODIGY to determine the Gibbs free energy (ΔG) parameter.. PRODIGY forecasts protein–protein binding affinity using a contact/functional-based approach [34].

### 1.9. MD simulation method

#### 1- MD simulations of CPPs candidates to LSTc ligand.

Complexes of LSTc and the CPPs PB2−1, PB1−1, PB1−2, PB1−3, and PB2−2 from optimal docking results. These were used in the molecular dynamics simulations. Complexes were prepared for structural optimization and minimization simulations. The GROMACS software package (versions 35 and 36) was used for energy minimization and equilibration processes of the MD simulations. The CHARMM36 force field was used to describe the system. All complexes were oriented in a cubic box (10 Å × 10 Å) to solvate using the explicit TIP3P water model, and counter ions were added to neutralize the systems to simulate physiological conditions [35]. The solvated system underwent steepest-descent energy minimization utilizing 50,000 units for each complex. Position restraints for both NVT and NPT ensemble For equilibration was calibrated for 100 ps with modified Berendsen thermostat and Parrinello Rahman pressure coupling [36,37]. A pressure of 1 bar and a temperature of 300 K (physiological temperature) were all well equilibrated, and the production MD simulation was set out for 100 ns for each complex. Throughout the simulation, trajectories were recorded at 20 ps intervals to enable further analysis of the simulation results.

#### 2- Molecular dynamics simulation of the PB1−1 CPP with POPC membrane.

Molecular dynamics (MD) simulations provide a method for examining the interatomic interactions between peptides and membranes and have become an effective complement to experiments. For cell penetration simulation, after docking, the top-scoring peptide of the identified Cell-Penetrating Peptides in Influenza A (H1N1) according to the HADDOCK score was selected and set out to investigate the CPP crossing mechanism through the cell membrane phospholipids in the simulation environment. Classical MD simulations were conducted using GROMACS software package (versions 35 and 36), in conjunction with the CHARMM36 force field. Due to literature Palmitoyl oleyl phosphatidylcholine (POPC) is widely used in MD simulations of biological cell membrane systems because the results are consistent with experimental data [38]. Therefore, the starting structure of a pure palmitoyloleoyl-phosphatidylcholine (POPC) simple bilayer model involving 128 lipids in each leaflet was first generated using the CHARMM-GUI Membrane Builder (www.charmm-gui.org). Membranes without water were used to construct systems containing the PB1−1 peptide molecules. The peptides were first oriented above the outer membrane leaflet using the “insertmolecules” tool in the GROMACS package. The system was placed in a cubic box, maintaining a uniform edge distance of 10 Å, and solvated with 7933 TIP3P water model to achieve physiological conditions. Subsequently, the system was neutralized by adding neutralizing Na+ and Cl − ions. Energy minimization of the system was conducted using 50,000 steps of steepest descent with a convergence tolerance of 1000 kJ/mol/nm. Then, 100 ns of MD simulations were conducted to equilibrate the two-step process under periodic boundary settings using NVT and NPT runs sequentially to fix the position of the head groups of the membrane lipids and peptide before the production simulation (N = constant number, V = constant volume, T = constant temperature, P = constant pressure). The system temperature and pressure were kept constant at 300 K and 1 bar 1.01325 bar using the Berendsen weak-coupling scheme. The CHARMM36 force field was used for the system with 2 fs time steps and a 1.4 nm cutoff for all interactions. The length of the production runs was set to 1000 ns.

## 2. Results

### 2.1. Identification of Cell-Penetrating Peptides (CPPs) in Influenza A (H1N1) proteins

We used many different bioinformaticdrug tools to examine the whole influenza A virus (H1N1 subtype) proteome to identify possible CPPs. The sequences of key influenza proteins are listed in Table 1. First, we used the CellPPD server to search for a set of influenza virus protein sequences in the FASTA format and identify possible cell-penetrating peptides (CPPs). A group of peptides had a strong ability to enter cells, as indicated by their high scores in the SVM assessment. Accordingly, the PB1 protein showed the highest number of identified CPPs. NS2 protein had the lowest number of identified CPPs, and only a limited number of peptides met the CPP criteria. The CPPs predicted by the CellPPD server were then sent to the C2Pred server for further evaluation. We observed minor variations in the predictions generated by the two bioinformatic tools. For instance, CellPPD classified the GPIYRRVNGK peptide, originating from nucleoprotein (NP), as a CPP with an SVM score of 0.22. However, C2Pred placed this peptide in the non-CPP category, giving it a probability score of 0.387432, which is below the 0.5 level required for classification as CPP. To further validate the results, we used the PreTP-EL web server to evaluate the predicted CPPs by targeting an additional validation layer. Accordingly, the PreTP-EL server works differently from the C2Pred server. For example, GPIYRRVNGK was marked as a non-CPP by C2Pred but a CPP by PreTP-EL. With a score of 0.3316, it remains below the cut-off threshold of 0.5 for CPP classification. S1 File shows all the known CPPs, along with their SVM score and the chance that they occurred from all three servers: CellPPD, C2Pred, and PreTP-EL. Table 2 presents the top-performing CPPs selected in this study, highlighting their physicochemical properties, cellular localization, and uptake efficiency, which are crucial for evaluating their potential for cargo delivery.

**Table 1:** Influenza A (H1N1) genome segments and encoded proteins screened for CPPs. For each RNA segment, the nucleotide length (nt), protein product(s) (name and abbreviation), and NCBI RefSeq accession number are listed. The entries collectively span the entire influenza A/H1N1 proteome used for CPP screening.

RNA Segment	nucleotide Length	Protein	Accession Number
PB2	2341	PB2 (Polymerase Basic 2)	NP_040987.1
PB1	2341	PB1 (Polymerase Basic 1)	NP_040985.1
		PB1-F2 (Polymerase Basic 1 Frame 2)	YP_418248.1
PA	2233	PA (Polymerase Acid)	NP_040986.1
		PA-X	YP_006495785.1
HA	1775	HA (Hemagglutinin)	NP_040980.1
NP	1565	NP (Nucleoprotein)	NP_040982.1
NA	1409	NA (Neuraminidase)	NP_040981.1
M	1027	M1 (Matrix Protein 1)	NP_040978.1
		M2 (Matrix Protein 2)	NP_040979.2
NS	890	NS1 (non-structural protein 1)	NP_040984.1
		NEP (Nuclear Export Protein, formerly NS2 non-structural protein 2)	NP_040983.1

**Table 2:** Physicochemical and predictive properties of the five selected CPP candidates from influenza A/H1N1. For each peptide, the epitope sequence and source protein are listed together with CellPPD SVM score*, C2Pred*, PreTP-EL*, MLCPP uptake probability*, hydrophobicity (Hphob)ᵃ, hydropathicity (Hpath)^b^, amphipathicity (Amph)^c^, hydrophilicity (HPhil)^d^, net chargeᵉ, isoelectric point (pI), molecular weight, Boman index, TMHMM-predicted cellular localization^f^ with N-in total probability^g^, IEDB score*, and estimated half-life in E. coli (HL1) and in mammalian cells (HL2). All five peptides score as CPPs across multiple tools and share favorable features—cationic/amphipathic profiles, *predicted intracellular localization, favorable estimated half-lives, and non-toxic predictions. Notably, PB1-1/-2/-3 (PB1-derived) rank among the strongest.*

Epitope	Peptide name	SVM^*^	C2Pred^*^	PreTP-EL^*^	MLCPP^*^	Hphob^a^	Hpath^b^	Amph^c^	HPhil^d^	Charge^e^	pI	Mol wt	Boman index	Cellular localization^f^	Total probability (N-in)^g^	IEDB^*^	HL^1^	HL^2^
VRKTRFLPVA	PB2−1	0.09	0.42	0.17	0.51	−0.24	0.16	0.86	0.08	3	12.01	1186.6	2.01	inside	0.63	0.13	>10 h	100 h
**RGDTQIQTRR**	**PB1−1**	0.06	0.62	0.36	0.003	−0.69	−2.13	0.99	0.98	2	11.7	1230.49	6.38	inside	0.94	0.01	2 m	1 h
**QRKRRVRDNM**	**PB1–2**	0.26	0.62	0.24	0.22	−0.94	−2.63	1.47	1.56	4	12.01	1358.72	7.97	inside	0.97	0.13	10 h	0.8 h
**THFQRKRRVR**	**PB1–3**	0.53	0.41	0.14	0.16	−0.83	−2.23	1.62	1.03	5.5	12.48	1383.76	7.09	inside	0.95	−0.12	>10 h	7.2 h
DRFLRVRDQR	PB2−2	0.13	0.46	0.26	0.03	−0.75	−1.77	1.1	1.24	2	11.53	1360.67	7.07	inside	0.86	0.11	>10 h	1.1 h

#### Uptake efficiency analysis of identified CPPs.

To optimize the efficiency of the uptake of the identified CPPs, all identified CPPs in the initial stage were sent to the MLCPP server using the CellPPD web server. Table 2 and S1 File present the analysis results, listing the peptides and classifying their adsorption efficiencies. The results showed that peptides made from PB1 protein consistently showed high uptake efficiency. These peptides showed a significant presence of CPPs in preliminary analysis. This fits with what we already know, because PB1 contains peptides that have a good chance of exhibiting CPP activity. On the other hand, we also classified NS2-derived peptides, which demonstrated CPP-reducing potential in CellPPD, C2Pred, and PreTP-EL analyses, as low-absorption peptides at this stage. This finding highlights how different viral proteins can work differently with CPP and how important uptake efficiency is when determining how useful these peptides might be as a whole in therapy.

### 2.2. Physicochemical properties of CPPs

Multiple computational tools were used to ascertain the physicochemical characteristics of the identified CPPs, as outlined in the methods section. Table 2 and S1 File present the physicochemical properties of these peptides. Net charge is a critical factor in interactions with cell membranes, with most cell-penetrating peptides (CPPs) exhibiting a positive charge. This feature is crucial for interacting with negatively charged cell membranes, facilitating peptide penetration and the delivery of molecular cargo. One of these peptides, PB1−3, derived from the *PB1* polymerase gene, has a net charge of 5.50 and a high Bowman index of 7.09, which may interact more effectively with biological targets. The average hydropathicity was calculated using the CellPPD server. The results indicated that the peptides exhibited predominantly negative values. A negative GRAVY value indicated that the peptide exhibited hydrophilic characteristics, thereby enhancing its capacity to interact with water molecules. Cell penetrating peptides (CPPs) must be hydrophilic to dissolve and remain stable in water-based solutions, such as biological fluids. The peptides originating from PB2, PB1, M1, and PA-X proteins demonstrated the longest anticipated half-life in mammals. The *PB2* gene peptides PB2−1 and VSIDRFLRVR code for the RXXR sequence and are often linked to better membrane translocation and internalization. Among these CPPs, only one peptide from the *PB1* polymerase gene encodes PB1−1, an RGD sequence motif known for its cell-adhesion properties. Collectively, these results show a wide range of physicochemical properties, clearly indicating that the identified CPPs can cross membranes and interact with cell parts. This analysis highlights critical insights into peptide stability, solubility, and bioavailability, which are essential considerations for therapeutic delivery system applications.

### 2.3 Membrane-binding potential of CPPs

The ability of CPPs to interact with cellular membranes is essential for their efficacy in delivering therapeutic molecules to cells. The peptide PB1−2, which is derived from the PB1 protein, had the highest Boman index (7.97), indicating that it has a better ability to bind to cellular membranes and interact with cellular proteins. This elevated score suggests that the peptide may play a significant role in enhancing the membrane permeability, thereby facilitating the delivery of therapeutic molecules. Several peptides, particularly those from the *NS1*, *PB2*, and *PB1* polymerase genes, showed a strong ability to bind to membranes. Four CPPs from the *PB1* polymerase gene, one CPP from the *NS1* gene, and one CPP from the *PB2* gene had high Boman indices, indicating that they could interact strongly with membranes. In addition to Boman indices, the peptide PB1−1, which is linked to the *PB1* polymerase gene, had a Boman index of 6.38. The RGD motif mediates cell adhesion and is associated with integrin binding. Consequently, the PB1−1 peptide derived from the PB1 polymerase gene of influenza virus may exhibit membrane-binding capabilities and specific cell-targeting properties.

**Fig 1:**
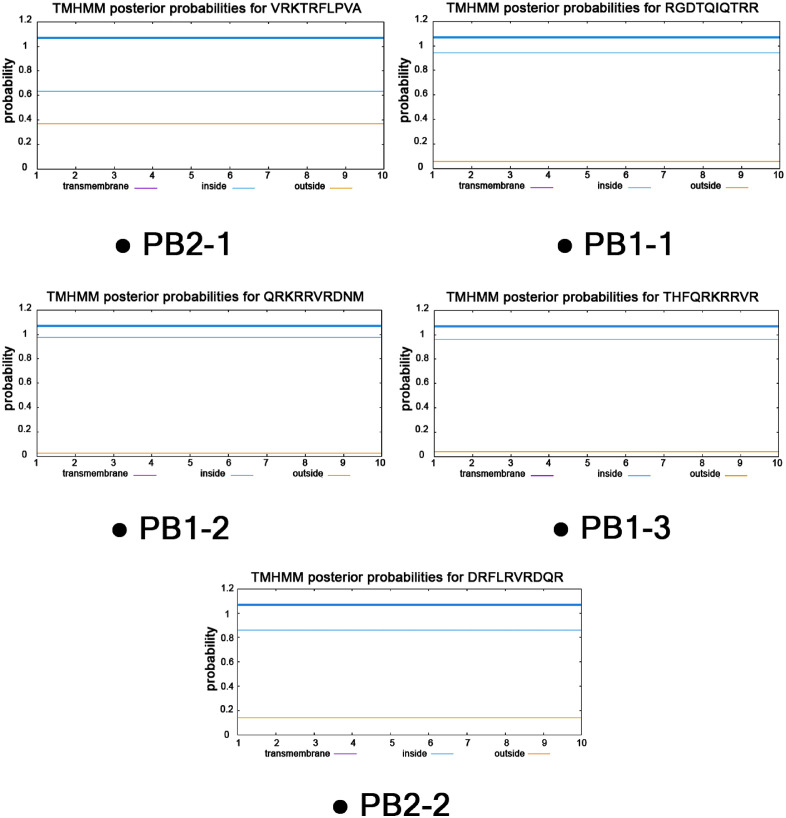
Cellular localization profiles predicted by TMHMM for the five selected influenza-derived CPPs. As shown by the probability plots, all peptides are predicted to localize on the cytoplasmic side of the membrane without any transmembrane domains, indicating their compatibility with membrane traversal. This result indicates that all five candidate CPPs can readily penetrate cells, supporting their potential role as effective CPP–cargo carriers for therapeutic delivery.

The TMHMM data also supported the Boman index results, showing that many of these peptides are likely to interact with or pass through the cell membranes, which supports their potential for therapeutic delivery. Notably, the *PB1* gene exhibited the greatest number of CPPs with the potential for membrane interactions among the listed genes. The *PA* gene predominantly predicted the extracellular localization of peptides, suggesting potential interactions with membrane proteins or the cell surface. The Boman index and TMHMM predictions show the different features of CPPs’ membrane binding and localization that were found in this study. Peptides with high Boman index values, especially those derived from the PB1, NS1, and PB2 genes, have great potential for use as carriers for therapeutic molecules. Table 2 and S1 File illustrate the specific results of the Boman index and TMHMM predictions. Some examples of the TMHMM prediction results are illustrated in Fig 1.

### 2.4. Immunogenicity assessment

We assessed the immunogenicity of each peptide using the IEDB immunogenicity predictor. We identified several CPP candidates that demonstrated binding with reduced immunogenic potential. Our analysis revealed that one variant from the polymerase *PB2* gene and another from the *M2* gene had the lowest predicted immunogenicity. Furthermore, we predicted low immunogenicity in two CPPs derived from the polymerase PA gene and in three from the polymerase PB1 gene (Table 2 and S1 File). These results are important because they show that peptides with low immunogenicity are less likely to elicit strong immune responses. This means that there is a lower chance of undesirable reactions, such as allergic reactions or peptide neutralization. Minimizing immune recognition enhances the likelihood that these CPPs will maintain their function as delivery vehicles, thereby reducing their chances of clearance or inactivation by the immune system.

### 2.5. Toxicity and allergenicity assessment

We assessed the toxicity and allergenicity of each CPP by using various web-based specialized tools. The results are summarized in S1 File. The ToxinPred web server classifies none of these CPPs as being toxic. This is significant because it suggests that none of the CPP candidates is likely to cause toxic effects, which is a crucial factor for their safe application in therapy. The lack of toxic peptides was a positive outcome, indicating that there were no toxicological concerns associated with the introduction of CPPs. Furthermore, allergenicity data emphasizes the need to employ various predictive tools. The distinctions between AllerTop and AllergenFP illustrate the challenges associated with accurately predicting the allergenic potential. The focus was on identifying non-allergenic CPPs on both platforms to minimize the risk of adverse immune responses, including hypersensitivity and allergic reactions. Non-toxic, non-allergenic CPPs are suitable candidates for therapeutic development because of their capacity to reduce the risk of adverse side effects, thereby improving their clinical feasibility.

### 2.6. Half-life estimation

The ProtParam web server was used to predict the half-life and instability index parameters of each peptide in *E. coli* and mammalian cells. We considered the instability index to be the primary criterion for assessing peptide stability. The peptides of mammalian cells had an estimated half-life of approximately 100 h and included proteins such as PB2, PB1, M1, and PA. Table 2 and S1 File show the sequences of VGRRATAILR, VNRANQRLNP, KKYTSGRQEK, VRKTRFLPVA, VSIDRFLRVR, VLVMKRKRDS, VLKRWRLFSK, VYWKQWLSLR, VKLYRKLKRE, and VSRRTSPALK. These results are important because peptide stability directly influences therapeutic efficacy to a great extent. In addition, peptides with longer half-life are less likely to be rapidly degraded by cellular proteases. In other words, they can remain active in biological environments for a longer time. The increased stability of these peptides also enhances their efficacy as vaccine carriers and therapeutic agents because of their prolonged interactions with biological targets. The highest stability index of the peptides was assessed for NP and PA proteins. The highest peptide stability index was assessed for the NP and PA proteins.

#### Evaluation of helix properties.

**Fig 2:**
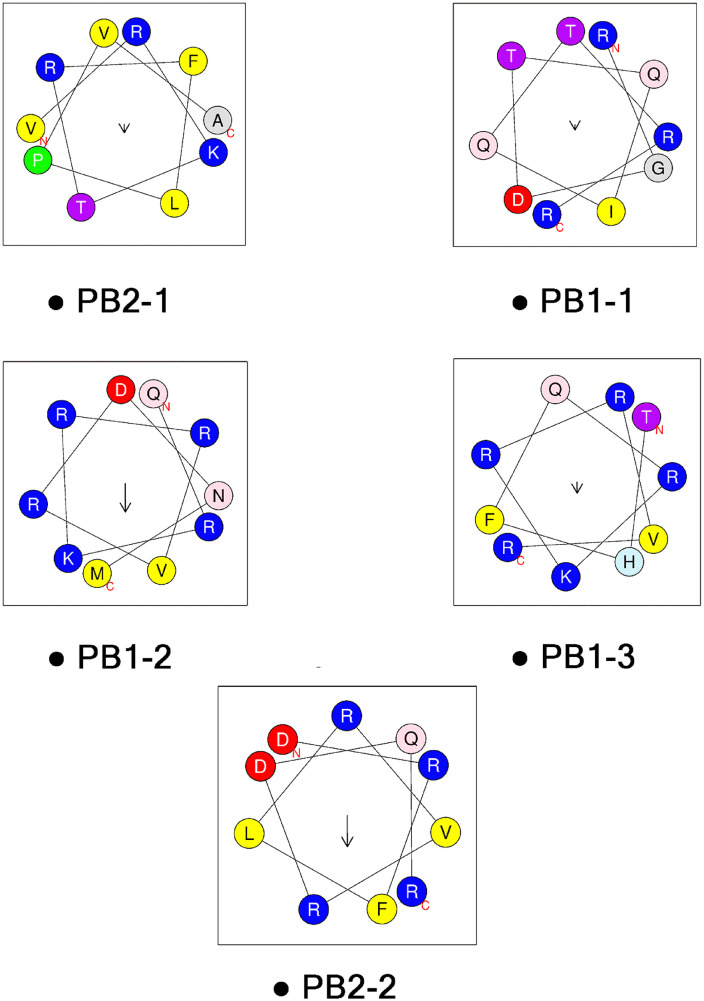
Helical-wheel diagrams (HeliQuest) of five CPP. Residues: hydrophobic (yellow), positive (blue), negative (red), nonpolar (pink/gray); black arrow is directed to hydrophobic moment. PB1−2/-3 and PB2−1/-2 are well-characterized amphipathic α-helices with an uninterrupted hydrophobic surface opposite to a cationic/polar surface, whereas PB1−1 lacks an uninterrupted hydrophobic area–in agreement with lower amphipathicity and lower capacity for direct membrane insertion.

The helical wheels of the short peptides identified as CPPs were analyzed using the Heliquest server. The helical wheel representation of a peptide is as follows. Each peptide sequence that includes a minimum of five contiguous hydrophobic residues, Leu, Ile, Ala, Val, Pro, Met, Phe, Trp, and Tyr, displays one hydrophobic face on the helical wheel. The results are shown in Fig 2.

### 2.7. Prediction of CPP structure

**Fig 3:**
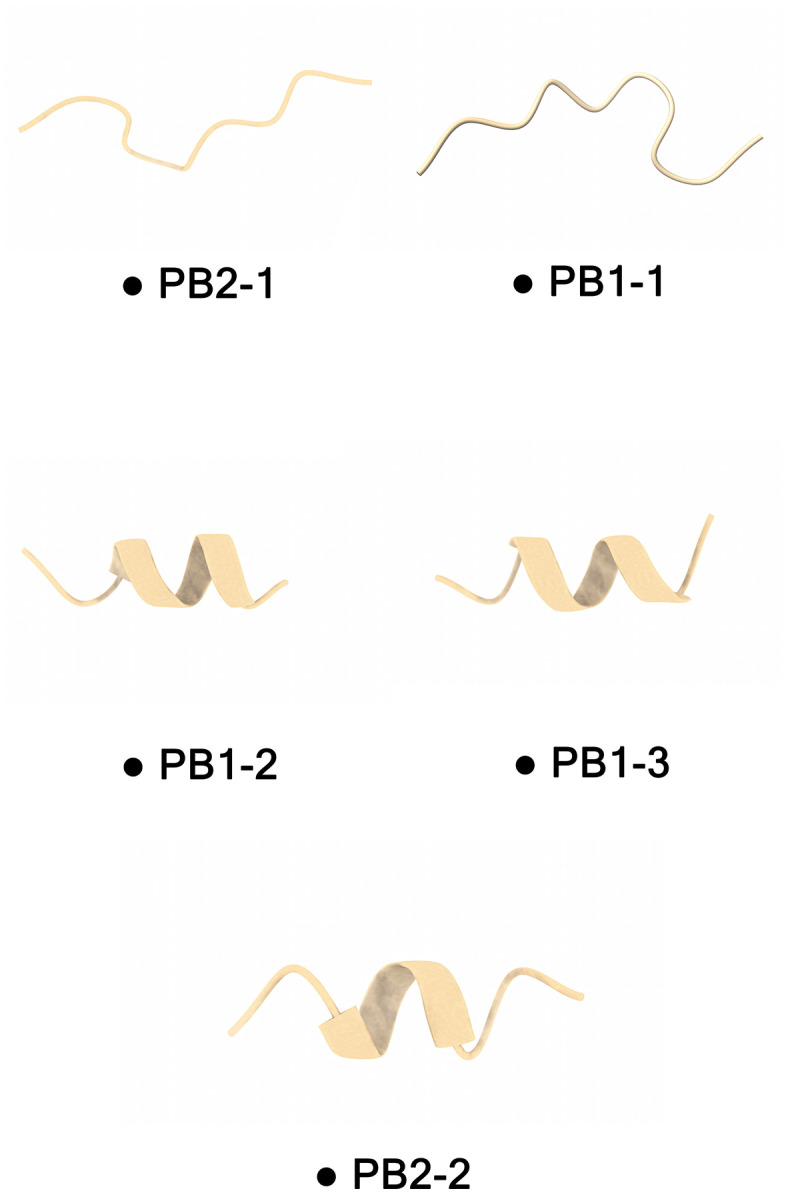
3D structures predicted under AlphaFold of five CPPs from influenza (all models pLDDT > 90). PB1−2, PB1−3, and PB2−2 show short α-helical segments, whereas PB1−1 and PB2−1 are largely coil-like. This pattern may suggest greater membrane partitioning/interaction for the helical peptides, while PB1–1/PB2–1 might rely more on receptor-mediated uptake; these are hypothesis-generating inferences given peptide length and AlphaFold’s limits on disordered peptides.

Following the CPPs 3D structure prediction by the Alphafold server, the pLDDT scores across all the models compared we identified those with the highest structural reliability (Fig 3). The results showed that all five selected peptides had high pLDDT scores (>90), indicating strong structural confidence and stability. These models offer valuable insights into the spatial arrangement and folding of peptides, which are critical for evaluating the membrane permeability and therapeutic delivery potential (S1 Fig).

**Fig 4:**
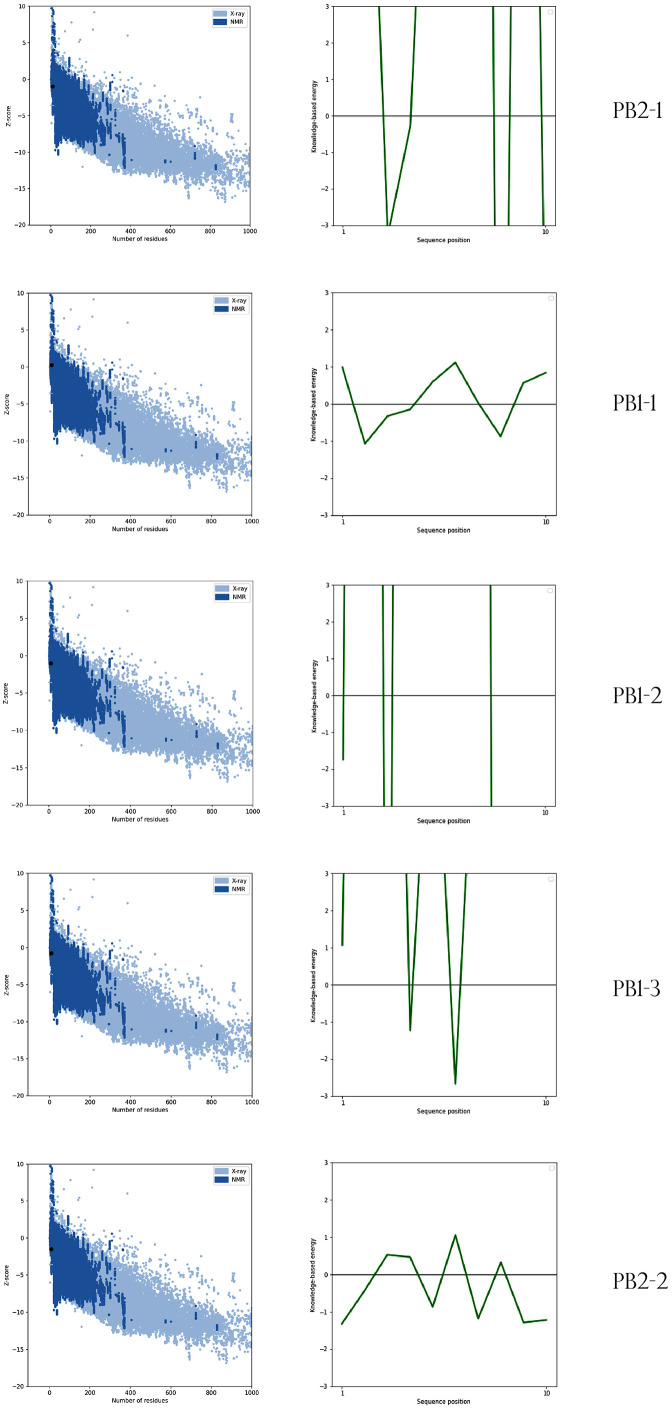
Verification of predicted structures of peptides using ProSA-web. In all five of the peptide models, the Z-score lies well within the range set by benchmark native protein structures solved by X-ray crystallography or NMR. This result confirms that all of these models are very reliable in terms of structural accuracy. Each of the peptides receives a Z-score within –0.24 to –1.52 that means that predicted structures are energetically favorable for additional biological interpretation.

The tertiary structure models were submitted to the GalaxyWeb server for refinement (Table 3). Validation was performed using ProSA-web servers. As shown in Table 4 and Fig 4, all five selected constructs showed negative Z-scores (ranging from −0.24 to −1.52).

**Table 3:** Structural quality metrics for refined models of the five selected CPPs (GalaxyRefiner results, CASP15 criteria). For each peptide, the top refined model’s evaluation scores are reported: GDT-HA (Global Distance Test High Accuracy), RMSD, MolProbity score, clashscore, % poor rotamers, and % Ramachandran-favored residues. Refinement improves 3D structural quality (higher GDT-HA, lower RMSD, fewer steric clashes/poor rotamers, and higher fractions of Ramachandran-favored residues). These metrics indicate that after refinement, all five peptide structures are of high 3D structural quality and reliability, suitable for detailed structural and functional analyses.

Model	GDT-HA	RMSD	MolProbity	Clash score	Poor rotamers	Rama favored
**Initial**	1.0000	0.000	3.852	20.0	33.3	62.5
**MODEL 1 of PB2−1**	0.8750	0.664	0.500	0.0	0.0	100.0
**MODEL 1 of PB1−1**	1.0000	0.236	2.151	10.5	0.0	87.5
**MODEL 2 of PB1–2**	0.646	0.700	2.684	5.2	11.1	87.5
**MODEL 3 of PB1–3**	0.8500	0.533	2.850	5.2	0.0	87.5
**MODEL 4 of PB2−2**	0.8750	0.530	0.500	0.0	0.0	100.0

**Table 4:** Z-scores from ProSA-web for AlphaFold/GalaxyRefine models of five influenza-derived CPPs. All models exhibit negative Z-scores in the native-structure range (more negatively = better), so these models are of acceptable quality to be used in dockings and molecular dynamics (MD) analysis; PB2−2 possesses the best value (−1.51) while PB1−1 possesses the worst (−0.24).

Peptide	Peptide name	Z-Score
VRKTRFLPVA	PB2−1	−0.98
QRKRRVRDNM	PB1–2	−1.02
RGDTQIQTRR	PB1−1	−0.24
THFQRKRRVR	PB1–3	−0.75
DRFLRVRDQR	PB2−2	−1.51

### 2.8. Molecular docking

**Fig 5:**
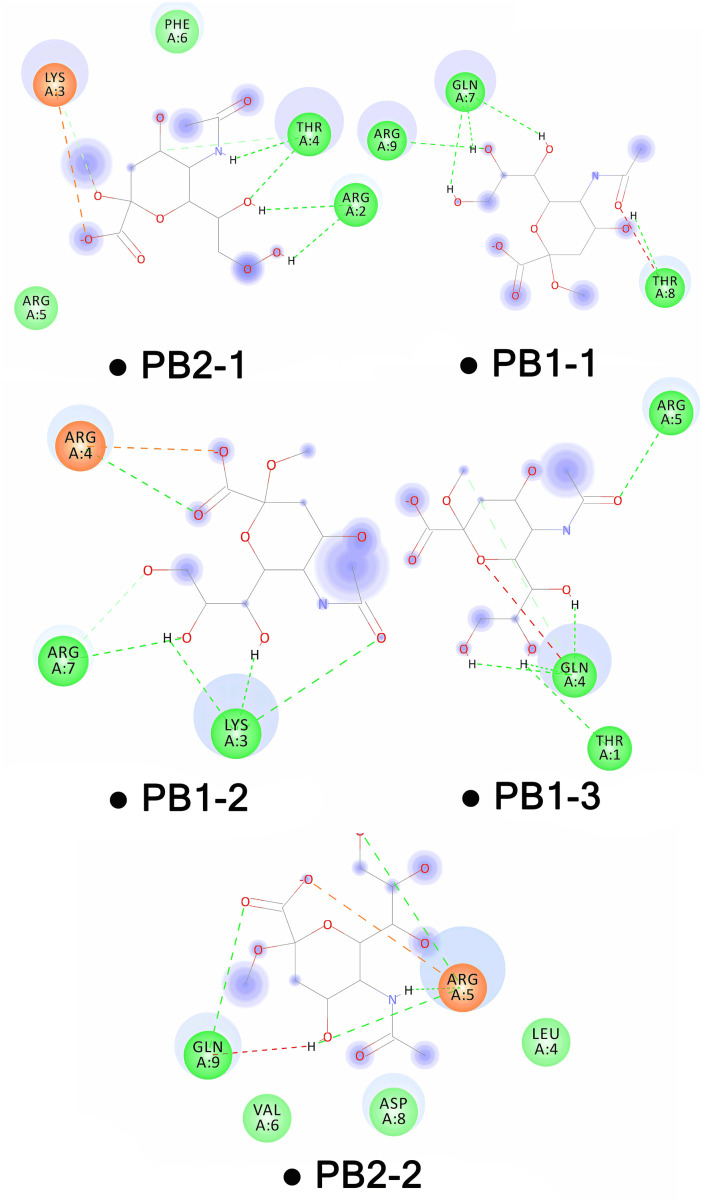
Represents HADDOCK 2D interaction maps of five CPP–LSTc complexes. Dashed lines indicate conventional hydrogen bonds (green), carbon–hydrogen bonds (gray), and salt links (orange). In both complexes, basic/polar residues (Arg, Lys, Gln, Thr) are predominant contacts. PB1−1 has the tightest interaction network, whereas PB2−1 has more dispersed contacts. Quantitative bond lengths and energies are listed in Tables 5–6.

Table 5 summarizes the binding affinities, electrostatic interactions, Van der Waals interactions, desolvation energies, and the HADDOCK scores. Table 6 details the interaction distances between the peptides and sialic acid, along with the residues involved. This docking study revealed that PB1−1 had the best binding affinity, with a ΔG of −8.16 kcal/mol, whereas PB1−3 had the least favorable at −7.37 kcal/mol. PB2−1 achieved the most favorable (lowest) HADDOCK score (−47.2 ± 3.7). While PB2−2 showed the least favorable HADDOCK score (−31.9 ± 5.2). In terms of interaction energies, PB1−2 had the highest electrostatic contribution of −156.9 ± 16.8 kcal/mol, while in the case of van der Waals energy, PB1−1 had the highest value of −29.9 + /- 1.9 kcal/mol. It showed that the favored peptide in terms of desolvation energy is PB2−1 at −6.0 ± 2.8 kcal/mol, while the least favored is PB1−2 with 8.4 ± 0.8 kcal/mol. The analysis also showed that PB1−1 had the greatest number of interactions, whereas PB1−2 formed the most number of hydrogen bonds. PB1−1 exhibited the shortest hydrogen bond distance between GLN5 (Glutamine 5) at 1.68 Å (Table 6 and Fig 5). Complementary docking was performed with HDOCK (distinct from HADDOCK). HDOCK ranked multiple candidate poses per CPP/LSTc complex by docking score; more negative values indicate better docking, and we retained the top-ranked (model 1) pose for each peptide. Docking scores for PB2−2, PB1−2, PB1−1, PB1−3, and PB2−1 were −122.63, −138.55, −110.05, −128.55, and −127.35, respectively; these model-1 poses were selected for further analysis.

**Table 5:** HADDOCK score summary for CPP–LSTc-peptide complexes. Lower (more negative) values indicate preferred docking. PB2-1 possesses the best HADDOCK score (−47.2 ± 3.7), while PB1-1 possesses the best ΔG (−8.16 kcal/mol) as well as strongest van der Waals term (−29.9 ± 1.9 kcal/mol).

Ligand	Peptide	Peptide name	ΔG (Kcal/mol)	Electrostatic energy	Van der Waals energy	Desolvation energy	HADDOCK score
*LSTc*	**VRKTRFLPVA**	PB2−1	−7.4749608	−73.0 + /- 20.4	−29.2 + /- 4.0	−6.0 + /- 2.8	−47.2 + /- 3.7
*LSTc*	**QRKRRVRDNM**	PB1–2	−7.9417469	−156.9 + /- 16.8	−22.3 + /- 2.1	8.4 + /- 0.8	−43.9 + /- 1.7
*LSTc*	**RGDTQIQTRR**	PB1−1	−8.1646437	−87.3 + /- 21.4	−29.9 + /- 1.9	3.7 + /- 1.8	−43.6 + /- 0.7
*LSTc*	**THFQRKRRVR**	PB1–3	−7.3705962	−103.7 + /- 31.8	−23.8 + /- 5.0	3.9 + /- 2.7	−38.9 + /- 2.8
*LSTc*	**DRFLRVRDQR**	PB2−2	−7.6959455	−55.9 + /- 15.6	−25.3 + /- 3.4	−1.0 + /- 2.9	−31.9 + /- 5.2

**Table 6:** Key contacts between CPPs and sialic acid (LSTc). This table reports, for each peptide, the contacting residues and their distances to sialic acid, resolved by interaction type (conventional H-bond, carbon H-bond, salt bridge). In every peptide, binding is mediated by polar and/or cationic residues—Lys and Arg (cationic) and Gln and Thr (polar, uncharged)—via hydrogen bonds and, where present, salt bridges.

Peptide	Residues interaction with SA	Peptide-Sialic acid interaction Distance Å		
		Conventional hydrogen bond	Carbon hydrogen bond	Salt bridge
PB2−1	LYS3	1.96	2.5, 2.53	–
PB1–2	GLN1	1.96	–	–
	ARG4	2.57	–	2.24
	ARG7	2.85	3.15	–
PB1−1	ARG1	–	–	1.67
	THR4	2.70	3.5	–
	GLN5	1.68	–	–
PB1–3	ARG10	1.86, 2.39	–	–
	LYS6	–	–	2.11
PB2−2	ARG2	–	3.77, 2.99	1.86

#### MD simulation.

**Fig 6:**
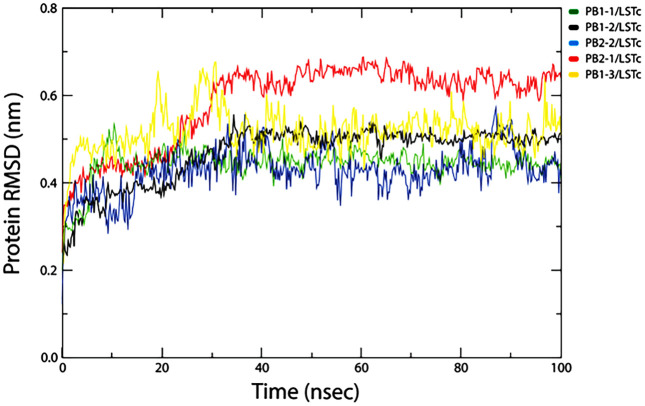
Shows time evolution of the backbone RMSD of five CPP–LSTc complexes over a 100-nanosecond MD simulation. Every curve represents root-mean-square deviation of a complex’s Cα atoms from its native structure. All complexes reach a steady plateau at around 30–40 nanoseconds, which implies that these complexes equilibrate and remain structurally stable over time of observation. Interestingly, PB1–1/LSTc and PB2–2/LSTc complexes have the least deviations of RMSD, reflecting a high relative conformational stability within the group.

To verify the convergence of the simulations and conformational stability of. The distribution of the RMSD for each system is shown in Fig 6. It is evident from Fig 6 that all CPPs- *LSTc* complex*es* show increasing behavior until ~ 40 ns, which may be due to the “relaxation” of the complex in the water environment, which is commonly observed in all MD simulation types and then presents a constant pattern of fluctuation up to 100 ns. In the case of complex PB2−1/ *LSTc*, relatively large RMSD value (0.5 ± 0.2) nm were observed compared to other complexes. except to PB2−1, other complex showed a lower degree of fluctuation within 0.2 to 0.5 nm of RMSD over 40–60 ns in simulation time highlights structural integrity and/or firm binding in CPPs- *LSTc* complexes. The average RMSD value was found to vary between (0.4 ± 0.3) Å for all complex.

#### Root mean square fluctuation (RMSF).

**Fig 7:**
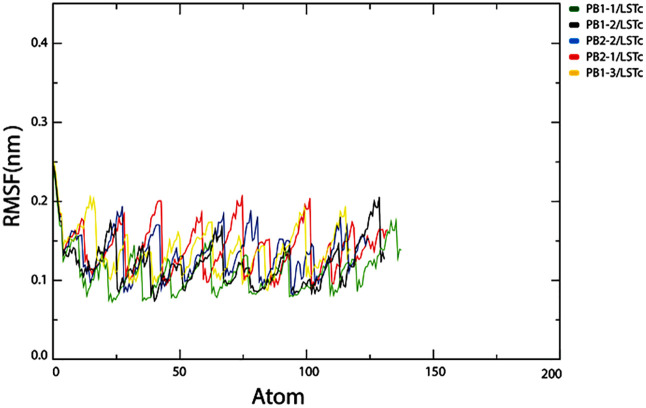
Illustrates root-mean-square fluctuations (RMSF) of CPP–LSTc complex’s Cα atoms from the molecular dynamics simulation. Per-residue RMSF is relatively low (<0.2 nm) across all five complexes, reflecting minimal flexibility across most regions with slight increases at termini. PB1−1 and PB2−2 exhibit relatively lower variations compared to PB2−1 with larger peaks, which is in agreement with Fig 6’s stability pattern.

The dynamic behavior of the CPPs LSTc complexes was further investigated by determining the residual fluctuations and root mean square fluctuations (RMSFs) of the Cα atoms in each complex. The high and low RMSF values indicate the flexibility and rigidity of our CPPs–LSTc complexes, respectively. Fig 7 shows a fluctuation value of less than approximately 0.2 nm for most of the Cα atoms, except for the N- and C-terminal residues. Residual fluctuations in regions around residues 20–30 and 60–70 were higher. The average RMSF values for the region of ligand-binding sites, including PHE3,LEU4 of PB2−2 CPP and VAL6,ASN9,MET10 of PB1−2 CPP and ARG10,HIS2,ARG7, and LYS6 of PB1−3 CPP, PRO8,ARG5,LYS3,ARG2 of PB2−1 CPP and ARG10,ARG9,GLN7,GLN5, and ARG1 of PB1−1 CPP, displayed the lowest fluctuation.

#### Radius of gyration (Rg).

**Fig 8:**
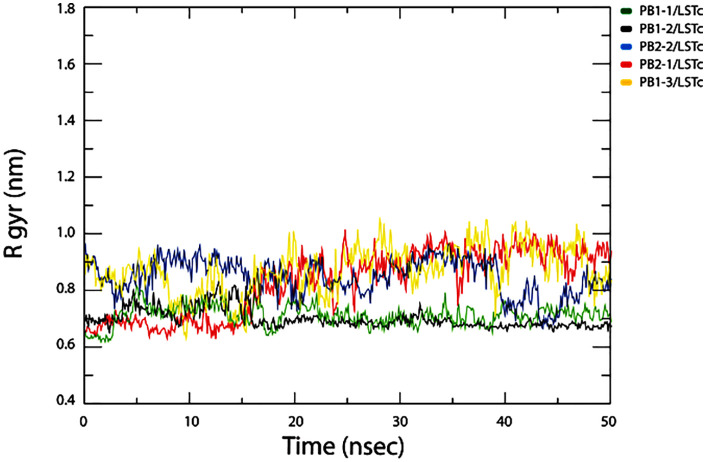
Radius of gyration (Rg) of each CPP–LSTc complex over a 50-ns MD simulation. Rg values for all five complexes remain comparatively steady throughout the simulation, showing no significant increase or unfolding event. Only minor Rg fluctuations occur, reflecting small dynamic adjustments but no loss of compactness. Among the complexes, PB1–1/LSTc maintains the lowest Rg (most compact structure) over time, indicating a particularly tight and stable complex formation.

Rg represents the total compactness manner and flexibility of the protein inside a biological environment over simulation time. Rg indicates protein structure variation during MD simulations, with higher Rg values indicating less compactness of the protein structure and lower Rg values indicating greater stability and compactness. Rg average values of CPPs–LSTc complexes was around 0.8 nm.The Rg results are in good agreement with that of RMSD and RMSF The Rg results are shown in Fig 8. Rg represents the total compactness and flexibility of the protein inside the biological environment over the simulation time.

#### Dynamics simulation of membrane penetration by the PB1−1 peptide.

**Fig 9:**
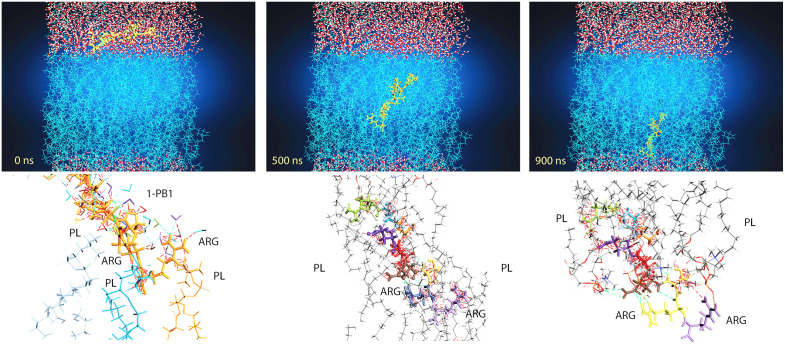
Time-resolved insertion of PB1−1 into a POPC bilayer (900-ns MD). Snapshots taken at 0, 500, and 900 ns illustrate the step-wise penetration of PB1−1 across the bilayer. In expanded insets, the arginines of PB1−1 form electrostatic as well as hydrogen-bonding contacts with lipid phosphohead groups, in effect securing the peptide. Together, these images support a step-wise mechanism of PB1−1 membrane insertion in which cationic groups always engage lipid headgroups to gain access to the hydrophobic core of the membrane commensurate with its membrane-penetrating ability.

**Fig 10:**
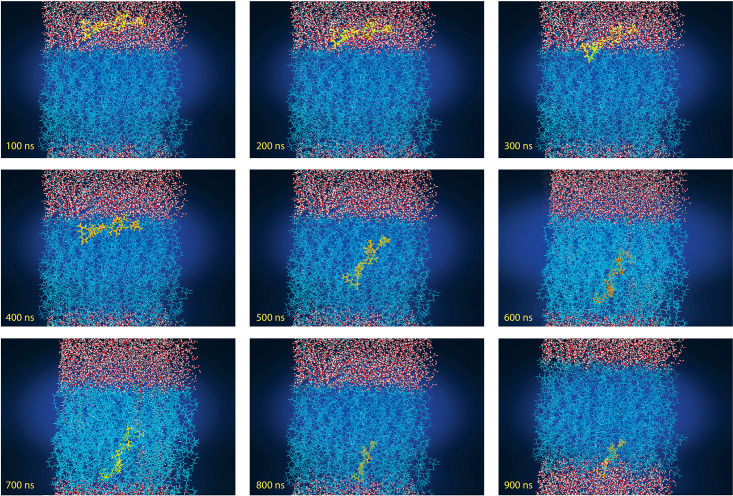
Trajectory of PB1−1 traversing through the POPC membrane (900-ns MD). A series of snapshots separated by 100 nanoseconds depicts PB1−1 binding to the outer leaflet, then gradually passing through the bilayer to ultimately reach the inner leaflet. Water molecules are used to simulate both sides’ aqueous environment. This trajectory is compatible with finding that PB1−1 is capable of passing through the model lipid bilayer in the timescale of the simulation.

**Fig 11:**
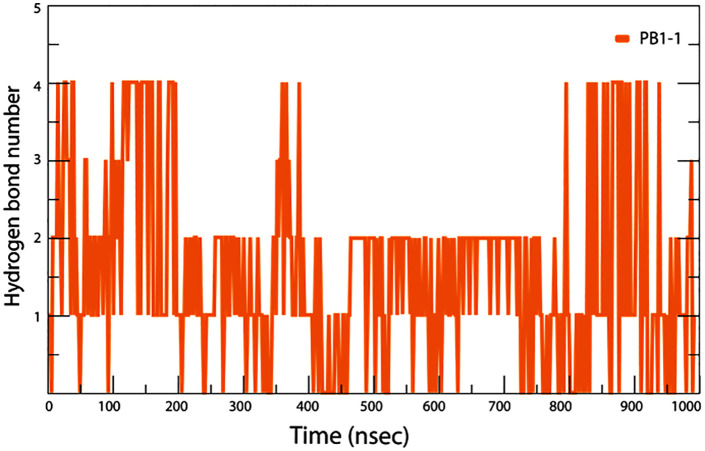
Illustrates the hydrogen-bond count between PB1−1 and the POPC membrane throughout a 1000-ns MD simulation. The curve reveals two significant peaks, which correspond to phases of heightened hydrogen bonding during the initial insertion into the membrane and subsequently as stabilization occurs within the bilayer. Between these peaks, the H-bond count experiences a dip, reflecting the breaking and reforming of bonds. This dynamic pattern of hydrogen bonding suggests that PB1−1 transiently establishes and exchanges hydrogen bonds as it penetrates, thereby facilitating its gradual entry and stable association within the depths of the bilayer.

As described in the previous sections, molecular dynamics simulations provide insight into the molecular behavior of CPPs when they cross through the membrane. PB1−1 contains one hydrophobic, three positively charged, and one negatively charged. The simulated membrane, composed of POPC lipids, consisted of a negatively charged phosphatidyl group, a positively charged choline group, and two fatty acid chains. The results show that PB1−1 with its C-terminus, specifically arginine 9,10, anchored to the membrane before sinking into the POPC membrane and connected to the surface with five hydrogen bonds and three salt bridges. PB1−1 has three critical cationic residues: arginine. It has a critical role in the membrane penetration of CPP by strongly interacting with negatively charged lipid phosphates [39]. These interactions, as shown in Figs 9 and 10, help PB1−1 to cross into the hydrophobic region of the POPC bilayer membrane,but polar residues tend to maintain interactions with the lipid head groups. Thus, both electrostatic and hydrophobic interactions are involved in PB1−1 crossing through the membrane. The aliphatic portion of the arginine 9,10 side chain and the Ile6 (Interleukin 6) residue play a key role in hydrophobic interactions with hydrophobic regions of the POPC bilayer at approximately 500 ns of simulation time. The number of H-bonds at the first and end points of the simulation due to the PIP3 water molecule showed an increased number of interactions compared to approximately 500 ns. PB1−1 CPP contains two glutamine (GLN) residues that feature an amide group in the side chain that acts as a hydrogen bond donor, which is known to be a key player in protein folding, stability, and interactions with other molecules. The insertion resulted in slight membrane thinning. Therefore, the charged residues of PB1−1, as shown in Figs 9 and 10, swapped the interacting partners with the head groups of the other leaflet, resulting in complete penetration. The Root Mean Square Deviation (RMSD) diagram of the α-carbon atoms of PB1−1 was plotted and compared to the 0 ns geometry of the trajectories The RMSD value changes indicated an unstable conformation of the system after the equilibration step. To gain further insight into the nature of the interactions between thePB1−1 and the membrane during 1000 ns simulations, the average number of hydrogen bonds between these groups was calculated and plotted, as shown in Fig 11. The results revealed that the PB1−1 residues formed frequent hydrogen bonds with the phosphate and ester groups of the POPC bilayer. The average number of H-bonds between the PB1−1 peptide and membrane was four during the first contact. Subsequently, the hydrogen bond number decreased to 2 until 500 ns of simulation, whereas it increased to 4 after 900 ns of simulation. This indicates a tendency for the change hydrogen bonds during membrane penetration by PB1−1. As shown in Fig 11, the number of hydrogen bonds between PB1−1 and the lipid head groups tends to form a double peak during penetration and crossing through the membrane. This may be due to the shift in the hydrogen bond partners of the PB1−1 to head groups of the outer and inner membrane leaflets. PB1−1 contact with the lipid tail also showed a single peak during penetration simulation. Further analysis showed that the hydrophobic contacts during the simulation became significantly high, which likely enabled the peptide to gain hydrophobic interactions during penetration. In addition, the salt bridge interaction between PB1−1 and the membrane showed the lowest number of interactions at approximately 500 ns of simulation time, but an increasing pattern of this interaction can be seen during the crossing of each leaflet. A different pattern of hydrophobic interactions than the salt-bridge interaction was observed over the simulation time. Table 7 presents the results. Owing to the limitations of the software, we were unable to track other interactions between the PB1−1 and membrane following the simulation process. A collection of snapshots capturing the trajectory of atoms for each 100 ns interval from 0 geometry coordinates to 900 ns was extracted and illustrated to provide a deeper insight into CPP penetration while crossing the POPC membrane, as shown in Fig 10.

**Table 7:** Time-dependent peptide–membrane interactions for PB1−1 within a POPC bilayer. The numbers of hydrogen bonds, salt bridges, and hydrophobic contacts are shown at 0, 500, and 1000 ns (4/3/2 → 2/1/8 → 4/4/3). In general, interactions become hydrophobic-dominated from polar-dominated at 0 ns to hydrophobic-dominated at 500 ns then revert back to polar dominance at 1000 ns.

Md time Step	Entry	Peptide	H-Bond	Salt Bridge	Hydrophobic
**0 ns**	1	PB1−1	4	3	2
**500 ns**	2		2	1	8
**1000 ns**	3		4	4	3

**Fig 12:**
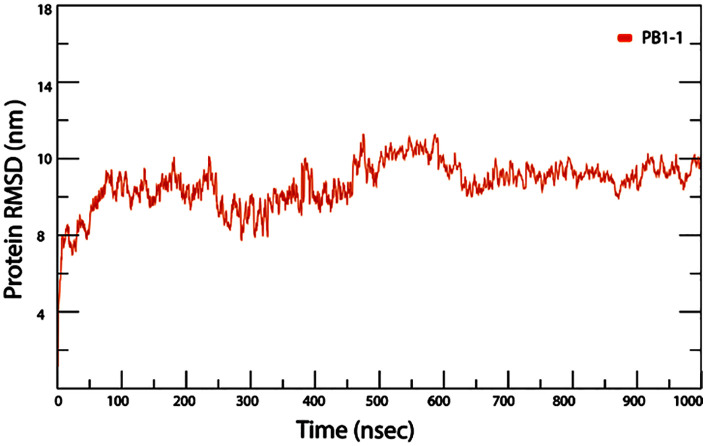
Represents the RMSD plot of PB1−1 peptide backbone, i.e., of its C α-atoms, during a 1000-nanosecond molecular dynamics trajectory. This plot highlights PB-1’s conformational stability throughout the course of simulation. Following a spike during equilibration, the RMSD of the peptide fluctuates within a medium range throughout the course of the simulation without exhibiting any extreme deviation. This low and constant RMSD supports that PB1−1 retains its conformational stability when placed within the membrane, unveiling the ability of the peptide to preserve its conformational integrity as well as membrane association on larger timescales.

To examine conformational changes in the peptide during penetration, MD trajectories generated by Gromacs were analyzed for structural changes, hydrogen bonding, hydrophobic contacts, and root mean square deviation (RMSD) of the alpha-carbon atoms. RMSD is considered a key indicator for assessing structural stability. The RMSD value was negatively correlated with peptide stability depending on the running time. A larger RMSD value indicated more unstable backbone atoms. The RMSD value, as shown in Fig 12, was calculated after relaxation/equilibration by comparing it to the 0 ns of the trajectories for further analysis. The RMSD at approximately 150 ns and 500 ns of simulation time represents the highest fluctuation, which may be due to the crossing of the membrane from the upper leaflet to the intermembrane space.

## Discussion

Successful vaccine development remains a top priority because of the inefficiency of immunization mechanisms against most viruses. Therefore, it is important to enhance intracellular antigen delivery systems, particularly for second-generation subunit and mRNA vaccines. Cell-penetrating peptides (CPPs) are potentially powerful non-viral vectors with low immunogenicity, scalability, and safety that can transport proteins, nucleic acids, and antigens directly into antigen-presenting cells (APCs), thereby supporting enhanced antigen presentation and strong immune responses [40–42]. CPPs (also known as protein transduction domains, PTDs) can cross cell membranes and form electrostatic complexes with negatively charged in vitro-transcribed mRNA (IVT-mRNA). These complexes protect the mRNA from nuclease degradation and facilitate cellular uptake, primarily via endocytosis [43–45]. In addition to non-covalent complexation, covalent strategies have also been explored—for example, conjugation of the a CPP peptide to IVT-mRNA significantly enhanced its serum stability and protein expression in human fibroblasts and stem cells (Miliotou et al., 2021) [46]. These findings underscore the potential of CPPs as effective non-viral carriers for intracellular mRNA delivery.

In addition to their general ability to translocate across membranes, CPPs can be created to bind to target receptors, such as alpha-sialic acid, for tissue-specific delivery, which is particularly useful in respiratory vaccine delivery. CPPs have already been shown effective receptor-mediated transcytosis across barriers like the blood-brain barrier and could similarly serve to deliver to lung epithelial cells [47]. Some CPPs have been identified in viruses such as HIV-1, SARS-CoV-2, Dengue, and HSV-1, which are usually essential for viral entry [13,48]. Bolhassani et al. (2021) employed CellPPD and C2Pred to search the SARS-CoV-2 proteome for novel CPPs with high uptake and low predicted toxicity [13]. Similarly, Hemmati et al. (2020) identified over 300 SARS-CoV-2 CPPs, some of which have dual functions in viral pathogenesis and therapeutic delivery [49]. HIV-1 CPPs have also been reported to be effective for intracellular delivery of therapeutic molecules [50], and machine learning approaches such as SVM, Random Forest, and ANN have also improved the accuracy of prediction and peptide design [51].

Bioinformatics tools are crucial for identification and characterization of CPPs for therapeutic delivery. In this study, we had two main objectives: (1) in silico screening of the entire Influenza A (H1N1) proteome for novel CPPs with good delivery potential, and (2) identification of their sialic acid binding capacity as this interaction is essential for influenza virus entry into lung epithelial cells. Targeting sialic acid achieves tissue specificity, which is a key factor in the development of lung-targeted vaccines. To this end, we extracted key influenza protein sequences, such as HA and NA, NP, NS1 and NS2-NEP, PA, PB1, and PB2, and matrix proteins M1 and M2 and submitted them to the CellPPD web server for CPP prediction. We identified a remarkable number of CPPs in the influenza proteome via in silico analysis, using CellPPD, C2Pred, PreTP-EL, and MLCPP [52–54]. Although the CellPPD server identified more CPPs at an SVM prediction threshold of 0.0, the C2Pred results provided a more conservative estimate, marking some peptides as non-CPPs, despite a low but positive SVM score. In contrast, the results of PreTP-EL were completely opposite to those of C2Pred, and the peptides identified as CPP by C2Pred were identified as non-CPP by PreTP-EL. This underscores the need for further experimental validation to confirm the true CPP activity of the identified candidates. We then used the MLCPP server to evaluate the uptake efficiency and cell membrane penetration of CPPs. Next, we characterized the physicochemical properties and calculated the membrane-binding potential with cellular localization using the Boman index and TMHMM web server. The immunogenic properties of each CPP were evaluated using the IEDB server, as well as the toxicity and allergenicity properties of each CPP, and the half-life and stability index of each CPP were examined using the ParatParam server. These CPPs were classified based on the presence of motifs, such as tumor-homing motifs (RGD) and tumor-penetrating motifs (RXXR) [13]. Our results indicate that the influenza virus proteome contains a significant number of CPPs, primarily originating from the polymerase PB1 protein. Furthermore, we identified 11 CPPs in HA and twelve in NA, both of which are influenza surface proteins. Many CPPs found in HA and NA have amphipathic properties that facilitate membrane interactions. The most efficient uptake was observed for the CPPs made from polymerase PB1. The exceptionally high uptake efficiency of PB1-derived peptides was likely due to their arginine and tryptophan residue content, as well as their potential for forming amphipathic helices. The disposition of these residues in the helix is accountable for interaction with the membrane. However, it must be noted that peptides with a high hydrophobic moment (μH) and pore-forming capacity can also irreversibly destabilize lipid bilayers, leading to cytotoxicity. Therefore, μH should be optimized with caution must be done to obtain a balance between efficacy and safety. Hence, μH should be minimized to reduce the membrane disruption caused by CPPs [55]. Subsequent analysis indicated that all predicted CPPs derived from the PB1 protein exhibited nontoxic characteristics; most were non-sensitizing, possessed a favorable half-life, and demonstrated a high affinity for binding to plasma membranes, facilitating cellular penetration. PB1-derived CPPs may facilitate viral entry into host cells. Among the CPPs derived from PB1, PB1–1 contained an RGD motif. The TMHMM server predicted the cellular localization of this CPP, which had a net charge of +2.00 and was nontoxic, with an immunogenicity score of approximately 0.01702 and a half-life of one hour in mammalian cells. Additionally, two CPPs (PB2–1 and PB2–2) featured an RXXR motif in their N- and C-terminal regions, respectively. PB2–1, with a charge of +3.00, is non-toxic, allergenic, has an immunogenicity score of approximately 0.13778, and has a half-life of approximately 100 hours in mammals. PB2–2, with a charge of +2.00, is non-toxic, non-allergenic, has an immunogenicity score of approximately 0.1172, and has a half-life of 1.1 hours. In addition, the Boman index for PB2–2 was 7.07, indicating a strong binding potential. Furthermore, the cpp derived from PB1 PB1–2, which had a net charge of +4.00, was found to be non-toxic, exhibiting an immunogenicity score of approximately 0.13634 and a half-life of 0.8 hours in mammalian cells. The TMHMM web server revealed the subcellular localization of PB1–2, which had the highest Bowman index among the predicted CPPs (7.97), indicating a very strong binding potential. Furthermore, the PB1-derived CPP PB1–3 demonstrated no toxicity, with an immunogenicity score of approximately −0.12164, and a half-life of 7.2 hours in mammalian cells. The net charge for PB1–3 was + 5.50, which was the highest among the predicted CPPs and thus showed strong binding and cell membrane penetration.

Based on these physicochemical findings, targeting specific receptors, such as alpha-sialic acid, is a modern strategy for tissue-specific delivery. We performed molecular docking using superior CPPs derived from influenza polymerase proteins (PB1 and PB2). In the present study, we attempted to elucidate the ability of CPPs to interact with sialic acid, and thus, their potential for efficient internalization and targeting of sialic acid to lung tissue. We used LSTc, a sialylated glycan analog, to model the initial attachment of influenza-derived CPPs to airway cell receptors, as the human respiratory epithelium is rich in α2,6-linked sialylated glycoconjugates that serve as influenza virus receptors [56]. The results obtained for the interaction of the five peptides with LSTc indicated considerable variation in their interaction profiles, stabilities, and binding affinities. The peptides were evaluated by considering the HADDOCK scores, binding energies, and interaction types with the receptor. For HADDOCK, more-negative scores are more favorable. Correspondingly, in terms of the HADDOCK scores, PB2–1 had the most favorable HADDOCK score (−47.2 ± 3.7), which was closely followed by PB1–2 (−43.9 ± 1.7) and PB1–1 (−43.6 ± 0.7). These scores indicate stable CPP–LSTc complexes. On the other hand, PB2–2 showed less stability. Based on the binding affinities depicted by their ΔG values, PB1–1 is the best candidate because its ΔG is favorable (−8.16 kcal/mol), which indicates that it creates a very strong and thermodynamically stable interaction with the sialic acid-rich receptor. PB1–2 (ΔG = −7.94 kcal/mol) also formed a stable complex. Other peptides had relatively negative binding energies, PB1–3 (−7.37 kcal/mol) and PB2–1 (−7.47 kcal/mol), which suggested that they were moderately stable. PB2–2 had a ΔG of −7.69 kcal/mol, which also indicated stable but comparatively weaker binding. Further insights into binding dynamics were obtained through the analysis of molecular interactions. PB1-1 forms hydrogen bonds with Gln5 and Thr4, and a salt bridge with Arg1. The hydrogen bond distances ranged from 1.68 to 3.5 Å, supported by a salt bridge with ARG1 at 1.67 Å distance. This strong network of interactions explains its superior binding affinity and stability. In contrast, PB1–2 had established important interactions with GLN1, ARG4, and ARG7. It binds via a combination of hydrogen bonds and electrostatic interactions, contributing to its high binding affinity, although the PB1–2 has a more favorable HADDOCK score than PB1–3. In contrast, peptide PB2–2 exhibits two longer carbon H-bonds and one salt bridge (1.86 Å); together with its HADDOCK score (−31.9 ± 5.2), it ranks as the least favorable complex. Overall, PB1–1 is top by ΔG and interaction network, whereas PB2–1 leads by HADDOCK; PB1–2 is consistently strong across both metrics. PB1–3 is also promising because of the variety of interactions it may form, although its ΔG is slightly higher than those mentioned here. We tested the ability of CPPs 1–5 to interact with LSTc, a sialic acid analog present on the membrane surfaces. Among these candidates, PB1–1 stands out mechanistically. As one of the most hydrophilic peptides in its class, PB1–1 significantly enhances its potential, mostly because of three arginine residues present in it, which interact with the negatively charged phosphate groups of membrane phospholipids. The positive charges of arginine aid hydrogen bond interactions as well as membrane translocation, both of which are important parameters in the functionality of CPPs [27]. Arginine arrangement supports membrane interaction. [57]. As shown in Figs 9 and 10, the addition of two arginine residues at PB1–1’s C-terminal portion increases its adhesion potential with negatively charged phosphate groups of phosphatidylcholine in the POPC membrane. In addition, PB1–1 contains two glutamine side groups whose amide groups can act as hydrogen bond donors. Such sequences form hydrogen bond contacts with membrane acyl tails under simulated molecular dynamics structures, mostly at approximately 500 ns. This result is in agreement with a previous study that reported that guanidino groups are primarily responsible for assuring the internalization of such CPPs [58]. In summary, such mechanistic studies show that PB1–1 is the most promising candidate with potential for implementation in vaccines and antigen delivery. However, we acknowledge that binding to an isolated glycan (LSTc) is an idealized scenario with important limitations. Although glycan binding may facilitate surface attachment, it does not necessarily imply efficient cellular uptake [59,60]. Such binding highlights a possible uptake mechanism (glycan-mediated docking), but does not prove that such binding drives cellular entry into real airway tissue. Experimental validation will be crucial—e.g., glycan blocking assays or cell uptake studies—to confirm whether the observed CPP–LSTc interactions lead to meaningful receptor-mediated uptake in airway epithelial cells. Given these limitations, we emphasize the need for in vitro and in vivo follow-up studies. To determine the novelty and translational potential of the identified candidate CPPs, we performed BLAST alignments against two reference databases: CPPsite 2.0, representing experimentally confirmed CPPs, and THPdb, representing FDA-approved therapeutic proteins and peptides. The lack of significant sequence homology confirms the novelty of the peptides discovered here, and that they have not been previously reported in clinical or CPP contexts. These results demonstrate their potential as new candidates for intracellular delivery applications [61,62]. Compared to canonical CPPs, the PB1-derived peptides occupy an intermediate physicochemical niche. HIV-TAT is a very short, highly basic (≈+8) non‐amphipathic CPP whose uptake requires ~8 arginine/lysine residues. whereas penetratin (16 amino acids, ≈+7) is a known amphipathic peptide [63]. Because CPPs are generally cationic at physiological pH, they bind to anionic membranes; accordingly, PB1–2 and PB1–3 (net charges +4 and +5) are moderately cationic and are predicted to form short amphipathic helices that juxtapose hydrophobic and positively‐charged faces. Such amphipathic helices are known to insert into membranes via their hydrophobic face while using the cationic face to bind negatively charged cargo [64].

In particular, arginine/lysine-rich CPPs can electrostatically condense nucleic acids (and other anionic cargos) into stable complexes [64,65]. Consistent with this mechanism, Comparable strategies involving SV40 NLS and WSQP spacers improved CPP-mediated delivery in previous studies [66]. In contrast, PB1−1 (net charge +2) is much less cationic and hydrophobic, suggesting it would bind nucleic acids only weakly. Its RGD motif instead implies integrin-mediated uptake, which could favor delivery of protein or peptide antigens. Together, these features support efficient, low-toxicity intracellular delivery and make PB1−1 a promising candidate for tissue-specific CPP–cargo delivery strategies (e.g., cancer-targeted immunotherapies) [13]. Thus, PB1−2 and PB1−3 are predicted to be especially effective for anionic cargos (e.g., DNA/RNA), whereas PB1−1 may preferentially carry proteinaceous cargo via receptor targeting. These structure–function hypotheses could be tested by synthesizing the peptides and comparing their binding and cell uptake of model nucleic acid versus protein cargos in future delivery assays. Contemporary reviews agree that CPP uptake proceeds primarily via two routes—energy-dependent endocytosis and energy-independent direct translocation—with pathway usage dictated by sequence, cargo, and cell context [67]. Cationic CPPs carrying nucleic acids tend to enter predominantly by endocytosis (macropinocytosis, clathrin/caveolae-mediated), after which endosomal escape becomes rate-limiting; in contrast, highly amphipathic/arg-rich peptides at sufficient local concentration can exhibit partial direct translocation across the plasma membrane [63,68]. Consistent with this, Deshayes et al. (2005) highlighted that CPPs with a net positive charge, amphipathic character, and α-helical conformation can directly translocate across lipid bilayers through non-endocytic mechanisms, including transient pore formation and inverted micelle structures [65]. Recent studies emphasize that classical cell-penetrating peptides (CPPs) with high positive charge and amphipathic α-helices efficiently bind and transport nucleic acids, whereas lower-charge peptides bearing RGD motifs (e.g., PB1−1) tend to engage integrin-mediated, receptor-dependent uptake [63]. Our findings on PB1−1 are in line with the description of some virus-derived CPPs that effectively penetrate host cells by preferentially targeting nuclear localization signals for cellular internalization [69]. Docking results suggest that PB1−1 may preferentially interact with sialic acid–rich receptors, implying potential for lung-specific targeting, though this remains to be tested experimentally. This suggests that PB1−1 may represent a promising peptide for respiratory vaccine delivery pending experimental validation. The specific hydrogen bond interactions and low binding energy of this peptide suggest that it can easily enter lung cells via sialic acid-based pathways. Modification of CPP sequences to include hydrophilic residues and charged modifications have been shown to improve cellular uptake and binding affinity.

Optimizations, including the addition of nuclear localization signals (NLS) or other trafficking motifs, may be beneficial for PB1−1. Nevertheless, these computational predictions remain hypothetical and require validation using experimental assays [70]. Future studies will experimentally validate how the physicochemical traits of the identified CPPs—charge, hydrophobicity, and amphipathicity—govern delivery efficiency across diverse cargo types. Systematic CPP variants will be tested with small molecules, nucleic acids, and proteins to define property–cargo relationships. Quantitative uptake and cytosolic delivery assays will clarify the mechanistic basis of peptide–cargo–membrane interactions. These efforts will guide rational design of optimized, cargo-specific influenza-derived CPPs for targeted delivery applications.

## Conclusion

Together, our integrative in silico approach enabled the identification of a novel CPP, PB1−1, with double functional activity for penetration across the membrane and lung-specific delivery by sialic acid binding. Its structural and biophysical properties, complemented by molecular docking and molecular dynamics, make PB1−1 an ideal candidate for respiratory vaccine delivery. Further in vitro and in vivo studies are warranted to validate its therapeutic use.

## Supporting information

S1 File In silico prediction and comprehensive characterization of predicted cell-penetrating peptides (CPPs) derived from influenza A (H1N1) virus. (DOCX)

S1 Fig AlphaFold-based 3D structure predictions and confidence scores for the selected CPPs. The predicted structural depictions for the selected five CPPs are shown next to confidence values as represented by the pLDDT (predicted Local Distance Difference Test) score. All peptides had significantly high pLDDT values (above 90), indicating strong confidence in the accuracy and stability of the predicted conformations. The confidence range color-coding scheme employed by AlphaFold was shown for identification purposes.
(PNG)

## References

[1] Sánchez-Navarro M. Advances in peptide-mediated cytosolic delivery of proteins. Adv Drug Deliv Rev. 2021;171:187–98. doi: 10.1016/j.addr.2021.02.003 33561452

[2] Langel Ü. CPP, cell-penetrating peptides. Springer; 2019.

[3] Taylor RE, Zahid M. Cell penetrating peptides, novel vectors for gene therapy. Pharmaceutics. 2020;12(3):225. doi: 10.3390/pharmaceutics12030225 32138146 PMC7150854

[4] Dowaidar M. Uptake pathways of cell-penetrating peptides in the context of drug delivery, gene therapy, and vaccine development. Cell Signal. 2024;117:111116. doi: 10.1016/j.cellsig.2024.111116 38408550

[5] Xie J, Zhang P, Crite M, DiMaio D. Papillomaviruses Go Retro. Pathogens. 2020;9(4):267. doi: 10.3390/pathogens9040267 32272661 PMC7238053

[6] Kalafatovic D, Giralt E. Cell-penetrating peptides: Design strategies beyond primary structure and amphipathicity. Molecules. 2017;22(11):1929. doi: 10.3390/molecules22111929 29117144 PMC6150340

[7] Porosk L, Gaidutšik I, Langel Ü. Approaches for the discovery of new cell-penetrating peptides. Expert Opin Drug Discov. 2021;16(5):553–65. doi: 10.1080/17460441.2021.1851187 33874824

[8] Langel Ü. Methods for CPP selection, prediction and in silico analysis. CPP, Cell-Penetrating Peptides. Springer. 2023. p. 83–94.

[9] Manavalan B, Subramaniyam S, Shin TH, Kim MO, Lee G. Machine-learning-based prediction of cell-penetrating peptides and their uptake efficiency with improved accuracy. J Proteome Res. 2018;17(8):2715–26. doi: 10.1021/acs.jproteome.8b00148 29893128

[10] de Oliveira ECL, Santana K, Josino L, Lima E Lima AH, de Souza de Sales Júnior C. Predicting cell-penetrating peptides using machine learning algorithms and navigating in their chemical space. Sci Rep. 2021;11(1):7628. doi: 10.1038/s41598-021-87134-w 33828175 PMC8027643

[11] Yamamoto Y, Tamiya S, Shibuya M, Nakase I, Yoshioka Y. Peptides with the multibasic cleavage site of the hemagglutinin from highly pathogenic influenza viruses act as cell-penetrating via binding to heparan sulfate and neuropilins. Biochem Biophys Res Commun. 2019;512(3):453–9. doi: 10.1016/j.bbrc.2019.03.068 30904159

[12] Dadonaite B, Gilbertson B, Knight ML, Trifkovic S, Rockman S, Laederach A, et al. The structure of the influenza A virus genome. Nat Microbiol. 2019;4(11):1781–9. doi: 10.1038/s41564-019-0513-7 31332385 PMC7191640

[13] Kardani K, Bolhassani A. Exploring novel and potent cell penetrating peptides in the proteome of SARS-COV-2 using bioinformatics approaches. PLoS One. 2021;16(2):e0247396. doi: 10.1371/journal.pone.0247396 33606823 PMC7894964

[14] Tang H, Su Z-D, Wei H-H, Chen W, Lin H. Prediction of cell-penetrating peptides with feature selection techniques. Biochem Biophys Res Commun. 2016;477(1):150–4. doi: 10.1016/j.bbrc.2016.06.035 27291150

[15] Guo Y, Yan K, Lv H, Liu B. PreTP-EL: prediction of therapeutic peptides based on ensemble learning. Brief Bioinform. 2021;22(6):bbab358. doi: 10.1093/bib/bbab358 34459488

[16] Manavalan B, Subramaniyam S, Shin TH, Kim MO, Lee G. Machine-Learning-based prediction of cell-penetrating peptides and their uptake efficiency with improved accuracy. J Proteome Res. 2018;17(8):2715–26. doi: 10.1021/acs.jproteome.8b00148 29893128

[17] De Cena GL, Scavassa BV, Conceição K. In silico prediction of anti-infective and cell-penetrating peptides from thalassophryne nattereri natterin toxins. Pharmaceuticals (Basel). 2022;15(9):1141. doi: 10.3390/ph15091141 36145362 PMC9501638

[18] Chen L, Guo X, Wang L, Geng J, Wu J, Hu B, et al. In silico identification and experimental validation of cellular uptake by a new cell penetrating peptide P1 derived from MARCKS. Drug Deliv. 2021;28(1):1637–48. doi: 10.1080/10717544.2021.1960922 34338123 PMC8330795

[19] Kuriakose A, Chirmule N, Nair P. Immunogenicity of biotherapeutics: Causes and association with posttranslational modifications. J Immunol Res. 2016;2016:1298473. doi: 10.1155/2016/1298473 27437405 PMC4942633

[20] Pant P, Sehgal A, Gupta T, Sharma P. Identification of potent cell penetrating, nontoxic peptides for inhibiting MDM2-p53 Interactions: Characterization of anticancer peptides via molecular dynamics simulations. J Pept Sci. 2025;31(7):e70033. doi: 10.1002/psc.70033 40396628

[21] Hemmati S, Rasekhi Kazerooni H. Polypharmacological cell-penetrating peptides from venomous marine animals based on immunomodulating, antimicrobial, and anticancer properties. Mar Drugs. 2022;20(12):763. doi: 10.3390/md20120763 36547910 PMC9787916

[22] Maleš M, Juretić D, Zoranić L. Role of peptide associations in enhancing the antimicrobial activity of adepantins: Comparative molecular dynamics simulations and design assessments. Int J Mol Sci. 2024;25(22):12009. doi: 10.3390/ijms252212009 39596078 PMC11593906

[23] Baek M, DiMaio F, Anishchenko I, Dauparas J, Ovchinnikov S, Lee GR, et al. Accurate prediction of protein structures and interactions using a three-track neural network. Science. 2021;373(6557):871–6. doi: 10.1126/science.abj8754 34282049 PMC7612213

[24] Jumper J, Evans R, Pritzel A, Green T, Figurnov M, Ronneberger O, et al. Highly accurate protein structure prediction with AlphaFold. Nature. 2021;596(7873):583–9. doi: 10.1038/s41586-021-03819-2 34265844 PMC8371605

[25] Evans R, O’Neill M, Pritzel A, Antropova N, Senior A, Green T. Protein complex prediction with AlphaFold-Multimer. bioRxiv. 2021. doi: 10.1101/2021.10.04.463034

[26] Marzella DF, Parizi FM, van Tilborg D, Renaud N, Sybrandi D, Buzatu R, et al. PANDORA: A fast, anchor-restrained modelling protocol for peptide: MHC complexes. Front Immunol. 2022;13:878762. doi: 10.3389/fimmu.2022.878762 35619705 PMC9127323

[27] Zhou P, Shi X, Xia J, Hu H. In silico design and in vitro validation of a multi-epitope peptide vaccine targeting triple-negative breast cancer. Front Oncol. 2025;15:1611991. doi: 10.3389/fonc.2025.1611991 40626008 PMC12229876

[28] Ghosh P, Patra P, Mondal N, Chini DS, Patra BC. Multi epitopic peptide based vaccine development targeting immobilization antigen of ichthyophthirius multifiliis: A computational approach. Int J Pept Res Ther. 2023;29(1):11. doi: 10.1007/s10989-022-10475-1 36532362 PMC9734321

[29] Elshafei SO, Mahmoud NA, Almofti YA. Immunoinformatics, molecular docking and dynamics simulation approaches unveil a multi epitope-based potent peptide vaccine candidate against avian leukosis virus. Sci Rep. 2024;14(1):2870. doi: 10.1038/s41598-024-53048-6 38311642 PMC10838928

[30] Huang QJ, Kim R, Song K, Grigorieff N, Munro JB, Schiffer CA, et al. Virion-associated influenza hemagglutinin clusters upon sialic acid binding visualized by cryo-electron tomography. bioRxiv. 2024:2024.10.15.618557. doi: 10.1101/2024.10.15.618557 40244672 PMC12037027

[31] Xie J, Bi Y, Zhang H, Dong S, Teng L, Lee RJ, et al. Cell-penetrating peptides in diagnosis and treatment of human diseases: From preclinical research to clinical application. Front Pharmacol. 2020;11:697. doi: 10.3389/fphar.2020.00697 32508641 PMC7251059

[32] Waqas M, Haider A, Rehman A, Qasim M, Umar A, Sufyan M, et al. Immunoinformatics and molecular docking studies predicted potential multiepitope-based peptide vaccine and novel compounds against novel SARS-CoV-2 through virtual screening. Biomed Res Int. 2021;2021:1596834. doi: 10.1155/2021/1596834 33728324 PMC7910514

[33] Yan Y, Tao H, He J, Huang S-Y. The HDOCK server for integrated protein-protein docking. Nat Protoc. 2020;15(5):1829–52. doi: 10.1038/s41596-020-0312-x 32269383

[34] Xue LC, Rodrigues JP, Kastritis PL, Bonvin AM, Vangone A. PRODIGY: a web server for predicting the binding affinity of protein-protein complexes. Bioinformatics. 2016;32(23):3676–8. doi: 10.1093/bioinformatics/btw514 27503228

[35] Jorgensen WL, Chandrasekhar J, Madura JD, Impey RW, Klein ML. Comparison of simple potential functions for simulating liquid water. The Journal of Chemical Physics. 1983;79(2):926–35. doi: 10.1063/1.445869

[36] Abraham MJ, Murtola T, Schulz R, Páll S, Smith JC, Hess B, et al. GROMACS: High performance molecular simulations through multi-level parallelism from laptops to supercomputers. SoftwareX. 2015;1–2:19–25. doi: 10.1016/j.softx.2015.06.001

[37] Lemkul JA. Introductory tutorials for simulating protein dynamics with GROMACS. J Phys Chem B. 2024;128(39):9418–35. doi: 10.1021/acs.jpcb.4c04901 39305267 PMC11457149

[38] Sajadi F, Rowley CN. Simulations of lipid bilayers using the CHARMM36 force field with the TIP3P-FB and TIP4P-FB water models. PeerJ. 2018;6:e5472. doi: 10.7717/peerj.5472 30128211 PMC6097494

[39] Su Y, Doherty T, Waring AJ, Ruchala P, Hong M. Roles of arginine and lysine residues in the translocation of a cell-penetrating peptide from (13)C, (31)P, and (19)F solid-state NMR. Biochemistry. 2009;48(21):4587–95. doi: 10.1021/bi900080d 19364134 PMC2723743

[40] Jiang S, Zu C, Wang B, Zhong Y. Enhancing DNA vaccine delivery through stearyl-modified cell-penetrating peptides: Improved antigen expression and immune response in vitro and in vivo. Vaccines (Basel). 2025;13(1):94. doi: 10.3390/vaccines13010094 39852873 PMC11768954

[41] Grau M, Walker PR, Derouazi M. Mechanistic insights into the efficacy of cell penetrating peptide-based cancer vaccines. Cell Mol Life Sci. 2018;75(16):2887–96. doi: 10.1007/s00018-018-2785-0 29508006 PMC6061156

[42] Yang J, Luo Y, Shibu MA, Toth I, Skwarczynskia M. Cell-penetrating peptides: efficient vectors for vaccine delivery. Curr Drug Deliv. 2019;16(5):430–43. doi: 10.2174/1567201816666190123120915 30760185 PMC6637094

[43] Miliotou AN, Georgiou-Siafis SK, Ntenti C, Pappas IS, Papadopoulou LC. Recruiting In vitro transcribed mrna against cancer immunotherapy: A contemporary appraisal of the current landscape. Curr Issues Mol Biol. 2023;45(11):9181–214. doi: 10.3390/cimb45110576 37998753 PMC10670245

[44] Liang H, Xing Y, Wang K, Zhang Y, Yin F, Li Z. Peptides: potential delivery systems for mRNA. RSC Chem Biol. 2025;6(5):666–77. doi: 10.1039/d4cb00295d 40071030 PMC11891934

[45] Georgiou-Siafis SK, Miliotou AN, Ntenti C, Pappas IS, Papadopoulou LC. An innovative PTD-IVT-mRNA delivery platform for CAR immunotherapy of ErbB(+) solid tumor neoplastic cells. Biomedicines. 2022;10(11):2885. doi: 10.3390/biomedicines10112885 36359405 PMC9687928

[46] Miliotou AN, Pappas IS, Spyroulias G, Vlachaki E, Tsiftsoglou AS, Vizirianakis IS, et al. Development of a novel PTD-mediated IVT-mRNA delivery platform for potential protein replacement therapy of metabolic/genetic disorders. Mol Ther Nucleic Acids. 2021;26:694–710. doi: 10.1016/j.omtn.2021.09.008 34703653 PMC8517095

[47] Porosk L, Langel Ü. Approaches for evaluation of novel CPP-based cargo delivery systems. Front Pharmacol. 2022;13:1056467. doi: 10.3389/fphar.2022.1056467 36339538 PMC9634181

[48] Agarwal G, Gabrani R. Antiviral peptides: Identification and validation. Int J Pept Res Ther. 2021;27(1):149–68. doi: 10.1007/s10989-020-10072-0 32427225 PMC7233194

[49] Hemmati S, Behzadipour Y, Haddad M. Decoding the proteome of severe acute respiratory syndrome coronavirus 2 (SARS-CoV-2) for cell-penetrating peptides involved in pathogenesis or applicable as drug delivery vectors. Infect Genet Evol. 2020;85:104474. doi: 10.1016/j.meegid.2020.104474 32712315 PMC7378008

[50] Catalina-Hernandez E, Aguilella-Arzo M, Peralvarez-Marin A, Lopez-Martin M. Computational insights into membrane disruption by cell-penetrating peptides. J Chem Inf Model. 2025;65(3):1549–59. doi: 10.1021/acs.jcim.4c01940 39823544 PMC11815844

[51] Ramasundaram M, Sohn H, Madhavan T. A bird’s-eye view of the biological mechanism and machine learning prediction approaches for cell-penetrating peptides. Front Artif Intell. 2025;7:1497307. doi: 10.3389/frai.2024.1497307 39839972 PMC11747587

[52] Park H, Park J-H, Kim MS, Cho K, Shin J-M. In Silico screening and optimization of cell-penetrating peptides using deep learning methods. Biomolecules. 2023;13(3):522. doi: 10.3390/biom13030522 36979457 PMC10046020

[53] Wei H-H, Yang W, Tang H, Lin H. The development of machine learning methods in cell-penetrating peptides identification: A brief review. Curr Drug Metab. 2019;20(3):217–23. doi: 10.2174/1389200219666181010114750 30317992

[54] Imre A, Balogh B, Mándity I. GraphCPP: The new state-of-the-art method for cell-penetrating peptide prediction via graph neural networks. Br J Pharmacol. 2025;182(3):495–509. doi: 10.1111/bph.17388 39568115

[55] Takechi-Haraya Y, Ohgita T, Kotani M, Kono H, Saito C, Tamagaki-Asahina H, et al. Effect of hydrophobic moment on membrane interaction and cell penetration of apolipoprotein E-derived arginine-rich amphipathic α-helical peptides. Sci Rep. 2022;12(1):4959. doi: 10.1038/s41598-022-08876-9 35322082 PMC8943082

[56] Kajiwara N, Nomura N, Ukaji M, Yamamoto N, Kohara M, Yasui F, et al. Cell-penetrating peptide-mediated cell entry of H5N1 highly pathogenic avian influenza virus. Sci Rep. 2020;10(1):18008. doi: 10.1038/s41598-020-74604-w 33093460 PMC7582914

[57] Allolio C, Magarkar A, Jurkiewicz P, Baxová K, Javanainen M, Mason PE, et al. Arginine-rich cell-penetrating peptides induce membrane multilamellarity and subsequently enter via formation of a fusion pore. Proc Natl Acad Sci U S A. 2018;115(47):11923–8. doi: 10.1073/pnas.1811520115 30397112 PMC6255155

[58] Yamashita H, Oba M, Misawa T, Tanaka M, Hattori T, Naito M, et al. A helix-stabilized cell-penetrating peptide as an intracellular delivery tool. Chembiochem. 2016;17(2):137–40. doi: 10.1002/cbic.201500468 26560998

[59] Lopuszynski J, Wang J, Zahid M. Beyond transduction: Anti-inflammatory effects of cell penetrating peptides. Molecules. 2024;29(17):4088. doi: 10.3390/molecules29174088 39274936 PMC11397606

[60] Walrant A, Tazi F, Khemaissa S, Sagan S. Molecular aspects of cell-penetrating peptides: key amino acids, membrane partners, and non-covalent interactions. Comptes Rendus Chimie. 2025;28(G1):37–51. doi: 10.5802/crchim.359

[61] Agrawal P, Bhalla S, Usmani SS, Singh S, Chaudhary K, Raghava GPS, et al. CPPsite 2.0: a repository of experimentally validated cell-penetrating peptides. Nucleic Acids Res. 2016;44(D1):D1098-103. doi: 10.1093/nar/gkv1266 26586798 PMC4702894

[62] Usmani SS, Bedi G, Samuel JS, Singh S, Kalra S, Kumar P, et al. THPdb: Database of FDA-approved peptide and protein therapeutics. PLoS One. 2017;12(7):e0181748. doi: 10.1371/journal.pone.0181748 28759605 PMC5536290

[63] Timotievich ED, Shilovskiy IP, Khaitov MR. Cell-penetrating peptides as vehicles for delivery of therapeutic nucleic acids. Mechanisms and application in medicine. Biochemistry (Mosc). 2023;88(11):1800–17. doi: 10.1134/S0006297923110111 38105200

[64] Moreno-Vargas LM, Prada-Gracia D. Exploring the chemical features and biomedical relevance of cell-penetrating peptides. Int J Mol Sci. 2024;26(1):59. doi: 10.3390/ijms26010059 39795918 PMC11720145

[65] Deshayes S, Morris MC, Divita G, Heitz F. Cell-penetrating peptides: tools for intracellular delivery of therapeutics. Cell Mol Life Sci. 2005;62(16):1839–49. doi: 10.1007/s00018-005-5109-0 15968462 PMC11139131

[66] Kadkhodayan S, Bolhassani A, Sadat SM, Irani S, Fotouhi F. The efficiency of tat cell penetrating peptide for intracellular uptake of HIV-1 Nef expressed in E. coli and Mammalian Cell. Curr Drug Deliv. 2017;14(4):536–42. doi: 10.2174/1567201813666161006114448 27719633

[67] Ruseska I, Zimmer A. Internalization mechanisms of cell-penetrating peptides. Beilstein J Nanotechnol. 2020;11:101–23. doi: 10.3762/bjnano.11.10 31976201 PMC6964662

[68] Tarvirdipour S, Skowicki M, Schoenenberger C-A, Palivan CG. Peptide-assisted nucleic acid delivery systems on the rise. Int J Mol Sci. 2021;22(16):9092. doi: 10.3390/ijms22169092 34445799 PMC8396486

[69] Namazi F, Bolhassani A, Sadat SM, Irani S. Delivery of HIV-1 polyepitope constructs using cationic and amphipathic cell penetrating peptides into mammalian cells. Curr HIV Res. 2019;17(6):408–28. doi: 10.2174/1570162X17666191121114522 31755394

[70] Wang T, Chen S, Wang Y, Zhang Y, Song X, Bi Z. From in silico to in vitro: A comprehensive guide to validating bioinformatics findings. 2025.

