# Supplementary material for: Designing influenza virus-derived cell-penetrating peptides for antigen delivery: Integrating uptake efficiency, safety, and receptor targeting
Source: PLoS One. 2025 Dec 9;20(12):e0338028. doi: 10.1371/journal.pone.0338028 (PMC12688144; doi:10.1371/journal.pone.0338028)
Supplement: S1 File — (DOCX) [file pone.0338028.s001.docx]

**Table S1. Prediction of cell-penetrating peptides (CPPs) using CellPPD, C2Pred and PreTP-EL web servers.**

| **Epitope** | **CellPPD (SVM score)** | **CPP probability score by C2Pred** | **Predicted Probability**  **PreTP-EL** | **Predicted Class**  **PreTP-EL** |
| --- | --- | --- | --- | --- |
| **NP** |  |  |  |  |
| RSRYWAIRTR | 0.32 | 0.592917 | 0.1711 | CPP |
| RMIKRGINDR | 0.24 | 0.286003 | 0.4069 | CPP |
| GPIYRRVNGK | 0.22 | 0.387432 | 0.3316 | CPP |
| KGTKVVPRGK | 0.20 | 0.177829 | 0.3005 | CPP |
| RRIWRQANNG | 0.19 | 0.967999 | 0.176 | CPP |
| YQRTRALVRT | 0.19 | 0.309629 | 0.2084 | CPP |
| RYWAIRTRSG | 0.19 | 0.489918 | 0.2091 | CPP |
| YRRVNGKWMR | 0.17 | 0.421069 | 0.3462 | CPP |
| GRKTRIAYER | 0.15 | 0.905802 | 0.2224 | CPP |
| RTRALVRTGM | 0.14 | 0.308361 | 0.2134 | CPP |
| SRYWAIRTRS | 0.13 | 0.592917 | 0.1881 | CPP |
| HPSAGKDPKK | 0.12 | 0.351198 | 0.2499 | CPP |
| KEEIRRIWRQ | 0.12 | 0.940236 | 0.2866 | CPP |
| EIRRIWRQAN | 0.11 | 0.958813 | 0.2282 | CPP |
| RGENGRKTRI | 0.09 | 0.816015 | 0.2009 | CPP |
| EEIRRIWRQA | 0.08 | 0.940236 | 0.2306 | CPP |
| FLARSALILR | 0.08 | 0.404196 | 0.1186 | CPP |
| ESSTLELRSR | 0.08 | 0.933678 | 0.1327 | CPP |
| IRRIWRQANN | 0.06 | 0.958813 | 0.1611 | CPP |
| RNFWRGENGR | 0.04 | 0.858907 | 0.3269 | CPP |
| RKTRIAYERM | 0.04 | 0.771652 | 0.3273 | CPP |
| LSAFDERRNK | 0.03 | 0.221823 | 0.3605 | CPP |
| GKWMRELILY | 0.03 | 0.203961 | 0.5762 | non-Cpp |
| TYQRTRALVR | 0.03 | 0.349810 | 0.1911 | CPP |
| RGINDRNFWR | 0.03 | 0.268042 | 0.4678 | CPP |
| WRGENGRKTR | 0.03 | 0.866365 | 0.2241 | CPP |
| PSAGKDPKKT | 0.02 | 0.454553 | 0.2015 | CPP |
| **HA** |  |  |  |  |
| KNSYVNKKGK | 0.22 | 0.054113 | 0.4436 | CPP |
| [KLCRLKGIAP](https://webs.iiitd.edu.in/raghava/cellppd/pepsearch1.php?seq=KLCRLKGIAP&thval=0.0) | 0.17 | 0.208446 | 0.1433 | CPP |
| [PEIAERPKVR](https://webs.iiitd.edu.in/raghava/cellppd/pepsearch1.php?seq=PEIAERPKVR&thval=0.0) | 0.11 | 0.666021 | 0.2545 | CPP |
| [KLKNSYVNKK](https://webs.iiitd.edu.in/raghava/cellppd/pepsearch1.php?seq=KLKNSYVNKK&thval=0.0) | 0.10 | 0.076390 | 0.405 | CPP |
| **Epitope** | **CellPPD (SVM score)** | **CPP probability score by C2Pred** | **Predicted Probability**  **PreTP-EL** | **Predicted Class**  **PreTP-EL** |
| [PKYVRSAKLR](https://webs.iiitd.edu.in/raghava/cellppd/pepsearch1.php?seq=PKYVRSAKLR&thval=0.0) | 0.10 | 0.679905 | 0.1288 | CPP |
| [KYVRSAKLRM](https://webs.iiitd.edu.in/raghava/cellppd/pepsearch1.php?seq=KYVRSAKLRM&thval=0.0) | 0.10 | 0.500000 | 0.2505 | CPP |
| [MNYYWTLLKP](https://webs.iiitd.edu.in/raghava/cellppd/pepsearch1.php?seq=MNYYWTLLKP&thval=0.0) | 0.07 | 0.884811 | 0.547 | non-CPP |
| [HNGKLCRLKG](https://webs.iiitd.edu.in/raghava/cellppd/pepsearch1.php?seq=HNGKLCRLKG&thval=0.0) | 0.04 | 0.287996 | 0.1931 | CPP |
| [GKLCRLKGIA](https://webs.iiitd.edu.in/raghava/cellppd/pepsearch1.php?seq=GKLCRLKGIA&thval=0.0) | 0.04 | 0.208446 | 0.1348 | CPP |
| [GKEFNKLEKR](https://webs.iiitd.edu.in/raghava/cellppd/pepsearch1.php?seq=GKEFNKLEKR&thval=0.0) | 0.02 | 0.325547 | 0.4094 | CPP |
| [RRFTPEIAER](https://webs.iiitd.edu.in/raghava/cellppd/pepsearch1.php?seq=RRFTPEIAER&thval=0.0) | 0.00 | 0.933289 | 0.2375 | CPP |
| **NS1** |  |  |  |  |
| [RLRRDQKSLR](https://webs.iiitd.edu.in/raghava/cellppd/pepsearch1.php?seq=RLRRDQKSLR&thval=0.0) | 0.57 | 0.976407 | 0.1208 | CPP |
| [RRDQKSLRGR](https://webs.iiitd.edu.in/raghava/cellppd/pepsearch1.php?seq=RRDQKSLRGR&thval=0.0) | 0.44 | 0.954928 | 0.1253 | CPP |
| [FLDRLRRDQK](https://webs.iiitd.edu.in/raghava/cellppd/pepsearch1.php?seq=FLDRLRRDQK&thval=0.0) | 0.32 | 0.818091 | 0.2007 | CPP |
| [RPPLTPKQKR](https://webs.iiitd.edu.in/raghava/cellppd/pepsearch1.php?seq=RPPLTPKQKR&thval=0.0) | 0.29 | 0.954020 | 0.1023 | CPP |
| [DAPFLDRLRR](https://webs.iiitd.edu.in/raghava/cellppd/pepsearch1.php?seq=DAPFLDRLRR&thval=0.0) | 0.27 | 0.818091 | 0.1753 | CPP |
| [DRLRRDQKSL](https://webs.iiitd.edu.in/raghava/cellppd/pepsearch1.php?seq=DRLRRDQKSL&thval=0.0) | 0.13 | 0.982321 | 0.1591 | CPP |
| [FLWHVRKRVA](https://webs.iiitd.edu.in/raghava/cellppd/pepsearch1.php?seq=FLWHVRKRVA&thval=0.0) | 0.12 | 0.459609 | 0.2418 | CPP |
| [DCFLWHVRKR](https://webs.iiitd.edu.in/raghava/cellppd/pepsearch1.php?seq=DCFLWHVRKR&thval=0.0) | 0.11 | 0.818397 | 0.4496 | CPP |
| [APFLDRLRRD](https://webs.iiitd.edu.in/raghava/cellppd/pepsearch1.php?seq=APFLDRLRRD&thval=0.0) | 0.05 | 0.818091 | 0.1719 | CPP |
| [EWSMLIPKQK](https://webs.iiitd.edu.in/raghava/cellppd/pepsearch1.php?seq=EWSMLIPKQK&thval=0.0) | 0.05 | 0.464553 | 0.505 | non-CPP |
| [LQRFAWRSSN](https://webs.iiitd.edu.in/raghava/cellppd/pepsearch1.php?seq=LQRFAWRSSN&thval=0.0) | 0.03 | 0.439168 | 0.269 | CPP |
| [GKQIVERILK](https://webs.iiitd.edu.in/raghava/cellppd/pepsearch1.php?seq=GKQIVERILK&thval=0.0) | 0.01 | 0.197411 | 0.3519 | CPP |
| [CFLWHVRKRV](https://webs.iiitd.edu.in/raghava/cellppd/pepsearch1.php?seq=CFLWHVRKRV&thval=0.0) | 0.00 | 0.818397 | 0.2748 | CPP |
| **PB2** |  |  |  |  |
| [RRATAILRKA](https://webs.iiitd.edu.in/raghava/cellppd/pepsearch1.php?seq=RRATAILRKA&thval=0.0) | 0.41 | 0.797095 | 0.0685 | CPP |
| [GRRATAILRK](https://webs.iiitd.edu.in/raghava/cellppd/pepsearch1.php?seq=GRRATAILRK&thval=0.0) | 0.39 | 0.698650 | 0.0803 | CPP |
| [RKATRRLIQL](https://webs.iiitd.edu.in/raghava/cellppd/pepsearch1.php?seq=RKATRRLIQL&thval=0.0) | 0.39 | 0.974975 | 0.0812 | CPP |
| [TAILRKATRR](https://webs.iiitd.edu.in/raghava/cellppd/pepsearch1.php?seq=TAILRKATRR&thval=0.0) | 0.31 | 0.868546 | 0.0629 | CPP |
| [KNPALRMKWM](https://webs.iiitd.edu.in/raghava/cellppd/pepsearch1.php?seq=KNPALRMKWM&thval=0.0) | 0.28 | 0.691326 | 0.4616 | CPP |
| [RATAILRKAT](https://webs.iiitd.edu.in/raghava/cellppd/pepsearch1.php?seq=RATAILRKAT&thval=0.0) | 0.26 | 0.700620 | 0.075 | CPP |
| [ILRKATRRLI](https://webs.iiitd.edu.in/raghava/cellppd/pepsearch1.php?seq=ILRKATRRLI&thval=0.0) | 0.25 | 0.987924 | 0.0576 | CPP |
| [AILRKATRRL](https://webs.iiitd.edu.in/raghava/cellppd/pepsearch1.php?seq=AILRKATRRL&thval=0.0) | 0.23 | 0.872089 | 0.0507 | CPP |
| [SQTATKRIRM](https://webs.iiitd.edu.in/raghava/cellppd/pepsearch1.php?seq=SQTATKRIRM&thval=0.0) | 0.21 | 0.828479 | 0.4127 | CPP |
| [ATAILRKATR](https://webs.iiitd.edu.in/raghava/cellppd/pepsearch1.php?seq=ATAILRKATR&thval=0.0) | 0.17 | 0.816872 | 0.0735 | CPP |
| [KATRRLIQLI](https://webs.iiitd.edu.in/raghava/cellppd/pepsearch1.php?seq=KATRRLIQLI&thval=0.0) | 0.15 | 0.974569 | 0.1134 | CPP |
| [RANQRLNPMH](https://webs.iiitd.edu.in/raghava/cellppd/pepsearch1.php?seq=RANQRLNPMH&thval=0.0) | 0.15 | 0.685965 | 0.3738 | CPP |
| [QSRTREILTK](https://webs.iiitd.edu.in/raghava/cellppd/pepsearch1.php?seq=QSRTREILTK&thval=0.0) | 0.13 | 0.879518 | 0.3004 | CPP |

| **Epitope** | **CellPPD (SVM score)** | **CPP probability score by C2Pred** | **Predicted Probability**  **PreTP-EL** | **Predicted Class**  **PreTP-EL** |
| --- | --- | --- | --- | --- |
| [DRFLRVRDQR](https://webs.iiitd.edu.in/raghava/cellppd/pepsearch1.php?seq=DRFLRVRDQR&thval=0.0) | 0.13 | 0.467146 | 0.2605 | CPP |
| [LRKATRRLIQ](https://webs.iiitd.edu.in/raghava/cellppd/pepsearch1.php?seq=LRKATRRLIQ&thval=0.0) | 0.12 | 0.987924 | 0.0652 | CPP |
| [RLIQLIVSGR](https://webs.iiitd.edu.in/raghava/cellppd/pepsearch1.php?seq=RLIQLIVSGR&thval=0.0) | 0.12 | 0.310935 | 0.2097 | CPP |
| [AIIKKYTSGR](https://webs.iiitd.edu.in/raghava/cellppd/pepsearch1.php?seq=AIIKKYTSGR&thval=0.0) | 0.10 | 0.314587 | 0.3327 | CPP |
| [VGRRATAILR](https://webs.iiitd.edu.in/raghava/cellppd/pepsearch1.php?seq=VGRRATAILR&thval=0.0) | 0.10 | 0.248507 | 0.1199 | CPP |
| [VNRANQRLNP](https://webs.iiitd.edu.in/raghava/cellppd/pepsearch1.php?seq=VNRANQRLNP&thval=0.0) | 0.10 | 0.355447 | 0.3758 | CPP |
| [KKYTSGRQEK](https://webs.iiitd.edu.in/raghava/cellppd/pepsearch1.php?seq=KKYTSGRQEK&thval=0.0) | 0.09 | 0.364530 | 0.4379 | CPP |
| [SQLTITKEKK](https://webs.iiitd.edu.in/raghava/cellppd/pepsearch1.php?seq=SQLTITKEKK&thval=0.0) | 0.09 | 0.975792 | 0.2943 | CPP |
| [VRKTRFLPVA](https://webs.iiitd.edu.in/raghava/cellppd/pepsearch1.php?seq=VRKTRFLPVA&thval=0.0) | 0.09 | 0.428707 | 0.1777 | CPP |
| [DSQTATKRIR](https://webs.iiitd.edu.in/raghava/cellppd/pepsearch1.php?seq=DSQTATKRIR&thval=0.0) | 0.09 | 0.629674 | 0.4363 | CPP |
| [LIIAARNIVR](https://webs.iiitd.edu.in/raghava/cellppd/pepsearch1.php?seq=LIIAARNIVR&thval=0.0) | 0.08 | 0.184951 | 0.1872 | CPP |
| [FAAAPPKQSR](https://webs.iiitd.edu.in/raghava/cellppd/pepsearch1.php?seq=FAAAPPKQSR&thval=0.0) | 0.08 | 0.756423 | 0.1709 | CPP |
| [KRITEMIPER](https://webs.iiitd.edu.in/raghava/cellppd/pepsearch1.php?seq=KRITEMIPER&thval=0.0) | 0.07 | 0.639023 | 0.4231 | CPP |
| [KRTSGSSVKR](https://webs.iiitd.edu.in/raghava/cellppd/pepsearch1.php?seq=KRTSGSSVKR&thval=0.0) | 0.07 | 0.583304 | 0.1486 | CPP |
| [VSIDRFLRVR](https://webs.iiitd.edu.in/raghava/cellppd/pepsearch1.php?seq=VSIDRFLRVR&thval=0.0) | 0.07 | 0.448961 | 0.3137 | CPP |
| [QIIKLLPFAA](https://webs.iiitd.edu.in/raghava/cellppd/pepsearch1.php?seq=QIIKLLPFAA&thval=0.0) | 0.07 | 0.841673 | 0.1819 | CPP |
| [TATKRIRMAI](https://webs.iiitd.edu.in/raghava/cellppd/pepsearch1.php?seq=TATKRIRMAI&thval=0.0) | 0.07 | 0.557242 | 0.2524 | CPP |
| [MSMRGVRISK](https://webs.iiitd.edu.in/raghava/cellppd/pepsearch1.php?seq=MSMRGVRISK&thval=0.0) | 0.06 | 0.172056 | 0.4219 | CPP |
| [NKATKRLTVL](https://webs.iiitd.edu.in/raghava/cellppd/pepsearch1.php?seq=NKATKRLTVL&thval=0.0) | 0.06 | 0.979811 | 0.1845 | CPP |
| [RNLMSQSRTR](https://webs.iiitd.edu.in/raghava/cellppd/pepsearch1.php?seq=RNLMSQSRTR&thval=0.0) | 0.05 | 0.530996 | 0.2797 | CPP |
| [AQIIKLLPFA](https://webs.iiitd.edu.in/raghava/cellppd/pepsearch1.php?seq=AQIIKLLPFA&thval=0.0) | 0.04 | 0.821141 | 0.1987 | CPP |
| [MHQLLRHFQK](https://webs.iiitd.edu.in/raghava/cellppd/pepsearch1.php?seq=MHQLLRHFQK&thval=0.0) | 0.03 | 0.924359 | 0.3958 | CPP |
| [KATKRLTVLG](https://webs.iiitd.edu.in/raghava/cellppd/pepsearch1.php?seq=KATKRLTVLG&thval=0.0) | 0.03 | 0.949815 | 0.1336 | CPP |
| [MKRKRDSSIL](https://webs.iiitd.edu.in/raghava/cellppd/pepsearch1.php?seq=MKRKRDSSIL&thval=0.0) | 0.03 | 0.815099 | 0.2017 | CPP |
| [LVRKTRFLPV](https://webs.iiitd.edu.in/raghava/cellppd/pepsearch1.php?seq=LVRKTRFLPV&thval=0.0) | 0.02 | 0.589446 | 0.1541 | CPP |
| [RKTRFLPVAG](https://webs.iiitd.edu.in/raghava/cellppd/pepsearch1.php?seq=RKTRFLPVAG&thval=0.0) | 0.02 | 0.339920 | 0.1847 | CPP |
| [RNIVRRAAVS](https://webs.iiitd.edu.in/raghava/cellppd/pepsearch1.php?seq=RNIVRRAAVS&thval=0.0) | 0.02 | 0.178243 | 0.1568 | CPP |
| [VLVMKRKRDS](https://webs.iiitd.edu.in/raghava/cellppd/pepsearch1.php?seq=VLVMKRKRDS&thval=0.0) | 0.02 | 0.580435 | 0.2462 | CPP |
| [LLRHFQKDAK](https://webs.iiitd.edu.in/raghava/cellppd/pepsearch1.php?seq=LLRHFQKDAK&thval=0.0) | 0.01 | 0.940346 | 0.3167 | CPP |
| [LLPFAAAPPK](https://webs.iiitd.edu.in/raghava/cellppd/pepsearch1.php?seq=LLPFAAAPPK&thval=0.0) | 0.01 | 0.803844 | 0.1159 | CPP |
| [MERIKELRNL](https://webs.iiitd.edu.in/raghava/cellppd/pepsearch1.php?seq=MERIKELRNL&thval=0.0) | 0.00 | 0.912982 | 0.4233 | CPP |
| [RRRVDINPGH](https://webs.iiitd.edu.in/raghava/cellppd/pepsearch1.php?seq=RRRVDINPGH&thval=0.0) | 0.00 | 0.276320 | 0.2994 | CPP |
| [ATKRLTVLGK](https://webs.iiitd.edu.in/raghava/cellppd/pepsearch1.php?seq=ATKRLTVLGK&thval=0.0) | 0.00 | 0.955670 | 0.1256 | CPP |
| **M2** |  |  |  |  |
| [KCIYRRFKYG](https://webs.iiitd.edu.in/raghava/cellppd/pepsearch1.php?seq=KCIYRRFKYG&thval=0.0) | 0.36 | 0.635126 | 0.3661 | CPP |
| [RLFFKCIYRR](https://webs.iiitd.edu.in/raghava/cellppd/pepsearch1.php?seq=RLFFKCIYRR&thval=0.0) | 0.19 | 0.531900 | 0.23 | CPP |
| **Epitope** | **CellPPD (SVM score)** | **CPP probability score by C2Pred** | **Predicted Probability**  **PreTP-EL** | **Predicted Class**  **PreTP-EL** |
| [IYRRFKYGLK](https://webs.iiitd.edu.in/raghava/cellppd/pepsearch1.php?seq=IYRRFKYGLK&thval=0.0) | 0.14 | 0.810444 | 0.2159 | CPP |
| [YRRFKYGLKG](https://webs.iiitd.edu.in/raghava/cellppd/pepsearch1.php?seq=YRRFKYGLKG&thval=0.0) | 0.11 | 0.500000 | 0.2062 | CPP |
| [FFKCIYRRFK](https://webs.iiitd.edu.in/raghava/cellppd/pepsearch1.php?seq=FFKCIYRRFK&thval=0.0) | 0.10 | 0.635126 | 0.3504 | CPP |
| [FKCIYRRFKY](https://webs.iiitd.edu.in/raghava/cellppd/pepsearch1.php?seq=FKCIYRRFKY&thval=0.0) | 0.10 | 0.635126 | 0.3847 | CPP |
| [CIYRRFKYGL](https://webs.iiitd.edu.in/raghava/cellppd/pepsearch1.php?seq=CIYRRFKYGL&thval=0.0) | 0.10 | 0.428028 | 0.4123 | CPP |
| [WILDRLFFKC](https://webs.iiitd.edu.in/raghava/cellppd/pepsearch1.php?seq=WILDRLFFKC&thval=0.0) | 0.07 | 0.782855 | 0.4614 | CPP |
| [LWILDRLFFK](https://webs.iiitd.edu.in/raghava/cellppd/pepsearch1.php?seq=LWILDRLFFK&thval=0.0) | 0.06 | 0.857737 | 0.318 | CPP |
| **NA** |  |  |  |  |
| [IKSWRKKILR](https://webs.iiitd.edu.in/raghava/cellppd/pepsearch1.php?seq=IKSWRKKILR&thval=0.0) | 0.39 | 0.964861 | 0.1168 | CPP |
| [ELIRGRPKEK](https://webs.iiitd.edu.in/raghava/cellppd/pepsearch1.php?seq=ELIRGRPKEK&thval=0.0) | 0.37 | 0.908036 | 0.1577 | CPP |
| [YKIFKIEKGK](https://webs.iiitd.edu.in/raghava/cellppd/pepsearch1.php?seq=YKIFKIEKGK&thval=0.0) | 0.20 | 0.603098 | 0.4612 | CPP |
| [ETIKSWRKKI](https://webs.iiitd.edu.in/raghava/cellppd/pepsearch1.php?seq=ETIKSWRKKI&thval=0.0) | 0.19 | 0.705195 | 0.2651 | CPP |
| [TIKSWRKKIL](https://webs.iiitd.edu.in/raghava/cellppd/pepsearch1.php?seq=TIKSWRKKIL&thval=0.0) | 0.19 | 0.964662 | 0.1922 | CPP |
| [KIFKIEKGKV](https://webs.iiitd.edu.in/raghava/cellppd/pepsearch1.php?seq=KIFKIEKGKV&thval=0.0) | 0.19 | 0.392078 | 0.4474 | CPP |
| [LIRGRPKEKT](https://webs.iiitd.edu.in/raghava/cellppd/pepsearch1.php?seq=LIRGRPKEKT&thval=0.0) | 0.14 | 0.912019 | 0.1523 | CPP |
| [TETIKSWRKK](https://webs.iiitd.edu.in/raghava/cellppd/pepsearch1.php?seq=TETIKSWRKK&thval=0.0) | 0.08 | 0.705195 | 0.2673 | CPP |
| [KIEKGKVTKS](https://webs.iiitd.edu.in/raghava/cellppd/pepsearch1.php?seq=KIEKGKVTKS&thval=0.0) | 0.08 | 0.392078 | 0.4426 | CPP |
| [GVWIGRTKSH](https://webs.iiitd.edu.in/raghava/cellppd/pepsearch1.php?seq=GVWIGRTKSH&thval=0.0) | 0.04 | 0.378033 | 0.5692 | non-CPP |
| [FWVELIRGRP](https://webs.iiitd.edu.in/raghava/cellppd/pepsearch1.php?seq=FWVELIRGRP&thval=0.0) | 0.02 | 0.908036 | 0.3252 | CPP |
| [PIRGWAIYSK](https://webs.iiitd.edu.in/raghava/cellppd/pepsearch1.php?seq=PIRGWAIYSK&thval=0.0) | 0.00 | 0.619087 | 0.4092 | CPP |
| **PB1-F2** |  |  |  |  |
| [KTRVLKRWRL](https://webs.iiitd.edu.in/raghava/cellppd/pepsearch1.php?seq=KTRVLKRWRL&thval=0.0) | 0.54 | 0.972567 | 0.1071 | CPP |
| [LKRWRLFSKH](https://webs.iiitd.edu.in/raghava/cellppd/pepsearch1.php?seq=LKRWRLFSKH&thval=0.0) | 0.40 | 0.905850 | 0.1377 | CPP |
| [VLKRWRLFSK](https://webs.iiitd.edu.in/raghava/cellppd/pepsearch1.php?seq=VLKRWRLFSK&thval=0.0) | 0.38 | 0.905850 | 0.1346 | CPP |
| [LKTRVLKRWR](https://webs.iiitd.edu.in/raghava/cellppd/pepsearch1.php?seq=LKTRVLKRWR&thval=0.0) | 0.37 | 0.940004 | 0.1022 | CPP |
| [FLKTRVLKRW](https://webs.iiitd.edu.in/raghava/cellppd/pepsearch1.php?seq=FLKTRVLKRW&thval=0.0) | 0.22 | 0.839233 | 0.1554 | CPP |
| [RVLKRWRLFS](https://webs.iiitd.edu.in/raghava/cellppd/pepsearch1.php?seq=RVLKRWRLFS&thval=0.0) | 0.22 | 0.905850 | 0.1079 | CPP |
| [KRWRLFSKHE](https://webs.iiitd.edu.in/raghava/cellppd/pepsearch1.php?seq=KRWRLFSKHE&thval=0.0) | 0.19 | 0.936260 | 0.2269 | CPP |
| [WKQWLSLRNP](https://webs.iiitd.edu.in/raghava/cellppd/pepsearch1.php?seq=WKQWLSLRNP&thval=0.0) | 0.17 | 0.786391 | 0.2359 | CPP |
| [TRVLKRWRLF](https://webs.iiitd.edu.in/raghava/cellppd/pepsearch1.php?seq=TRVLKRWRLF&thval=0.0) | 0.16 | 0.892810 | 0.121 | CPP |
| [KQWLSLRNPI](https://webs.iiitd.edu.in/raghava/cellppd/pepsearch1.php?seq=KQWLSLRNPI&thval=0.0) | 0.10 | 0.779118 | 0.2463 | CPP |
| [RNSTRLMGHC](https://webs.iiitd.edu.in/raghava/cellppd/pepsearch1.php?seq=RNSTRLMGHC&thval=0.0) | 0.05 | 0.173810 | 0.5212 | non-CPP |
| [GQQTPKLEHR](https://webs.iiitd.edu.in/raghava/cellppd/pepsearch1.php?seq=GQQTPKLEHR&thval=0.0) | 0.03 | 0.701127 | 0.5139 | non-CPP |
| [KQIVYWKQWL](https://webs.iiitd.edu.in/raghava/cellppd/pepsearch1.php?seq=KQIVYWKQWL&thval=0.0) | 0.03 | 0.201718 | 0.6054 | non-CPP |
| [HRNSTRLMGH](https://webs.iiitd.edu.in/raghava/cellppd/pepsearch1.php?seq=HRNSTRLMGH&thval=0.0) | 0.02 | 0.216480 | 0.4477 | CPP |

| **Epitope** | **CellPPD (SVM score)** | **CPP probability score by C2Pred** | **Predicted Probability**  **PreTP-EL** | **Predicted Class**  **PreTP-EL** |
| --- | --- | --- | --- | --- |
| [VYWKQWLSLR](https://webs.iiitd.edu.in/raghava/cellppd/pepsearch1.php?seq=VYWKQWLSLR&thval=0.0) | 0.00 | 0.396143 | 0.3395 | CPP |
| **PA** |  |  |  |  |
| [KIPKTKNMKK](https://webs.iiitd.edu.in/raghava/cellppd/pepsearch1.php?seq=KIPKTKNMKK&thval=0.0) | 0.61 | 0.677321 | 0.3677 | CPP |
| [RIKTRLFTIR](https://webs.iiitd.edu.in/raghava/cellppd/pepsearch1.php?seq=RIKTRLFTIR&thval=0.0) | 0.43 | 0.852958 | 0.1317 | CPP |
| [KTTPRPLRLP](https://webs.iiitd.edu.in/raghava/cellppd/pepsearch1.php?seq=KTTPRPLRLP&thval=0.0) | 0.39 | 0.962691 | 0.0745 | CPP |
| [ESRARIKTRL](https://webs.iiitd.edu.in/raghava/cellppd/pepsearch1.php?seq=ESRARIKTRL&thval=0.0) | 0.35 | 0.920098 | 0.1166 | CPP |
| [RARIKTRLFT](https://webs.iiitd.edu.in/raghava/cellppd/pepsearch1.php?seq=RARIKTRLFT&thval=0.0) | 0.33 | 0.809643 | 0.1101 | CPP |
| [FLKTTPRPLR](https://webs.iiitd.edu.in/raghava/cellppd/pepsearch1.php?seq=FLKTTPRPLR&thval=0.0) | 0.33 | 0.766167 | 0.0984 | CPP |
| [FIIKGRSHLR](https://webs.iiitd.edu.in/raghava/cellppd/pepsearch1.php?seq=FIIKGRSHLR&thval=0.0) | 0.30 | 0.479150 | 0.1673 | CPP |
| [LKTTPRPLRL](https://webs.iiitd.edu.in/raghava/cellppd/pepsearch1.php?seq=LKTTPRPLRL&thval=0.0) | 0.27 | 0.951361 | 0.0777 | CPP |
| [PRPLRLPNGP](https://webs.iiitd.edu.in/raghava/cellppd/pepsearch1.php?seq=PRPLRLPNGP&thval=0.0) | 0.27 | 0.752817 | 0.1131 | CPP |
| [KNMKKTSQLK](https://webs.iiitd.edu.in/raghava/cellppd/pepsearch1.php?seq=KNMKKTSQLK&thval=0.0) | 0.24 | 0.970219 | 0.3654 | CPP |
| [EKIPKTKNMK](https://webs.iiitd.edu.in/raghava/cellppd/pepsearch1.php?seq=EKIPKTKNMK&thval=0.0) | 0.21 | 0.626595 | 0.5927 | non-CPP |
| [GKVCRTLLAK](https://webs.iiitd.edu.in/raghava/cellppd/pepsearch1.php?seq=GKVCRTLLAK&thval=0.0) | 0.21 | 0.454973 | 0.2472 | CPP |
| [SRARIKTRLF](https://webs.iiitd.edu.in/raghava/cellppd/pepsearch1.php?seq=SRARIKTRLF&thval=0.0) | 0.17 | 0.783507 | 0.1108 | CPP |
| [KKTSQLKWAL](https://webs.iiitd.edu.in/raghava/cellppd/pepsearch1.php?seq=KKTSQLKWAL&thval=0.0) | 0.14 | 0.936287 | 0.1773 | CPP |
| [KGRSHLRNDT](https://webs.iiitd.edu.in/raghava/cellppd/pepsearch1.php?seq=KGRSHLRNDT&thval=0.0) | 0.13 | 0.560870 | 0.295 | CPP |
| [TTPRPLRLPN](https://webs.iiitd.edu.in/raghava/cellppd/pepsearch1.php?seq=TTPRPLRLPN&thval=0.0) | 0.09 | 0.886076 | 0.1028 | CPP |
| [ARIKTRLFTI](https://webs.iiitd.edu.in/raghava/cellppd/pepsearch1.php?seq=ARIKTRLFTI&thval=0.0) | 0.08 | 0.832779 | 0.1573 | CPP |
| [KCMRTFFGWK](https://webs.iiitd.edu.in/raghava/cellppd/pepsearch1.php?seq=KCMRTFFGWK&thval=0.0) | 0.07 | 0.447831 | 0.6968 | non-CPP |
| [EESRARIKTR](https://webs.iiitd.edu.in/raghava/cellppd/pepsearch1.php?seq=EESRARIKTR&thval=0.0) | 0.06 | 0.890664 | 0.1397 | CPP |
| [PFLKTTPRPL](https://webs.iiitd.edu.in/raghava/cellppd/pepsearch1.php?seq=PFLKTTPRPL&thval=0.0) | 0.06 | 0.726349 | 0.114 | CPP |
| [NGTSKIKMKW](https://webs.iiitd.edu.in/raghava/cellppd/pepsearch1.php?seq=NGTSKIKMKW&thval=0.0) | 0.06 | 0.557199 | 0.593 | non-CPP |
| [TPRPLRLPNG](https://webs.iiitd.edu.in/raghava/cellppd/pepsearch1.php?seq=TPRPLRLPNG&thval=0.0) | 0.05 | 0.842005 | 0.1202 | CPP |
| [KTKNMKKTSQ](https://webs.iiitd.edu.in/raghava/cellppd/pepsearch1.php?seq=KTKNMKKTSQ&thval=0.0) | 0.05 | 0.937044 | 0.4513 | CPP |
| [HIYYLEKANK](https://webs.iiitd.edu.in/raghava/cellppd/pepsearch1.php?seq=HIYYLEKANK&thval=0.0) | 0.04 | 0.903898 | 0.669 | non-CPP |
| [KMKWGMEMRR](https://webs.iiitd.edu.in/raghava/cellppd/pepsearch1.php?seq=KMKWGMEMRR&thval=0.0) | 0.04 | 0.491738 | 0.5448 | non-CPP |
| [KVCRTLLAKS](https://webs.iiitd.edu.in/raghava/cellppd/pepsearch1.php?seq=KVCRTLLAKS&thval=0.0) | 0.03 | 0.454973 | 0.2318 | CPP |
| [RFIEIGVTRR](https://webs.iiitd.edu.in/raghava/cellppd/pepsearch1.php?seq=RFIEIGVTRR&thval=0.0) | 0.02 | 0.228127 | 0.3861 | CPP |
| [TKEGRRKTNL](https://webs.iiitd.edu.in/raghava/cellppd/pepsearch1.php?seq=TKEGRRKTNL&thval=0.0) | 0.02 | 0.390533 | 0.2193 | CPP |
| [PLRLPNGPPC](https://webs.iiitd.edu.in/raghava/cellppd/pepsearch1.php?seq=PLRLPNGPPC&thval=0.0) | 0.01 | 0.410268 | 0.2025 | CPP |
| [PKTKNMKKTS](https://webs.iiitd.edu.in/raghava/cellppd/pepsearch1.php?seq=PKTKNMKKTS&thval=0.0) | 0.01 | 0.937044 | 0.3686 | CPP |
| [HCRATEYIMK](https://webs.iiitd.edu.in/raghava/cellppd/pepsearch1.php?seq=HCRATEYIMK&thval=0.0) | 0.01 | 0.870900 | 0.8473 | non-CPP |
| [KEGRRKTNLY](https://webs.iiitd.edu.in/raghava/cellppd/pepsearch1.php?seq=KEGRRKTNLY&thval=0.0) | 0.00 | 0.197137 | 0.259 | CPP |
| **Epitope** | **CellPPD (SVM score)** | **CPP probability score by C2Pred** | **Predicted Probability**  **PreTP-EL** | **Predicted Class**  **PreTP-EL** |
| **M1** |  |  |  |  |
| [KAVKLYRKLK](https://webs.iiitd.edu.in/raghava/cellppd/pepsearch1.php?seq=KAVKLYRKLK&thval=0.0) | 0.70 | 0.351095 | 0.1149 | CPP |
| [RGLQRRRFVQ](https://webs.iiitd.edu.in/raghava/cellppd/pepsearch1.php?seq=RGLQRRRFVQ&thval=0.0) | 0.41 | 0.481758 | 0.1366 | CPP |
| [KLYRKLKREI](https://webs.iiitd.edu.in/raghava/cellppd/pepsearch1.php?seq=KLYRKLKREI&thval=0.0) | 0.40 | 0.616420 | 0.1222 | CPP |
| [AVKLYRKLKR](https://webs.iiitd.edu.in/raghava/cellppd/pepsearch1.php?seq=AVKLYRKLKR&thval=0.0) | 0.28 | 0.482452 | 0.0903 | CPP |
| [LQRRRFVQNA](https://webs.iiitd.edu.in/raghava/cellppd/pepsearch1.php?seq=LQRRRFVQNA&thval=0.0) | 0.17 | 0.601823 | 0.1693 | CPP |
| [RKLKREITFH](https://webs.iiitd.edu.in/raghava/cellppd/pepsearch1.php?seq=RKLKREITFH&thval=0.0) | 0.12 | 0.991469 | 0.2515 | CPP |
| [GLQRRRFVQN](https://webs.iiitd.edu.in/raghava/cellppd/pepsearch1.php?seq=GLQRRRFVQN&thval=0.0) | 0.11 | 0.417491 | 0.2037 | CPP |
| [VKLYRKLKRE](https://webs.iiitd.edu.in/raghava/cellppd/pepsearch1.php?seq=VKLYRKLKRE&thval=0.0) | 0.11 | 0.616420 | 0.1305 | CPP |
| [ERGLQRRRFV](https://webs.iiitd.edu.in/raghava/cellppd/pepsearch1.php?seq=ERGLQRRRFV&thval=0.0) | 0.08 | 0.723717 | 0.1724 | CPP |
| [WLKTRPILSP](https://webs.iiitd.edu.in/raghava/cellppd/pepsearch1.php?seq=WLKTRPILSP&thval=0.0) | 0.07 | 0.812047 | 0.1617 | CPP |
| [DKAVKLYRKL](https://webs.iiitd.edu.in/raghava/cellppd/pepsearch1.php?seq=DKAVKLYRKL&thval=0.0) | 0.02 | 0.322783 | 0.2358 | CPP |
| [MDKAVKLYRK](https://webs.iiitd.edu.in/raghava/cellppd/pepsearch1.php?seq=MDKAVKLYRK&thval=0.0) | 0.01 | 0.282299 | 0.4213 | CPP |
| PA-X |  |  |  |  |
| [RASCLKCPKK](https://webs.iiitd.edu.in/raghava/cellppd/pepsearch1.php?seq=RASCLKCPKK&thval=0.0) | 0.52 | 0.268280 | 0.1335 | CPP |
| [EKRQLKKGLK](https://webs.iiitd.edu.in/raghava/cellppd/pepsearch1.php?seq=EKRQLKKGLK&thval=0.0) | 0.44 | 0.736765 | 0.1083 | CPP |
| [RIKTRLFTIR](https://webs.iiitd.edu.in/raghava/cellppd/pepsearch1.php?seq=RIKTRLFTIR&thval=0.0) | 0.43 | 0.852958 | 0.1317 | CPP |
| [KRQLKKGLKS](https://webs.iiitd.edu.in/raghava/cellppd/pepsearch1.php?seq=KRQLKKGLKS&thval=0.0) | 0.36 | 0.736765 | 0.0638 | CPP |
| [ESRARIKTRL](https://webs.iiitd.edu.in/raghava/cellppd/pepsearch1.php?seq=ESRARIKTRL&thval=0.0) | 0.35 | 0.920098 | 0.1166 | CPP |
| [RARIKTRLFT](https://webs.iiitd.edu.in/raghava/cellppd/pepsearch1.php?seq=RARIKTRLFT&thval=0.0) | 0.33 | 0.809643 | 0.1101 | CPP |
| [RQLKKGLKSQ](https://webs.iiitd.edu.in/raghava/cellppd/pepsearch1.php?seq=RQLKKGLKSQ&thval=0.0) | 0.24 | 0.658294 | 0.0791 | CPP |
| [REEKRQLKKG](https://webs.iiitd.edu.in/raghava/cellppd/pepsearch1.php?seq=REEKRQLKKG&thval=0.0) | 0.20 | 0.841008 | 0.1356 | CPP |
| [SRARIKTRLF](https://webs.iiitd.edu.in/raghava/cellppd/pepsearch1.php?seq=SRARIKTRLF&thval=0.0) | 0.17 | 0.783507 | 0.1108 | CPP |
| [PREEKRQLKK](https://webs.iiitd.edu.in/raghava/cellppd/pepsearch1.php?seq=PREEKRQLKK&thval=0.0) | 0.17 | 0.945533 | 0.148 | CPP |
| [LRASCLKCPK](https://webs.iiitd.edu.in/raghava/cellppd/pepsearch1.php?seq=LRASCLKCPK&thval=0.0) | 0.15 | 0.291811 | 0.1468 | CPP |
| [KVSRRTSPAL](https://webs.iiitd.edu.in/raghava/cellppd/pepsearch1.php?seq=KVSRRTSPAL&thval=0.0) | 0.13 | 0.688270 | 0.1168 | CPP |
| [ARIKTRLFTI](https://webs.iiitd.edu.in/raghava/cellppd/pepsearch1.php?seq=ARIKTRLFTI&thval=0.0) | 0.08 | 0.832779 | 0.1573 | CPP |
| [PTKVSRRTSP](https://webs.iiitd.edu.in/raghava/cellppd/pepsearch1.php?seq=PTKVSRRTSP&thval=0.0) | 0.08 | 0.710399 | 0.113 | CPP |
| [VSRRTSPALK](https://webs.iiitd.edu.in/raghava/cellppd/pepsearch1.php?seq=VSRRTSPALK&thval=0.0) | 0.08 | 0.866018 | 0.1126 | CPP |
| [NRTATLRASC](https://webs.iiitd.edu.in/raghava/cellppd/pepsearch1.php?seq=NRTATLRASC&thval=0.0) | 0.08 | 0.616475 | 0.2134 | CPP |
| [EESRARIKTR](https://webs.iiitd.edu.in/raghava/cellppd/pepsearch1.php?seq=EESRARIKTR&thval=0.0) | 0.06 | 0.890664 | 0.1397 | CPP |
| [SRRTSPALKI](https://webs.iiitd.edu.in/raghava/cellppd/pepsearch1.php?seq=SRRTSPALKI&thval=0.0) | 0.06 | 0.866018 | 0.0955 | CPP |
| [SNRTATLRAS](https://webs.iiitd.edu.in/raghava/cellppd/pepsearch1.php?seq=SNRTATLRAS&thval=0.0) | 0.06 | 0.550042 | 0.1229 | CPP |
| [HIYYLEKANK](https://webs.iiitd.edu.in/raghava/cellppd/pepsearch1.php?seq=HIYYLEKANK&thval=0.0) | 0.04 | 0.903898 | 0.669 | non-CPP |
| [RFIEIGVTRR](https://webs.iiitd.edu.in/raghava/cellppd/pepsearch1.php?seq=RFIEIGVTRR&thval=0.0) | 0.02 | 0.228127 | 0.3861 | CPP |
| [ASLPTKVSRR](https://webs.iiitd.edu.in/raghava/cellppd/pepsearch1.php?seq=ASLPTKVSRR&thval=0.0) | 0.02 | 0.812164 | 0.1335 | CPP |
| **Epitope** | **CellPPD (SVM score)** | **CPP probability score by C2Pred** | **Predicted Probability**  **PreTP-EL** | **Predicted Class**  **PreTP-EL** |
| [TKVSRRTSPA](https://webs.iiitd.edu.in/raghava/cellppd/pepsearch1.php?seq=TKVSRRTSPA&thval=0.0) | 0.02 | 0.710399 | 0.1306 | CPP |
| [RRTSPALKIL](https://webs.iiitd.edu.in/raghava/cellppd/pepsearch1.php?seq=RRTSPALKIL&thval=0.0) | 0.02 | 0.906091 | 0.0918 | CPP |
| [SPREEKRQLK](https://webs.iiitd.edu.in/raghava/cellppd/pepsearch1.php?seq=SPREEKRQLK&thval=0.0) | 0.00 | 0.938925 | 0.1863 | CPP |
| PB1 |  |  |  |  |
| [FQRKRRVRDN](https://webs.iiitd.edu.in/raghava/cellppd/pepsearch1.php?seq=FQRKRRVRDN&thval=0.0) | 0.57 | 0.559212 | 0.2004 | CPP |
| [THFQRKRRVR](https://webs.iiitd.edu.in/raghava/cellppd/pepsearch1.php?seq=THFQRKRRVR&thval=0.0) | 0.53 | 0.417970 | 0.1421 | CPP |
| [KMITQRTIGK](https://webs.iiitd.edu.in/raghava/cellppd/pepsearch1.php?seq=KMITQRTIGK&thval=0.0) | 0.39 | 0.243300 | 0.4467 | CPP |
| [RRVRDNMTKK](https://webs.iiitd.edu.in/raghava/cellppd/pepsearch1.php?seq=RRVRDNMTKK&thval=0.0) | 0.33 | 0.510594 | 0.2946 | CPP |
| [GITTHFQRKR](https://webs.iiitd.edu.in/raghava/cellppd/pepsearch1.php?seq=GITTHFQRKR&thval=0.0) | 0.30 | 0.353497 | 0.2471 | CPP |
| [HFQRKRRVRD](https://webs.iiitd.edu.in/raghava/cellppd/pepsearch1.php?seq=HFQRKRRVRD&thval=0.0) | 0.30 | 0.905057 | 0.1966 | CPP |
| [KKIEKIRPLL](https://webs.iiitd.edu.in/raghava/cellppd/pepsearch1.php?seq=KKIEKIRPLL&thval=0.0) | 0.30 | 0.915916 | 0.1703 | CPP |
| [RLNKRSYLIR](https://webs.iiitd.edu.in/raghava/cellppd/pepsearch1.php?seq=RLNKRSYLIR&thval=0.0) | 0.29 | 0.968922 | 0.1322 | CPP |
| [DAERGKLKRR](https://webs.iiitd.edu.in/raghava/cellppd/pepsearch1.php?seq=DAERGKLKRR&thval=0.0) | 0.29 | 0.979273 | 0.1231 | CPP |
| [ERGKLKRRAI](https://webs.iiitd.edu.in/raghava/cellppd/pepsearch1.php?seq=ERGKLKRRAI&thval=0.0) | 0.28 | 0.782463 | 0.0707 | CPP |
| [KDAERGKLKR](https://webs.iiitd.edu.in/raghava/cellppd/pepsearch1.php?seq=KDAERGKLKR&thval=0.0) | 0.27 | 0.961435 | 0.1627 | CPP |
| [QRKRRVRDNM](https://webs.iiitd.edu.in/raghava/cellppd/pepsearch1.php?seq=QRKRRVRDNM&thval=0.0) | 0.26 | 0.624240 | 0.2458 | CPP |
| [AERGKLKRRA](https://webs.iiitd.edu.in/raghava/cellppd/pepsearch1.php?seq=AERGKLKRRA&thval=0.0) | 0.26 | 0.969680 | 0.0433 | CPP |
| [KRRVRDNMTK](https://webs.iiitd.edu.in/raghava/cellppd/pepsearch1.php?seq=KRRVRDNMTK&thval=0.0) | 0.25 | 0.667438 | 0.3131 | CPP |
| [TQRTIGKRKQ](https://webs.iiitd.edu.in/raghava/cellppd/pepsearch1.php?seq=TQRTIGKRKQ&thval=0.0) | 0.24 | 0.317664 | 0.161 | CPP |
| [RGKLKRRAIA](https://webs.iiitd.edu.in/raghava/cellppd/pepsearch1.php?seq=RGKLKRRAIA&thval=0.0) | 0.23 | 0.782463 | 0.0485 | CPP |
| [SWIPKRNRSI](https://webs.iiitd.edu.in/raghava/cellppd/pepsearch1.php?seq=SWIPKRNRSI&thval=0.0) | 0.22 | 0.571732 | 0.1998 | CPP |
| [MITQRTIGKR](https://webs.iiitd.edu.in/raghava/cellppd/pepsearch1.php?seq=MITQRTIGKR&thval=0.0) | 0.21 | 0.290496 | 0.3602 | CPP |
| [GKLKRRAIAT](https://webs.iiitd.edu.in/raghava/cellppd/pepsearch1.php?seq=GKLKRRAIAT&thval=0.0) | 0.21 | 0.783223 | 0.0685 | CPP |
| [KRRAIATPGM](https://webs.iiitd.edu.in/raghava/cellppd/pepsearch1.php?seq=KRRAIATPGM&thval=0.0) | 0.20 | 0.281324 | 0.2228 | CPP |
| [KKLWEQTRSK](https://webs.iiitd.edu.in/raghava/cellppd/pepsearch1.php?seq=KKLWEQTRSK&thval=0.0) | 0.20 | 0.394164 | 0.3782 | CPP |
| [ITTHFQRKRR](https://webs.iiitd.edu.in/raghava/cellppd/pepsearch1.php?seq=ITTHFQRKRR&thval=0.0) | 0.19 | 0.436991 | 0.1433 | CPP |
| [TTHFQRKRRV](https://webs.iiitd.edu.in/raghava/cellppd/pepsearch1.php?seq=TTHFQRKRRV&thval=0.0) | 0.19 | 0.436991 | 0.1608 | CPP |
| [RKRRVRDNMT](https://webs.iiitd.edu.in/raghava/cellppd/pepsearch1.php?seq=RKRRVRDNMT&thval=0.0) | 0.19 | 0.777305 | 0.2391 | CPP |
| [KSMKLRTQIP](https://webs.iiitd.edu.in/raghava/cellppd/pepsearch1.php?seq=KSMKLRTQIP&thval=0.0) | 0.19 | 0.252381 | 0.3369 | CPP |
| [TRKKIEKIRP](https://webs.iiitd.edu.in/raghava/cellppd/pepsearch1.php?seq=TRKKIEKIRP&thval=0.0) | 0.19 | 0.969438 | 0.1412 | CPP |
| [HSWIPKRNRS](https://webs.iiitd.edu.in/raghava/cellppd/pepsearch1.php?seq=HSWIPKRNRS&thval=0.0) | 0.19 | 0.571732 | 0.1945 | CPP |
| [WIPKRNRSIL](https://webs.iiitd.edu.in/raghava/cellppd/pepsearch1.php?seq=WIPKRNRSIL&thval=0.0) | 0.17 | 0.540618 | 0.1873 | CPP |
| [STRKKIEKIR](https://webs.iiitd.edu.in/raghava/cellppd/pepsearch1.php?seq=STRKKIEKIR&thval=0.0) | 0.16 | 0.969438 | 0.1573 | CPP |
| [MGITTHFQRK](https://webs.iiitd.edu.in/raghava/cellppd/pepsearch1.php?seq=MGITTHFQRK&thval=0.0) | 0.15 | 0.334181 | 0.6431 | non-CPP |
| [KYFNDSTRKK](https://webs.iiitd.edu.in/raghava/cellppd/pepsearch1.php?seq=KYFNDSTRKK&thval=0.0) | 0.14 | 0.272252 | 0.4746 | CPP |
| **Epitope** | **CellPPD (SVM score)** | **CPP probability score by C2Pred** | **Predicted Probability**  **PreTP-EL** | **Predicted Class**  **PreTP-EL** |
| [NDSTRKKIEK](https://webs.iiitd.edu.in/raghava/cellppd/pepsearch1.php?seq=NDSTRKKIEK&thval=0.0) | 0.14 | 0.474747 | 0.395 | CPP |
| [KIEKIRPLLI](https://webs.iiitd.edu.in/raghava/cellppd/pepsearch1.php?seq=KIEKIRPLLI&thval=0.0) | 0.13 | 0.897872 | 0.2452 | CPP |
| [KLWEQTRSKA](https://webs.iiitd.edu.in/raghava/cellppd/pepsearch1.php?seq=KLWEQTRSKA&thval=0.0) | 0.13 | 0.373930 | 0.4061 | CPP |
| [KLKRRAIATP](https://webs.iiitd.edu.in/raghava/cellppd/pepsearch1.php?seq=KLKRRAIATP&thval=0.0) | 0.12 | 0.783223 | 0.0724 | CPP |
| [GINMSKKKSY](https://webs.iiitd.edu.in/raghava/cellppd/pepsearch1.php?seq=GINMSKKKSY&thval=0.0) | 0.12 | 0.200451 | 0.5054 | non-CPP |
| [ITQRTIGKRK](https://webs.iiitd.edu.in/raghava/cellppd/pepsearch1.php?seq=ITQRTIGKRK&thval=0.0) | 0.11 | 0.317664 | 0.1742 | CPP |
| [MSKKKSYINR](https://webs.iiitd.edu.in/raghava/cellppd/pepsearch1.php?seq=MSKKKSYINR&thval=0.0) | 0.11 | 0.197307 | 0.3974 | CPP |
| [RQTYDWTLNR](https://webs.iiitd.edu.in/raghava/cellppd/pepsearch1.php?seq=RQTYDWTLNR&thval=0.0) | 0.10 | 0.895062 | 0.5492 | non-CPP |
| [QTRRSFEIKK](https://webs.iiitd.edu.in/raghava/cellppd/pepsearch1.php?seq=QTRRSFEIKK&thval=0.0) | 0.09 | 0.971726 | 0.3563 | CPP |
| [THSWIPKRNR](https://webs.iiitd.edu.in/raghava/cellppd/pepsearch1.php?seq=THSWIPKRNR&thval=0.0) | 0.09 | 0.314132 | 0.224 | CPP |
| [DRFYRTCKLH](https://webs.iiitd.edu.in/raghava/cellppd/pepsearch1.php?seq=DRFYRTCKLH&thval=0.0) | 0.07 | 0.254063 | 0.5827 | non-CPP |
| [NLGQKRYTKT](https://webs.iiitd.edu.in/raghava/cellppd/pepsearch1.php?seq=NLGQKRYTKT&thval=0.0) | 0.06 | 0.772517 | 0.3366 | CPP |
| [RGDTQIQTRR](https://webs.iiitd.edu.in/raghava/cellppd/pepsearch1.php?seq=RGDTQIQTRR&thval=0.0) | 0.06 | 0.625262 | 0.3689 | CPP |
| [RRSFEIKKLW](https://webs.iiitd.edu.in/raghava/cellppd/pepsearch1.php?seq=RRSFEIKKLW&thval=0.0) | 0.06 | 0.967512 | 0.2881 | CPP |
| [RARIDARIDF](https://webs.iiitd.edu.in/raghava/cellppd/pepsearch1.php?seq=RARIDARIDF&thval=0.0) | 0.06 | 0.308162 | 0.2242 | CPP |
| [KRSYLIRALT](https://webs.iiitd.edu.in/raghava/cellppd/pepsearch1.php?seq=KRSYLIRALT&thval=0.0) | 0.04 | 0.938347 | 0.133 | CPP |
| [KRYTKTTYWW](https://webs.iiitd.edu.in/raghava/cellppd/pepsearch1.php?seq=KRYTKTTYWW&thval=0.0) | 0.04 | 0.988894 | 0.396 | CPP |
| [TRRSFEIKKL](https://webs.iiitd.edu.in/raghava/cellppd/pepsearch1.php?seq=TRRSFEIKKL&thval=0.0) | 0.04 | 0.966977 | 0.229 | CPP |
| [RKKIEKIRPL](https://webs.iiitd.edu.in/raghava/cellppd/pepsearch1.php?seq=RKKIEKIRPL&thval=0.0) | 0.03 | 0.952453 | 0.1191 | CPP |
| [GVDRFYRTCK](https://webs.iiitd.edu.in/raghava/cellppd/pepsearch1.php?seq=GVDRFYRTCK&thval=0.0) | 0.02 | 0.246222 | 0.6121 | non-CPP |
| [PSSSYRRPVG](https://webs.iiitd.edu.in/raghava/cellppd/pepsearch1.php?seq=PSSSYRRPVG&thval=0.0) | 0.02 | 0.367792 | 0.1728 | CPP |
| [MVSRARIDAR](https://webs.iiitd.edu.in/raghava/cellppd/pepsearch1.php?seq=MVSRARIDAR&thval=0.0) | 0.02 | 0.541798 | 0.3035 | CPP |
| [TQIQTRRSFE](https://webs.iiitd.edu.in/raghava/cellppd/pepsearch1.php?seq=TQIQTRRSFE&thval=0.0) | 0.01 | 0.614049 | 0.4535 | CPP |
| NEP |  |  |  |  |
| [FQDILLRMSK](https://webs.iiitd.edu.in/raghava/cellppd/pepsearch1.php?seq=FQDILLRMSK&thval=0.0) | 0.10 | 0.310246 | 0.4076 | CPP |
| [LQNRNEKWRE](https://webs.iiitd.edu.in/raghava/cellppd/pepsearch1.php?seq=LQNRNEKWRE&thval=0.0) | 0.00 | 0.352092 | 0.486 | CPP |

**Table S2. Predicted CPPs and their uptake efficiency using MLCPP web server.**

| **Epitope** | **Predicted Class by MLCPP** | **Uptake efficiency by MLCPP** | **Probability Uptake efficiency** |
| --- | --- | --- | --- |
| **NP** |  |  |  |
| RSRYWAIRTR | CPP | Low | 0.08610015 |
| RMIKRGINDR | CPP | Low | 0.0015004347 |
| GPIYRRVNGK | CPP | High | 0.9420671 |
| KGTKVVPRGK | CPP | Low | 0.11161355 |
| RRIWRQANNG | CPP | Low | 0.12637168 |
| YQRTRALVRT | CPP | High | 0.54660654 |
| RYWAIRTRSG | CPP | Low | 0.0006469945 |
| YRRVNGKWMR | CPP | High | 0.8408103 |
| GRKTRIAYER | CPP | Low | 0.019436885 |
| RTRALVRTGM | CPP | Low | 0.10272128 |
| SRYWAIRTRS | CPP | Low | 0.061379243 |
| HPSAGKDPKK | CPP | Low | 0.06393183 |
| KEEIRRIWRQ | CPP | Low | 0.035165217 |
| EIRRIWRQAN | CPP | Low | 0.49803308 |
| RGENGRKTRI | CPP | Low | 0.0026375542 |
| EEIRRIWRQA | CPP | Low | 0.08983125 |
| FLARSALILR | non-Cpp | - | - |
| ESSTLELRSR | CPP | Low | 0.014424583 |
| IRRIWRQANN | CPP | Low | 0.018466275 |
| RNFWRGENGR | CPP | Low | 0.00024154787 |
| RKTRIAYERM | CPP | Low | 0.0024465292 |
| LSAFDERRNK | CPP | Low | 0.4871673 |
| GKWMRELILY | non-Cpp | - | - |
| TYQRTRALVR | CPP | Low | 0.009827522 |
| RGINDRNFWR | CPP | Low | 0.023594065 |
| WRGENGRKTR | CPP | Low | 0.0010799407 |
| PSAGKDPKKT | CPP | Low | 0.1998218 |
| **HA** |  |  |  |
| [KNSYVNKKGK](https://webs.iiitd.edu.in/raghava/cellppd/pepsearch1.php?seq=KNSYVNKKGK&thval=0.0) | CPP | Low | 0.16539033 |
| [KLCRLKGIAP](https://webs.iiitd.edu.in/raghava/cellppd/pepsearch1.php?seq=KLCRLKGIAP&thval=0.0) | non-Cpp | - | - |
| [PEIAERPKVR](https://webs.iiitd.edu.in/raghava/cellppd/pepsearch1.php?seq=PEIAERPKVR&thval=0.0) | non-Cpp | - | - |
| [KLKNSYVNKK](https://webs.iiitd.edu.in/raghava/cellppd/pepsearch1.php?seq=KLKNSYVNKK&thval=0.0) | CPP | Low | 0.0069689807 |
| [PKYVRSAKLR](https://webs.iiitd.edu.in/raghava/cellppd/pepsearch1.php?seq=PKYVRSAKLR&thval=0.0) | non-Cpp | - | - |
| **Epitope** | **Predicted Class by MLCPP** | **Uptake efficiency by MLCPP** | **Probability Uptake efficiency** |
| [KYVRSAKLRM](https://webs.iiitd.edu.in/raghava/cellppd/pepsearch1.php?seq=KYVRSAKLRM&thval=0.0) | non-Cpp | - | - |
| [MNYYWTLLKP](https://webs.iiitd.edu.in/raghava/cellppd/pepsearch1.php?seq=MNYYWTLLKP&thval=0.0) | CPP | Low | 0.017377097 |
| [HNGKLCRLKG](https://webs.iiitd.edu.in/raghava/cellppd/pepsearch1.php?seq=HNGKLCRLKG&thval=0.0) | CPP | Low | 0.0032379034 |
| [GKLCRLKGIA](https://webs.iiitd.edu.in/raghava/cellppd/pepsearch1.php?seq=GKLCRLKGIA&thval=0.0) | CPP | Low | 0.12571323 |
| [GKEFNKLEKR](https://webs.iiitd.edu.in/raghava/cellppd/pepsearch1.php?seq=GKEFNKLEKR&thval=0.0) | CPP | Low | 0.11459363 |
| [RRFTPEIAER](https://webs.iiitd.edu.in/raghava/cellppd/pepsearch1.php?seq=RRFTPEIAER&thval=0.0) | CPP | Low | 0.058768854 |
| **NS1** |  |  |  |
| [RLRRDQKSLR](https://webs.iiitd.edu.in/raghava/cellppd/pepsearch1.php?seq=RLRRDQKSLR&thval=0.0) | CPP | Low | 0.04297347 |
| [RRDQKSLRGR](https://webs.iiitd.edu.in/raghava/cellppd/pepsearch1.php?seq=RRDQKSLRGR&thval=0.0) | CPP | Low | 0.0607516 |
| [FLDRLRRDQK](https://webs.iiitd.edu.in/raghava/cellppd/pepsearch1.php?seq=FLDRLRRDQK&thval=0.0) | CPP | Low | 0.2791845 |
| [RPPLTPKQKR](https://webs.iiitd.edu.in/raghava/cellppd/pepsearch1.php?seq=RPPLTPKQKR&thval=0.0) | CPP | Low | 0.7787175 |
| [DAPFLDRLRR](https://webs.iiitd.edu.in/raghava/cellppd/pepsearch1.php?seq=DAPFLDRLRR&thval=0.0) | CPP | Low | 0.15322384 |
| [DRLRRDQKSL](https://webs.iiitd.edu.in/raghava/cellppd/pepsearch1.php?seq=DRLRRDQKSL&thval=0.0) | CPP | Low | 0.0254691 |
| [FLWHVRKRVA](https://webs.iiitd.edu.in/raghava/cellppd/pepsearch1.php?seq=FLWHVRKRVA&thval=0.0) | CPP | Low | 0.115647994 |
| [DCFLWHVRKR](https://webs.iiitd.edu.in/raghava/cellppd/pepsearch1.php?seq=DCFLWHVRKR&thval=0.0) | CPP | Low | 0.019846726 |
| [APFLDRLRRD](https://webs.iiitd.edu.in/raghava/cellppd/pepsearch1.php?seq=APFLDRLRRD&thval=0.0) | CPP | Low | 0.0032625662 |
| [EWSMLIPKQK](https://webs.iiitd.edu.in/raghava/cellppd/pepsearch1.php?seq=EWSMLIPKQK&thval=0.0) | CPP | Low | 0.043113917 |
| [LQRFAWRSSN](https://webs.iiitd.edu.in/raghava/cellppd/pepsearch1.php?seq=LQRFAWRSSN&thval=0.0) | CPP | Low | 0.16217129 |
| [GKQIVERILK](https://webs.iiitd.edu.in/raghava/cellppd/pepsearch1.php?seq=GKQIVERILK&thval=0.0) | non-Cpp | - | - |
| [CFLWHVRKRV](https://webs.iiitd.edu.in/raghava/cellppd/pepsearch1.php?seq=CFLWHVRKRV&thval=0.0) | CPP | Low | 0.058601037 |
| **PB2** |  |  |  |
| [RRATAILRKA](https://webs.iiitd.edu.in/raghava/cellppd/pepsearch1.php?seq=RRATAILRKA&thval=0.0) | CPP | Low | 0.10791558 |
| [GRRATAILRK](https://webs.iiitd.edu.in/raghava/cellppd/pepsearch1.php?seq=GRRATAILRK&thval=0.0) | CPP | Low | 0.0011832521 |
| [RKATRRLIQL](https://webs.iiitd.edu.in/raghava/cellppd/pepsearch1.php?seq=RKATRRLIQL&thval=0.0) | CPP | Low | 0.0057017994 |
| [TAILRKATRR](https://webs.iiitd.edu.in/raghava/cellppd/pepsearch1.php?seq=TAILRKATRR&thval=0.0) | CPP | Low | 0.0036875831 |
| [KNPALRMKWM](https://webs.iiitd.edu.in/raghava/cellppd/pepsearch1.php?seq=KNPALRMKWM&thval=0.0) | CPP | Low | 0.010372712 |
| [RATAILRKAT](https://webs.iiitd.edu.in/raghava/cellppd/pepsearch1.php?seq=RATAILRKAT&thval=0.0) | CPP | Low | 0.08183215 |
| [ILRKATRRLI](https://webs.iiitd.edu.in/raghava/cellppd/pepsearch1.php?seq=ILRKATRRLI&thval=0.0) | CPP | Low | 0.000643615 |
| [AILRKATRRL](https://webs.iiitd.edu.in/raghava/cellppd/pepsearch1.php?seq=AILRKATRRL&thval=0.0) | CPP | Low | 7.36501e-05 |
| [SQTATKRIRM](https://webs.iiitd.edu.in/raghava/cellppd/pepsearch1.php?seq=SQTATKRIRM&thval=0.0) | CPP | Low | 0.034151956 |
| [ATAILRKATR](https://webs.iiitd.edu.in/raghava/cellppd/pepsearch1.php?seq=ATAILRKATR&thval=0.0) | CPP | Low | 0.0021576518 |
| [KATRRLIQLI](https://webs.iiitd.edu.in/raghava/cellppd/pepsearch1.php?seq=KATRRLIQLI&thval=0.0) | CPP | Low | 0.001080746 |
| [RANQRLNPMH](https://webs.iiitd.edu.in/raghava/cellppd/pepsearch1.php?seq=RANQRLNPMH&thval=0.0) | CPP | Low | 0.025480501 |
| [QSRTREILTK](https://webs.iiitd.edu.in/raghava/cellppd/pepsearch1.php?seq=QSRTREILTK&thval=0.0) | CPP | Low | 0.27660638 |
| [DRFLRVRDQR](https://webs.iiitd.edu.in/raghava/cellppd/pepsearch1.php?seq=DRFLRVRDQR&thval=0.0) | CPP | Low | 0.03438238 |
| [LRKATRRLIQ](https://webs.iiitd.edu.in/raghava/cellppd/pepsearch1.php?seq=LRKATRRLIQ&thval=0.0) | CPP | Low | 0.0033566977 |
| **Epitope** | **Predicted Class by MLCPP** | **Uptake efficiency by MLCPP** | **Probability Uptake efficiency** |
| [RLIQLIVSGR](https://webs.iiitd.edu.in/raghava/cellppd/pepsearch1.php?seq=RLIQLIVSGR&thval=0.0) | CPP | High | 0.74270374 |
| [AIIKKYTSGR](https://webs.iiitd.edu.in/raghava/cellppd/pepsearch1.php?seq=AIIKKYTSGR&thval=0.0) | CPP | High | 0.95666915 |
| [VGRRATAILR](https://webs.iiitd.edu.in/raghava/cellppd/pepsearch1.php?seq=VGRRATAILR&thval=0.0) | CPP | Low | 0.005339522 |
| [VNRANQRLNP](https://webs.iiitd.edu.in/raghava/cellppd/pepsearch1.php?seq=VNRANQRLNP&thval=0.0) | non-Cpp | - | - |
| [KKYTSGRQEK](https://webs.iiitd.edu.in/raghava/cellppd/pepsearch1.php?seq=KKYTSGRQEK&thval=0.0) | CPP | Low | 0.05159665 |
| [SQLTITKEKK](https://webs.iiitd.edu.in/raghava/cellppd/pepsearch1.php?seq=SQLTITKEKK&thval=0.0) | CPP | Low | 0.25104094 |
| [VRKTRFLPVA](https://webs.iiitd.edu.in/raghava/cellppd/pepsearch1.php?seq=VRKTRFLPVA&thval=0.0) | CPP | High | 0.5188882 |
| [DSQTATKRIR](https://webs.iiitd.edu.in/raghava/cellppd/pepsearch1.php?seq=DSQTATKRIR&thval=0.0) | CPP | Low | 0.33839735 |
| [LIIAARNIVR](https://webs.iiitd.edu.in/raghava/cellppd/pepsearch1.php?seq=LIIAARNIVR&thval=0.0) | CPP | Low | 0.1311531 |
| [FAAAPPKQSR](https://webs.iiitd.edu.in/raghava/cellppd/pepsearch1.php?seq=FAAAPPKQSR&thval=0.0) | CPP | Low | 0.040096838 |
| [KRITEMIPER](https://webs.iiitd.edu.in/raghava/cellppd/pepsearch1.php?seq=KRITEMIPER&thval=0.0) | CPP | High | 0.65697443 |
| [KRTSGSSVKR](https://webs.iiitd.edu.in/raghava/cellppd/pepsearch1.php?seq=KRTSGSSVKR&thval=0.0) | CPP | Low | 0.42772737 |
| [VSIDRFLRVR](https://webs.iiitd.edu.in/raghava/cellppd/pepsearch1.php?seq=VSIDRFLRVR&thval=0.0) | CPP | Low | 0.2358192 |
| [QIIKLLPFAA](https://webs.iiitd.edu.in/raghava/cellppd/pepsearch1.php?seq=QIIKLLPFAA&thval=0.0) | non-Cpp | - | - |
| [TATKRIRMAI](https://webs.iiitd.edu.in/raghava/cellppd/pepsearch1.php?seq=TATKRIRMAI&thval=0.0) | CPP | Low | 0.00050434674 |
| [MSMRGVRISK](https://webs.iiitd.edu.in/raghava/cellppd/pepsearch1.php?seq=MSMRGVRISK&thval=0.0) | non-Cpp | - | - |
| [NKATKRLTVL](https://webs.iiitd.edu.in/raghava/cellppd/pepsearch1.php?seq=NKATKRLTVL&thval=0.0) | CPP | Low | 0.00077984715 |
| [RNLMSQSRTR](https://webs.iiitd.edu.in/raghava/cellppd/pepsearch1.php?seq=RNLMSQSRTR&thval=0.0) | CPP | Low | 0.007253824 |
| [AQIIKLLPFA](https://webs.iiitd.edu.in/raghava/cellppd/pepsearch1.php?seq=AQIIKLLPFA&thval=0.0) | non-Cpp | - | - |
| [MHQLLRHFQK](https://webs.iiitd.edu.in/raghava/cellppd/pepsearch1.php?seq=MHQLLRHFQK&thval=0.0) | CPP | Low | 0.0010637514 |
| [KATKRLTVLG](https://webs.iiitd.edu.in/raghava/cellppd/pepsearch1.php?seq=KATKRLTVLG&thval=0.0) | CPP | Low | 5.4063545e-05 |
| [MKRKRDSSIL](https://webs.iiitd.edu.in/raghava/cellppd/pepsearch1.php?seq=MKRKRDSSIL&thval=0.0) | CPP | Low | 0.46125346 |
| [LVRKTRFLPV](https://webs.iiitd.edu.in/raghava/cellppd/pepsearch1.php?seq=LVRKTRFLPV&thval=0.0) | CPP | Low | 0.16725644 |
| [RKTRFLPVAG](https://webs.iiitd.edu.in/raghava/cellppd/pepsearch1.php?seq=RKTRFLPVAG&thval=0.0) | CPP | High | 0.5510806 |
| [RNIVRRAAVS](https://webs.iiitd.edu.in/raghava/cellppd/pepsearch1.php?seq=RNIVRRAAVS&thval=0.0) | CPP | Low | 0.0130923195 |
| [VLVMKRKRDS](https://webs.iiitd.edu.in/raghava/cellppd/pepsearch1.php?seq=VLVMKRKRDS&thval=0.0) | CPP | Low | 0.21296674 |
| [LLRHFQKDAK](https://webs.iiitd.edu.in/raghava/cellppd/pepsearch1.php?seq=LLRHFQKDAK&thval=0.0) | CPP | Low | 0.45969155 |
| [LLPFAAAPPK](https://webs.iiitd.edu.in/raghava/cellppd/pepsearch1.php?seq=LLPFAAAPPK&thval=0.0) | CPP | Low | 0.0042887316 |
| [MERIKELRNL](https://webs.iiitd.edu.in/raghava/cellppd/pepsearch1.php?seq=MERIKELRNL&thval=0.0) | non-Cpp | - | - |
| [RRRVDINPGH](https://webs.iiitd.edu.in/raghava/cellppd/pepsearch1.php?seq=RRRVDINPGH&thval=0.0) | CPP | Low | 0.0008894238 |
| [ATKRLTVLGK](https://webs.iiitd.edu.in/raghava/cellppd/pepsearch1.php?seq=ATKRLTVLGK&thval=0.0) | CPP | Low | 0.00018777845 |
| **M2** |  |  |  |
| [KCIYRRFKYG](https://webs.iiitd.edu.in/raghava/cellppd/pepsearch1.php?seq=KCIYRRFKYG&thval=0.0) | CPP | High | 0.96097726 |
| [RLFFKCIYRR](https://webs.iiitd.edu.in/raghava/cellppd/pepsearch1.php?seq=RLFFKCIYRR&thval=0.0) | CPP | Low | 0.13432483 |
| [IYRRFKYGLK](https://webs.iiitd.edu.in/raghava/cellppd/pepsearch1.php?seq=IYRRFKYGLK&thval=0.0) | CPP | High | 0.9108882 |
| [YRRFKYGLKG](https://webs.iiitd.edu.in/raghava/cellppd/pepsearch1.php?seq=YRRFKYGLKG&thval=0.0) | CPP | Low | 0.31795976 |
| **Epitope** | **Predicted Class by MLCPP** | **Uptake efficiency by MLCPP** | **Probability Uptake efficiency** |
| [FFKCIYRRFK](https://webs.iiitd.edu.in/raghava/cellppd/pepsearch1.php?seq=FFKCIYRRFK&thval=0.0) | CPP | High | 0.9004561 |
| [FKCIYRRFKY](https://webs.iiitd.edu.in/raghava/cellppd/pepsearch1.php?seq=FKCIYRRFKY&thval=0.0) | CPP | High | 0.84529793 |
| [CIYRRFKYGL](https://webs.iiitd.edu.in/raghava/cellppd/pepsearch1.php?seq=CIYRRFKYGL&thval=0.0) | CPP | High | 0.94066703 |
| [WILDRLFFKC](https://webs.iiitd.edu.in/raghava/cellppd/pepsearch1.php?seq=WILDRLFFKC&thval=0.0) | CPP | Low | 0.009057058 |
| [LWILDRLFFK](https://webs.iiitd.edu.in/raghava/cellppd/pepsearch1.php?seq=LWILDRLFFK&thval=0.0) | CPP | Low | 0.13271984 |
| **NA** |  |  |  |
| [IKSWRKKILR](https://webs.iiitd.edu.in/raghava/cellppd/pepsearch1.php?seq=IKSWRKKILR&thval=0.0) | CPP | Low | 0.4967278 |
| [ELIRGRPKEK](https://webs.iiitd.edu.in/raghava/cellppd/pepsearch1.php?seq=ELIRGRPKEK&thval=0.0) | CPP | Low | 0.04539804 |
| [YKIFKIEKGK](https://webs.iiitd.edu.in/raghava/cellppd/pepsearch1.php?seq=YKIFKIEKGK&thval=0.0) | CPP | Low | 0.35251254 |
| [ETIKSWRKKI](https://webs.iiitd.edu.in/raghava/cellppd/pepsearch1.php?seq=ETIKSWRKKI&thval=0.0) | CPP | High | 0.5745883 |
| [TIKSWRKKIL](https://webs.iiitd.edu.in/raghava/cellppd/pepsearch1.php?seq=TIKSWRKKIL&thval=0.0) | CPP | High | 0.5817866 |
| [KIFKIEKGKV](https://webs.iiitd.edu.in/raghava/cellppd/pepsearch1.php?seq=KIFKIEKGKV&thval=0.0) | CPP | Low | 0.2785626 |
| [LIRGRPKEKT](https://webs.iiitd.edu.in/raghava/cellppd/pepsearch1.php?seq=LIRGRPKEKT&thval=0.0) | CPP | Low | 0.0038192477 |
| [TETIKSWRKK](https://webs.iiitd.edu.in/raghava/cellppd/pepsearch1.php?seq=TETIKSWRKK&thval=0.0) | CPP | Low | 0.033265512 |
| [KIEKGKVTKS](https://webs.iiitd.edu.in/raghava/cellppd/pepsearch1.php?seq=KIEKGKVTKS&thval=0.0) | CPP | Low | 0.4003408 |
| [GVWIGRTKSH](https://webs.iiitd.edu.in/raghava/cellppd/pepsearch1.php?seq=GVWIGRTKSH&thval=0.0) | CPP | Low | 0.023228277 |
| [FWVELIRGRP](https://webs.iiitd.edu.in/raghava/cellppd/pepsearch1.php?seq=FWVELIRGRP&thval=0.0) | CPP | Low | 0.0028522129 |
| [PIRGWAIYSK](https://webs.iiitd.edu.in/raghava/cellppd/pepsearch1.php?seq=PIRGWAIYSK&thval=0.0) | CPP | Low | 0.0026042378 |
| **PB1-F2** |  |  |  |
| [KTRVLKRWRL](https://webs.iiitd.edu.in/raghava/cellppd/pepsearch1.php?seq=KTRVLKRWRL&thval=0.0) | CPP | High | 0.8651146 |
| [LKRWRLFSKH](https://webs.iiitd.edu.in/raghava/cellppd/pepsearch1.php?seq=LKRWRLFSKH&thval=0.0) | CPP | High | 0.69275504 |
| [VLKRWRLFSK](https://webs.iiitd.edu.in/raghava/cellppd/pepsearch1.php?seq=VLKRWRLFSK&thval=0.0) | CPP | Low | 0.0803874 |
| [LKTRVLKRWR](https://webs.iiitd.edu.in/raghava/cellppd/pepsearch1.php?seq=LKTRVLKRWR&thval=0.0) | CPP | High | 0.9634459 |
| [FLKTRVLKRW](https://webs.iiitd.edu.in/raghava/cellppd/pepsearch1.php?seq=FLKTRVLKRW&thval=0.0) | CPP | Low | 0.05589191 |
| [RVLKRWRLFS](https://webs.iiitd.edu.in/raghava/cellppd/pepsearch1.php?seq=RVLKRWRLFS&thval=0.0) | CPP | High | 0.9759319 |
| [KRWRLFSKHE](https://webs.iiitd.edu.in/raghava/cellppd/pepsearch1.php?seq=KRWRLFSKHE&thval=0.0) | CPP | High | 0.86983955 |
| [WKQWLSLRNP](https://webs.iiitd.edu.in/raghava/cellppd/pepsearch1.php?seq=WKQWLSLRNP&thval=0.0) | CPP | High | 0.9567846 |
| [TRVLKRWRLF](https://webs.iiitd.edu.in/raghava/cellppd/pepsearch1.php?seq=TRVLKRWRLF&thval=0.0) | CPP | Low | 0.020524835 |
| [KQWLSLRNPI](https://webs.iiitd.edu.in/raghava/cellppd/pepsearch1.php?seq=KQWLSLRNPI&thval=0.0) | CPP | Low | 0.2330274 |
| [RNSTRLMGHC](https://webs.iiitd.edu.in/raghava/cellppd/pepsearch1.php?seq=RNSTRLMGHC&thval=0.0) | CPP | High | 0.5204829 |
| [GQQTPKLEHR](https://webs.iiitd.edu.in/raghava/cellppd/pepsearch1.php?seq=GQQTPKLEHR&thval=0.0) | CPP | High | 0.99631894 |
| [KQIVYWKQWL](https://webs.iiitd.edu.in/raghava/cellppd/pepsearch1.php?seq=KQIVYWKQWL&thval=0.0) | CPP | Low | 0.12410101 |
| [HRNSTRLMGH](https://webs.iiitd.edu.in/raghava/cellppd/pepsearch1.php?seq=HRNSTRLMGH&thval=0.0) | CPP | Low | 0.046717048 |
| [VYWKQWLSLR](https://webs.iiitd.edu.in/raghava/cellppd/pepsearch1.php?seq=VYWKQWLSLR&thval=0.0) | CPP | Low | 0.07367178 |
| **PA** |  |  |  |
| [KIPKTKNMKK](https://webs.iiitd.edu.in/raghava/cellppd/pepsearch1.php?seq=KIPKTKNMKK&thval=0.0) | CPP | High | 0.9730952 |
| **Epitope** | **Predicted Class by MLCPP** | **Uptake efficiency by MLCPP** | **Probability Uptake efficiency** |
| [RIKTRLFTIR](https://webs.iiitd.edu.in/raghava/cellppd/pepsearch1.php?seq=RIKTRLFTIR&thval=0.0) | CPP | Low | 0.0141728055 |
| [KTTPRPLRLP](https://webs.iiitd.edu.in/raghava/cellppd/pepsearch1.php?seq=KTTPRPLRLP&thval=0.0) | CPP | High | 0.9351717 |
| [ESRARIKTRL](https://webs.iiitd.edu.in/raghava/cellppd/pepsearch1.php?seq=ESRARIKTRL&thval=0.0) | CPP | Low | 0.018628335 |
| [RARIKTRLFT](https://webs.iiitd.edu.in/raghava/cellppd/pepsearch1.php?seq=RARIKTRLFT&thval=0.0) | CPP | Low | 0.010101924 |
| [FLKTTPRPLR](https://webs.iiitd.edu.in/raghava/cellppd/pepsearch1.php?seq=FLKTTPRPLR&thval=0.0) | CPP | Low | 0.022842897 |
| [FIIKGRSHLR](https://webs.iiitd.edu.in/raghava/cellppd/pepsearch1.php?seq=FIIKGRSHLR&thval=0.0) | CPP | Low | 0.030095981 |
| [LKTTPRPLRL](https://webs.iiitd.edu.in/raghava/cellppd/pepsearch1.php?seq=LKTTPRPLRL&thval=0.0) | CPP | Low | 0.33858475 |
| [PRPLRLPNGP](https://webs.iiitd.edu.in/raghava/cellppd/pepsearch1.php?seq=PRPLRLPNGP&thval=0.0) | CPP | Low | 0.148261 |
| [KNMKKTSQLK](https://webs.iiitd.edu.in/raghava/cellppd/pepsearch1.php?seq=KNMKKTSQLK&thval=0.0) | CPP | High | 0.568485 |
| [EKIPKTKNMK](https://webs.iiitd.edu.in/raghava/cellppd/pepsearch1.php?seq=EKIPKTKNMK&thval=0.0) | CPP | Low | 0.06137005 |
| [GKVCRTLLAK](https://webs.iiitd.edu.in/raghava/cellppd/pepsearch1.php?seq=GKVCRTLLAK&thval=0.0) | CPP | Low | 0.08940117 |
| [SRARIKTRLF](https://webs.iiitd.edu.in/raghava/cellppd/pepsearch1.php?seq=SRARIKTRLF&thval=0.0) | CPP | Low | 0.0087242415 |
| [KKTSQLKWAL](https://webs.iiitd.edu.in/raghava/cellppd/pepsearch1.php?seq=KKTSQLKWAL&thval=0.0) | CPP | Low | 0.026231198 |
| [KGRSHLRNDT](https://webs.iiitd.edu.in/raghava/cellppd/pepsearch1.php?seq=KGRSHLRNDT&thval=0.0) | CPP | Low | 0.03203429 |
| [TTPRPLRLPN](https://webs.iiitd.edu.in/raghava/cellppd/pepsearch1.php?seq=TTPRPLRLPN&thval=0.0) | CPP | High | 0.88072115 |
| [ARIKTRLFTI](https://webs.iiitd.edu.in/raghava/cellppd/pepsearch1.php?seq=ARIKTRLFTI&thval=0.0) | CPP | Low | 0.08195846 |
| [KCMRTFFGWK](https://webs.iiitd.edu.in/raghava/cellppd/pepsearch1.php?seq=KCMRTFFGWK&thval=0.0) | CPP | High | 0.5757508 |
| [EESRARIKTR](https://webs.iiitd.edu.in/raghava/cellppd/pepsearch1.php?seq=EESRARIKTR&thval=0.0) | CPP | Low | 0.007795441 |
| [PFLKTTPRPL](https://webs.iiitd.edu.in/raghava/cellppd/pepsearch1.php?seq=PFLKTTPRPL&thval=0.0) | CPP | Low | 0.01875168 |
| [NGTSKIKMKW](https://webs.iiitd.edu.in/raghava/cellppd/pepsearch1.php?seq=NGTSKIKMKW&thval=0.0) | CPP | Low | 0.1349617 |
| [TPRPLRLPNG](https://webs.iiitd.edu.in/raghava/cellppd/pepsearch1.php?seq=TPRPLRLPNG&thval=0.0) | CPP | Low | 0.08355592 |
| [KTKNMKKTSQ](https://webs.iiitd.edu.in/raghava/cellppd/pepsearch1.php?seq=KTKNMKKTSQ&thval=0.0) | CPP | High | 0.7990742 |
| [HIYYLEKANK](https://webs.iiitd.edu.in/raghava/cellppd/pepsearch1.php?seq=HIYYLEKANK&thval=0.0) | CPP | Low | 0.0029348219 |
| [KMKWGMEMRR](https://webs.iiitd.edu.in/raghava/cellppd/pepsearch1.php?seq=KMKWGMEMRR&thval=0.0) | non-Cpp | - | - |
| [KVCRTLLAKS](https://webs.iiitd.edu.in/raghava/cellppd/pepsearch1.php?seq=KVCRTLLAKS&thval=0.0) | CPP | High | 0.64026225 |
| [RFIEIGVTRR](https://webs.iiitd.edu.in/raghava/cellppd/pepsearch1.php?seq=RFIEIGVTRR&thval=0.0) | CPP | Low | 0.0004843474 |
| [TKEGRRKTNL](https://webs.iiitd.edu.in/raghava/cellppd/pepsearch1.php?seq=TKEGRRKTNL&thval=0.0) | CPP | High | 0.91495895 |
| [PLRLPNGPPC](https://webs.iiitd.edu.in/raghava/cellppd/pepsearch1.php?seq=PLRLPNGPPC&thval=0.0) | CPP | Low | 0.4389023 |
| [PKTKNMKKTS](https://webs.iiitd.edu.in/raghava/cellppd/pepsearch1.php?seq=PKTKNMKKTS&thval=0.0) | CPP | Low | 0.32222143 |
| [HCRATEYIMK](https://webs.iiitd.edu.in/raghava/cellppd/pepsearch1.php?seq=HCRATEYIMK&thval=0.0) | non-Cpp | - | - |
| [KEGRRKTNLY](https://webs.iiitd.edu.in/raghava/cellppd/pepsearch1.php?seq=KEGRRKTNLY&thval=0.0) | CPP | Low | 0.04528389 |
| **M1** |  |  |  |
| [KAVKLYRKLK](https://webs.iiitd.edu.in/raghava/cellppd/pepsearch1.php?seq=KAVKLYRKLK&thval=0.0) | CPP | Low | 0.3979155 |
| [RGLQRRRFVQ](https://webs.iiitd.edu.in/raghava/cellppd/pepsearch1.php?seq=RGLQRRRFVQ&thval=0.0) | CPP | Low | 0.0769112 |
| [KLYRKLKREI](https://webs.iiitd.edu.in/raghava/cellppd/pepsearch1.php?seq=KLYRKLKREI&thval=0.0) | CPP | Low | 0.10402707 |
| [AVKLYRKLKR](https://webs.iiitd.edu.in/raghava/cellppd/pepsearch1.php?seq=AVKLYRKLKR&thval=0.0) | CPP | Low | 0.4842886 |
| **Epitope** | **Predicted Class by MLCPP** | **Uptake efficiency by MLCPP** | **Probability Uptake efficiency** |
| [LQRRRFVQNA](https://webs.iiitd.edu.in/raghava/cellppd/pepsearch1.php?seq=LQRRRFVQNA&thval=0.0) | CPP | Low | 0.4965157 |
| [RKLKREITFH](https://webs.iiitd.edu.in/raghava/cellppd/pepsearch1.php?seq=RKLKREITFH&thval=0.0) | CPP | Low | 0.30892646 |
| [GLQRRRFVQN](https://webs.iiitd.edu.in/raghava/cellppd/pepsearch1.php?seq=GLQRRRFVQN&thval=0.0) | CPP | Low | 0.019338774 |
| [VKLYRKLKRE](https://webs.iiitd.edu.in/raghava/cellppd/pepsearch1.php?seq=VKLYRKLKRE&thval=0.0) | CPP | Low | 0.08830796 |
| [ERGLQRRRFV](https://webs.iiitd.edu.in/raghava/cellppd/pepsearch1.php?seq=ERGLQRRRFV&thval=0.0) | CPP | Low | 0.056321766 |
| [WLKTRPILSP](https://webs.iiitd.edu.in/raghava/cellppd/pepsearch1.php?seq=WLKTRPILSP&thval=0.0) | CPP | Low | 0.004475281 |
| [DKAVKLYRKL](https://webs.iiitd.edu.in/raghava/cellppd/pepsearch1.php?seq=DKAVKLYRKL&thval=0.0) | CPP | Low | 0.185663 |
| [MDKAVKLYRK](https://webs.iiitd.edu.in/raghava/cellppd/pepsearch1.php?seq=MDKAVKLYRK&thval=0.0) | CPP | Low | 0.34526846 |
| **PA-X** |  |  |  |
| [RASCLKCPKK](https://webs.iiitd.edu.in/raghava/cellppd/pepsearch1.php?seq=RASCLKCPKK&thval=0.0) | CPP | Low | 0.4408803 |
| [EKRQLKKGLK](https://webs.iiitd.edu.in/raghava/cellppd/pepsearch1.php?seq=EKRQLKKGLK&thval=0.0) | CPP | High | 0.57627815 |
| [RIKTRLFTIR](https://webs.iiitd.edu.in/raghava/cellppd/pepsearch1.php?seq=RIKTRLFTIR&thval=0.0) | CPP | Low | 0.0141728055 |
| [KRQLKKGLKS](https://webs.iiitd.edu.in/raghava/cellppd/pepsearch1.php?seq=KRQLKKGLKS&thval=0.0) | CPP | High | 0.52424765 |
| [ESRARIKTRL](https://webs.iiitd.edu.in/raghava/cellppd/pepsearch1.php?seq=ESRARIKTRL&thval=0.0) | CPP | Low | 0.018628335 |
| [RARIKTRLFT](https://webs.iiitd.edu.in/raghava/cellppd/pepsearch1.php?seq=RARIKTRLFT&thval=0.0) | CPP | Low | 0.010101924 |
| [RQLKKGLKSQ](https://webs.iiitd.edu.in/raghava/cellppd/pepsearch1.php?seq=RQLKKGLKSQ&thval=0.0) | CPP | High | 0.9041738 |
| [REEKRQLKKG](https://webs.iiitd.edu.in/raghava/cellppd/pepsearch1.php?seq=REEKRQLKKG&thval=0.0) | CPP | Low | 0.039454013 |
| [SRARIKTRLF](https://webs.iiitd.edu.in/raghava/cellppd/pepsearch1.php?seq=SRARIKTRLF&thval=0.0) | CPP | Low | 0.0087242415 |
| [PREEKRQLKK](https://webs.iiitd.edu.in/raghava/cellppd/pepsearch1.php?seq=PREEKRQLKK&thval=0.0) | CPP | Low | 0.0032261468 |
| [LRASCLKCPK](https://webs.iiitd.edu.in/raghava/cellppd/pepsearch1.php?seq=LRASCLKCPK&thval=0.0) | CPP | Low | 0.027496977 |
| [KVSRRTSPAL](https://webs.iiitd.edu.in/raghava/cellppd/pepsearch1.php?seq=KVSRRTSPAL&thval=0.0) | CPP | Low | 0.021341622 |
| [ARIKTRLFTI](https://webs.iiitd.edu.in/raghava/cellppd/pepsearch1.php?seq=ARIKTRLFTI&thval=0.0) | CPP | Low | 0.08195846 |
| [PTKVSRRTSP](https://webs.iiitd.edu.in/raghava/cellppd/pepsearch1.php?seq=PTKVSRRTSP&thval=0.0) | CPP | High | 0.72708595 |
| [VSRRTSPALK](https://webs.iiitd.edu.in/raghava/cellppd/pepsearch1.php?seq=VSRRTSPALK&thval=0.0) | CPP | Low | 0.013248629 |
| [NRTATLRASC](https://webs.iiitd.edu.in/raghava/cellppd/pepsearch1.php?seq=NRTATLRASC&thval=0.0) | CPP | Low | 0.27128464 |
| [EESRARIKTR](https://webs.iiitd.edu.in/raghava/cellppd/pepsearch1.php?seq=EESRARIKTR&thval=0.0) | CPP | Low | 0.007795441 |
| [SRRTSPALKI](https://webs.iiitd.edu.in/raghava/cellppd/pepsearch1.php?seq=SRRTSPALKI&thval=0.0) | CPP | Low | 0.022487085 |
| [SNRTATLRAS](https://webs.iiitd.edu.in/raghava/cellppd/pepsearch1.php?seq=SNRTATLRAS&thval=0.0) | CPP | Low | 0.053759426 |
| [HIYYLEKANK](https://webs.iiitd.edu.in/raghava/cellppd/pepsearch1.php?seq=HIYYLEKANK&thval=0.0) | CPP | Low | 0.0029348219 |
| [RFIEIGVTRR](https://webs.iiitd.edu.in/raghava/cellppd/pepsearch1.php?seq=RFIEIGVTRR&thval=0.0) | CPP | Low | 0.0004843474 |
| [ASLPTKVSRR](https://webs.iiitd.edu.in/raghava/cellppd/pepsearch1.php?seq=ASLPTKVSRR&thval=0.0) | CPP | High | 0.6343591 |
| [TKVSRRTSPA](https://webs.iiitd.edu.in/raghava/cellppd/pepsearch1.php?seq=TKVSRRTSPA&thval=0.0) | CPP | Low | 0.45996445 |
| [RRTSPALKIL](https://webs.iiitd.edu.in/raghava/cellppd/pepsearch1.php?seq=RRTSPALKIL&thval=0.0) | CPP | Low | 0.001928574 |
| [SPREEKRQLK](https://webs.iiitd.edu.in/raghava/cellppd/pepsearch1.php?seq=SPREEKRQLK&thval=0.0) | CPP | Low | 0.18840447 |
| PB1 |  |  |  |
| [FQRKRRVRDN](https://webs.iiitd.edu.in/raghava/cellppd/pepsearch1.php?seq=FQRKRRVRDN&thval=0.0) | CPP | Low | 0.34227335 |
| **Epitope** | **Predicted Class by MLCPP** | **Uptake efficiency by MLCPP** | **Probability Uptake efficiency** |
| [THFQRKRRVR](https://webs.iiitd.edu.in/raghava/cellppd/pepsearch1.php?seq=THFQRKRRVR&thval=0.0) | CPP | Low | 0.1661719 |
| [KMITQRTIGK](https://webs.iiitd.edu.in/raghava/cellppd/pepsearch1.php?seq=KMITQRTIGK&thval=0.0) | CPP | Low | 0.09273988 |
| [RRVRDNMTKK](https://webs.iiitd.edu.in/raghava/cellppd/pepsearch1.php?seq=RRVRDNMTKK&thval=0.0) | CPP | Low | 0.026920518 |
| [GITTHFQRKR](https://webs.iiitd.edu.in/raghava/cellppd/pepsearch1.php?seq=GITTHFQRKR&thval=0.0) | CPP | Low | 0.0011615106 |
| [HFQRKRRVRD](https://webs.iiitd.edu.in/raghava/cellppd/pepsearch1.php?seq=HFQRKRRVRD&thval=0.0) | CPP | Low | 0.082075946 |
| [KKIEKIRPLL](https://webs.iiitd.edu.in/raghava/cellppd/pepsearch1.php?seq=KKIEKIRPLL&thval=0.0) | CPP | Low | 0.005651606 |
| [RLNKRSYLIR](https://webs.iiitd.edu.in/raghava/cellppd/pepsearch1.php?seq=RLNKRSYLIR&thval=0.0) | CPP | Low | 0.35210693 |
| [DAERGKLKRR](https://webs.iiitd.edu.in/raghava/cellppd/pepsearch1.php?seq=DAERGKLKRR&thval=0.0) | CPP | Low | 0.014366756 |
| [ERGKLKRRAI](https://webs.iiitd.edu.in/raghava/cellppd/pepsearch1.php?seq=ERGKLKRRAI&thval=0.0) | CPP | Low | 0.3468235 |
| [KDAERGKLKR](https://webs.iiitd.edu.in/raghava/cellppd/pepsearch1.php?seq=KDAERGKLKR&thval=0.0) | CPP | Low | 0.009762266 |
| [QRKRRVRDNM](https://webs.iiitd.edu.in/raghava/cellppd/pepsearch1.php?seq=QRKRRVRDNM&thval=0.0) | CPP | Low | 0.2236256 |
| [AERGKLKRRA](https://webs.iiitd.edu.in/raghava/cellppd/pepsearch1.php?seq=AERGKLKRRA&thval=0.0) | CPP | Low | 0.005663855 |
| [KRRVRDNMTK](https://webs.iiitd.edu.in/raghava/cellppd/pepsearch1.php?seq=KRRVRDNMTK&thval=0.0) | CPP | Low | 0.09777354 |
| [TQRTIGKRKQ](https://webs.iiitd.edu.in/raghava/cellppd/pepsearch1.php?seq=TQRTIGKRKQ&thval=0.0) | CPP | High | 0.9864139 |
| [RGKLKRRAIA](https://webs.iiitd.edu.in/raghava/cellppd/pepsearch1.php?seq=RGKLKRRAIA&thval=0.0) | CPP | High | 0.7837708 |
| [SWIPKRNRSI](https://webs.iiitd.edu.in/raghava/cellppd/pepsearch1.php?seq=SWIPKRNRSI&thval=0.0) | CPP | High | 0.9106479 |
| [MITQRTIGKR](https://webs.iiitd.edu.in/raghava/cellppd/pepsearch1.php?seq=MITQRTIGKR&thval=0.0) | CPP | Low | 0.0026274156 |
| [GKLKRRAIAT](https://webs.iiitd.edu.in/raghava/cellppd/pepsearch1.php?seq=GKLKRRAIAT&thval=0.0) | CPP | High | 0.8124223 |
| [KRRAIATPGM](https://webs.iiitd.edu.in/raghava/cellppd/pepsearch1.php?seq=KRRAIATPGM&thval=0.0) | non-Cpp | - | - |
| [KKLWEQTRSK](https://webs.iiitd.edu.in/raghava/cellppd/pepsearch1.php?seq=KKLWEQTRSK&thval=0.0) | CPP | Low | 0.10689548 |
| [ITTHFQRKRR](https://webs.iiitd.edu.in/raghava/cellppd/pepsearch1.php?seq=ITTHFQRKRR&thval=0.0) | CPP | High | 0.50204945 |
| [TTHFQRKRRV](https://webs.iiitd.edu.in/raghava/cellppd/pepsearch1.php?seq=TTHFQRKRRV&thval=0.0) | CPP | High | 0.5422515 |
| [RKRRVRDNMT](https://webs.iiitd.edu.in/raghava/cellppd/pepsearch1.php?seq=RKRRVRDNMT&thval=0.0) | CPP | Low | 0.14544499 |
| [KSMKLRTQIP](https://webs.iiitd.edu.in/raghava/cellppd/pepsearch1.php?seq=KSMKLRTQIP&thval=0.0) | CPP | High | 0.88957304 |
| [TRKKIEKIRP](https://webs.iiitd.edu.in/raghava/cellppd/pepsearch1.php?seq=TRKKIEKIRP&thval=0.0) | CPP | High | 0.70477086 |
| [HSWIPKRNRS](https://webs.iiitd.edu.in/raghava/cellppd/pepsearch1.php?seq=HSWIPKRNRS&thval=0.0) | CPP | High | 0.9542241 |
| [WIPKRNRSIL](https://webs.iiitd.edu.in/raghava/cellppd/pepsearch1.php?seq=WIPKRNRSIL&thval=0.0) | CPP | High | 0.538253 |
| [STRKKIEKIR](https://webs.iiitd.edu.in/raghava/cellppd/pepsearch1.php?seq=STRKKIEKIR&thval=0.0) | CPP | Low | 0.28518024 |
| [MGITTHFQRK](https://webs.iiitd.edu.in/raghava/cellppd/pepsearch1.php?seq=MGITTHFQRK&thval=0.0) | CPP | Low | 0.05705954 |
| [KYFNDSTRKK](https://webs.iiitd.edu.in/raghava/cellppd/pepsearch1.php?seq=KYFNDSTRKK&thval=0.0) | CPP | Low | 0.022195732 |
| [NDSTRKKIEK](https://webs.iiitd.edu.in/raghava/cellppd/pepsearch1.php?seq=NDSTRKKIEK&thval=0.0) | CPP | Low | 0.017657328 |
| [KIEKIRPLLI](https://webs.iiitd.edu.in/raghava/cellppd/pepsearch1.php?seq=KIEKIRPLLI&thval=0.0) | CPP | Low | 0.06540234 |
| [KLWEQTRSKA](https://webs.iiitd.edu.in/raghava/cellppd/pepsearch1.php?seq=KLWEQTRSKA&thval=0.0) | non-Cpp | - | - |
| [KLKRRAIATP](https://webs.iiitd.edu.in/raghava/cellppd/pepsearch1.php?seq=KLKRRAIATP&thval=0.0) | CPP | Low | 0.051993772 |
| [GINMSKKKSY](https://webs.iiitd.edu.in/raghava/cellppd/pepsearch1.php?seq=GINMSKKKSY&thval=0.0) | CPP | Low | 0.056290444 |
| [ITQRTIGKRK](https://webs.iiitd.edu.in/raghava/cellppd/pepsearch1.php?seq=ITQRTIGKRK&thval=0.0) | CPP | Low | 0.46160167 |
| **Epitope** | **Predicted Class by MLCPP** | **Uptake efficiency by MLCPP** | **Probability Uptake efficiency** |
| [MSKKKSYINR](https://webs.iiitd.edu.in/raghava/cellppd/pepsearch1.php?seq=MSKKKSYINR&thval=0.0) | CPP | Low | 0.44668812 |
| [RQTYDWTLNR](https://webs.iiitd.edu.in/raghava/cellppd/pepsearch1.php?seq=RQTYDWTLNR&thval=0.0) | CPP | Low | 0.048720352 |
| [QTRRSFEIKK](https://webs.iiitd.edu.in/raghava/cellppd/pepsearch1.php?seq=QTRRSFEIKK&thval=0.0) | CPP | Low | 0.2023194 |
| [THSWIPKRNR](https://webs.iiitd.edu.in/raghava/cellppd/pepsearch1.php?seq=THSWIPKRNR&thval=0.0) | CPP | Low | 0.40051278 |
| [DRFYRTCKLH](https://webs.iiitd.edu.in/raghava/cellppd/pepsearch1.php?seq=DRFYRTCKLH&thval=0.0) | CPP | Low | 0.0003330288 |
| [NLGQKRYTKT](https://webs.iiitd.edu.in/raghava/cellppd/pepsearch1.php?seq=NLGQKRYTKT&thval=0.0) | CPP | High | 0.715216 |
| [RGDTQIQTRR](https://webs.iiitd.edu.in/raghava/cellppd/pepsearch1.php?seq=RGDTQIQTRR&thval=0.0) | CPP | Low | 0.0038314422 |
| [RRSFEIKKLW](https://webs.iiitd.edu.in/raghava/cellppd/pepsearch1.php?seq=RRSFEIKKLW&thval=0.0) | CPP | Low | 0.021220228 |
| [RARIDARIDF](https://webs.iiitd.edu.in/raghava/cellppd/pepsearch1.php?seq=RARIDARIDF&thval=0.0) | CPP | Low | 0.00015148155 |
| [KRSYLIRALT](https://webs.iiitd.edu.in/raghava/cellppd/pepsearch1.php?seq=KRSYLIRALT&thval=0.0) | CPP | Low | 0.008845749 |
| [KRYTKTTYWW](https://webs.iiitd.edu.in/raghava/cellppd/pepsearch1.php?seq=KRYTKTTYWW&thval=0.0) | CPP | High | 0.8910054 |
| [TRRSFEIKKL](https://webs.iiitd.edu.in/raghava/cellppd/pepsearch1.php?seq=TRRSFEIKKL&thval=0.0) | CPP | High | 0.6137328 |
| [RKKIEKIRPL](https://webs.iiitd.edu.in/raghava/cellppd/pepsearch1.php?seq=RKKIEKIRPL&thval=0.0) | CPP | Low | 0.44784606 |
| [GVDRFYRTCK](https://webs.iiitd.edu.in/raghava/cellppd/pepsearch1.php?seq=GVDRFYRTCK&thval=0.0) | CPP | Low | 0.0033262498 |
| [PSSSYRRPVG](https://webs.iiitd.edu.in/raghava/cellppd/pepsearch1.php?seq=PSSSYRRPVG&thval=0.0) | CPP | Low | 0.3398981 |
| [MVSRARIDAR](https://webs.iiitd.edu.in/raghava/cellppd/pepsearch1.php?seq=MVSRARIDAR&thval=0.0) | CPP | Low | 0.0026689502 |
| [TQIQTRRSFE](https://webs.iiitd.edu.in/raghava/cellppd/pepsearch1.php?seq=TQIQTRRSFE&thval=0.0) | CPP | High | 0.537722 |
| NEP |  |  |  |
| [FQDILLRMSK](https://webs.iiitd.edu.in/raghava/cellppd/pepsearch1.php?seq=FQDILLRMSK&thval=0.0) | CPP | Low | 0.019369137 |
| [LQNRNEKWRE](https://webs.iiitd.edu.in/raghava/cellppd/pepsearch1.php?seq=LQNRNEKWRE&thval=0.0) | CPP | Low | 0.06303896 |

**Table S3. Physicochemical properties and CellPPD SVM scores of predicted cell-penetrating peptides (CPPs).**

| **The properties of peptides determined by CellPPD** | | | | | | | | | | | | |
| --- | --- | --- | --- | --- | --- | --- | --- | --- | --- | --- | --- | --- |
| [**Peptide Sequence**](https://webs.iiitd.edu.in/raghava/cellppd/prot_submitfreq.php?ran=31881) | [**SVM score**](https://webs.iiitd.edu.in/raghava/cellppd/prot_submitfreq.php?ran=31881) | [**Prediction**](https://webs.iiitd.edu.in/raghava/cellppd/prot_submitfreq.php?ran=31881) | [**Hydrophobicity**](https://webs.iiitd.edu.in/raghava/cellppd/prot_submitfreq.php?ran=31881) | [**Steric hindrance**](https://webs.iiitd.edu.in/raghava/cellppd/prot_submitfreq.php?ran=31881) | [**Sidebulk**](https://webs.iiitd.edu.in/raghava/cellppd/prot_submitfreq.php?ran=31881) | [**Hydropathicity**](https://webs.iiitd.edu.in/raghava/cellppd/prot_submitfreq.php?ran=31881) | [**Amphipathicity**](https://webs.iiitd.edu.in/raghava/cellppd/prot_submitfreq.php?ran=31881) | [**Hydrophilicity**](https://webs.iiitd.edu.in/raghava/cellppd/prot_submitfreq.php?ran=31881) | [**Net Hydrogen**](https://webs.iiitd.edu.in/raghava/cellppd/prot_submitfreq.php?ran=31881) | [**Charge**](https://webs.iiitd.edu.in/raghava/cellppd/prot_submitfreq.php?ran=31881) | [**pI**](https://webs.iiitd.edu.in/raghava/cellppd/prot_submitfreq.php?ran=31881) | [**Mol wt**](https://webs.iiitd.edu.in/raghava/cellppd/prot_submitfreq.php?ran=31881) |
| **NP** |  |  |  |  |  |  |  |  |  |  |  |  |
| RSRYWAIRTR | 0.32 | CPP | -0.61 | 0.62 | 0.62 | -1.54 | 0.98 | 0.39 | 2.00 | 4.00 | 12.01 | 1364.70 |
| RMIKRGINDR | 0.24 | CPP | -0.59 | 0.71 | 0.71 | -1.39 | 1.10 | 1.03 | 1.70 | 3.00 | 11.72 | 1258.65 |
| GPIYRRVNGK | 0.22 | CPP | -0.37 | 0.66 | 0.66 | -1.14 | 0.86 | 0.36 | 1.30 | 3.00 | 11.01 | 1159.50 |
| KGTKVVPRGK | 0.20 | CPP | -0.39 | 0.64 | 0.64 | -1.09 | 1.35 | 0.86 | 1.10 | 4.00 | 11.27 | 1069.46 |
| RRIWRQANNG | 0.19 | CPP | -0.57 | 0.66 | 0.66 | -1.90 | 0.86 | 0.39 | 1.90 | 3.00 | 12.31 | 1270.56 |
| YQRTRALVRT | 0.19 | CPP | -0.50 | 0.62 | 0.62 | -0.99 | 0.86 | 0.23 | 1.70 | 3.00 | 11.72 | 1263.60 |
| RYWAIRTRSG | 0.19 | CPP | -0.42 | 0.62 | 0.62 | -1.13 | 0.74 | 0.09 | 1.60 | 3.00 | 11.72 | 1265.57 |
| YRRVNGKWMR | 0.17 | CPP | -0.57 | 0.68 | 0.68 | -1.74 | 1.10 | 0.37 | 1.80 | 4.00 | 11.73 | 1365.76 |
| GRKTRIAYER | 0.15 | CPP | -0.60 | 0.65 | 0.65 | -1.70 | 1.23 | 1.00 | 1.70 | 3.00 | 10.91 | 1249.57 |
| RTRALVRTGM | 0.14 | CPP | -0.39 | 0.63 | 0.63 | -0.36 | 0.74 | 0.31 | 1.40 | 3.00 | 12.31 | 1160.54 |
| SRYWAIRTRS | 0.13 | CPP | -0.46 | 0.60 | 0.60 | -1.17 | 0.74 | 0.12 | 1.70 | 3.00 | 11.72 | 1295.59 |
| HPSAGKDPKK | 0.12 | CPP | -0.44 | 0.53 | 0.53 | -2.10 | 1.25 | 1.13 | 0.90 | 2.50 | 9.72 | 1064.34 |
| KEEIRRIWRQ | 0.12 | CPP | -0.65 | 0.67 | 0.67 | -1.98 | 1.48 | 1.12 | 1.90 | 2.00 | 10.75 | 1413.79 |
| EIRRIWRQAN | 0.11 | CPP | -0.51 | 0.66 | 0.66 | -1.41 | 0.99 | 0.49 | 1.80 | 2.00 | 11.70 | 1341.68 |
| RGENGRKTRI | 0.09 | CPP | -0.68 | 0.68 | 0.68 | -2.14 | 1.23 | 1.30 | 1.80 | 3.00 | 11.72 | 1186.48 |
| EEIRRIWRQA | 0.08 | CPP | -0.51 | 0.65 | 0.65 | -1.41 | 1.11 | 0.77 | 1.70 | 1.00 | 9.86 | 1356.69 |
| FLARSALILR | 0.08 | CPP | -0.03 | 0.59 | 0.59 | 1.25 | 0.49 | -0.44 | 0.90 | 2.00 | 12.01 | 1159.58 |
| ESSTLELRSR | 0.08 | CPP | -0.47 | 0.59 | 0.59 | -1.15 | 0.74 | 0.89 | 1.40 | 0.00 | 6.50 | 1177.41 |
| IRRIWRQANN | 0.06 | CPP | -0.52 | 0.67 | 0.67 | -1.41 | 0.86 | 0.21 | 1.90 | 3.00 | 12.31 | 1326.67 |
| RNFWRGENGR | 0.04 | CPP | -0.59 | 0.68 | 0.68 | -2.29 | 0.86 | 0.65 | 1.80 | 2.00 | 11.70 | 1291.53 |
| RKTRIAYERM | 0.04 | CPP | -0.59 | 0.66 | 0.66 | -1.47 | 1.23 | 0.87 | 1.70 | 3.00 | 10.91 | 1323.71 |
| LSAFDERRNK | 0.03 | CPP | -0.55 | 0.65 | 0.65 | -1.58 | 0.98 | 1.07 | 1.50 | 1.00 | 9.10 | 1235.49 |
| GKWMRELILY | 0.03 | CPP | -0.09 | 0.65 | 0.65 | -0.05 | 0.74 | -0.34 | 0.90 | 1.00 | 8.93 | 1308.76 |
| TYQRTRALVR | 0.03 | CPP | -0.50 | 0.62 | 0.62 | -0.99 | 0.86 | 0.23 | 1.70 | 3.00 | 11.72 | 1263.60 |
| RGINDRNFWR | 0.03 | CPP | -0.54 | 0.69 | 0.69 | -1.80 | 0.74 | 0.47 | 1.80 | 2.00 | 11.70 | 1333.61 |
| WRGENGRKTR | 0.03 | CPP | -0.71 | 0.66 | 0.66 | -2.68 | 1.23 | 1.14 | 1.90 | 3.00 | 11.72 | 1259.53 |
| [**Peptide Sequence**](https://webs.iiitd.edu.in/raghava/cellppd/prot_submitfreq.php?ran=31881) | [**SVM score**](https://webs.iiitd.edu.in/raghava/cellppd/prot_submitfreq.php?ran=31881) | [**Prediction**](https://webs.iiitd.edu.in/raghava/cellppd/prot_submitfreq.php?ran=31881) | [**Hydrophobicity**](https://webs.iiitd.edu.in/raghava/cellppd/prot_submitfreq.php?ran=31881) | [**Steric hindrance**](https://webs.iiitd.edu.in/raghava/cellppd/prot_submitfreq.php?ran=31881) | [**Sidebulk**](https://webs.iiitd.edu.in/raghava/cellppd/prot_submitfreq.php?ran=31881) | [**Hydropathicity**](https://webs.iiitd.edu.in/raghava/cellppd/prot_submitfreq.php?ran=31881) | [**Amphipathicity**](https://webs.iiitd.edu.in/raghava/cellppd/prot_submitfreq.php?ran=31881) | [**Hydrophilicity**](https://webs.iiitd.edu.in/raghava/cellppd/prot_submitfreq.php?ran=31881) | [**Net Hydrogen**](https://webs.iiitd.edu.in/raghava/cellppd/prot_submitfreq.php?ran=31881) | [**Charge**](https://webs.iiitd.edu.in/raghava/cellppd/prot_submitfreq.php?ran=31881) | [**pI**](https://webs.iiitd.edu.in/raghava/cellppd/prot_submitfreq.php?ran=31881) | [**Mol wt**](https://webs.iiitd.edu.in/raghava/cellppd/prot_submitfreq.php?ran=31881) |
| PSAGKDPKKT | 0.02 | CPP | -0.42 | 0.58 | 0.58 | -1.85 | 1.10 | 1.14 | 0.90 | 2.00 | 9.72 | 1028.30 |
| **HA** |  |  |  |  |  |  |  |  |  |  |  |  |
| [KNSYVNKKGK](https://webs.iiitd.edu.in/raghava/cellppd/pepsearch1.php?seq=KNSYVNKKGK&thval=0.0) | 0.22 | CPP | -0.52 | 0.68 | 0.68 | -2.09 | 1.47 | 0.89 | 1.40 | 4.00 | 10.18 | 1165.50 |
| [KLCRLKGIAP](https://webs.iiitd.edu.in/raghava/cellppd/pepsearch1.php?seq=KLCRLKGIAP&thval=0.0) | 0.17 | CPP | -0.18 | 0.60 | 0.60 | 0.21 | 0.98 | 0.21 | 0.80 | 3.00 | 10.07 | 1098.56 |
| [PEIAERPKVR](https://webs.iiitd.edu.in/raghava/cellppd/pepsearch1.php?seq=PEIAERPKVR&thval=0.0) | 0.11 | CPP | -0.45 | 0.60 | 0.60 | -1.26 | 1.11 | 1.12 | 1.20 | 1.00 | 9.10 | 1194.53 |
| [KLKNSYVNKK](https://webs.iiitd.edu.in/raghava/cellppd/pepsearch1.php?seq=KLKNSYVNKK&thval=0.0) | 0.10 | CPP | -0.49 | 0.67 | 0.67 | -1.67 | 1.47 | 0.71 | 1.40 | 4.00 | 10.18 | 1221.61 |
| [PKYVRSAKLR](https://webs.iiitd.edu.in/raghava/cellppd/pepsearch1.php?seq=PKYVRSAKLR&thval=0.0) | 0.10 | CPP | -0.47 | 0.61 | 0.61 | -1.07 | 1.22 | 0.62 | 1.40 | 4.00 | 11.10 | 1217.61 |
| [KYVRSAKLRM](https://webs.iiitd.edu.in/raghava/cellppd/pepsearch1.php?seq=KYVRSAKLRM&thval=0.0) | 0.10 | CPP | -0.44 | 0.65 | 0.65 | -0.72 | 1.22 | 0.49 | 1.40 | 4.00 | 11.10 | 1251.69 |
| [MNYYWTLLKP](https://webs.iiitd.edu.in/raghava/cellppd/pepsearch1.php?seq=MNYYWTLLKP&thval=0.0) | 0.07 | CPP | -0.03 | 0.61 | 0.61 | -0.37 | 0.37 | -1.01 | 0.80 | 1.00 | 8.83 | 1328.74 |
| [HNGKLCRLKG](https://webs.iiitd.edu.in/raghava/cellppd/pepsearch1.php?seq=HNGKLCRLKG&thval=0.0) | 0.04 | CPP | -0.36 | 0.58 | 0.58 | -0.97 | 1.12 | 0.41 | 1.10 | 3.50 | 10.07 | 1125.51 |
| [GKLCRLKGIA](https://webs.iiitd.edu.in/raghava/cellppd/pepsearch1.php?seq=GKLCRLKGIA&thval=0.0) | 0.04 | CPP | -0.16 | 0.63 | 0.63 | 0.33 | 0.98 | 0.21 | 0.80 | 3.00 | 10.07 | 1058.50 |
| [GKEFNKLEKR](https://webs.iiitd.edu.in/raghava/cellppd/pepsearch1.php?seq=GKEFNKLEKR&thval=0.0) | 0.02 | CPP | -0.56 | 0.67 | 0.67 | -2.05 | 1.60 | 1.39 | 1.40 | 2.00 | 9.72 | 1248.59 |
| [RRFTPEIAER](https://webs.iiitd.edu.in/raghava/cellppd/pepsearch1.php?seq=RRFTPEIAER&thval=0.0) | 0.00 | CPP | -0.52 | 0.62 | 0.62 | -1.37 | 0.99 | 0.98 | 1.50 | 1.00 | 9.86 | 1274.57 |
| **NS1** |  |  |  |  |  |  |  |  |  |  |  |  |
| [RLRRDQKSLR](https://webs.iiitd.edu.in/raghava/cellppd/pepsearch1.php?seq=RLRRDQKSLR&thval=0.0) | 0.57 | CPP | -0.88 | 0.64 | 0.64 | -2.21 | 1.47 | 1.49 | 2.20 | 4.00 | 12.01 | 1327.69 |
| [RRDQKSLRGR](https://webs.iiitd.edu.in/raghava/cellppd/pepsearch1.php?seq=RRDQKSLRGR&thval=0.0) | 0.44 | CPP | -0.91 | 0.66 | 0.66 | -2.63 | 1.47 | 1.67 | 2.20 | 4.00 | 12.01 | 1271.58 |
| [FLDRLRRDQK](https://webs.iiitd.edu.in/raghava/cellppd/pepsearch1.php?seq=FLDRLRRDQK&thval=0.0) | 0.32 | CPP | -0.68 | 0.67 | 0.67 | -1.75 | 1.23 | 1.21 | 1.80 | 2.00 | 10.75 | 1346.69 |
| [RPPLTPKQKR](https://webs.iiitd.edu.in/raghava/cellppd/pepsearch1.php?seq=RPPLTPKQKR&thval=0.0) | 0.29 | CPP | -0.63 | 0.55 | 0.55 | -2.20 | 1.35 | 1.00 | 1.50 | 4.00 | 12.02 | 1220.62 |
| [DAPFLDRLRR](https://webs.iiitd.edu.in/raghava/cellppd/pepsearch1.php?seq=DAPFLDRLRR&thval=0.0) | 0.27 | CPP | -0.49 | 0.62 | 0.62 | -0.99 | 0.74 | 0.84 | 1.40 | 1.00 | 9.86 | 1258.57 |
| [DRLRRDQKSL](https://webs.iiitd.edu.in/raghava/cellppd/pepsearch1.php?seq=DRLRRDQKSL&thval=0.0) | 0.13 | CPP | -0.77 | 0.65 | 0.65 | -2.11 | 1.23 | 1.49 | 1.90 | 2.00 | 10.75 | 1286.59 |
| [FLWHVRKRVA](https://webs.iiitd.edu.in/raghava/cellppd/pepsearch1.php?seq=FLWHVRKRVA&thval=0.0) | 0.12 | CPP | -0.22 | 0.57 | 0.57 | -0.02 | 1.00 | -0.27 | 1.20 | 3.50 | 12.01 | 1311.74 |
| [DCFLWHVRKR](https://webs.iiitd.edu.in/raghava/cellppd/pepsearch1.php?seq=DCFLWHVRKR&thval=0.0) | 0.11 | CPP | -0.37 | 0.58 | 0.58 | -0.72 | 1.00 | 0.13 | 1.30 | 2.50 | 9.55 | 1359.75 |
| [APFLDRLRRD](https://webs.iiitd.edu.in/raghava/cellppd/pepsearch1.php?seq=APFLDRLRRD&thval=0.0) | 0.05 | CPP | -0.49 | 0.62 | 0.62 | -0.99 | 0.74 | 0.84 | 1.40 | 1.00 | 9.86 | 1258.57 |
| [EWSMLIPKQK](https://webs.iiitd.edu.in/raghava/cellppd/pepsearch1.php?seq=EWSMLIPKQK&thval=0.0) | 0.05 | CPP | -0.20 | 0.61 | 0.61 | -0.79 | 0.99 | 0.12 | 0.90 | 1.00 | 8.94 | 1259.68 |
| [LQRFAWRSSN](https://webs.iiitd.edu.in/raghava/cellppd/pepsearch1.php?seq=LQRFAWRSSN&thval=0.0) | 0.03 | CPP | -0.36 | 0.61 | 0.61 | -1.01 | 0.61 | -0.12 | 1.50 | 2.00 | 12.01 | 1264.54 |
| [GKQIVERILK](https://webs.iiitd.edu.in/raghava/cellppd/pepsearch1.php?seq=GKQIVERILK&thval=0.0) | 0.01 | CPP | -0.26 | 0.67 | 0.67 | -0.27 | 1.23 | 0.53 | 1.10 | 2.00 | 10.01 | 1183.62 |
| [CFLWHVRKRV](https://webs.iiitd.edu.in/raghava/cellppd/pepsearch1.php?seq=CFLWHVRKRV&thval=0.0) | 0.00 | CPP | -0.24 | 0.58 | 0.58 | 0.05 | 1.00 | -0.32 | 1.20 | 3.50 | 10.87 | 1343.80 |
| **PB2** |  |  |  |  |  |  |  |  |  |  |  |  |
| [RRATAILRKA](https://webs.iiitd.edu.in/raghava/cellppd/pepsearch1.php?seq=RRATAILRKA&thval=0.0) | 0.41 | CPP | -0.46 | 0.60 | 0.60 | -0.44 | 1.10 | 0.65 | 1.50 | 4.00 | 12.31 | 1155.54 |
| [GRRATAILRK](https://webs.iiitd.edu.in/raghava/cellppd/pepsearch1.php?seq=GRRATAILRK&thval=0.0) | 0.39 | CPP | -0.46 | 0.62 | 0.62 | -0.66 | 1.10 | 0.70 | 1.50 | 4.00 | 12.31 | 1141.52 |
| [**Peptide Sequence**](https://webs.iiitd.edu.in/raghava/cellppd/prot_submitfreq.php?ran=31881) | [**SVM score**](https://webs.iiitd.edu.in/raghava/cellppd/prot_submitfreq.php?ran=31881) | [**Prediction**](https://webs.iiitd.edu.in/raghava/cellppd/prot_submitfreq.php?ran=31881) | [**Hydrophobicity**](https://webs.iiitd.edu.in/raghava/cellppd/prot_submitfreq.php?ran=31881) | [**Steric hindrance**](https://webs.iiitd.edu.in/raghava/cellppd/prot_submitfreq.php?ran=31881) | [**Sidebulk**](https://webs.iiitd.edu.in/raghava/cellppd/prot_submitfreq.php?ran=31881) | [**Hydropathicity**](https://webs.iiitd.edu.in/raghava/cellppd/prot_submitfreq.php?ran=31881) | [**Amphipathicity**](https://webs.iiitd.edu.in/raghava/cellppd/prot_submitfreq.php?ran=31881) | [**Hydrophilicity**](https://webs.iiitd.edu.in/raghava/cellppd/prot_submitfreq.php?ran=31881) | [**Net Hydrogen**](https://webs.iiitd.edu.in/raghava/cellppd/prot_submitfreq.php?ran=31881) | [**Charge**](https://webs.iiitd.edu.in/raghava/cellppd/prot_submitfreq.php?ran=31881) | [**pI**](https://webs.iiitd.edu.in/raghava/cellppd/prot_submitfreq.php?ran=31881) | [**Mol wt**](https://webs.iiitd.edu.in/raghava/cellppd/prot_submitfreq.php?ran=31881) |
| [RKATRRLIQL](https://webs.iiitd.edu.in/raghava/cellppd/pepsearch1.php?seq=RKATRRLIQL&thval=0.0) | 0.39 | CPP | -0.52 | 0.62 | 0.62 | -0.77 | 1.23 | 0.59 | 1.70 | 4.00 | 12.31 | 1254.69 |
| [TAILRKATRR](https://webs.iiitd.edu.in/raghava/cellppd/pepsearch1.php?seq=TAILRKATRR&thval=0.0) | 0.31 | CPP | -0.50 | 0.60 | 0.60 | -0.69 | 1.10 | 0.66 | 1.60 | 4.00 | 12.31 | 1185.57 |
| [KNPALRMKWM](https://webs.iiitd.edu.in/raghava/cellppd/pepsearch1.php?seq=KNPALRMKWM&thval=0.0) | 0.28 | CPP | -0.30 | 0.63 | 0.63 | -0.89 | 0.98 | 0.09 | 1.10 | 3.00 | 11.17 | 1274.75 |
| [RATAILRKAT](https://webs.iiitd.edu.in/raghava/cellppd/pepsearch1.php?seq=RATAILRKAT&thval=0.0) | 0.26 | CPP | -0.30 | 0.59 | 0.59 | -0.06 | 0.86 | 0.31 | 1.20 | 3.00 | 12.01 | 1100.46 |
| [ILRKATRRLI](https://webs.iiitd.edu.in/raghava/cellppd/pepsearch1.php?seq=ILRKATRRLI&thval=0.0) | 0.25 | CPP | -0.38 | 0.62 | 0.62 | 0.03 | 1.10 | 0.39 | 1.50 | 4.00 | 12.31 | 1239.72 |
| [AILRKATRRL](https://webs.iiitd.edu.in/raghava/cellppd/pepsearch1.php?seq=AILRKATRRL&thval=0.0) | 0.23 | CPP | -0.43 | 0.60 | 0.60 | -0.24 | 1.10 | 0.52 | 1.50 | 4.00 | 12.31 | 1197.63 |
| [SQTATKRIRM](https://webs.iiitd.edu.in/raghava/cellppd/pepsearch1.php?seq=SQTATKRIRM&thval=0.0) | 0.21 | CPP | -0.47 | 0.63 | 0.63 | -1.04 | 0.98 | 0.51 | 1.50 | 3.00 | 12.01 | 1191.55 |
| [ATAILRKATR](https://webs.iiitd.edu.in/raghava/cellppd/pepsearch1.php?seq=ATAILRKATR&thval=0.0) | 0.17 | CPP | -0.30 | 0.59 | 0.59 | -0.06 | 0.86 | 0.31 | 1.20 | 3.00 | 12.01 | 1100.46 |
| [KATRRLIQLI](https://webs.iiitd.edu.in/raghava/cellppd/pepsearch1.php?seq=KATRRLIQLI&thval=0.0) | 0.15 | CPP | -0.27 | 0.62 | 0.62 | 0.13 | 0.98 | 0.11 | 1.30 | 3.00 | 12.01 | 1211.67 |
| [RANQRLNPMH](https://webs.iiitd.edu.in/raghava/cellppd/pepsearch1.php?seq=RANQRLNPMH&thval=0.0) | 0.15 | CPP | -0.49 | 0.58 | 0.58 | -1.68 | 0.76 | 0.25 | 1.50 | 2.50 | 12.01 | 1236.56 |
| [QSRTREILTK](https://webs.iiitd.edu.in/raghava/cellppd/pepsearch1.php?seq=QSRTREILTK&thval=0.0) | 0.13 | CPP | -0.53 | 0.62 | 0.62 | -1.38 | 1.11 | 0.81 | 1.60 | 2.00 | 10.84 | 1231.56 |
| [DRFLRVRDQR](https://webs.iiitd.edu.in/raghava/cellppd/pepsearch1.php?seq=DRFLRVRDQR&thval=0.0) | 0.13 | CPP | -0.75 | 0.68 | 0.68 | -1.77 | 1.10 | 1.24 | 2.00 | 2.00 | 11.53 | 1360.67 |
| [LRKATRRLIQ](https://webs.iiitd.edu.in/raghava/cellppd/pepsearch1.php?seq=LRKATRRLIQ&thval=0.0) | 0.12 | CPP | -0.52 | 0.62 | 0.62 | -0.77 | 1.23 | 0.59 | 1.70 | 4.00 | 12.31 | 1254.69 |
| [RLIQLIVSGR](https://webs.iiitd.edu.in/raghava/cellppd/pepsearch1.php?seq=RLIQLIVSGR&thval=0.0) | 0.12 | CPP | -0.12 | 0.64 | 0.64 | 0.71 | 0.61 | -0.22 | 1.10 | 2.00 | 12.01 | 1154.58 |
| [AIIKKYTSGR](https://webs.iiitd.edu.in/raghava/cellppd/pepsearch1.php?seq=AIIKKYTSGR&thval=0.0) | 0.10 | CPP | -0.25 | 0.64 | 0.64 | -0.47 | 0.98 | 0.25 | 1.10 | 3.00 | 10.30 | 1136.50 |
| [VGRRATAILR](https://webs.iiitd.edu.in/raghava/cellppd/pepsearch1.php?seq=VGRRATAILR&thval=0.0) | 0.10 | CPP | -0.30 | 0.62 | 0.62 | 0.15 | 0.74 | 0.25 | 1.30 | 3.00 | 12.31 | 1112.48 |
| [VNRANQRLNP](https://webs.iiitd.edu.in/raghava/cellppd/pepsearch1.php?seq=VNRANQRLNP&thval=0.0) | 0.10 | CPP | -0.49 | 0.64 | 0.64 | -1.48 | 0.61 | 0.30 | 1.60 | 2.00 | 12.01 | 1181.46 |
| [KKYTSGRQEK](https://webs.iiitd.edu.in/raghava/cellppd/pepsearch1.php?seq=KKYTSGRQEK&thval=0.0) | 0.09 | CPP | -0.66 | 0.65 | 0.65 | -2.64 | 1.60 | 1.28 | 1.60 | 3.00 | 10.01 | 1224.52 |
| [SQLTITKEKK](https://webs.iiitd.edu.in/raghava/cellppd/pepsearch1.php?seq=SQLTITKEKK&thval=0.0) | 0.09 | CPP | -0.40 | 0.62 | 0.62 | -1.26 | 1.35 | 0.81 | 1.20 | 2.00 | 9.72 | 1175.54 |
| [VRKTRFLPVA](https://webs.iiitd.edu.in/raghava/cellppd/pepsearch1.php?seq=VRKTRFLPVA&thval=0.0) | 0.09 | CPP | -0.24 | 0.61 | 0.61 | 0.16 | 0.86 | 0.08 | 1.10 | 3.00 | 12.01 | 1186.60 |
| [DSQTATKRIR](https://webs.iiitd.edu.in/raghava/cellppd/pepsearch1.php?seq=DSQTATKRIR&thval=0.0) | 0.09 | CPP | -0.57 | 0.63 | 0.63 | -1.58 | 0.98 | 0.94 | 1.60 | 2.00 | 10.84 | 1175.44 |
| [LIIAARNIVR](https://webs.iiitd.edu.in/raghava/cellppd/pepsearch1.php?seq=LIIAARNIVR&thval=0.0) | 0.08 | CPP | -0.04 | 0.65 | 0.65 | 1.26 | 0.49 | -0.35 | 1.00 | 2.00 | 12.01 | 1138.57 |
| [FAAAPPKQSR](https://webs.iiitd.edu.in/raghava/cellppd/pepsearch1.php?seq=FAAAPPKQSR&thval=0.0) | 0.08 | CPP | -0.26 | 0.55 | 0.55 | -0.77 | 0.74 | 0.25 | 0.90 | 2.00 | 11.01 | 1072.35 |
| [KRITEMIPER](https://webs.iiitd.edu.in/raghava/cellppd/pepsearch1.php?seq=KRITEMIPER&thval=0.0) | 0.07 | CPP | -0.44 | 0.65 | 0.65 | -1.13 | 1.11 | 0.97 | 1.30 | 1.00 | 9.10 | 1272.67 |
| [KRTSGSSVKR](https://webs.iiitd.edu.in/raghava/cellppd/pepsearch1.php?seq=KRTSGSSVKR&thval=0.0) | 0.07 | CPP | -0.60 | 0.62 | 0.62 | -1.61 | 1.22 | 1.10 | 1.60 | 4.00 | 12.02 | 1105.39 |
| [VSIDRFLRVR](https://webs.iiitd.edu.in/raghava/cellppd/pepsearch1.php?seq=VSIDRFLRVR&thval=0.0) | 0.07 | CPP | -0.33 | 0.67 | 0.67 | 0.17 | 0.74 | 0.32 | 1.40 | 2.00 | 11.70 | 1260.64 |
| [QIIKLLPFAA](https://webs.iiitd.edu.in/raghava/cellppd/pepsearch1.php?seq=QIIKLLPFAA&thval=0.0) | 0.07 | CPP | 0.18 | 0.59 | 0.59 | 1.40 | 0.49 | -0.75 | 0.40 | 1.00 | 9.11 | 1113.56 |
| [TATKRIRMAI](https://webs.iiitd.edu.in/raghava/cellppd/pepsearch1.php?seq=TATKRIRMAI&thval=0.0) | 0.07 | CPP | -0.28 | 0.63 | 0.63 | 0.02 | 0.86 | 0.23 | 1.20 | 3.00 | 12.01 | 1160.58 |
| [MSMRGVRISK](https://webs.iiitd.edu.in/raghava/cellppd/pepsearch1.php?seq=MSMRGVRISK&thval=0.0) | 0.06 | CPP | -0.32 | 0.67 | 0.67 | -0.24 | 0.86 | 0.37 | 1.20 | 3.00 | 12.01 | 1164.59 |
| [NKATKRLTVL](https://webs.iiitd.edu.in/raghava/cellppd/pepsearch1.php?seq=NKATKRLTVL&thval=0.0) | 0.06 | CPP | -0.31 | 0.61 | 0.61 | -0.36 | 0.98 | 0.28 | 1.20 | 3.00 | 11.17 | 1143.54 |
| [**Peptide Sequence**](https://webs.iiitd.edu.in/raghava/cellppd/prot_submitfreq.php?ran=31881) | [**SVM score**](https://webs.iiitd.edu.in/raghava/cellppd/prot_submitfreq.php?ran=31881) | [**Prediction**](https://webs.iiitd.edu.in/raghava/cellppd/prot_submitfreq.php?ran=31881) | [**Hydrophobicity**](https://webs.iiitd.edu.in/raghava/cellppd/prot_submitfreq.php?ran=31881) | [**Steric hindrance**](https://webs.iiitd.edu.in/raghava/cellppd/prot_submitfreq.php?ran=31881) | [**Sidebulk**](https://webs.iiitd.edu.in/raghava/cellppd/prot_submitfreq.php?ran=31881) | [**Hydropathicity**](https://webs.iiitd.edu.in/raghava/cellppd/prot_submitfreq.php?ran=31881) | [**Amphipathicity**](https://webs.iiitd.edu.in/raghava/cellppd/prot_submitfreq.php?ran=31881) | [**Hydrophilicity**](https://webs.iiitd.edu.in/raghava/cellppd/prot_submitfreq.php?ran=31881) | [**Net Hydrogen**](https://webs.iiitd.edu.in/raghava/cellppd/prot_submitfreq.php?ran=31881) | [**Charge**](https://webs.iiitd.edu.in/raghava/cellppd/prot_submitfreq.php?ran=31881) | [**pI**](https://webs.iiitd.edu.in/raghava/cellppd/prot_submitfreq.php?ran=31881) | [**Mol wt**](https://webs.iiitd.edu.in/raghava/cellppd/prot_submitfreq.php?ran=31881) |
| [RNLMSQSRTR](https://webs.iiitd.edu.in/raghava/cellppd/pepsearch1.php?seq=RNLMSQSRTR&thval=0.0) | 0.05 | CPP | -0.65 | 0.64 | 0.64 | -1.71 | 0.86 | 0.65 | 1.90 | 3.00 | 12.31 | 1248.56 |
| [AQIIKLLPFA](https://webs.iiitd.edu.in/raghava/cellppd/pepsearch1.php?seq=AQIIKLLPFA&thval=0.0) | 0.04 | CPP | 0.18 | 0.59 | 0.59 | 1.40 | 0.49 | -0.75 | 0.40 | 1.00 | 9.11 | 1113.56 |
| [MHQLLRHFQK](https://webs.iiitd.edu.in/raghava/cellppd/pepsearch1.php?seq=MHQLLRHFQK&thval=0.0) | 0.03 | CPP | -0.31 | 0.53 | 0.53 | -0.95 | 1.15 | -0.20 | 1.20 | 3.00 | 11.01 | 1337.77 |
| [KATKRLTVLG](https://webs.iiitd.edu.in/raghava/cellppd/pepsearch1.php?seq=KATKRLTVLG&thval=0.0) | 0.03 | CPP | -0.23 | 0.61 | 0.61 | -0.05 | 0.98 | 0.26 | 1.00 | 3.00 | 11.17 | 1086.49 |
| [MKRKRDSSIL](https://webs.iiitd.edu.in/raghava/cellppd/pepsearch1.php?seq=MKRKRDSSIL&thval=0.0) | 0.03 | CPP | -0.54 | 0.66 | 0.66 | -1.17 | 1.22 | 1.07 | 1.50 | 3.00 | 11.01 | 1233.63 |
| [LVRKTRFLPV](https://webs.iiitd.edu.in/raghava/cellppd/pepsearch1.php?seq=LVRKTRFLPV&thval=0.0) | 0.02 | CPP | -0.21 | 0.61 | 0.61 | 0.36 | 0.86 | -0.05 | 1.10 | 3.00 | 12.01 | 1228.69 |
| **M2** |  |  |  |  |  |  |  |  |  |  |  |  |
| [KCIYRRFKYG](https://webs.iiitd.edu.in/raghava/cellppd/pepsearch1.php?seq=KCIYRRFKYG&thval=0.0) | 0.36 | CPP | -0.41 | 0.68 | 0.68 | -1.00 | 1.22 | 0.21 | 1.40 | 4.00 | 10.04 | 1333.75 |
| [RLFFKCIYRR](https://webs.iiitd.edu.in/raghava/cellppd/pepsearch1.php?seq=RLFFKCIYRR&thval=0.0) | 0.19 | CPP | -0.38 | 0.67 | 0.67 | -0.23 | 1.10 | 0.01 | 1.50 | 4.00 | 10.92 | 1401.87 |
| [IYRRFKYGLK](https://webs.iiitd.edu.in/raghava/cellppd/pepsearch1.php?seq=IYRRFKYGLK&thval=0.0) | 0.14 | CPP | -0.36 | 0.67 | 0.67 | -0.87 | 1.22 | 0.13 | 1.40 | 4.00 | 10.45 | 1343.78 |
| [YRRFKYGLKG](https://webs.iiitd.edu.in/raghava/cellppd/pepsearch1.php?seq=YRRFKYGLKG&thval=0.0) | 0.11 | CPP | -0.42 | 0.67 | 0.67 | -1.36 | 1.22 | 0.31 | 1.40 | 4.00 | 10.45 | 1287.67 |
| [FFKCIYRRFK](https://webs.iiitd.edu.in/raghava/cellppd/pepsearch1.php?seq=FFKCIYRRFK&thval=0.0) | 0.10 | CPP | -0.31 | 0.68 | 0.68 | -0.27 | 1.22 | -0.06 | 1.30 | 4.00 | 10.32 | 1407.87 |
| [FKCIYRRFKY](https://webs.iiitd.edu.in/raghava/cellppd/pepsearch1.php?seq=FKCIYRRFKY&thval=0.0) | 0.10 | CPP | -0.37 | 0.68 | 0.68 | -0.68 | 1.22 | -0.04 | 1.40 | 4.00 | 10.04 | 1423.87 |
| [CIYRRFKYGL](https://webs.iiitd.edu.in/raghava/cellppd/pepsearch1.php?seq=CIYRRFKYGL&thval=0.0) | 0.10 | CPP | -0.25 | 0.67 | 0.67 | -0.23 | 0.86 | -0.27 | 1.20 | 3.00 | 9.80 | 1318.74 |
| [WILDRLFFKC](https://webs.iiitd.edu.in/raghava/cellppd/pepsearch1.php?seq=WILDRLFFKC&thval=0.0) | 0.07 | CPP | -0.02 | 0.64 | 0.64 | 0.74 | 0.61 | -0.58 | 0.80 | 1.00 | 8.57 | 1340.79 |
| [LWILDRLFFK](https://webs.iiitd.edu.in/raghava/cellppd/pepsearch1.php?seq=LWILDRLFFK&thval=0.0) | 0.06 | CPP | 0.03 | 0.63 | 0.63 | 0.87 | 0.61 | -0.66 | 0.80 | 1.00 | 9.10 | 1350.82 |
| **NA** |  |  |  |  |  |  |  |  |  |  |  |  |
| [IKSWRKKILR](https://webs.iiitd.edu.in/raghava/cellppd/pepsearch1.php?seq=IKSWRKKILR&thval=0.0) | 0.39 | CPP | -0.47 | 0.64 | 0.64 | -0.96 | 1.59 | 0.65 | 1.60 | 5.00 | 12.03 | 1327.83 |
| [ELIRGRPKEK](https://webs.iiitd.edu.in/raghava/cellppd/pepsearch1.php?seq=ELIRGRPKEK&thval=0.0) | 0.37 | CPP | -0.56 | 0.64 | 0.64 | -1.75 | 1.48 | 1.44 | 1.40 | 2.00 | 10.00 | 1225.60 |
| [YKIFKIEKGK](https://webs.iiitd.edu.in/raghava/cellppd/pepsearch1.php?seq=YKIFKIEKGK&thval=0.0) | 0.20 | CPP | -0.28 | 0.69 | 0.69 | -0.90 | 1.59 | 0.66 | 1.00 | 3.00 | 9.84 | 1253.70 |
| [ETIKSWRKKI](https://webs.iiitd.edu.in/raghava/cellppd/pepsearch1.php?seq=ETIKSWRKKI&thval=0.0) | 0.19 | CPP | -0.43 | 0.64 | 0.64 | -1.31 | 1.47 | 0.79 | 1.40 | 3.00 | 10.30 | 1288.70 |
| [TIKSWRKKIL](https://webs.iiitd.edu.in/raghava/cellppd/pepsearch1.php?seq=TIKSWRKKIL&thval=0.0) | 0.19 | CPP | -0.31 | 0.62 | 0.62 | -0.58 | 1.35 | 0.31 | 1.30 | 4.00 | 11.27 | 1272.75 |
| [KIFKIEKGKV](https://webs.iiitd.edu.in/raghava/cellppd/pepsearch1.php?seq=KIFKIEKGKV&thval=0.0) | 0.19 | CPP | -0.23 | 0.69 | 0.69 | -0.35 | 1.59 | 0.74 | 0.90 | 3.00 | 10.01 | 1189.66 |
| [LIRGRPKEKT](https://webs.iiitd.edu.in/raghava/cellppd/pepsearch1.php?seq=LIRGRPKEKT&thval=0.0) | 0.14 | CPP | -0.52 | 0.62 | 0.62 | -1.47 | 1.35 | 1.10 | 1.40 | 3.00 | 11.01 | 1197.59 |
| [TETIKSWRKK](https://webs.iiitd.edu.in/raghava/cellppd/pepsearch1.php?seq=TETIKSWRKK&thval=0.0) | 0.08 | CPP | -0.52 | 0.62 | 0.62 | -1.83 | 1.47 | 0.93 | 1.50 | 3.00 | 10.30 | 1276.64 |
| [KIEKGKVTKS](https://webs.iiitd.edu.in/raghava/cellppd/pepsearch1.php?seq=KIEKGKVTKS&thval=0.0) | 0.08 | CPP | -0.40 | 0.65 | 0.65 | -1.23 | 1.59 | 1.16 | 1.10 | 3.00 | 10.01 | 1117.50 |
| [GVWIGRTKSH](https://webs.iiitd.edu.in/raghava/cellppd/pepsearch1.php?seq=GVWIGRTKSH&thval=0.0) | 0.04 | CPP | -0.17 | 0.57 | 0.57 | -0.61 | 0.76 | -0.13 | 1.00 | 2.50 | 11.01 | 1140.46 |
| [FWVELIRGRP](https://webs.iiitd.edu.in/raghava/cellppd/pepsearch1.php?seq=FWVELIRGRP&thval=0.0) | 0.02 | CPP | -0.13 | 0.62 | 0.62 | -0.01 | 0.62 | -0.20 | 1.00 | 1.00 | 9.95 | 1272.66 |
| [PIRGWAIYSK](https://webs.iiitd.edu.in/raghava/cellppd/pepsearch1.php?seq=PIRGWAIYSK&thval=0.0) | 0.00 | CPP | -0.09 | 0.61 | 0.61 | -0.26 | 0.61 | -0.35 | 0.90 | 2.00 | 10.01 | 1190.55 |
| **PB1-F2** |  |  |  |  |  |  |  |  |  |  |  |  |
| [**Peptide Sequence**](https://webs.iiitd.edu.in/raghava/cellppd/prot_submitfreq.php?ran=31881) | [**SVM score**](https://webs.iiitd.edu.in/raghava/cellppd/prot_submitfreq.php?ran=31881) | [**Prediction**](https://webs.iiitd.edu.in/raghava/cellppd/prot_submitfreq.php?ran=31881) | [**Hydrophobicity**](https://webs.iiitd.edu.in/raghava/cellppd/prot_submitfreq.php?ran=31881) | [**Steric hindrance**](https://webs.iiitd.edu.in/raghava/cellppd/prot_submitfreq.php?ran=31881) | [**Sidebulk**](https://webs.iiitd.edu.in/raghava/cellppd/prot_submitfreq.php?ran=31881) | [**Hydropathicity**](https://webs.iiitd.edu.in/raghava/cellppd/prot_submitfreq.php?ran=31881) | [**Amphipathicity**](https://webs.iiitd.edu.in/raghava/cellppd/prot_submitfreq.php?ran=31881) | [**Hydrophilicity**](https://webs.iiitd.edu.in/raghava/cellppd/prot_submitfreq.php?ran=31881) | [**Net Hydrogen**](https://webs.iiitd.edu.in/raghava/cellppd/prot_submitfreq.php?ran=31881) | [**Charge**](https://webs.iiitd.edu.in/raghava/cellppd/prot_submitfreq.php?ran=31881) | [**pI**](https://webs.iiitd.edu.in/raghava/cellppd/prot_submitfreq.php?ran=31881) | [**Mol wt**](https://webs.iiitd.edu.in/raghava/cellppd/prot_submitfreq.php?ran=31881) |
| [KTRVLKRWRL](https://webs.iiitd.edu.in/raghava/cellppd/pepsearch1.php?seq=KTRVLKRWRL&thval=0.0) | 0.54 | CPP | -0.57 | 0.62 | 0.62 | -1.11 | 1.47 | 0.61 | 1.80 | 5.00 | 12.31 | 1355.84 |
| [LKRWRLFSKH](https://webs.iiitd.edu.in/raghava/cellppd/pepsearch1.php?seq=LKRWRLFSKH&thval=0.0) | 0.40 | CPP | -0.43 | 0.55 | 0.55 | -1.13 | 1.37 | 0.23 | 1.50 | 4.50 | 12.02 | 1370.81 |
| [VLKRWRLFSK](https://webs.iiitd.edu.in/raghava/cellppd/pepsearch1.php?seq=VLKRWRLFSK&thval=0.0) | 0.38 | CPP | -0.34 | 0.62 | 0.62 | -0.39 | 1.22 | 0.13 | 1.40 | 4.00 | 12.02 | 1332.80 |
| [LKTRVLKRWR](https://webs.iiitd.edu.in/raghava/cellppd/pepsearch1.php?seq=LKTRVLKRWR&thval=0.0) | 0.37 | CPP | -0.57 | 0.62 | 0.62 | -1.11 | 1.47 | 0.61 | 1.80 | 5.00 | 12.31 | 1355.84 |
| [FLKTRVLKRW](https://webs.iiitd.edu.in/raghava/cellppd/pepsearch1.php?seq=FLKTRVLKRW&thval=0.0) | 0.22 | CPP | -0.33 | 0.62 | 0.62 | -0.38 | 1.22 | 0.06 | 1.40 | 4.00 | 12.02 | 1346.83 |
| [RVLKRWRLFS](https://webs.iiitd.edu.in/raghava/cellppd/pepsearch1.php?seq=RVLKRWRLFS&thval=0.0) | 0.22 | CPP | -0.41 | 0.62 | 0.62 | -0.45 | 1.10 | 0.13 | 1.60 | 4.00 | 12.31 | 1360.81 |
| [KRWRLFSKHE](https://webs.iiitd.edu.in/raghava/cellppd/pepsearch1.php?seq=KRWRLFSKHE&thval=0.0) | 0.19 | CPP | -0.55 | 0.57 | 0.57 | -1.86 | 1.50 | 0.71 | 1.60 | 3.50 | 11.01 | 1386.76 |
| [WKQWLSLRNP](https://webs.iiitd.edu.in/raghava/cellppd/pepsearch1.php?seq=WKQWLSLRNP&thval=0.0) | 0.17 | CPP | -0.27 | 0.58 | 0.58 | -1.20 | 0.74 | -0.37 | 1.30 | 2.00 | 11.01 | 1327.70 |
| [TRVLKRWRLF](https://webs.iiitd.edu.in/raghava/cellppd/pepsearch1.php?seq=TRVLKRWRLF&thval=0.0) | 0.16 | CPP | -0.40 | 0.62 | 0.62 | -0.44 | 1.10 | 0.06 | 1.60 | 4.00 | 12.31 | 1374.84 |
| [KQWLSLRNPI](https://webs.iiitd.edu.in/raghava/cellppd/pepsearch1.php?seq=KQWLSLRNPI&thval=0.0) | 0.10 | CPP | -0.24 | 0.59 | 0.59 | -0.66 | 0.74 | -0.21 | 1.20 | 2.00 | 11.01 | 1254.65 |
| [RNSTRLMGHC](https://webs.iiitd.edu.in/raghava/cellppd/pepsearch1.php?seq=RNSTRLMGHC&thval=0.0) | 0.05 | CPP | -0.40 | 0.58 | 0.58 | -0.94 | 0.64 | 0.15 | 1.30 | 2.50 | 10.38 | 1174.50 |
| [GQQTPKLEHR](https://webs.iiitd.edu.in/raghava/cellppd/pepsearch1.php?seq=GQQTPKLEHR&thval=0.0) | 0.03 | CPP | -0.48 | 0.55 | 0.55 | -2.10 | 1.13 | 0.67 | 1.30 | 1.50 | 9.10 | 1193.48 |
| [KQIVYWKQWL](https://webs.iiitd.edu.in/raghava/cellppd/pepsearch1.php?seq=KQIVYWKQWL&thval=0.0) | 0.03 | CPP | -0.10 | 0.64 | 0.64 | -0.54 | 0.98 | -0.78 | 1.10 | 2.00 | 9.72 | 1391.84 |
| [HRNSTRLMGH](https://webs.iiitd.edu.in/raghava/cellppd/pepsearch1.php?seq=HRNSTRLMGH&thval=0.0) | 0.02 | CPP | -0.45 | 0.52 | 0.52 | -1.51 | 0.78 | 0.20 | 1.40 | 3.00 | 12.01 | 1208.51 |
| [VYWKQWLSLR](https://webs.iiitd.edu.in/raghava/cellppd/pepsearch1.php?seq=VYWKQWLSLR&thval=0.0) | 0.00 | CPP | -0.14 | 0.60 | 0.60 | -0.40 | 0.74 | -0.77 | 1.20 | 2.00 | 10.01 | 1378.79 |
| **PA** |  |  |  |  |  |  |  |  |  |  |  |  |
| [KIPKTKNMKK](https://webs.iiitd.edu.in/raghava/cellppd/pepsearch1.php?seq=KIPKTKNMKK&thval=0.0) | 0.61 | CPP | -0.54 | 0.65 | 0.65 | -1.89 | 1.84 | 1.17 | 1.30 | 5.00 | 10.61 | 1215.71 |
| [RIKTRLFTIR](https://webs.iiitd.edu.in/raghava/cellppd/pepsearch1.php?seq=RIKTRLFTIR&thval=0.0) | 0.43 | CPP | -0.41 | 0.64 | 0.64 | -0.32 | 1.10 | 0.33 | 1.60 | 4.00 | 12.31 | 1303.76 |
| [KTTPRPLRLP](https://webs.iiitd.edu.in/raghava/cellppd/pepsearch1.php?seq=KTTPRPLRLP&thval=0.0) | 0.39 | CPP | -0.41 | 0.52 | 0.52 | -1.15 | 0.86 | 0.46 | 1.20 | 3.00 | 12.01 | 1178.58 |
| [ESRARIKTRL](https://webs.iiitd.edu.in/raghava/cellppd/pepsearch1.php?seq=ESRARIKTRL&thval=0.0) | 0.35 | CPP | -0.59 | 0.62 | 0.62 | -1.23 | 1.23 | 1.08 | 1.70 | 3.00 | 11.72 | 1229.58 |
| [RARIKTRLFT](https://webs.iiitd.edu.in/raghava/cellppd/pepsearch1.php?seq=RARIKTRLFT&thval=0.0) | 0.33 | CPP | -0.46 | 0.62 | 0.62 | -0.59 | 1.10 | 0.46 | 1.60 | 4.00 | 12.31 | 1261.67 |
| [FLKTTPRPLR](https://webs.iiitd.edu.in/raghava/cellppd/pepsearch1.php?seq=FLKTTPRPLR&thval=0.0) | 0.33 | CPP | -0.34 | 0.56 | 0.56 | -0.71 | 0.86 | 0.21 | 1.20 | 3.00 | 12.01 | 1228.64 |
| [FIIKGRSHLR](https://webs.iiitd.edu.in/raghava/cellppd/pepsearch1.php?seq=FIIKGRSHLR&thval=0.0) | 0.30 | CPP | -0.25 | 0.59 | 0.59 | -0.17 | 1.00 | 0.09 | 1.20 | 3.50 | 12.01 | 1226.64 |
| [LKTTPRPLRL](https://webs.iiitd.edu.in/raghava/cellppd/pepsearch1.php?seq=LKTTPRPLRL&thval=0.0) | 0.27 | CPP | -0.35 | 0.54 | 0.54 | -0.61 | 0.86 | 0.28 | 1.20 | 3.00 | 12.01 | 1194.63 |
| [PRPLRLPNGP](https://webs.iiitd.edu.in/raghava/cellppd/pepsearch1.php?seq=PRPLRLPNGP&thval=0.0) | 0.27 | CPP | -0.32 | 0.53 | 0.53 | -1.17 | 0.49 | 0.26 | 1.00 | 2.00 | 12.01 | 1116.47 |
| [KNMKKTSQLK](https://webs.iiitd.edu.in/raghava/cellppd/pepsearch1.php?seq=KNMKKTSQLK&thval=0.0) | 0.24 | CPP | -0.54 | 0.65 | 0.65 | -1.84 | 1.59 | 0.92 | 1.40 | 4.00 | 10.49 | 1205.63 |
| [EKIPKTKNMK](https://webs.iiitd.edu.in/raghava/cellppd/pepsearch1.php?seq=EKIPKTKNMK&thval=0.0) | 0.21 | CPP | -0.49 | 0.65 | 0.65 | -1.85 | 1.59 | 1.17 | 1.20 | 3.00 | 10.01 | 1216.65 |
| [GKVCRTLLAK](https://webs.iiitd.edu.in/raghava/cellppd/pepsearch1.php?seq=GKVCRTLLAK&thval=0.0) | 0.21 | CPP | -0.21 | 0.61 | 0.61 | 0.27 | 0.98 | 0.20 | 0.90 | 3.00 | 10.07 | 1088.52 |
| [SRARIKTRLF](https://webs.iiitd.edu.in/raghava/cellppd/pepsearch1.php?seq=SRARIKTRLF&thval=0.0) | 0.17 | CPP | -0.47 | 0.62 | 0.62 | -0.60 | 1.10 | 0.53 | 1.60 | 4.00 | 12.31 | 1247.64 |
| [KKTSQLKWAL](https://webs.iiitd.edu.in/raghava/cellppd/pepsearch1.php?seq=KKTSQLKWAL&thval=0.0) | 0.14 | CPP | -0.28 | 0.59 | 0.59 | -0.82 | 1.23 | 0.16 | 1.10 | 3.00 | 10.31 | 1202.61 |
| [**Peptide Sequence**](https://webs.iiitd.edu.in/raghava/cellppd/prot_submitfreq.php?ran=31881) | [**SVM score**](https://webs.iiitd.edu.in/raghava/cellppd/prot_submitfreq.php?ran=31881) | [**Prediction**](https://webs.iiitd.edu.in/raghava/cellppd/prot_submitfreq.php?ran=31881) | [**Hydrophobicity**](https://webs.iiitd.edu.in/raghava/cellppd/prot_submitfreq.php?ran=31881) | [**Steric hindrance**](https://webs.iiitd.edu.in/raghava/cellppd/prot_submitfreq.php?ran=31881) | [**Sidebulk**](https://webs.iiitd.edu.in/raghava/cellppd/prot_submitfreq.php?ran=31881) | [**Hydropathicity**](https://webs.iiitd.edu.in/raghava/cellppd/prot_submitfreq.php?ran=31881) | [**Amphipathicity**](https://webs.iiitd.edu.in/raghava/cellppd/prot_submitfreq.php?ran=31881) | [**Hydrophilicity**](https://webs.iiitd.edu.in/raghava/cellppd/prot_submitfreq.php?ran=31881) | [**Net Hydrogen**](https://webs.iiitd.edu.in/raghava/cellppd/prot_submitfreq.php?ran=31881) | [**Charge**](https://webs.iiitd.edu.in/raghava/cellppd/prot_submitfreq.php?ran=31881) | [**pI**](https://webs.iiitd.edu.in/raghava/cellppd/prot_submitfreq.php?ran=31881) | [**Mol wt**](https://webs.iiitd.edu.in/raghava/cellppd/prot_submitfreq.php?ran=31881) |
| [KGRSHLRNDT](https://webs.iiitd.edu.in/raghava/cellppd/pepsearch1.php?seq=KGRSHLRNDT&thval=0.0) | 0.13 | CPP | -0.61 | 0.58 | 0.58 | -2.12 | 1.00 | 0.98 | 1.60 | 2.50 | 10.84 | 1183.43 |
| [TTPRPLRLPN](https://webs.iiitd.edu.in/raghava/cellppd/pepsearch1.php?seq=TTPRPLRLPN&thval=0.0) | 0.09 | CPP | -0.37 | 0.53 | 0.53 | -1.11 | 0.49 | 0.18 | 1.20 | 2.00 | 12.01 | 1164.51 |
| [ARIKTRLFTI](https://webs.iiitd.edu.in/raghava/cellppd/pepsearch1.php?seq=ARIKTRLFTI&thval=0.0) | 0.08 | CPP | -0.21 | 0.63 | 0.63 | 0.31 | 0.86 | -0.02 | 1.20 | 3.00 | 12.01 | 1218.65 |
| [KCMRTFFGWK](https://webs.iiitd.edu.in/raghava/cellppd/pepsearch1.php?seq=KCMRTFFGWK&thval=0.0) | 0.07 | CPP | -0.21 | 0.66 | 0.66 | -0.43 | 0.98 | -0.21 | 1.00 | 3.00 | 10.07 | 1303.74 |
| [EESRARIKTR](https://webs.iiitd.edu.in/raghava/cellppd/pepsearch1.php?seq=EESRARIKTR&thval=0.0) | 0.06 | CPP | -0.71 | 0.64 | 0.64 | -1.96 | 1.36 | 1.56 | 1.80 | 2.00 | 10.75 | 1245.53 |
| [PFLKTTPRPL](https://webs.iiitd.edu.in/raghava/cellppd/pepsearch1.php?seq=PFLKTTPRPL&thval=0.0) | 0.06 | CPP | -0.18 | 0.53 | 0.53 | -0.42 | 0.61 | -0.09 | 0.80 | 2.00 | 11.01 | 1169.57 |
| [NGTSKIKMKW](https://webs.iiitd.edu.in/raghava/cellppd/pepsearch1.php?seq=NGTSKIKMKW&thval=0.0) | 0.06 | CPP | -0.29 | 0.65 | 0.65 | -1.16 | 1.10 | 0.26 | 1.10 | 3.00 | 10.31 | 1192.59 |
| [TPRPLRLPNG](https://webs.iiitd.edu.in/raghava/cellppd/pepsearch1.php?seq=TPRPLRLPNG&thval=0.0) | 0.05 | CPP | -0.33 | 0.55 | 0.55 | -1.08 | 0.49 | 0.22 | 1.10 | 2.00 | 12.01 | 1120.46 |
| [KTKNMKKTSQ](https://webs.iiitd.edu.in/raghava/cellppd/pepsearch1.php?seq=KTKNMKKTSQ&thval=0.0) | 0.05 | CPP | -0.61 | 0.65 | 0.65 | -2.29 | 1.59 | 1.06 | 1.50 | 4.00 | 10.49 | 1193.57 |
| [HIYYLEKANK](https://webs.iiitd.edu.in/raghava/cellppd/pepsearch1.php?seq=HIYYLEKANK&thval=0.0) | 0.04 | CPP | -0.23 | 0.59 | 0.59 | -1.05 | 1.01 | 0.00 | 1.00 | 1.50 | 8.77 | 1278.62 |
| [KMKWGMEMRR](https://webs.iiitd.edu.in/raghava/cellppd/pepsearch1.php?seq=KMKWGMEMRR&thval=0.0) | 0.04 | CPP | -0.50 | 0.69 | 0.69 | -1.59 | 1.35 | 0.77 | 1.40 | 3.00 | 11.01 | 1352.84 |
| [KVCRTLLAKS](https://webs.iiitd.edu.in/raghava/cellppd/pepsearch1.php?seq=KVCRTLLAKS&thval=0.0) | 0.03 | CPP | -0.25 | 0.60 | 0.60 | 0.23 | 0.98 | 0.23 | 1.00 | 3.00 | 10.07 | 1118.54 |
| [RFIEIGVTRR](https://webs.iiitd.edu.in/raghava/cellppd/pepsearch1.php?seq=RFIEIGVTRR&thval=0.0) | 0.02 | CPP | -0.33 | 0.67 | 0.67 | -0.21 | 0.86 | 0.40 | 1.40 | 2.00 | 11.70 | 1246.62 |
| [TKEGRRKTNL](https://webs.iiitd.edu.in/raghava/cellppd/pepsearch1.php?seq=TKEGRRKTNL&thval=0.0) | 0.02 | CPP | -0.66 | 0.64 | 0.64 | -2.18 | 1.35 | 1.26 | 1.70 | 3.00 | 11.01 | 1202.52 |
| [PLRLPNGPPC](https://webs.iiitd.edu.in/raghava/cellppd/pepsearch1.php?seq=PLRLPNGPPC&thval=0.0) | 0.01 | CPP | -0.14 | 0.52 | 0.52 | -0.47 | 0.25 | -0.14 | 0.60 | 1.00 | 8.60 | 1063.42 |
| [PKTKNMKKTS](https://webs.iiitd.edu.in/raghava/cellppd/pepsearch1.php?seq=PKTKNMKKTS&thval=0.0) | 0.01 | CPP | -0.55 | 0.62 | 0.62 | -2.10 | 1.47 | 1.04 | 1.30 | 4.00 | 10.49 | 1162.55 |
| [HCRATEYIMK](https://webs.iiitd.edu.in/raghava/cellppd/pepsearch1.php?seq=HCRATEYIMK&thval=0.0) | 0.01 | CPP | -0.28 | 0.59 | 0.59 | -0.64 | 0.88 | 0.12 | 1.00 | 1.50 | 8.54 | 1251.62 |
| [KEGRRKTNLY](https://webs.iiitd.edu.in/raghava/cellppd/pepsearch1.php?seq=KEGRRKTNLY&thval=0.0) | 0.00 | CPP | -0.64 | 0.66 | 0.66 | -2.24 | 1.35 | 1.07 | 1.70 | 3.00 | 10.29 | 1264.59 |
| **M1** |  |  |  |  |  |  |  |  |  |  |  |  |
| [KAVKLYRKLK](https://webs.iiitd.edu.in/raghava/cellppd/pepsearch1.php?seq=KAVKLYRKLK&thval=0.0) | 0.70 | CPP | -0.43 | 0.64 | 0.64 | -0.78 | 1.71 | 0.71 | 1.30 | 5.00 | 10.59 | 1246.75 |
| [RGLQRRRFVQ](https://webs.iiitd.edu.in/raghava/cellppd/pepsearch1.php?seq=RGLQRRRFVQ&thval=0.0) | 0.41 | CPP | -0.66 | 0.67 | 0.67 | -1.46 | 1.23 | 0.66 | 2.00 | 4.00 | 12.48 | 1315.69 |
| [KLYRKLKREI](https://webs.iiitd.edu.in/raghava/cellppd/pepsearch1.php?seq=KLYRKLKREI&thval=0.0) | 0.40 | CPP | -0.56 | 0.65 | 0.65 | -1.34 | 1.72 | 1.03 | 1.60 | 4.00 | 10.45 | 1346.83 |
| [AVKLYRKLKR](https://webs.iiitd.edu.in/raghava/cellppd/pepsearch1.php?seq=AVKLYRKLKR&thval=0.0) | 0.28 | CPP | -0.49 | 0.64 | 0.64 | -0.84 | 1.59 | 0.71 | 1.50 | 5.00 | 11.17 | 1274.76 |
| [LQRRRFVQNA](https://webs.iiitd.edu.in/raghava/cellppd/pepsearch1.php?seq=LQRRRFVQNA&thval=0.0) | 0.17 | CPP | -0.54 | 0.66 | 0.66 | -1.14 | 0.99 | 0.33 | 1.80 | 3.00 | 12.31 | 1287.63 |
| [RKLKREITFH](https://webs.iiitd.edu.in/raghava/cellppd/pepsearch1.php?seq=RKLKREITFH&thval=0.0) | 0.12 | CPP | -0.51 | 0.59 | 0.59 | -1.31 | 1.50 | 0.80 | 1.50 | 3.50 | 11.01 | 1327.74 |
| [GLQRRRFVQN](https://webs.iiitd.edu.in/raghava/cellppd/pepsearch1.php?seq=GLQRRRFVQN&thval=0.0) | 0.11 | CPP | -0.55 | 0.68 | 0.68 | -1.36 | 0.99 | 0.38 | 1.80 | 3.00 | 12.31 | 1273.61 |
| [VKLYRKLKRE](https://webs.iiitd.edu.in/raghava/cellppd/pepsearch1.php?seq=VKLYRKLKRE&thval=0.0) | 0.11 | CPP | -0.58 | 0.65 | 0.65 | -1.37 | 1.72 | 1.06 | 1.60 | 4.00 | 10.45 | 1332.80 |
| [ERGLQRRRFV](https://webs.iiitd.edu.in/raghava/cellppd/pepsearch1.php?seq=ERGLQRRRFV&thval=0.0) | 0.08 | CPP | -0.65 | 0.67 | 0.67 | -1.46 | 1.23 | 0.94 | 1.90 | 3.00 | 12.01 | 1316.67 |
| [WLKTRPILSP](https://webs.iiitd.edu.in/raghava/cellppd/pepsearch1.php?seq=WLKTRPILSP&thval=0.0) | 0.07 | CPP | -0.13 | 0.54 | 0.54 | -0.19 | 0.61 | -0.29 | 0.90 | 2.00 | 11.01 | 1210.63 |
| [DKAVKLYRKL](https://webs.iiitd.edu.in/raghava/cellppd/pepsearch1.php?seq=DKAVKLYRKL&thval=0.0) | 0.02 | CPP | -0.39 | 0.65 | 0.65 | -0.74 | 1.35 | 0.71 | 1.20 | 3.00 | 10.01 | 1233.66 |
| [**Peptide Sequence**](https://webs.iiitd.edu.in/raghava/cellppd/prot_submitfreq.php?ran=31881) | [**SVM score**](https://webs.iiitd.edu.in/raghava/cellppd/prot_submitfreq.php?ran=31881) | [**Prediction**](https://webs.iiitd.edu.in/raghava/cellppd/prot_submitfreq.php?ran=31881) | [**Hydrophobicity**](https://webs.iiitd.edu.in/raghava/cellppd/prot_submitfreq.php?ran=31881) | [**Steric hindrance**](https://webs.iiitd.edu.in/raghava/cellppd/prot_submitfreq.php?ran=31881) | [**Sidebulk**](https://webs.iiitd.edu.in/raghava/cellppd/prot_submitfreq.php?ran=31881) | [**Hydropathicity**](https://webs.iiitd.edu.in/raghava/cellppd/prot_submitfreq.php?ran=31881) | [**Amphipathicity**](https://webs.iiitd.edu.in/raghava/cellppd/prot_submitfreq.php?ran=31881) | [**Hydrophilicity**](https://webs.iiitd.edu.in/raghava/cellppd/prot_submitfreq.php?ran=31881) | [**Net Hydrogen**](https://webs.iiitd.edu.in/raghava/cellppd/prot_submitfreq.php?ran=31881) | [**Charge**](https://webs.iiitd.edu.in/raghava/cellppd/prot_submitfreq.php?ran=31881) | [**pI**](https://webs.iiitd.edu.in/raghava/cellppd/prot_submitfreq.php?ran=31881) | [**Mol wt**](https://webs.iiitd.edu.in/raghava/cellppd/prot_submitfreq.php?ran=31881) |
| [MDKAVKLYRK](https://webs.iiitd.edu.in/raghava/cellppd/pepsearch1.php?seq=MDKAVKLYRK&thval=0.0) | 0.01 | CPP | -0.42 | 0.67 | 0.67 | -0.93 | 1.35 | 0.76 | 1.20 | 3.00 | 10.01 | 1251.69 |
| **PA-X** |  |  |  |  |  |  |  |  |  |  |  |  |
| [RASCLKCPKK](https://webs.iiitd.edu.in/raghava/cellppd/pepsearch1.php?seq=RASCLKCPKK&thval=0.0) | 0.52 | CPP | -0.45 | 0.59 | 0.59 | -0.80 | 1.35 | 0.80 | 1.10 | 4.00 | 9.86 | 1133.56 |
| [EKRQLKKGLK](https://webs.iiitd.edu.in/raghava/cellppd/pepsearch1.php?seq=EKRQLKKGLK&thval=0.0) | 0.44 | CPP | -0.62 | 0.65 | 0.65 | -1.99 | 1.96 | 1.46 | 1.50 | 4.00 | 10.47 | 1227.67 |
| [RIKTRLFTIR](https://webs.iiitd.edu.in/raghava/cellppd/pepsearch1.php?seq=RIKTRLFTIR&thval=0.0) | 0.43 | CPP | -0.41 | 0.64 | 0.64 | -0.32 | 1.10 | 0.33 | 1.60 | 4.00 | 12.31 | 1303.76 |
| [KRQLKKGLKS](https://webs.iiitd.edu.in/raghava/cellppd/pepsearch1.php?seq=KRQLKKGLKS&thval=0.0) | 0.36 | CPP | -0.59 | 0.64 | 0.64 | -1.72 | 1.84 | 1.19 | 1.50 | 5.00 | 11.34 | 1185.63 |
| [ESRARIKTRL](https://webs.iiitd.edu.in/raghava/cellppd/pepsearch1.php?seq=ESRARIKTRL&thval=0.0) | 0.35 | CPP | -0.59 | 0.62 | 0.62 | -1.23 | 1.23 | 1.08 | 1.70 | 3.00 | 11.72 | 1229.58 |
| [RARIKTRLFT](https://webs.iiitd.edu.in/raghava/cellppd/pepsearch1.php?seq=RARIKTRLFT&thval=0.0) | 0.33 | CPP | -0.46 | 0.62 | 0.62 | -0.59 | 1.10 | 0.46 | 1.60 | 4.00 | 12.31 | 1261.67 |
| [RQLKKGLKSQ](https://webs.iiitd.edu.in/raghava/cellppd/pepsearch1.php?seq=RQLKKGLKSQ&thval=0.0) | 0.24 | CPP | -0.55 | 0.64 | 0.64 | -1.68 | 1.60 | 0.91 | 1.50 | 4.00 | 11.27 | 1185.59 |
| [REEKRQLKKG](https://webs.iiitd.edu.in/raghava/cellppd/pepsearch1.php?seq=REEKRQLKKG&thval=0.0) | 0.20 | CPP | -0.81 | 0.66 | 0.66 | -2.78 | 1.97 | 1.94 | 1.80 | 3.00 | 10.29 | 1271.63 |
| [SRARIKTRLF](https://webs.iiitd.edu.in/raghava/cellppd/pepsearch1.php?seq=SRARIKTRLF&thval=0.0) | 0.17 | CPP | -0.47 | 0.62 | 0.62 | -0.60 | 1.10 | 0.53 | 1.60 | 4.00 | 12.31 | 1247.64 |
| [PREEKRQLKK](https://webs.iiitd.edu.in/raghava/cellppd/pepsearch1.php?seq=PREEKRQLKK&thval=0.0) | 0.17 | CPP | -0.83 | 0.63 | 0.63 | -2.90 | 1.97 | 1.94 | 1.80 | 3.00 | 10.29 | 1311.69 |
| [LRASCLKCPK](https://webs.iiitd.edu.in/raghava/cellppd/pepsearch1.php?seq=LRASCLKCPK&thval=0.0) | 0.15 | CPP | -0.29 | 0.57 | 0.57 | -0.03 | 0.98 | 0.32 | 0.90 | 3.00 | 9.53 | 1118.55 |
| [KVSRRTSPAL](https://webs.iiitd.edu.in/raghava/cellppd/pepsearch1.php?seq=KVSRRTSPAL&thval=0.0) | 0.13 | CPP | -0.41 | 0.57 | 0.57 | -0.70 | 0.86 | 0.54 | 1.30 | 3.00 | 12.01 | 1114.44 |
| [ARIKTRLFTI](https://webs.iiitd.edu.in/raghava/cellppd/pepsearch1.php?seq=ARIKTRLFTI&thval=0.0) | 0.08 | CPP | -0.21 | 0.63 | 0.63 | 0.31 | 0.86 | -0.02 | 1.20 | 3.00 | 12.01 | 1218.65 |
| [PTKVSRRTSP](https://webs.iiitd.edu.in/raghava/cellppd/pepsearch1.php?seq=PTKVSRRTSP&thval=0.0) | 0.08 | CPP | -0.51 | 0.56 | 0.56 | -1.49 | 0.86 | 0.73 | 1.40 | 3.00 | 12.01 | 1128.42 |
| [VSRRTSPALK](https://webs.iiitd.edu.in/raghava/cellppd/pepsearch1.php?seq=VSRRTSPALK&thval=0.0) | 0.08 | CPP | -0.41 | 0.57 | 0.57 | -0.70 | 0.86 | 0.54 | 1.30 | 3.00 | 12.01 | 1114.44 |
| [NRTATLRASC](https://webs.iiitd.edu.in/raghava/cellppd/pepsearch1.php?seq=NRTATLRASC&thval=0.0) | 0.08 | CPP | -0.37 | 0.59 | 0.59 | -0.48 | 0.49 | 0.19 | 1.30 | 2.00 | 10.38 | 1092.36 |
| [EESRARIKTR](https://webs.iiitd.edu.in/raghava/cellppd/pepsearch1.php?seq=EESRARIKTR&thval=0.0) | 0.06 | CPP | -0.71 | 0.64 | 0.64 | -1.96 | 1.36 | 1.56 | 1.80 | 2.00 | 10.75 | 1245.53 |
| [SRRTSPALKI](https://webs.iiitd.edu.in/raghava/cellppd/pepsearch1.php?seq=SRRTSPALKI&thval=0.0) | 0.06 | CPP | -0.39 | 0.57 | 0.57 | -0.67 | 0.86 | 0.51 | 1.30 | 3.00 | 12.01 | 1128.47 |
| [SNRTATLRAS](https://webs.iiitd.edu.in/raghava/cellppd/pepsearch1.php?seq=SNRTATLRAS&thval=0.0) | 0.06 | CPP | -0.40 | 0.58 | 0.58 | -0.81 | 0.49 | 0.32 | 1.40 | 2.00 | 12.01 | 1076.30 |
| [HIYYLEKANK](https://webs.iiitd.edu.in/raghava/cellppd/pepsearch1.php?seq=HIYYLEKANK&thval=0.0) | 0.04 | CPP | -0.23 | 0.59 | 0.59 | -1.05 | 1.01 | 0.00 | 1.00 | 1.50 | 8.77 | 1278.62 |
| [RFIEIGVTRR](https://webs.iiitd.edu.in/raghava/cellppd/pepsearch1.php?seq=RFIEIGVTRR&thval=0.0) | 0.02 | CPP | -0.33 | 0.67 | 0.67 | -0.21 | 0.86 | 0.40 | 1.40 | 2.00 | 11.70 | 1246.62 |
| [ASLPTKVSRR](https://webs.iiitd.edu.in/raghava/cellppd/pepsearch1.php?seq=ASLPTKVSRR&thval=0.0) | 0.02 | CPP | -0.41 | 0.57 | 0.57 | -0.70 | 0.86 | 0.54 | 1.30 | 3.00 | 12.01 | 1114.44 |
| [TKVSRRTSPA](https://webs.iiitd.edu.in/raghava/cellppd/pepsearch1.php?seq=TKVSRRTSPA&thval=0.0) | 0.02 | CPP | -0.48 | 0.57 | 0.57 | -1.15 | 0.86 | 0.68 | 1.40 | 3.00 | 12.01 | 1102.38 |
| [RRTSPALKIL](https://webs.iiitd.edu.in/raghava/cellppd/pepsearch1.php?seq=RRTSPALKIL&thval=0.0) | 0.02 | CPP | -0.31 | 0.57 | 0.57 | -0.21 | 0.86 | 0.30 | 1.20 | 3.00 | 12.01 | 1154.56 |
| [SPREEKRQLK](https://webs.iiitd.edu.in/raghava/cellppd/pepsearch1.php?seq=SPREEKRQLK&thval=0.0) | 0.00 | CPP | -0.74 | 0.62 | 0.62 | -2.59 | 1.60 | 1.67 | 1.70 | 2.00 | 10.00 | 1270.59 |
| **PB1** |  |  |  |  |  |  |  |  |  |  |  |  |
| [FQRKRRVRDN](https://webs.iiitd.edu.in/raghava/cellppd/pepsearch1.php?seq=FQRKRRVRDN&thval=0.0) | 0.57 | CPP | -0.90 | 0.70 | 0.70 | -2.54 | 1.47 | 1.44 | 2.30 | 4.00 | 12.01 | 1374.70 |
| [THFQRKRRVR](https://webs.iiitd.edu.in/raghava/cellppd/pepsearch1.php?seq=THFQRKRRVR&thval=0.0) | 0.53 | CPP | -0.83 | 0.60 | 0.60 | -2.23 | 1.62 | 1.03 | 2.20 | 5.50 | 12.48 | 1383.76 |
| [**Peptide Sequence**](https://webs.iiitd.edu.in/raghava/cellppd/prot_submitfreq.php?ran=31881) | [**SVM score**](https://webs.iiitd.edu.in/raghava/cellppd/prot_submitfreq.php?ran=31881) | [**Prediction**](https://webs.iiitd.edu.in/raghava/cellppd/prot_submitfreq.php?ran=31881) | [**Hydrophobicity**](https://webs.iiitd.edu.in/raghava/cellppd/prot_submitfreq.php?ran=31881) | [**Steric hindrance**](https://webs.iiitd.edu.in/raghava/cellppd/prot_submitfreq.php?ran=31881) | [**Sidebulk**](https://webs.iiitd.edu.in/raghava/cellppd/prot_submitfreq.php?ran=31881) | [**Hydropathicity**](https://webs.iiitd.edu.in/raghava/cellppd/prot_submitfreq.php?ran=31881) | [**Amphipathicity**](https://webs.iiitd.edu.in/raghava/cellppd/prot_submitfreq.php?ran=31881) | [**Hydrophilicity**](https://webs.iiitd.edu.in/raghava/cellppd/prot_submitfreq.php?ran=31881) | [**Net Hydrogen**](https://webs.iiitd.edu.in/raghava/cellppd/prot_submitfreq.php?ran=31881) | [**Charge**](https://webs.iiitd.edu.in/raghava/cellppd/prot_submitfreq.php?ran=31881) | [**pI**](https://webs.iiitd.edu.in/raghava/cellppd/prot_submitfreq.php?ran=31881) | [**Mol wt**](https://webs.iiitd.edu.in/raghava/cellppd/prot_submitfreq.php?ran=31881) |
| [KMITQRTIGK](https://webs.iiitd.edu.in/raghava/cellppd/pepsearch1.php?seq=KMITQRTIGK&thval=0.0) | 0.39 | CPP | -0.31 | 0.66 | 0.66 | -0.67 | 1.10 | 0.35 | 1.20 | 3.00 | 11.17 | 1175.61 |
| [RRVRDNMTKK](https://webs.iiitd.edu.in/raghava/cellppd/pepsearch1.php?seq=RRVRDNMTKK&thval=0.0) | 0.33 | CPP | -0.82 | 0.69 | 0.69 | -2.29 | 1.47 | 1.50 | 2.00 | 4.00 | 11.73 | 1303.68 |
| [GITTHFQRKR](https://webs.iiitd.edu.in/raghava/cellppd/pepsearch1.php?seq=GITTHFQRKR&thval=0.0) | 0.30 | CPP | -0.46 | 0.59 | 0.59 | -1.41 | 1.13 | 0.36 | 1.50 | 3.50 | 12.01 | 1243.58 |
| [HFQRKRRVRD](https://webs.iiitd.edu.in/raghava/cellppd/pepsearch1.php?seq=HFQRKRRVRD&thval=0.0) | 0.30 | CPP | -0.88 | 0.62 | 0.62 | -2.51 | 1.62 | 1.37 | 2.20 | 4.50 | 12.01 | 1397.74 |
| [KKIEKIRPLL](https://webs.iiitd.edu.in/raghava/cellppd/pepsearch1.php?seq=KKIEKIRPLL&thval=0.0) | 0.30 | CPP | -0.32 | 0.62 | 0.62 | -0.47 | 1.47 | 0.78 | 1.10 | 3.00 | 10.30 | 1237.75 |
| [RLNKRSYLIR](https://webs.iiitd.edu.in/raghava/cellppd/pepsearch1.php?seq=RLNKRSYLIR&thval=0.0) | 0.29 | CPP | -0.55 | 0.65 | 0.65 | -1.09 | 1.10 | 0.48 | 1.80 | 4.00 | 11.73 | 1318.73 |
| [DAERGKLKRR](https://webs.iiitd.edu.in/raghava/cellppd/pepsearch1.php?seq=DAERGKLKRR&thval=0.0) | 0.29 | CPP | -0.79 | 0.66 | 0.66 | -2.31 | 1.60 | 1.87 | 1.80 | 3.00 | 10.91 | 1228.55 |
| [ERGKLKRRAI](https://webs.iiitd.edu.in/raghava/cellppd/pepsearch1.php?seq=ERGKLKRRAI&thval=0.0) | 0.28 | CPP | -0.64 | 0.65 | 0.65 | -1.51 | 1.60 | 1.39 | 1.70 | 4.00 | 11.73 | 1226.63 |
| [KDAERGKLKR](https://webs.iiitd.edu.in/raghava/cellppd/pepsearch1.php?seq=KDAERGKLKR&thval=0.0) | 0.27 | CPP | -0.72 | 0.66 | 0.66 | -2.25 | 1.72 | 1.87 | 1.60 | 3.00 | 10.29 | 1200.54 |
| [QRKRRVRDNM](https://webs.iiitd.edu.in/raghava/cellppd/pepsearch1.php?seq=QRKRRVRDNM&thval=0.0) | 0.26 | CPP | -0.94 | 0.71 | 0.71 | -2.63 | 1.47 | 1.56 | 2.30 | 4.00 | 12.01 | 1358.72 |
| [AERGKLKRRA](https://webs.iiitd.edu.in/raghava/cellppd/pepsearch1.php?seq=AERGKLKRRA&thval=0.0) | 0.26 | CPP | -0.69 | 0.63 | 0.63 | -1.78 | 1.60 | 1.52 | 1.70 | 4.00 | 11.73 | 1184.54 |
| [KRRVRDNMTK](https://webs.iiitd.edu.in/raghava/cellppd/pepsearch1.php?seq=KRRVRDNMTK&thval=0.0) | 0.25 | CPP | -0.82 | 0.69 | 0.69 | -2.29 | 1.47 | 1.50 | 2.00 | 4.00 | 11.73 | 1303.68 |
| [TQRTIGKRKQ](https://webs.iiitd.edu.in/raghava/cellppd/pepsearch1.php?seq=TQRTIGKRKQ&thval=0.0) | 0.24 | CPP | -0.66 | 0.65 | 0.65 | -2.11 | 1.47 | 0.98 | 1.80 | 4.00 | 12.02 | 1215.57 |
| [RGKLKRRAIA](https://webs.iiitd.edu.in/raghava/cellppd/pepsearch1.php?seq=RGKLKRRAIA&thval=0.0) | 0.23 | CPP | -0.56 | 0.64 | 0.64 | -0.98 | 1.47 | 1.04 | 1.60 | 5.00 | 12.31 | 1168.59 |
| [SWIPKRNRSI](https://webs.iiitd.edu.in/raghava/cellppd/pepsearch1.php?seq=SWIPKRNRSI&thval=0.0) | 0.22 | CPP | -0.40 | 0.61 | 0.61 | -1.15 | 0.86 | 0.28 | 1.50 | 3.00 | 12.01 | 1256.61 |
| [MITQRTIGKR](https://webs.iiitd.edu.in/raghava/cellppd/pepsearch1.php?seq=MITQRTIGKR&thval=0.0) | 0.21 | CPP | -0.38 | 0.66 | 0.66 | -0.73 | 0.98 | 0.35 | 1.40 | 3.00 | 12.01 | 1203.62 |
| [GKLKRRAIAT](https://webs.iiitd.edu.in/raghava/cellppd/pepsearch1.php?seq=GKLKRRAIAT&thval=0.0) | 0.21 | CPP | -0.40 | 0.62 | 0.62 | -0.60 | 1.22 | 0.70 | 1.30 | 4.00 | 12.02 | 1113.51 |
| [KRRAIATPGM](https://webs.iiitd.edu.in/raghava/cellppd/pepsearch1.php?seq=KRRAIATPGM&thval=0.0) | 0.20 | CPP | -0.32 | 0.61 | 0.61 | -0.56 | 0.86 | 0.45 | 1.10 | 3.00 | 12.01 | 1100.48 |
| [KKLWEQTRSK](https://webs.iiitd.edu.in/raghava/cellppd/pepsearch1.php?seq=KKLWEQTRSK&thval=0.0) | 0.20 | CPP | -0.59 | 0.62 | 0.62 | -2.18 | 1.60 | 0.99 | 1.60 | 3.00 | 10.30 | 1303.67 |
| [ITTHFQRKRR](https://webs.iiitd.edu.in/raghava/cellppd/pepsearch1.php?seq=ITTHFQRKRR&thval=0.0) | 0.19 | CPP | -0.65 | 0.59 | 0.59 | -1.82 | 1.37 | 0.66 | 1.90 | 4.50 | 12.31 | 1342.71 |
| [TTHFQRKRRV](https://webs.iiitd.edu.in/raghava/cellppd/pepsearch1.php?seq=TTHFQRKRRV&thval=0.0) | 0.19 | CPP | -0.67 | 0.59 | 0.59 | -1.85 | 1.37 | 0.69 | 1.90 | 4.50 | 12.31 | 1328.68 |
| [RKRRVRDNMT](https://webs.iiitd.edu.in/raghava/cellppd/pepsearch1.php?seq=RKRRVRDNMT&thval=0.0) | 0.19 | CPP | -0.89 | 0.69 | 0.69 | -2.35 | 1.35 | 1.50 | 2.20 | 4.00 | 12.01 | 1331.69 |
| [KSMKLRTQIP](https://webs.iiitd.edu.in/raghava/cellppd/pepsearch1.php?seq=KSMKLRTQIP&thval=0.0) | 0.19 | CPP | -0.36 | 0.61 | 0.61 | -0.87 | 1.10 | 0.42 | 1.20 | 3.00 | 11.17 | 1201.64 |
| [TRKKIEKIRP](https://webs.iiitd.edu.in/raghava/cellppd/pepsearch1.php?seq=TRKKIEKIRP&thval=0.0) | 0.19 | CPP | -0.62 | 0.64 | 0.64 | -1.75 | 1.72 | 1.40 | 1.60 | 4.00 | 11.10 | 1268.71 |
| [HSWIPKRNRS](https://webs.iiitd.edu.in/raghava/cellppd/pepsearch1.php?seq=HSWIPKRNRS&thval=0.0) | 0.19 | CPP | -0.52 | 0.54 | 0.54 | -1.92 | 1.00 | 0.41 | 1.60 | 3.50 | 12.01 | 1280.59 |
| [WIPKRNRSIL](https://webs.iiitd.edu.in/raghava/cellppd/pepsearch1.php?seq=WIPKRNRSIL&thval=0.0) | 0.17 | CPP | -0.32 | 0.61 | 0.61 | -0.69 | 0.86 | 0.07 | 1.40 | 3.00 | 12.01 | 1282.70 |
| [STRKKIEKIR](https://webs.iiitd.edu.in/raghava/cellppd/pepsearch1.php?seq=STRKKIEKIR&thval=0.0) | 0.16 | CPP | -0.64 | 0.65 | 0.65 | -1.67 | 1.72 | 1.43 | 1.70 | 4.00 | 11.10 | 1258.67 |
| [MGITTHFQRK](https://webs.iiitd.edu.in/raghava/cellppd/pepsearch1.php?seq=MGITTHFQRK&thval=0.0) | 0.15 | CPP | -0.26 | 0.60 | 0.60 | -0.77 | 0.88 | -0.07 | 1.10 | 2.50 | 11.01 | 1218.59 |
| [KYFNDSTRKK](https://webs.iiitd.edu.in/raghava/cellppd/pepsearch1.php?seq=KYFNDSTRKK&thval=0.0) | 0.14 | CPP | -0.62 | 0.67 | 0.67 | -2.32 | 1.35 | 1.03 | 1.60 | 3.00 | 10.01 | 1286.58 |
| [NDSTRKKIEK](https://webs.iiitd.edu.in/raghava/cellppd/pepsearch1.php?seq=NDSTRKKIEK&thval=0.0) | 0.14 | CPP | -0.68 | 0.67 | 0.67 | -2.37 | 1.47 | 1.63 | 1.60 | 2.00 | 9.72 | 1218.51 |
| [**Peptide Sequence**](https://webs.iiitd.edu.in/raghava/cellppd/prot_submitfreq.php?ran=31881) | [**SVM score**](https://webs.iiitd.edu.in/raghava/cellppd/prot_submitfreq.php?ran=31881) | [**Prediction**](https://webs.iiitd.edu.in/raghava/cellppd/prot_submitfreq.php?ran=31881) | [**Hydrophobicity**](https://webs.iiitd.edu.in/raghava/cellppd/prot_submitfreq.php?ran=31881) | [**Steric hindrance**](https://webs.iiitd.edu.in/raghava/cellppd/prot_submitfreq.php?ran=31881) | [**Sidebulk**](https://webs.iiitd.edu.in/raghava/cellppd/prot_submitfreq.php?ran=31881) | [**Hydropathicity**](https://webs.iiitd.edu.in/raghava/cellppd/prot_submitfreq.php?ran=31881) | [**Amphipathicity**](https://webs.iiitd.edu.in/raghava/cellppd/prot_submitfreq.php?ran=31881) | [**Hydrophilicity**](https://webs.iiitd.edu.in/raghava/cellppd/prot_submitfreq.php?ran=31881) | [**Net Hydrogen**](https://webs.iiitd.edu.in/raghava/cellppd/prot_submitfreq.php?ran=31881) | [**Charge**](https://webs.iiitd.edu.in/raghava/cellppd/prot_submitfreq.php?ran=31881) | [**pI**](https://webs.iiitd.edu.in/raghava/cellppd/prot_submitfreq.php?ran=31881) | [**Mol wt**](https://webs.iiitd.edu.in/raghava/cellppd/prot_submitfreq.php?ran=31881) |
| [KIEKIRPLLI](https://webs.iiitd.edu.in/raghava/cellppd/pepsearch1.php?seq=KIEKIRPLLI&thval=0.0) | 0.13 | CPP | -0.14 | 0.62 | 0.62 | 0.37 | 1.11 | 0.30 | 0.90 | 2.00 | 10.01 | 1222.74 |
| [KLWEQTRSKA](https://webs.iiitd.edu.in/raghava/cellppd/pepsearch1.php?seq=KLWEQTRSKA&thval=0.0) | 0.13 | CPP | -0.46 | 0.60 | 0.60 | -1.61 | 1.23 | 0.64 | 1.40 | 2.00 | 10.01 | 1246.57 |
| [KLKRRAIATP](https://webs.iiitd.edu.in/raghava/cellppd/pepsearch1.php?seq=KLKRRAIATP&thval=0.0) | 0.12 | CPP | -0.42 | 0.59 | 0.59 | -0.72 | 1.22 | 0.70 | 1.30 | 4.00 | 12.02 | 1153.57 |
| [GINMSKKKSY](https://webs.iiitd.edu.in/raghava/cellppd/pepsearch1.php?seq=GINMSKKKSY&thval=0.0) | 0.12 | CPP | -0.33 | 0.67 | 0.67 | -1.21 | 1.10 | 0.44 | 1.10 | 3.00 | 10.01 | 1155.52 |
| [ITQRTIGKRK](https://webs.iiitd.edu.in/raghava/cellppd/pepsearch1.php?seq=ITQRTIGKRK&thval=0.0) | 0.11 | CPP | -0.52 | 0.65 | 0.65 | -1.31 | 1.35 | 0.78 | 1.60 | 4.00 | 12.02 | 1200.60 |
| [MSKKKSYINR](https://webs.iiitd.edu.in/raghava/cellppd/pepsearch1.php?seq=MSKKKSYINR&thval=0.0) | 0.11 | CPP | -0.52 | 0.67 | 0.67 | -1.62 | 1.35 | 0.74 | 1.50 | 4.00 | 10.47 | 1254.65 |
| [RQTYDWTLNR](https://webs.iiitd.edu.in/raghava/cellppd/pepsearch1.php?seq=RQTYDWTLNR&thval=0.0) | 0.10 | CPP | -0.50 | 0.64 | 0.64 | -1.93 | 0.61 | 0.11 | 1.70 | 1.00 | 9.10 | 1352.61 |
| [QTRRSFEIKK](https://webs.iiitd.edu.in/raghava/cellppd/pepsearch1.php?seq=QTRRSFEIKK&thval=0.0) | 0.09 | CPP | -0.61 | 0.65 | 0.65 | -1.80 | 1.48 | 1.08 | 1.70 | 3.00 | 11.01 | 1292.64 |
| [THSWIPKRNR](https://webs.iiitd.edu.in/raghava/cellppd/pepsearch1.php?seq=THSWIPKRNR&thval=0.0) | 0.09 | CPP | -0.51 | 0.54 | 0.54 | -1.91 | 1.00 | 0.34 | 1.60 | 3.50 | 12.01 | 1294.62 |
| [DRFYRTCKLH](https://webs.iiitd.edu.in/raghava/cellppd/pepsearch1.php?seq=DRFYRTCKLH&thval=0.0) | 0.07 | CPP | -0.47 | 0.59 | 0.59 | -1.25 | 1.00 | 0.35 | 1.40 | 2.50 | 9.36 | 1338.68 |
| [NLGQKRYTKT](https://webs.iiitd.edu.in/raghava/cellppd/pepsearch1.php?seq=NLGQKRYTKT&thval=0.0) | 0.06 | CPP | -0.49 | 0.65 | 0.65 | -1.86 | 1.10 | 0.45 | 1.50 | 3.00 | 10.30 | 1208.53 |
| RGD[TQIQTRR](https://webs.iiitd.edu.in/raghava/cellppd/pepsearch1.php?seq=RGDTQIQTRR&thval=0.0) | 0.06 | CPP | -0.69 | 0.66 | 0.66 | -2.13 | 0.99 | 0.98 | 1.90 | 2.00 | 11.70 | 1230.49 |
| [RRSFEIKKLW](https://webs.iiitd.edu.in/raghava/cellppd/pepsearch1.php?seq=RRSFEIKKLW&thval=0.0) | 0.06 | CPP | -0.44 | 0.64 | 0.64 | -1.09 | 1.35 | 0.58 | 1.50 | 3.00 | 11.01 | 1362.78 |
| [RARIDARIDF](https://webs.iiitd.edu.in/raghava/cellppd/pepsearch1.php?seq=RARIDARIDF&thval=0.0) | 0.06 | CPP | -0.41 | 0.67 | 0.67 | -0.51 | 0.74 | 0.79 | 1.40 | 1.00 | 9.86 | 1232.53 |
| [KRSYLIRALT](https://webs.iiitd.edu.in/raghava/cellppd/pepsearch1.php?seq=KRSYLIRALT&thval=0.0) | 0.04 | CPP | -0.30 | 0.61 | 0.61 | -0.18 | 0.86 | 0.07 | 1.30 | 3.00 | 11.01 | 1220.62 |
| [KRYTKTTYWW](https://webs.iiitd.edu.in/raghava/cellppd/pepsearch1.php?seq=KRYTKTTYWW&thval=0.0) | 0.04 | CPP | -0.37 | 0.60 | 0.60 | -1.88 | 0.98 | -0.36 | 1.50 | 3.00 | 10.01 | 1432.78 |
| [TRRSFEIKKL](https://webs.iiitd.edu.in/raghava/cellppd/pepsearch1.php?seq=TRRSFEIKKL&thval=0.0) | 0.04 | CPP | -0.49 | 0.64 | 0.64 | -1.07 | 1.35 | 0.88 | 1.50 | 3.00 | 11.01 | 1277.67 |
| [RKKIEKIRPL](https://webs.iiitd.edu.in/raghava/cellppd/pepsearch1.php?seq=RKKIEKIRPL&thval=0.0) | 0.03 | CPP | -0.55 | 0.64 | 0.64 | -1.30 | 1.72 | 1.26 | 1.50 | 4.00 | 11.10 | 1280.77 |
| [GVDRFYRTCK](https://webs.iiitd.edu.in/raghava/cellppd/pepsearch1.php?seq=GVDRFYRTCK&thval=0.0) | 0.02 | CPP | -0.42 | 0.67 | 0.67 | -0.93 | 0.86 | 0.43 | 1.30 | 2.00 | 9.36 | 1244.56 |
| [PSSSYRRPVG](https://webs.iiitd.edu.in/raghava/cellppd/pepsearch1.php?seq=PSSSYRRPVG&thval=0.0) | 0.02 | CPP | -0.37 | 0.57 | 0.57 | -1.21 | 0.49 | 0.31 | 1.20 | 2.00 | 10.84 | 1105.34 |
| [MVSRARIDAR](https://webs.iiitd.edu.in/raghava/cellppd/pepsearch1.php?seq=MVSRARIDAR&thval=0.0) | 0.02 | CPP | -0.42 | 0.65 | 0.65 | -0.36 | 0.74 | 0.67 | 1.40 | 2.00 | 11.70 | 1174.51 |
| [TQIQTRRSFE](https://webs.iiitd.edu.in/raghava/cellppd/pepsearch1.php?seq=TQIQTRRSFE&thval=0.0) | 0.01 | CPP | -0.48 | 0.64 | 0.64 | -1.44 | 0.87 | 0.46 | 1.60 | 1.00 | 9.95 | 1265.53 |
| **NEP** |  |  |  |  |  |  |  |  |  |  |  |  |
| [FQDILLRMSK](https://webs.iiitd.edu.in/raghava/cellppd/pepsearch1.php?seq=FQDILLRMSK&thval=0.0) | 0.10 | CPP | -0.19 | 0.66 | 0.66 | 0.06 | 0.74 | 0.03 | 1.00 | 1.00 | 9.10 | 1250.67 |
| [LQNRNEKWRE](https://webs.iiitd.edu.in/raghava/cellppd/pepsearch1.php?seq=LQNRNEKWRE&thval=0.0) | 0.00 | CPP | -0.69 | 0.66 | 0.66 | -2.75 | 1.24 | 1.04 | 1.90 | 1.00 | 9.10 | 1372.65 |

**Table S4. Membrane-binding potential (Boman index) and predicted cellular localization of cell-penetrating peptide (CPP) candidates by TMHMM Server.**

| **Epitope** | **Protein binding Potential**  **(Boman index)** | **Cellular localization by**  **TMHMM Server** | **Total probability of N-in by TMHMM Server** |
| --- | --- | --- | --- |
| **NP** |  |  |  |
| RSRYWAIRTR | 5.67 | inside | 0.95423 |
| RMIKRGINDR | 5.25 | inside | 0.92017 |
| GPIYRRVNGK | 3.13 | inside | 0.73968 |
| KGTKVVPRGK | 2.41 | inside | 0.75226 |
| RRIWRQANNG | 5.35 | inside | 0.92502 |
| YQRTRALVRT | 4.48 | inside | 0.92401 |
| RYWAIRTRSG | 4.08 | inside | 0.88689 |
| YRRVNGKWMR | 4.74 | inside | 0.93136 |
| GRKTRIAYER | 5.21 | inside | 0.92892 |
| RTRALVRTGM | 3.58 | inside | 0.85566 |
| SRYWAIRTRS | 4.52 | inside | 0.90599 |
| HPSAGKDPKK | 3.06 | inside | 0.43309 |
| KEEIRRIWRQ | 5.73 | inside | 0.92411 |
| EIRRIWRQAN | 4.97 | inside | 0.92004 |
| RGENGRKTRI | 5.95 | inside | 0.90632 |
| EEIRRIWRQA | 4.99 | inside | 0.88724 |
| FLARSALILR | 0.69 | outside | 0.32148 |
| ESSTLELRSR | 4.63 | inside | 0.50808 |
| IRRIWRQANN | 4.96 | inside | 0.94367 |
| RNFWRGENGR | 5.76 | inside | 0.77549 |
| RKTRIAYERM | 5.07 | inside | 0.94992 |
| LSAFDERRNK | 5.12 | inside | 0.70898 |
| GKWMRELILY | 0.7 | inside | 0.49152 |
| TYQRTRALVR | 4.48 | inside | 0.92401 |
| **Epitope** | **Protein binding Potential**  **(Boman index)** | **Cellular localization by**  **TMHMM Server** | **Total probability of N-in by TMHMM Server** |
| RGINDRNFWR | 5.55 | inside | 0.83912 |
| WRGENGRKTR | 6.21 | inside | 0.90495 |
| PSAGKDPKKT | 2.85 | inside | 0.50257 |
| **HA** |  |  |  |
| [KNSYVNKKGK](https://webs.iiitd.edu.in/raghava/cellppd/pepsearch1.php?seq=KNSYVNKKGK&thval=0.0) | 3.4 | inside | 0.86954 |
| [KLCRLKGIAP](https://webs.iiitd.edu.in/raghava/cellppd/pepsearch1.php?seq=KLCRLKGIAP&thval=0.0) | 0.72 | inside | 0.54263 |
| [PEIAERPKVR](https://webs.iiitd.edu.in/raghava/cellppd/pepsearch1.php?seq=PEIAERPKVR&thval=0.0) | 3.82 | inside | 0.64877 |
| [KLKNSYVNKK](https://webs.iiitd.edu.in/raghava/cellppd/pepsearch1.php?seq=KLKNSYVNKK&thval=0.0) | 3 | inside | 0.85748 |
| [PKYVRSAKLR](https://webs.iiitd.edu.in/raghava/cellppd/pepsearch1.php?seq=PKYVRSAKLR&thval=0.0) | 3.37 | inside | 0.79761 |
| [KYVRSAKLRM](https://webs.iiitd.edu.in/raghava/cellppd/pepsearch1.php?seq=KYVRSAKLRM&thval=0.0) | 3.13 | inside | 0.87779 |
| [MNYYWTLLKP](https://webs.iiitd.edu.in/raghava/cellppd/pepsearch1.php?seq=MNYYWTLLKP&thval=0.0) | 0.05 | inside | 0.41464 |
| [HNGKLCRLKG](https://webs.iiitd.edu.in/raghava/cellppd/pepsearch1.php?seq=HNGKLCRLKG&thval=0.0) | 2.43 | inside | 0.64037 |
| [GKLCRLKGIA](https://webs.iiitd.edu.in/raghava/cellppd/pepsearch1.php?seq=GKLCRLKGIA&thval=0.0) | 0.62 | inside | 0.58738 |
| [GKEFNKLEKR](https://webs.iiitd.edu.in/raghava/cellppd/pepsearch1.php?seq=GKEFNKLEKR&thval=0.0) | 4.29 | inside | 0.70917 |
| [RRFTPEIAER](https://webs.iiitd.edu.in/raghava/cellppd/pepsearch1.php?seq=RRFTPEIAER&thval=0.0) | 5.12 | inside | 0.74641 |
| **NS1** |  |  |  |
| [RLRRDQKSLR](https://webs.iiitd.edu.in/raghava/cellppd/pepsearch1.php?seq=RLRRDQKSLR&thval=0.0) | 7.3 | inside | 0.90538 |
| [RRDQKSLRGR](https://webs.iiitd.edu.in/raghava/cellppd/pepsearch1.php?seq=RRDQKSLRGR&thval=0.0) | 7.7 | inside | 0.92177 |
| [FLDRLRRDQK](https://webs.iiitd.edu.in/raghava/cellppd/pepsearch1.php?seq=FLDRLRRDQK&thval=0.0) | 6.04 | inside | 0.77337 |
| [RPPLTPKQKR](https://webs.iiitd.edu.in/raghava/cellppd/pepsearch1.php?seq=RPPLTPKQKR&thval=0.0) | 4.41 | inside | 0.70216 |
| [DAPFLDRLRR](https://webs.iiitd.edu.in/raghava/cellppd/pepsearch1.php?seq=DAPFLDRLRR&thval=0.0) | 4.75 | inside | 0.49872 |
| [DRLRRDQKSL](https://webs.iiitd.edu.in/raghava/cellppd/pepsearch1.php?seq=DRLRRDQKSL&thval=0.0) | 6.68 | inside | 0.83323 |
| [FLWHVRKRVA](https://webs.iiitd.edu.in/raghava/cellppd/pepsearch1.php?seq=FLWHVRKRVA&thval=0.0) | 1.99 | inside | 0.68760 |
| [DCFLWHVRKR](https://webs.iiitd.edu.in/raghava/cellppd/pepsearch1.php?seq=DCFLWHVRKR&thval=0.0) | 3.32 | inside | 0.73934 |
| [APFLDRLRRD](https://webs.iiitd.edu.in/raghava/cellppd/pepsearch1.php?seq=APFLDRLRRD&thval=0.0) | 4.75 | inside | 0.49872 |
| [EWSMLIPKQK](https://webs.iiitd.edu.in/raghava/cellppd/pepsearch1.php?seq=EWSMLIPKQK&thval=0.0) | 1.23 | inside | 0.52200 |
| [LQRFAWRSSN](https://webs.iiitd.edu.in/raghava/cellppd/pepsearch1.php?seq=LQRFAWRSSN&thval=0.0) | 3.67 | inside | 0.65956 |
| [GKQIVERILK](https://webs.iiitd.edu.in/raghava/cellppd/pepsearch1.php?seq=GKQIVERILK&thval=0.0) | 1.86 | inside | 0.72076 |
| [CFLWHVRKRV](https://webs.iiitd.edu.in/raghava/cellppd/pepsearch1.php?seq=CFLWHVRKRV&thval=0.0) | 2.04 | inside | 0.73730 |
| **PB2** |  |  |  |
| [RRATAILRKA](https://webs.iiitd.edu.in/raghava/cellppd/pepsearch1.php?seq=RRATAILRKA&thval=0.0) | 3.76 | inside | 0.91542 |
| [GRRATAILRK](https://webs.iiitd.edu.in/raghava/cellppd/pepsearch1.php?seq=GRRATAILRK&thval=0.0) | 3.84 | inside | 0.88161 |
| [RKATRRLIQL](https://webs.iiitd.edu.in/raghava/cellppd/pepsearch1.php?seq=RKATRRLIQL&thval=0.0) | 4.18 | inside | 0.88139 |
| [TAILRKATRR](https://webs.iiitd.edu.in/raghava/cellppd/pepsearch1.php?seq=TAILRKATRR&thval=0.0) | 4.19 | inside | 0.93169 |
| [KNPALRMKWM](https://webs.iiitd.edu.in/raghava/cellppd/pepsearch1.php?seq=KNPALRMKWM&thval=0.0) | 1.89 | inside | 0.74938 |
| [RATAILRKAT](https://webs.iiitd.edu.in/raghava/cellppd/pepsearch1.php?seq=RATAILRKAT&thval=0.0) | 2.52 | inside | 0.88556 |
| [ILRKATRRLI](https://webs.iiitd.edu.in/raghava/cellppd/pepsearch1.php?seq=ILRKATRRLI&thval=0.0) | 3.13 | inside | 0.84137 |
| [AILRKATRRL](https://webs.iiitd.edu.in/raghava/cellppd/pepsearch1.php?seq=AILRKATRRL&thval=0.0) | 3.45 | inside | 0.85681 |
| [SQTATKRIRM](https://webs.iiitd.edu.in/raghava/cellppd/pepsearch1.php?seq=SQTATKRIRM&thval=0.0) | 4.03 | inside | 0.93641 |
| [ATAILRKATR](https://webs.iiitd.edu.in/raghava/cellppd/pepsearch1.php?seq=ATAILRKATR&thval=0.0) | 2.52 | inside | 0.88556 |
| [KATRRLIQLI](https://webs.iiitd.edu.in/raghava/cellppd/pepsearch1.php?seq=KATRRLIQLI&thval=0.0) | 2.2 | inside | 0.79413 |
| **Epitope** | **Protein binding Potential**  **(Boman index)** | **Cellular localization by**  **TMHMM Server** | **Total probability of N-in by TMHMM Server** |
| [RANQRLNPMH](https://webs.iiitd.edu.in/raghava/cellppd/pepsearch1.php?seq=RANQRLNPMH&thval=0.0) | 4.42 | inside | 0.78024 |
| [QSRTREILTK](https://webs.iiitd.edu.in/raghava/cellppd/pepsearch1.php?seq=QSRTREILTK&thval=0.0) | 4.64 | inside | 0.87464 |
| [DRFLRVRDQR](https://webs.iiitd.edu.in/raghava/cellppd/pepsearch1.php?seq=DRFLRVRDQR&thval=0.0) | 7.07 | inside | 0.86249 |
| [LRKATRRLIQ](https://webs.iiitd.edu.in/raghava/cellppd/pepsearch1.php?seq=LRKATRRLIQ&thval=0.0) | 4.18 | inside | 0.88139 |
| [RLIQLIVSGR](https://webs.iiitd.edu.in/raghava/cellppd/pepsearch1.php?seq=RLIQLIVSGR&thval=0.0) | 1.41 | inside | 0.51375 |
| [AIIKKYTSGR](https://webs.iiitd.edu.in/raghava/cellppd/pepsearch1.php?seq=AIIKKYTSGR&thval=0.0) | 1.95 | inside | 0.85230 |
| [VGRRATAILR](https://webs.iiitd.edu.in/raghava/cellppd/pepsearch1.php?seq=VGRRATAILR&thval=0.0) | 2.88 | inside | 0.81212 |
| [VNRANQRLNP](https://webs.iiitd.edu.in/raghava/cellppd/pepsearch1.php?seq=VNRANQRLNP&thval=0.0) | 4.45 | inside | 0.78865 |
| [KKYTSGRQEK](https://webs.iiitd.edu.in/raghava/cellppd/pepsearch1.php?seq=KKYTSGRQEK&thval=0.0) | 4.9 | inside | 0.90829 |
| [SQLTITKEKK](https://webs.iiitd.edu.in/raghava/cellppd/pepsearch1.php?seq=SQLTITKEKK&thval=0.0) | 2.77 | inside | 0.83102 |
| [VRKTRFLPVA](https://webs.iiitd.edu.in/raghava/cellppd/pepsearch1.php?seq=VRKTRFLPVA&thval=0.0) | 2.01 | inside | 0.63281 |
| [DSQTATKRIR](https://webs.iiitd.edu.in/raghava/cellppd/pepsearch1.php?seq=DSQTATKRIR&thval=0.0) | 5.14 | inside | 0.92989 |
| [LIIAARNIVR](https://webs.iiitd.edu.in/raghava/cellppd/pepsearch1.php?seq=LIIAARNIVR&thval=0.0) | 0.91 | inside | 0.74347 |
| [FAAAPPKQSR](https://webs.iiitd.edu.in/raghava/cellppd/pepsearch1.php?seq=FAAAPPKQSR&thval=0.0) | 2.1 | inside | 0.49291 |
| [KRITEMIPER](https://webs.iiitd.edu.in/raghava/cellppd/pepsearch1.php?seq=KRITEMIPER&thval=0.0) | 3.93 | inside | 0.80652 |
| [KRTSGSSVKR](https://webs.iiitd.edu.in/raghava/cellppd/pepsearch1.php?seq=KRTSGSSVKR&thval=0.0) | 4.87 | inside | 0.86391 |
| [VSIDRFLRVR](https://webs.iiitd.edu.in/raghava/cellppd/pepsearch1.php?seq=VSIDRFLRVR&thval=0.0) | 3.59 | inside | 0.67633 |
| [QIIKLLPFAA](https://webs.iiitd.edu.in/raghava/cellppd/pepsearch1.php?seq=QIIKLLPFAA&thval=0.0) | -1.51 | outside | 0.24541 |
| [TATKRIRMAI](https://webs.iiitd.edu.in/raghava/cellppd/pepsearch1.php?seq=TATKRIRMAI&thval=0.0) | 2.47 | inside | 0.92656 |
| [MSMRGVRISK](https://webs.iiitd.edu.in/raghava/cellppd/pepsearch1.php?seq=MSMRGVRISK&thval=0.0) | 2.75 | inside | 0.79831 |
| [NKATKRLTVL](https://webs.iiitd.edu.in/raghava/cellppd/pepsearch1.php?seq=NKATKRLTVL&thval=0.0) | 2.21 | inside | 0.80708 |
| [RNLMSQSRTR](https://webs.iiitd.edu.in/raghava/cellppd/pepsearch1.php?seq=RNLMSQSRTR&thval=0.0) | 5.9 | inside | 0.89570 |
| [AQIIKLLPFA](https://webs.iiitd.edu.in/raghava/cellppd/pepsearch1.php?seq=AQIIKLLPFA&thval=0.0) | -1.51 | outside | 0.24541 |
| [MHQLLRHFQK](https://webs.iiitd.edu.in/raghava/cellppd/pepsearch1.php?seq=MHQLLRHFQK&thval=0.0) | 2.57 | inside | 0.59242 |
| [KATKRLTVLG](https://webs.iiitd.edu.in/raghava/cellppd/pepsearch1.php?seq=KATKRLTVLG&thval=0.0) | 1.45 | inside | 0.70464 |
| [MKRKRDSSIL](https://webs.iiitd.edu.in/raghava/cellppd/pepsearch1.php?seq=MKRKRDSSIL&thval=0.0) | 4.42 | inside | 0.84093 |
| [LVRKTRFLPV](https://webs.iiitd.edu.in/raghava/cellppd/pepsearch1.php?seq=LVRKTRFLPV&thval=0.0) | 1.7 | inside | 0.49337 |
| [RKTRFLPVAG](https://webs.iiitd.edu.in/raghava/cellppd/pepsearch1.php?seq=RKTRFLPVAG&thval=0.0) | 2.32 | inside | 0.56928 |
| [RNIVRRAAVS](https://webs.iiitd.edu.in/raghava/cellppd/pepsearch1.php?seq=RNIVRRAAVS&thval=0.0) | 3.81 | inside | 0.89472 |
| [VLVMKRKRDS](https://webs.iiitd.edu.in/raghava/cellppd/pepsearch1.php?seq=VLVMKRKRDS&thval=0.0) | 3.77 | inside | 0.83937 |
| [LLRHFQKDAK](https://webs.iiitd.edu.in/raghava/cellppd/pepsearch1.php?seq=LLRHFQKDAK&thval=0.0) | 3.03 | inside | 0.62312 |
| [LLPFAAAPPK](https://webs.iiitd.edu.in/raghava/cellppd/pepsearch1.php?seq=LLPFAAAPPK&thval=0.0) | -1.27 | outside | 0.08284 |
| [MERIKELRNL](https://webs.iiitd.edu.in/raghava/cellppd/pepsearch1.php?seq=MERIKELRNL&thval=0.0) | 4.82 | inside | 0.70952 |
| [RRRVDINPGH](https://webs.iiitd.edu.in/raghava/cellppd/pepsearch1.php?seq=RRRVDINPGH&thval=0.0) | 5.48 | inside | 0.79765 |
| [ATKRLTVLGK](https://webs.iiitd.edu.in/raghava/cellppd/pepsearch1.php?seq=ATKRLTVLGK&thval=0.0) | 1.45 | inside | 0.70464 |
| **M2** |  |  |  |
| [KCIYRRFKYG](https://webs.iiitd.edu.in/raghava/cellppd/pepsearch1.php?seq=KCIYRRFKYG&thval=0.0) | 3.11 | inside | 0.89690 |
| [RLFFKCIYRR](https://webs.iiitd.edu.in/raghava/cellppd/pepsearch1.php?seq=RLFFKCIYRR&thval=0.0) | 3.33 | inside | 0.79337 |
| [IYRRFKYGLK](https://webs.iiitd.edu.in/raghava/cellppd/pepsearch1.php?seq=IYRRFKYGLK&thval=0.0) | 2.74 | inside | 0.79523 |
| [YRRFKYGLKG](https://webs.iiitd.edu.in/raghava/cellppd/pepsearch1.php?seq=YRRFKYGLKG&thval=0.0) | 3.14 | inside | 0.73865 |
| [FFKCIYRRFK](https://webs.iiitd.edu.in/raghava/cellppd/pepsearch1.php?seq=FFKCIYRRFK&thval=0.0) | 2.59 | inside | 0.77777 |
| [FKCIYRRFKY](https://webs.iiitd.edu.in/raghava/cellppd/pepsearch1.php?seq=FKCIYRRFKY&thval=0.0) | 2.9 | inside | 0.87795 |
| [CIYRRFKYGL](https://webs.iiitd.edu.in/raghava/cellppd/pepsearch1.php?seq=CIYRRFKYGL&thval=0.0) | 2.06 | inside | 0.75838 |
| **Epitope** | **Protein binding Potential**  **(Boman index)** | **Cellular localization by**  **TMHMM Server** | **Total probability of N-in by TMHMM Server** |
| [WILDRLFFKC](https://webs.iiitd.edu.in/raghava/cellppd/pepsearch1.php?seq=WILDRLFFKC&thval=0.0) | 0.48 | outside | 0.36081 |
| [LWILDRLFFK](https://webs.iiitd.edu.in/raghava/cellppd/pepsearch1.php?seq=LWILDRLFFK&thval=0.0) | 0.12 | outside | 0.20148 |
| **NA** |  |  |  |
| [IKSWRKKILR](https://webs.iiitd.edu.in/raghava/cellppd/pepsearch1.php?seq=IKSWRKKILR&thval=0.0) | 3.28 | inside | 0.89881 |
| [ELIRGRPKEK](https://webs.iiitd.edu.in/raghava/cellppd/pepsearch1.php?seq=ELIRGRPKEK&thval=0.0) | 4.37 | inside | 0.68475 |
| [YKIFKIEKGK](https://webs.iiitd.edu.in/raghava/cellppd/pepsearch1.php?seq=YKIFKIEKGK&thval=0.0) | 1.53 | inside | 0.78175 |
| [ETIKSWRKKI](https://webs.iiitd.edu.in/raghava/cellppd/pepsearch1.php?seq=ETIKSWRKKI&thval=0.0) | 3.21 | inside | 0.90251 |
| [TIKSWRKKIL](https://webs.iiitd.edu.in/raghava/cellppd/pepsearch1.php?seq=TIKSWRKKIL&thval=0.0) | 2.04 | inside | 0.86095 |
| [KIFKIEKGKV](https://webs.iiitd.edu.in/raghava/cellppd/pepsearch1.php?seq=KIFKIEKGKV&thval=0.0) | 1.12 | inside | 0.73914 |
| [LIRGRPKEKT](https://webs.iiitd.edu.in/raghava/cellppd/pepsearch1.php?seq=LIRGRPKEKT&thval=0.0) | 3.95 | inside | 0.76181 |
| [TETIKSWRKK](https://webs.iiitd.edu.in/raghava/cellppd/pepsearch1.php?seq=TETIKSWRKK&thval=0.0) | 3.96 | inside | 0.92072 |
| [KIEKGKVTKS](https://webs.iiitd.edu.in/raghava/cellppd/pepsearch1.php?seq=KIEKGKVTKS&thval=0.0) | 2.5 | inside | 0.83769 |
| [GVWIGRTKSH](https://webs.iiitd.edu.in/raghava/cellppd/pepsearch1.php?seq=GVWIGRTKSH&thval=0.0) | 1.79 | inside | 0.62998 |
| [FWVELIRGRP](https://webs.iiitd.edu.in/raghava/cellppd/pepsearch1.php?seq=FWVELIRGRP&thval=0.0) | 1.65 | outside | 0.33736 |
| [PIRGWAIYSK](https://webs.iiitd.edu.in/raghava/cellppd/pepsearch1.php?seq=PIRGWAIYSK&thval=0.0) | 0.9 | inside | 0.60362 |
| **PB1-F2** |  |  |  |
| [KTRVLKRWRL](https://webs.iiitd.edu.in/raghava/cellppd/pepsearch1.php?seq=KTRVLKRWRL&thval=0.0) | 4.22 | inside | 0.89846 |
| [LKRWRLFSKH](https://webs.iiitd.edu.in/raghava/cellppd/pepsearch1.php?seq=LKRWRLFSKH&thval=0.0) | 3.38 | inside | 0.67596 |
| [VLKRWRLFSK](https://webs.iiitd.edu.in/raghava/cellppd/pepsearch1.php?seq=VLKRWRLFSK&thval=0.0) | 2.51 | inside | 0.65543 |
| [LKTRVLKRWR](https://webs.iiitd.edu.in/raghava/cellppd/pepsearch1.php?seq=LKTRVLKRWR&thval=0.0) | 4.22 | inside | 0.89846 |
| [FLKTRVLKRW](https://webs.iiitd.edu.in/raghava/cellppd/pepsearch1.php?seq=FLKTRVLKRW&thval=0.0) | 2.43 | inside | 0.73690 |
| [RVLKRWRLFS](https://webs.iiitd.edu.in/raghava/cellppd/pepsearch1.php?seq=RVLKRWRLFS&thval=0.0) | 3.45 | inside | 0.68783 |
| [KRWRLFSKHE](https://webs.iiitd.edu.in/raghava/cellppd/pepsearch1.php?seq=KRWRLFSKHE&thval=0.0) | 4.55 | inside | 0.75641 |
| [WKQWLSLRNP](https://webs.iiitd.edu.in/raghava/cellppd/pepsearch1.php?seq=WKQWLSLRNP&thval=0.0) | 2.15 | inside | 0.46841 |
| [TRVLKRWRLF](https://webs.iiitd.edu.in/raghava/cellppd/pepsearch1.php?seq=TRVLKRWRLF&thval=0.0) | 3.36 | inside | 0.76382 |
| [KQWLSLRNPI](https://webs.iiitd.edu.in/raghava/cellppd/pepsearch1.php?seq=KQWLSLRNPI&thval=0.0) | 1.89 | inside | 0.50844 |
| [RNSTRLMGHC](https://webs.iiitd.edu.in/raghava/cellppd/pepsearch1.php?seq=RNSTRLMGHC&thval=0.0) | 3.76 | inside | 0.78931 |
| [GQQTPKLEHR](https://webs.iiitd.edu.in/raghava/cellppd/pepsearch1.php?seq=GQQTPKLEHR&thval=0.0) | 3.97 | inside | 0.63535 |
| [KQIVYWKQWL](https://webs.iiitd.edu.in/raghava/cellppd/pepsearch1.php?seq=KQIVYWKQWL&thval=0.0) | 0.37 | inside | 0.73355 |
| [HRNSTRLMGH](https://webs.iiitd.edu.in/raghava/cellppd/pepsearch1.php?seq=HRNSTRLMGH&thval=0.0) | 4.35 | inside | 0.73726 |
| [VYWKQWLSLR](https://webs.iiitd.edu.in/raghava/cellppd/pepsearch1.php?seq=VYWKQWLSLR&thval=0.0) | 1.1 | inside | 0.56222 |
| **PA** |  |  |  |
| [KIPKTKNMKK](https://webs.iiitd.edu.in/raghava/cellppd/pepsearch1.php?seq=KIPKTKNMKK&thval=0.0) | 2.96 | inside | 0.93088 |
| [RIKTRLFTIR](https://webs.iiitd.edu.in/raghava/cellppd/pepsearch1.php?seq=RIKTRLFTIR&thval=0.0) | 3.77 | inside | 0.87158 |
| [KTTPRPLRLP](https://webs.iiitd.edu.in/raghava/cellppd/pepsearch1.php?seq=KTTPRPLRLP&thval=0.0) | 3.06 | inside | 0.45903 |
| [ESRARIKTRL](https://webs.iiitd.edu.in/raghava/cellppd/pepsearch1.php?seq=ESRARIKTRL&thval=0.0) | 5.14 | inside | 0.88787 |
| [RARIKTRLFT](https://webs.iiitd.edu.in/raghava/cellppd/pepsearch1.php?seq=RARIKTRLFT&thval=0.0) | 4.08 | inside | 0.88423 |
| [FLKTTPRPLR](https://webs.iiitd.edu.in/raghava/cellppd/pepsearch1.php?seq=FLKTTPRPLR&thval=0.0) | 2.77 | inside | 0.46415 |
| [FIIKGRSHLR](https://webs.iiitd.edu.in/raghava/cellppd/pepsearch1.php?seq=FIIKGRSHLR&thval=0.0) | 2.47 | inside | 0.59726 |
| [LKTTPRPLRL](https://webs.iiitd.edu.in/raghava/cellppd/pepsearch1.php?seq=LKTTPRPLRL&thval=0.0) | 2.57 | inside | 0.46026 |
| [PRPLRLPNGP](https://webs.iiitd.edu.in/raghava/cellppd/pepsearch1.php?seq=PRPLRLPNGP&thval=0.0) | 2.57 | outside | 0.14050 |
| [KNMKKTSQLK](https://webs.iiitd.edu.in/raghava/cellppd/pepsearch1.php?seq=KNMKKTSQLK&thval=0.0) | 3.3 | inside | 0.90295 |
| [EKIPKTKNMK](https://webs.iiitd.edu.in/raghava/cellppd/pepsearch1.php?seq=EKIPKTKNMK&thval=0.0) | 3.09 | inside | 0.87930 |
| **Epitope** | **Protein binding Potential**  **(Boman index)** | **Cellular localization by**  **TMHMM Server** | **Total probability of N-in by TMHMM Server** |
| [GKVCRTLLAK](https://webs.iiitd.edu.in/raghava/cellppd/pepsearch1.php?seq=GKVCRTLLAK&thval=0.0) | 1.06 | inside | 0.71146 |
| [SRARIKTRLF](https://webs.iiitd.edu.in/raghava/cellppd/pepsearch1.php?seq=SRARIKTRLF&thval=0.0) | 4.16 | inside | 0.83926 |
| [KKTSQLKWAL](https://webs.iiitd.edu.in/raghava/cellppd/pepsearch1.php?seq=KKTSQLKWAL&thval=0.0) | 1.41 | inside | 0.70648 |
| [KGRSHLRNDT](https://webs.iiitd.edu.in/raghava/cellppd/pepsearch1.php?seq=KGRSHLRNDT&thval=0.0) | 5.55 | inside | 0.79028 |
| [TTPRPLRLPN](https://webs.iiitd.edu.in/raghava/cellppd/pepsearch1.php?seq=TTPRPLRLPN&thval=0.0) | 3.17 | outside | 0.39314 |
| [ARIKTRLFTI](https://webs.iiitd.edu.in/raghava/cellppd/pepsearch1.php?seq=ARIKTRLFTI&thval=0.0) | 2.09 | inside | 0.79852 |
| [KCMRTFFGWK](https://webs.iiitd.edu.in/raghava/cellppd/pepsearch1.php?seq=KCMRTFFGWK&thval=0.0) | 1.57 | inside | 0.73071 |
| [EESRARIKTR](https://webs.iiitd.edu.in/raghava/cellppd/pepsearch1.php?seq=EESRARIKTR&thval=0.0) | 6.31 | inside | 0.92362 |
| [PFLKTTPRPL](https://webs.iiitd.edu.in/raghava/cellppd/pepsearch1.php?seq=PFLKTTPRPL&thval=0.0) | 1.27 | outside | 0.20769 |
| [NGTSKIKMKW](https://webs.iiitd.edu.in/raghava/cellppd/pepsearch1.php?seq=NGTSKIKMKW&thval=0.0) | 1.87 | inside | 0.82704 |
| [TPRPLRLPNG](https://webs.iiitd.edu.in/raghava/cellppd/pepsearch1.php?seq=TPRPLRLPNG&thval=0.0) | 2.82 | outside | 0.26383 |
| [KTKNMKKTSQ](https://webs.iiitd.edu.in/raghava/cellppd/pepsearch1.php?seq=KTKNMKKTSQ&thval=0.0) | 4.05 | inside | 0.95069 |
| [HIYYLEKANK](https://webs.iiitd.edu.in/raghava/cellppd/pepsearch1.php?seq=HIYYLEKANK&thval=0.0) | 1.78 | inside | 0.72221 |
| [KMKWGMEMRR](https://webs.iiitd.edu.in/raghava/cellppd/pepsearch1.php?seq=KMKWGMEMRR&thval=0.0) | 3.74 | inside | 0.89264 |
| [KVCRTLLAKS](https://webs.iiitd.edu.in/raghava/cellppd/pepsearch1.php?seq=KVCRTLLAKS&thval=0.0) | 1.5 | inside | 0.75188 |
| [RFIEIGVTRR](https://webs.iiitd.edu.in/raghava/cellppd/pepsearch1.php?seq=RFIEIGVTRR&thval=0.0) | 3.63 | inside | 0.76945 |
| [TKEGRRKTNL](https://webs.iiitd.edu.in/raghava/cellppd/pepsearch1.php?seq=TKEGRRKTNL&thval=0.0) | 5.36 | inside | 0.90022 |
| [PLRLPNGPPC](https://webs.iiitd.edu.in/raghava/cellppd/pepsearch1.php?seq=PLRLPNGPPC&thval=0.0) | 0.95 | outside | 0.09789 |
| [PKTKNMKKTS](https://webs.iiitd.edu.in/raghava/cellppd/pepsearch1.php?seq=PKTKNMKKTS&thval=0.0) | 3.5 | inside | 0.89318 |
| [HCRATEYIMK](https://webs.iiitd.edu.in/raghava/cellppd/pepsearch1.php?seq=HCRATEYIMK&thval=0.0) | 2.42 | inside | 0.87896 |
| [KEGRRKTNLY](https://webs.iiitd.edu.in/raghava/cellppd/pepsearch1.php?seq=KEGRRKTNLY&thval=0.0) | 5.12 | inside | 0.89004 |
| **M1** |  |  |  |
| [KAVKLYRKLK](https://webs.iiitd.edu.in/raghava/cellppd/pepsearch1.php?seq=KAVKLYRKLK&thval=0.0) | 2.15 | inside | 0.86204 |
| [RGLQRRRFVQ](https://webs.iiitd.edu.in/raghava/cellppd/pepsearch1.php?seq=RGLQRRRFVQ&thval=0.0) | 5.78 | inside | 0.85092 |
| [KLYRKLKREI](https://webs.iiitd.edu.in/raghava/cellppd/pepsearch1.php?seq=KLYRKLKREI&thval=0.0) | 3.86 | inside | 0.86566 |
| [AVKLYRKLKR](https://webs.iiitd.edu.in/raghava/cellppd/pepsearch1.php?seq=AVKLYRKLKR&thval=0.0) | 3.09 | inside | 0.88045 |
| [LQRRRFVQNA](https://webs.iiitd.edu.in/raghava/cellppd/pepsearch1.php?seq=LQRRRFVQNA&thval=0.0) | 4.87 | inside | 0.85643 |
| [RKLKREITFH](https://webs.iiitd.edu.in/raghava/cellppd/pepsearch1.php?seq=RKLKREITFH&thval=0.0) | 4.21 | inside | 0.82102 |
| [GLQRRRFVQN](https://webs.iiitd.edu.in/raghava/cellppd/pepsearch1.php?seq=GLQRRRFVQN&thval=0.0) | 4.96 | inside | 0.80376 |
| [VKLYRKLKRE](https://webs.iiitd.edu.in/raghava/cellppd/pepsearch1.php?seq=VKLYRKLKRE&thval=0.0) | 3.95 | inside | 0.86220 |
| [ERGLQRRRFV](https://webs.iiitd.edu.in/raghava/cellppd/pepsearch1.php?seq=ERGLQRRRFV&thval=0.0) | 5.91 | inside | 0.79638 |
| [WLKTRPILSP](https://webs.iiitd.edu.in/raghava/cellppd/pepsearch1.php?seq=WLKTRPILSP&thval=0.0) | 0.93 | outside | 0.32184 |
| [DKAVKLYRKL](https://webs.iiitd.edu.in/raghava/cellppd/pepsearch1.php?seq=DKAVKLYRKL&thval=0.0) | 2.47 | inside | 0.77915 |
| [MDKAVKLYRK](https://webs.iiitd.edu.in/raghava/cellppd/pepsearch1.php?seq=MDKAVKLYRK&thval=0.0) | 2.73 | inside | 0.86517 |
| **PA-X** |  |  |  |
| [RASCLKCPKK](https://webs.iiitd.edu.in/raghava/cellppd/pepsearch1.php?seq=RASCLKCPKK&thval=0.0) | 2.56 | inside | 0.84454 |
| [EKRQLKKGLK](https://webs.iiitd.edu.in/raghava/cellppd/pepsearch1.php?seq=EKRQLKKGLK&thval=0.0) | 3.86 | inside | 0.81995 |
| [RIKTRLFTIR](https://webs.iiitd.edu.in/raghava/cellppd/pepsearch1.php?seq=RIKTRLFTIR&thval=0.0) | 3.77 | inside | 0.87158 |
| [KRQLKKGLKS](https://webs.iiitd.edu.in/raghava/cellppd/pepsearch1.php?seq=KRQLKKGLKS&thval=0.0) | 3.52 | inside | 0.81885 |
| [ESRARIKTRL](https://webs.iiitd.edu.in/raghava/cellppd/pepsearch1.php?seq=ESRARIKTRL&thval=0.0) | 5.14 | inside | 0.88787 |
| [RARIKTRLFT](https://webs.iiitd.edu.in/raghava/cellppd/pepsearch1.php?seq=RARIKTRLFT&thval=0.0) | 4.08 | inside | 0.88423 |
| [RQLKKGLKSQ](https://webs.iiitd.edu.in/raghava/cellppd/pepsearch1.php?seq=RQLKKGLKSQ&thval=0.0) | 3.52 | inside | 0.78387 |
| [REEKRQLKKG](https://webs.iiitd.edu.in/raghava/cellppd/pepsearch1.php?seq=REEKRQLKKG&thval=0.0) | 5.97 | inside | 0.88863 |
| **Epitope** | **Protein binding Potential**  **(Boman index)** | **Cellular localization by**  **TMHMM Server** | **Total probability of N-in by TMHMM Server** |
| [SRARIKTRLF](https://webs.iiitd.edu.in/raghava/cellppd/pepsearch1.php?seq=SRARIKTRLF&thval=0.0) | 4.16 | inside | 0.83926 |
| [PREEKRQLKK](https://webs.iiitd.edu.in/raghava/cellppd/pepsearch1.php?seq=PREEKRQLKK&thval=0.0) | 6.07 | inside | 0.86913 |
| [LRASCLKCPK](https://webs.iiitd.edu.in/raghava/cellppd/pepsearch1.php?seq=LRASCLKCPK&thval=0.0) | 1.52 | inside | 0.66428 |
| [KVSRRTSPAL](https://webs.iiitd.edu.in/raghava/cellppd/pepsearch1.php?seq=KVSRRTSPAL&thval=0.0) | 3.39 | inside | 0.70009 |
| [ARIKTRLFTI](https://webs.iiitd.edu.in/raghava/cellppd/pepsearch1.php?seq=ARIKTRLFTI&thval=0.0) | 2.09 | inside | 0.79852 |
| [PTKVSRRTSP](https://webs.iiitd.edu.in/raghava/cellppd/pepsearch1.php?seq=PTKVSRRTSP&thval=0.0) | 4.32 | inside | 0.73165 |
| [VSRRTSPALK](https://webs.iiitd.edu.in/raghava/cellppd/pepsearch1.php?seq=VSRRTSPALK&thval=0.0) | 3.39 | inside | 0.70009 |
| [NRTATLRASC](https://webs.iiitd.edu.in/raghava/cellppd/pepsearch1.php?seq=NRTATLRASC&thval=0.0) | 3.51 | inside | 0.87170 |
| [EESRARIKTR](https://webs.iiitd.edu.in/raghava/cellppd/pepsearch1.php?seq=EESRARIKTR&thval=0.0) | 6.31 | inside | 0.92362 |
| [SRRTSPALKI](https://webs.iiitd.edu.in/raghava/cellppd/pepsearch1.php?seq=SRRTSPALKI&thval=0.0) | 3.31 | inside | 0.71731 |
| [SNRTATLRAS](https://webs.iiitd.edu.in/raghava/cellppd/pepsearch1.php?seq=SNRTATLRAS&thval=0.0) | 3.98 | inside | 0.81415 |
| [HIYYLEKANK](https://webs.iiitd.edu.in/raghava/cellppd/pepsearch1.php?seq=HIYYLEKANK&thval=0.0) | 1.78 | inside | 0.72221 |
| [RFIEIGVTRR](https://webs.iiitd.edu.in/raghava/cellppd/pepsearch1.php?seq=RFIEIGVTRR&thval=0.0) | 3.63 | inside | 0.76945 |
| [ASLPTKVSRR](https://webs.iiitd.edu.in/raghava/cellppd/pepsearch1.php?seq=ASLPTKVSRR&thval=0.0) | 3.39 | inside | 0.70009 |
| [TKVSRRTSPA](https://webs.iiitd.edu.in/raghava/cellppd/pepsearch1.php?seq=TKVSRRTSPA&thval=0.0) | 4.14 | inside | 0.83107 |
| [RRTSPALKIL](https://webs.iiitd.edu.in/raghava/cellppd/pepsearch1.php?seq=RRTSPALKIL&thval=0.0) | 2.47 | inside | 0.62785 |
| [SPREEKRQLK](https://webs.iiitd.edu.in/raghava/cellppd/pepsearch1.php?seq=SPREEKRQLK&thval=0.0) | 5.85 | inside | 0.78279 |
| **PB1** |  |  |  |
| [FQRKRRVRDN](https://webs.iiitd.edu.in/raghava/cellppd/pepsearch1.php?seq=FQRKRRVRDN&thval=0.0) | 7.91 | inside | 0.95751 |
| [THFQRKRRVR](https://webs.iiitd.edu.in/raghava/cellppd/pepsearch1.php?seq=THFQRKRRVR&thval=0.0) | 7.09 | inside | 0.95947 |
| [KMITQRTIGK](https://webs.iiitd.edu.in/raghava/cellppd/pepsearch1.php?seq=KMITQRTIGK&thval=0.0) | 2.35 | inside | 0.90498 |
| [RRVRDNMTKK](https://webs.iiitd.edu.in/raghava/cellppd/pepsearch1.php?seq=RRVRDNMTKK&thval=0.0) | 6.73 | inside | 0.97160 |
| [GITTHFQRKR](https://webs.iiitd.edu.in/raghava/cellppd/pepsearch1.php?seq=GITTHFQRKR&thval=0.0) | 4.18 | inside | 0.86882 |
| [HFQRKRRVRD](https://webs.iiitd.edu.in/raghava/cellppd/pepsearch1.php?seq=HFQRKRRVRD&thval=0.0) | 7.71 | inside | 0.94774 |
| [KKIEKIRPLL](https://webs.iiitd.edu.in/raghava/cellppd/pepsearch1.php?seq=KKIEKIRPLL&thval=0.0) | 1.86 | inside | 0.62502 |
| [RLNKRSYLIR](https://webs.iiitd.edu.in/raghava/cellppd/pepsearch1.php?seq=RLNKRSYLIR&thval=0.0) | 4.57 | inside | 0.85468 |
| [DAERGKLKRR](https://webs.iiitd.edu.in/raghava/cellppd/pepsearch1.php?seq=DAERGKLKRR&thval=0.0) | 6.37 | inside | 0.89116 |
| [ERGKLKRRAI](https://webs.iiitd.edu.in/raghava/cellppd/pepsearch1.php?seq=ERGKLKRRAI&thval=0.0) | 5 | inside | 0.88597 |
| [KDAERGKLKR](https://webs.iiitd.edu.in/raghava/cellppd/pepsearch1.php?seq=KDAERGKLKR&thval=0.0) | 5.43 | inside | 0.87429 |
| [QRKRRVRDNM](https://webs.iiitd.edu.in/raghava/cellppd/pepsearch1.php?seq=QRKRRVRDNM&thval=0.0) | 7.97 | inside | 0.97549 |
| [AERGKLKRRA](https://webs.iiitd.edu.in/raghava/cellppd/pepsearch1.php?seq=AERGKLKRRA&thval=0.0) | 5.31 | inside | 0.89598 |
| [KRRVRDNMTK](https://webs.iiitd.edu.in/raghava/cellppd/pepsearch1.php?seq=KRRVRDNMTK&thval=0.0) | 6.74 | inside | 0.97160 |
| [TQRTIGKRKQ](https://webs.iiitd.edu.in/raghava/cellppd/pepsearch1.php?seq=TQRTIGKRKQ&thval=0.0) | 5.13 | inside | 0.95810 |
| [RGKLKRRAIA](https://webs.iiitd.edu.in/raghava/cellppd/pepsearch1.php?seq=RGKLKRRAIA&thval=0.0) | 4.14 | inside | 0.90132 |
| [SWIPKRNRSI](https://webs.iiitd.edu.in/raghava/cellppd/pepsearch1.php?seq=SWIPKRNRSI&thval=0.0) | 3.66 | inside | 0.79326 |
| [MITQRTIGKR](https://webs.iiitd.edu.in/raghava/cellppd/pepsearch1.php?seq=MITQRTIGKR&thval=0.0) | 3.29 | inside | 0.91519 |
| [GKLKRRAIAT](https://webs.iiitd.edu.in/raghava/cellppd/pepsearch1.php?seq=GKLKRRAIAT&thval=0.0) | 2.91 | inside | 0.86812 |
| [KRRAIATPGM](https://webs.iiitd.edu.in/raghava/cellppd/pepsearch1.php?seq=KRRAIATPGM&thval=0.0) | 2.61 | inside | 0.81272 |
| [KKLWEQTRSK](https://webs.iiitd.edu.in/raghava/cellppd/pepsearch1.php?seq=KKLWEQTRSK&thval=0.0) | 4.26 | inside | 0.87412 |
| [ITTHFQRKRR](https://webs.iiitd.edu.in/raghava/cellppd/pepsearch1.php?seq=ITTHFQRKRR&thval=0.0) | 5.77 | inside | 0.94499 |
| [TTHFQRKRRV](https://webs.iiitd.edu.in/raghava/cellppd/pepsearch1.php?seq=TTHFQRKRRV&thval=0.0) | 5.86 | inside | 0.94358 |
| [RKRRVRDNMT](https://webs.iiitd.edu.in/raghava/cellppd/pepsearch1.php?seq=RKRRVRDNMT&thval=0.0) | 7.67 | inside | 0.97557 |
| [KSMKLRTQIP](https://webs.iiitd.edu.in/raghava/cellppd/pepsearch1.php?seq=KSMKLRTQIP&thval=0.0) | 2.53 | inside | 0.76543 |
| **Epitope** | **Protein binding Potential**  **(Boman index)** | **Cellular localization by**  **TMHMM Server** | **Total probability of N-in by TMHMM Server** |
| [TRKKIEKIRP](https://webs.iiitd.edu.in/raghava/cellppd/pepsearch1.php?seq=TRKKIEKIRP&thval=0.0) | 4.6 | inside | 0.92225 |
| [HSWIPKRNRS](https://webs.iiitd.edu.in/raghava/cellppd/pepsearch1.php?seq=HSWIPKRNRS&thval=0.0) | 4.62 | inside | 0.78766 |
| [WIPKRNRSIL](https://webs.iiitd.edu.in/raghava/cellppd/pepsearch1.php?seq=WIPKRNRSIL&thval=0.0) | 2.83 | inside | 0.72104 |
| [STRKKIEKIR](https://webs.iiitd.edu.in/raghava/cellppd/pepsearch1.php?seq=STRKKIEKIR&thval=0.0) | 4.94 | inside | 0.94658 |
| [MGITTHFQRK](https://webs.iiitd.edu.in/raghava/cellppd/pepsearch1.php?seq=MGITTHFQRK&thval=0.0) | 2.46 | inside | 0.78691 |
| [KYFNDSTRKK](https://webs.iiitd.edu.in/raghava/cellppd/pepsearch1.php?seq=KYFNDSTRKK&thval=0.0) | 5 | inside | 0.88887 |
| [NDSTRKKIEK](https://webs.iiitd.edu.in/raghava/cellppd/pepsearch1.php?seq=NDSTRKKIEK&thval=0.0) | 5.47 | inside | 0.91645 |
| [KIEKIRPLLI](https://webs.iiitd.edu.in/raghava/cellppd/pepsearch1.php?seq=KIEKIRPLLI&thval=0.0) | 0.82 | inside | 0.49206 |
| [KLWEQTRSKA](https://webs.iiitd.edu.in/raghava/cellppd/pepsearch1.php?seq=KLWEQTRSKA&thval=0.0) | 3.52 | inside | 0.81513 |
| [KLKRRAIATP](https://webs.iiitd.edu.in/raghava/cellppd/pepsearch1.php?seq=KLKRRAIATP&thval=0.0) | 3 | inside | 0.84591 |
| [GINMSKKKSY](https://webs.iiitd.edu.in/raghava/cellppd/pepsearch1.php?seq=GINMSKKKSY&thval=0.0) | 2.2 | inside | 0.79902 |
| [RQTYDWTLNR](https://webs.iiitd.edu.in/raghava/cellppd/pepsearch1.php?seq=RQTYDWTLNR&thval=0.0) | 4.87 | inside | 0.86636 |
| [QTRRSFEIKK](https://webs.iiitd.edu.in/raghava/cellppd/pepsearch1.php?seq=QTRRSFEIKK&thval=0.0) | 5.13 | inside | 0.89792 |
| [THSWIPKRNR](https://webs.iiitd.edu.in/raghava/cellppd/pepsearch1.php?seq=THSWIPKRNR&thval=0.0) | 4.54 | inside | 0.84650 |
| [DRFYRTCKLH](https://webs.iiitd.edu.in/raghava/cellppd/pepsearch1.php?seq=DRFYRTCKLH&thval=0.0) | 4.22 | inside | 0.82813 |
| [NLGQKRYTKT](https://webs.iiitd.edu.in/raghava/cellppd/pepsearch1.php?seq=NLGQKRYTKT&thval=0.0) | 3.76 | inside | 0.88308 |
| [RGDTQIQTRR](https://webs.iiitd.edu.in/raghava/cellppd/pepsearch1.php?seq=RGDTQIQTRR&thval=0.0) | 6.38 | inside | 0.94119 |
| [RRSFEIKKLW](https://webs.iiitd.edu.in/raghava/cellppd/pepsearch1.php?seq=RRSFEIKKLW&thval=0.0) | 3.59 | inside | 0.75006 |
| [RARIDARIDF](https://webs.iiitd.edu.in/raghava/cellppd/pepsearch1.php?seq=RARIDARIDF&thval=0.0) | 4.57 | inside | 0.82692 |
| [KRSYLIRALT](https://webs.iiitd.edu.in/raghava/cellppd/pepsearch1.php?seq=KRSYLIRALT&thval=0.0) | 2.49 | inside | 0.77066 |
| [KRYTKTTYWW](https://webs.iiitd.edu.in/raghava/cellppd/pepsearch1.php?seq=KRYTKTTYWW&thval=0.0) | 2.93 | inside | 0.91762 |
| [TRRSFEIKKL](https://webs.iiitd.edu.in/raghava/cellppd/pepsearch1.php?seq=TRRSFEIKKL&thval=0.0) | 4.08 | inside | 0.79767 |
| [RKKIEKIRPL](https://webs.iiitd.edu.in/raghava/cellppd/pepsearch1.php?seq=RKKIEKIRPL&thval=0.0) | 3.85 | inside | 0.83931 |
| [GVDRFYRTCK](https://webs.iiitd.edu.in/raghava/cellppd/pepsearch1.php?seq=GVDRFYRTCK&thval=0.0) | 3.75 | inside | 0.83956 |
| [PSSSYRRPVG](https://webs.iiitd.edu.in/raghava/cellppd/pepsearch1.php?seq=PSSSYRRPVG&thval=0.0) | 3.52 | inside | 0.40657 |
| [MVSRARIDAR](https://webs.iiitd.edu.in/raghava/cellppd/pepsearch1.php?seq=MVSRARIDAR&thval=0.0) | 4.19 | inside | 0.88041 |
| [TQIQTRRSFE](https://webs.iiitd.edu.in/raghava/cellppd/pepsearch1.php?seq=TQIQTRRSFE&thval=0.0) | 4.83 | inside | 0.85036 |
| **NEP** |  |  |  |
| [ITQRTIGKRK](https://webs.iiitd.edu.in/raghava/cellppd/pepsearch1.php?seq=ITQRTIGKRK&thval=0.0) | 4.08 | inside | 0.94398 |
| [MSKKKSYINR](https://webs.iiitd.edu.in/raghava/cellppd/pepsearch1.php?seq=MSKKKSYINR&thval=0.0) | 3.78 | inside | 0.92002 |

**Table S5. Immunogenicity, toxicity, allergenicity, half-life and hemolytic potency of the predicted cell-penetrating peptides (CPPs).**

| **Epitope** | **Immunogenicity**  **(IEDB)** | **Toxicity** | **Allergenicity (AllerTop)** | **Allergenicity (AllergenFP)** | **Half-life in E.coli** | **Half-life in**  **mammalian** | **Instability index** |
| --- | --- | --- | --- | --- | --- | --- | --- |
| **NP** |  |  |  |  |  |  |  |
| RSRYWAIRTR | 0.45852 | Non-Toxin | PROBABLE ALLERGEN | PROBABLE ALLERGEN | 2 min | 1 hours | stable |
| RMIKRGINDR | 0.04238 | Non-Toxin | PROBABLE ALLERGEN | PROBABLE NONALLERGEN | 2 min | 1 hours | stable |
| GPIYRRVNGK | 0.19348 | Non-Toxin | PROBABLE NONALLERGEN | PROBABLE NONALLERGEN | >10 hours | 30 hours | stable |
| KGTKVVPRGK | -0.07096 | Non-Toxin | PROBABLE ALLERGEN | PROBABLE ALLERGEN | 3 min | 1.3 hours | stable |
| RRIWRQANNG | 0.23128 | Non-Toxin | PROBABLE ALLERGEN | PROBABLE NONALLERGEN | 2 min | 1 hours | unstable |
| YQRTRALVRT | 0.199 | Non-Toxin | PROBABLE NONALLERGEN | PROBABLE NONALLERGEN | 2 min | 2.8 hours | stable |
| RYWAIRTRSG | 0.27483 | Non-Toxin | PROBABLE ALLERGEN | PROBABLE ALLERGEN | 2 min | 1 hours | stable |
| YRRVNGKWMR | -0.03362 | Non-Toxin | PROBABLE ALLERGEN | PROBABLE ALLERGEN | 2 min | 2.8 hours | unstable |
| GRKTRIAYER | 0.24127 | Non-Toxin | PROBABLE ALLERGEN | PROBABLE ALLERGEN | >10 hours | 30 hours | stable |
| RTRALVRTGM | 0.18685 | Non-Toxin | PROBABLE ALLERGEN | PROBABLE ALLERGEN | 2 min | 1 hours | stable |
| SRYWAIRTRS | 0.50111 | Non-Toxin | PROBABLE NONALLERGEN | PROBABLE ALLERGEN | >10 hours | 1.9 hours | stable |
| HPSAGKDPKK | -0.30581 | Non-Toxin | PROBABLE NONALLERGEN | PROBABLE NONALLERGEN | >10 hours | 3.5 hours | stable |
| KEEIRRIWRQ | 0.60968 | Non-Toxin | PROBABLE NONALLERGEN | PROBABLE ALLERGEN | 3 min | 1.3 hours | unstable |
| EIRRIWRQAN | 0.388 | Non-Toxin | PROBABLE NONALLERGEN | PROBABLE ALLERGEN | >10 hours | 1 hours | unstable |
| RGENGRKTRI | -0.03061 | Non-Toxin | PROBABLE ALLERGEN | PROBABLE NONALLERGEN | 2 min | 1 hours | stable |
| EEIRRIWRQA | 0.45979 | Non-Toxin | PROBABLE ALLERGEN | PROBABLE ALLERGEN | >10 hours | 1 hours | unstable |
| FLARSALILR | 0.03718 | Non-Toxin | PROBABLE ALLERGEN | PROBABLE ALLERGEN | 2 min | 1.1 hours | unstable |
| ESSTLELRSR | 0.00864 | Non-Toxin | PROBABLE NONALLERGEN | PROBABLE NONALLERGEN | >10 hours | 1 hours | unstable |
| IRRIWRQANN | 0.33702 | Non-Toxin | PROBABLE ALLERGEN | PROBABLE ALLERGEN | >10 hours | 20 hours | unstable |
| RNFWRGENGR | 0.45288 | Non-Toxin | PROBABLE NONALLERGEN | PROBABLE NONALLERGEN | 2 min | 1 hours | stable |
| RKTRIAYERM | 0.34364 | Non-Toxin | PROBABLE NONALLERGEN | PROBABLE ALLERGEN | 2 min | 1 hours | stable |
| LSAFDERRNK | 0.33822 | Non-Toxin | PROBABLE NONALLERGEN | PROBABLE NONALLERGEN | 2 min | 5.5 hours | unstable |
| GKWMRELILY | 0.1385 | Non-Toxin | PROBABLE NONALLERGEN | PROBABLE ALLERGEN | >10 hours | 30 hours | stable |
| **Epitope** | **Immunogenicity**  **(IEDB)** | **Toxicity** | **Allergenicity (AllerTop)** | **Allergenicity (AllergenFP)** | **Half-life in E.coli** | **Half-life in**  **mammalian** | **Instability index** |
| TYQRTRALVR | 0.15427 | Non-Toxin | PROBABLE NONALLERGEN | PROBABLE ALLERGEN | >10 hours | 7.2 hours | stable |
| RGINDRNFWR | 0.33082 | Non-Toxin | PROBABLE ALLERGEN | PROBABLE NONALLERGEN | 2 min | 1 hours | stable |
| WRGENGRKTR | 0.02785 | Non-Toxin | PROBABLE NONALLERGEN | PROBABLE NONALLERGEN | 2 min | 2.8 hours | stable |
| PSAGKDPKKT | -0.46004 | Non-Toxin | PROBABLE NONALLERGEN | PROBABLE NONALLERGEN | ? | >20 hours | stable |
| **HA** |  |  |  |  |  |  |  |
| [KNSYVNKKGK](https://webs.iiitd.edu.in/raghava/cellppd/pepsearch1.php?seq=KNSYVNKKGK&thval=0.0) | 0.38056 | Non-Toxin | NON-ALLERGEN | ALLERGEN | 3 min | 1.3 hours | stable |
| [KLCRLKGIAP](https://webs.iiitd.edu.in/raghava/cellppd/pepsearch1.php?seq=KLCRLKGIAP&thval=0.0) | 0.10278 | Non-Toxin | ALLERGEN | NON-ALLERGEN | 3 min | 1.3 hours | stable |
| [PEIAERPKVR](https://webs.iiitd.edu.in/raghava/cellppd/pepsearch1.php?seq=PEIAERPKVR&thval=0.0) | 0.06215 | Non-Toxin | ALLERGEN | NON-ALLERGEN | ? | >20 hours | unstable |
| [KLKNSYVNKK](https://webs.iiitd.edu.in/raghava/cellppd/pepsearch1.php?seq=KLKNSYVNKK&thval=0.0) | -0.01914 | Non-Toxin | NON-ALLERGEN | ALLERGEN | 3 min | 1.3 hours | stable |
| [PKYVRSAKLR](https://webs.iiitd.edu.in/raghava/cellppd/pepsearch1.php?seq=PKYVRSAKLR&thval=0.0) | -0.11489 | Non-Toxin | NON-ALLERGEN | ALLERGEN | ? | >20 hours | unstable |
| [KYVRSAKLRM](https://webs.iiitd.edu.in/raghava/cellppd/pepsearch1.php?seq=KYVRSAKLRM&thval=0.0) | -0.11794 | Non-Toxin | NON-ALLERGEN | ALLERGEN | 3 min | 1.3 hours | unstable |
| [MNYYWTLLKP](https://webs.iiitd.edu.in/raghava/cellppd/pepsearch1.php?seq=MNYYWTLLKP&thval=0.0) | -0.22201 | Non-Toxin | ALLERGEN | NON-ALLERGEN | >10 hours | 30 hours | stable |
| [HNGKLCRLKG](https://webs.iiitd.edu.in/raghava/cellppd/pepsearch1.php?seq=HNGKLCRLKG&thval=0.0) | -0.23964 | Non-Toxin | ALLERGEN | ALLERGEN | >10 hours | 3.5 hours | stable |
| [GKLCRLKGIA](https://webs.iiitd.edu.in/raghava/cellppd/pepsearch1.php?seq=GKLCRLKGIA&thval=0.0) | -0.33381 | Non-Toxin | NON-ALLERGEN | ALLERGEN | >10 hours | 30 hours | stable |
| [GKEFNKLEKR](https://webs.iiitd.edu.in/raghava/cellppd/pepsearch1.php?seq=GKEFNKLEKR&thval=0.0) | -0.35594 | Non-Toxin | NON-ALLERGEN | ALLERGEN | >10 hours | 30 hours | unstable |
| [RRFTPEIAER](https://webs.iiitd.edu.in/raghava/cellppd/pepsearch1.php?seq=RRFTPEIAER&thval=0.0) | -0.38872 | Non-Toxin | NON-ALLERGEN | NON-ALLERGEN | 1 hours | 2 min | unstable |
| **NS1** |  |  |  |  |  |  |  |
| [RLRRDQKSLR](https://webs.iiitd.edu.in/raghava/cellppd/pepsearch1.php?seq=RLRRDQKSLR&thval=0.0) | -0.37142 | Non-Toxin | NON-ALLERGEN | NON-ALLERGEN | 2 min | 1 hours | unstable |
| [RRDQKSLRGR](https://webs.iiitd.edu.in/raghava/cellppd/pepsearch1.php?seq=RRDQKSLRGR&thval=0.0) | -0.42742 | Non-Toxin | NON-ALLERGEN | NON-ALLERGEN | 2 min | 1 hours | unstable |
| [FLDRLRRDQK](https://webs.iiitd.edu.in/raghava/cellppd/pepsearch1.php?seq=FLDRLRRDQK&thval=0.0) | 0.09864 | Non-Toxin | NON-ALLERGEN | NON-ALLERGEN | 2 min | 1.1 hours | unstable |
| [RPPLTPKQKR](https://webs.iiitd.edu.in/raghava/cellppd/pepsearch1.php?seq=RPPLTPKQKR&thval=0.0) | -0.41452 | Non-Toxin | ALLERGEN | ALLERGEN | 2 min | 1 hours | unstable |
| [DAPFLDRLRR](https://webs.iiitd.edu.in/raghava/cellppd/pepsearch1.php?seq=DAPFLDRLRR&thval=0.0) | 0.1946 | Non-Toxin | ALLERGEN | ALLERGEN | >10 hours | 1.1 hours | unstable |
| [DRLRRDQKSL](https://webs.iiitd.edu.in/raghava/cellppd/pepsearch1.php?seq=DRLRRDQKSL&thval=0.0) | -0.26722 | Non-Toxin | NON-ALLERGEN | NON-ALLERGEN | >10 hours | 1.1 hours | unstable |
| [FLWHVRKRVA](https://webs.iiitd.edu.in/raghava/cellppd/pepsearch1.php?seq=FLWHVRKRVA&thval=0.0) | 0.05985 | Non-Toxin | NON-ALLERGEN | ALLERGEN | 2 min | 1.1 hours | unstable |
| [DCFLWHVRKR](https://webs.iiitd.edu.in/raghava/cellppd/pepsearch1.php?seq=DCFLWHVRKR&thval=0.0) | 0.23058 | Non-Toxin | NON-ALLERGEN | ALLERGEN | >10 hours | 1.1 hours | unstable |
| [APFLDRLRRD](https://webs.iiitd.edu.in/raghava/cellppd/pepsearch1.php?seq=APFLDRLRRD&thval=0.0) | 0.16232 | Non-Toxin | ALLERGEN | ALLERGEN | >10 hours | 4.4 hours | unstable |
| **Epitope** | **Immunogenicity**  **(IEDB)** | **Toxicity** | **Allergenicity (AllerTop)** | **Allergenicity (AllergenFP)** | **Half-life in E.coli** | **Half-life in**  **mammalian** | **Instability index** |
| [EWSMLIPKQK](https://webs.iiitd.edu.in/raghava/cellppd/pepsearch1.php?seq=EWSMLIPKQK&thval=0.0) | -0.37172 | Non-Toxin | ALLERGEN | ALLERGEN | >10 hours | 1 hours | stable |
| [LQRFAWRSSN](https://webs.iiitd.edu.in/raghava/cellppd/pepsearch1.php?seq=LQRFAWRSSN&thval=0.0) | 0.20084 | Non-Toxin | NON-ALLERGEN | ALLERGEN | 2 min | 5.5 hours | unstable |
| [GKQIVERILK](https://webs.iiitd.edu.in/raghava/cellppd/pepsearch1.php?seq=GKQIVERILK&thval=0.0) | 0.38858 | Non-Toxin | NON-ALLERGEN | ALLERGEN | 30 hours | >10 hours | stable |
| [CFLWHVRKRV](https://webs.iiitd.edu.in/raghava/cellppd/pepsearch1.php?seq=CFLWHVRKRV&thval=0.0) | 0.18795 | Non-Toxin | NON-ALLERGEN | NONALLERGEN | >10 hours | 1.2 hours | unstable |
| PB2 |  |  |  |  |  |  |  |
| [RRATAILRKA](https://webs.iiitd.edu.in/raghava/cellppd/pepsearch1.php?seq=RRATAILRKA&thval=0.0) | 0.1267 | Non-Toxin | NON-ALLERGEN | ALLERGEN | 2 min | 1 hours | unstable |
| [GRRATAILRK](https://webs.iiitd.edu.in/raghava/cellppd/pepsearch1.php?seq=GRRATAILRK&thval=0.0) | 0.27823 | Non-Toxin | NON-ALLERGEN | ALLERGEN | >10 hours | 30 hours | unstable |
| [RKATRRLIQL](https://webs.iiitd.edu.in/raghava/cellppd/pepsearch1.php?seq=RKATRRLIQL&thval=0.0) | 0.18676 | Non-Toxin | NON-ALLERGEN | NON-ALLERGEN | 2 min | 1 hours | unstable |
| [TAILRKATRR](https://webs.iiitd.edu.in/raghava/cellppd/pepsearch1.php?seq=TAILRKATRR&thval=0.0) | -0.02773 | Non-Toxin | NON-ALLERGEN | NON-ALLERGEN | >10 hours | 7.2 hours | unstable |
| [KNPALRMKWM](https://webs.iiitd.edu.in/raghava/cellppd/pepsearch1.php?seq=KNPALRMKWM&thval=0.0) | -0.14251 | Non-Toxin | ALLERGEN | ALLERGEN | 3 min | 1.3 hours | unstable |
| [RATAILRKAT](https://webs.iiitd.edu.in/raghava/cellppd/pepsearch1.php?seq=RATAILRKAT&thval=0.0) | 0.06035 | Non-Toxin | NON-ALLERGEN | NON-ALLERGEN | 2 min | 1 hours | unstable |
| [ILRKATRRLI](https://webs.iiitd.edu.in/raghava/cellppd/pepsearch1.php?seq=ILRKATRRLI&thval=0.0) | -0.03838 | Non-Toxin | NON-ALLERGEN | ALLERGEN | >10 hours | 20 hours | unstable |
| [AILRKATRRL](https://webs.iiitd.edu.in/raghava/cellppd/pepsearch1.php?seq=AILRKATRRL&thval=0.0) | -0.01296 | Non-Toxin | NON-ALLERGEN | ALLERGEN | >10 hours | 4.4 hours | unstable |
| [SQTATKRIRM](https://webs.iiitd.edu.in/raghava/cellppd/pepsearch1.php?seq=SQTATKRIRM&thval=0.0) | 0.07105 | Non-Toxin | NON-ALLERGEN | ALLERGEN | >10 hours | 1.9 hours | unstable |
| [ATAILRKATR](https://webs.iiitd.edu.in/raghava/cellppd/pepsearch1.php?seq=ATAILRKATR&thval=0.0) | 0.03892 | Non-Toxin | NON-ALLERGEN | NON-ALLERGEN | >10 hours | 4.4 hours | unstable |
| [KATRRLIQLI](https://webs.iiitd.edu.in/raghava/cellppd/pepsearch1.php?seq=KATRRLIQLI&thval=0.0) | 0.12532 | Non-Toxin | NON-ALLERGEN | ALLERGEN | 3 min | 1.3 hours | unstable |
| [RANQRLNPMH](https://webs.iiitd.edu.in/raghava/cellppd/pepsearch1.php?seq=RANQRLNPMH&thval=0.0) | -0.19711 | Non-Toxin | NON-ALLERGEN | ALLERGEN | 2 min | 1 hours | unstable |
| [QSRTREILTK](https://webs.iiitd.edu.in/raghava/cellppd/pepsearch1.php?seq=QSRTREILTK&thval=0.0) | 0.34236 | Non-Toxin | ALLERGEN | NON-ALLERGEN | 10 hours | 0.8 hours | unstable |
| [DRFLRVRDQR](https://webs.iiitd.edu.in/raghava/cellppd/pepsearch1.php?seq=DRFLRVRDQR&thval=0.0) | 0.1172 | Non-Toxin | NON-ALLERGEN | ALLERGEN | >10 hours | 1.1 hours | stable |
| [LRKATRRLIQ](https://webs.iiitd.edu.in/raghava/cellppd/pepsearch1.php?seq=LRKATRRLIQ&thval=0.0) | 0.17469 | Non-Toxin | NON-ALLERGEN | NON-ALLERGEN | 2 min | 5.5 hours | unstable |
| [RLIQLIVSGR](https://webs.iiitd.edu.in/raghava/cellppd/pepsearch1.php?seq=RLIQLIVSGR&thval=0.0) | -0.03552 | Non-Toxin | ALLERGEN | NON-ALLERGEN | 2 min | 1 hours | stable |
| [AIIKKYTSGR](https://webs.iiitd.edu.in/raghava/cellppd/pepsearch1.php?seq=AIIKKYTSGR&thval=0.0) | -0.47068 | Non-Toxin | NON-ALLERGEN | ALLERGEN | >10 hours | 4.4 hours | stable |
| [VGRRATAILR](https://webs.iiitd.edu.in/raghava/cellppd/pepsearch1.php?seq=VGRRATAILR&thval=0.0) | 0.28745 | Non-Toxin | NON-ALLERGEN | NON-ALLERGEN | >10 hours | 100 hours | unstable |
| [VNRANQRLNP](https://webs.iiitd.edu.in/raghava/cellppd/pepsearch1.php?seq=VNRANQRLNP&thval=0.0) | -0.02735 | Non-Toxin | ALLERGEN | ALLERGEN | >10 hours | 100 hours | stable |
| [KKYTSGRQEK](https://webs.iiitd.edu.in/raghava/cellppd/pepsearch1.php?seq=KKYTSGRQEK&thval=0.0) | -0.08078 | Non-Toxin | NON-ALLERGEN | NON-ALLERGEN | >10 hours | 100 hours | stable |
| [SQLTITKEKK](https://webs.iiitd.edu.in/raghava/cellppd/pepsearch1.php?seq=SQLTITKEKK&thval=0.0) | -0.04164 | Non-Toxin | NON-ALLERGEN | NON-ALLERGEN | >10 hours | 1.9 hours | stable |
| [VRKTRFLPVA](https://webs.iiitd.edu.in/raghava/cellppd/pepsearch1.php?seq=VRKTRFLPVA&thval=0.0) | 0.13778 | Non-Toxin | ALLERGEN | ALLERGEN | >10 hours | 100 hours | unstable |
| [DSQTATKRIR](https://webs.iiitd.edu.in/raghava/cellppd/pepsearch1.php?seq=DSQTATKRIR&thval=0.0) | -0.0042 | Non-Toxin | NON-ALLERGEN | ALLERGEN | >10 hours | 1.1 hours | unstable |
| [LIIAARNIVR](https://webs.iiitd.edu.in/raghava/cellppd/pepsearch1.php?seq=LIIAARNIVR&thval=0.0) | 0.30142 | Non-Toxin | ALLERGEN | ALLERGEN | 2 min | 5.5 hours | unstable |
| [FAAAPPKQSR](https://webs.iiitd.edu.in/raghava/cellppd/pepsearch1.php?seq=FAAAPPKQSR&thval=0.0) | -0.36695 | Non-Toxin | NON-ALLERGEN | NON-ALLERGEN | 2 min | 1.1 hours | unstable |
| **Epitope** | **Immunogenicity**  **(IEDB)** | **Toxicity** | **Allergenicity (AllerTop)** | **Allergenicity (AllergenFP)** | **Half-life in E.coli** | **Half-life in**  **mammalian** | **Instability index** |
| [KRITEMIPER](https://webs.iiitd.edu.in/raghava/cellppd/pepsearch1.php?seq=KRITEMIPER&thval=0.0) | 0.18318 | Non-Toxin | ALLERGEN | ALLERGEN | 3 min | 1.3 hours | unstable |
| [KRTSGSSVKR](https://webs.iiitd.edu.in/raghava/cellppd/pepsearch1.php?seq=KRTSGSSVKR&thval=0.0) | -0.52886 | Non-Toxin | ALLERGEN | ALLERGEN | 3 min | 1.3 hours | unstable |
| [VSIDRFLRVR](https://webs.iiitd.edu.in/raghava/cellppd/pepsearch1.php?seq=VSIDRFLRVR&thval=0.0) | 0.28728 | Non-Toxin | NON-ALLERGEN | NON-ALLERGEN | >10 hours | 100 hours | stable |
| [QIIKLLPFAA](https://webs.iiitd.edu.in/raghava/cellppd/pepsearch1.php?seq=QIIKLLPFAA&thval=0.0) | -0.08418 | Non-Toxin | ALLERGEN | ALLERGEN | 10 hours | 0.8 hours | stable |
| [TATKRIRMAI](https://webs.iiitd.edu.in/raghava/cellppd/pepsearch1.php?seq=TATKRIRMAI&thval=0.0) | -0.10102 | Non-Toxin | NON-ALLERGEN | ALLERGEN | >10 hours | 7.2 hours | unstable |
| [MSMRGVRISK](https://webs.iiitd.edu.in/raghava/cellppd/pepsearch1.php?seq=MSMRGVRISK&thval=0.0) | 0.13266 | Non-Toxin | ALLERGEN | NON-ALLERGEN | >10 hours | 30 hours | stable |
| [NKATKRLTVL](https://webs.iiitd.edu.in/raghava/cellppd/pepsearch1.php?seq=NKATKRLTVL&thval=0.0) | -0.0614 | Non-Toxin | NON-ALLERGEN | ALLERGEN | >10 hours | 1.4 hours | unstable |
| [RNLMSQSRTR](https://webs.iiitd.edu.in/raghava/cellppd/pepsearch1.php?seq=RNLMSQSRTR&thval=0.0) | -0.54357 | Non-Toxin | NON-ALLERGEN | NON-ALLERGEN | 2 min | 1 hours | unstable |
| [AQIIKLLPFA](https://webs.iiitd.edu.in/raghava/cellppd/pepsearch1.php?seq=AQIIKLLPFA&thval=0.0) | 0.00492 | Non-Toxin | NON-ALLERGEN | ALLERGEN | >10 hours | 4.4 hours | stable |
| [MHQLLRHFQK](https://webs.iiitd.edu.in/raghava/cellppd/pepsearch1.php?seq=MHQLLRHFQK&thval=0.0) | 0.05241 | Non-Toxin | ALLERGEN | NON-ALLERGEN | >10 hours | 30 hours | unstable |
| [KATKRLTVLG](https://webs.iiitd.edu.in/raghava/cellppd/pepsearch1.php?seq=KATKRLTVLG&thval=0.0) | -0.0999 | Non-Toxin | NON-ALLERGEN | ALLERGEN | 3 min | 1.3 hours | unstable |
| [MKRKRDSSIL](https://webs.iiitd.edu.in/raghava/cellppd/pepsearch1.php?seq=MKRKRDSSIL&thval=0.0) | -0.34579 | Non-Toxin | NON-ALLERGEN | NON-ALLERGEN | >10 hours | 30 hours | unstable |
| [LVRKTRFLPV](https://webs.iiitd.edu.in/raghava/cellppd/pepsearch1.php?seq=LVRKTRFLPV&thval=0.0) | -0.01764 | Non-Toxin | ALLERGEN | NON-ALLERGEN | 2 min | 5.5 hours | unstable |
| [RKTRFLPVAG](https://webs.iiitd.edu.in/raghava/cellppd/pepsearch1.php?seq=RKTRFLPVAG&thval=0.0) | 0.21514 | Non-Toxin | ALLERGEN | ALLERGEN | 2 min | 1 hours | unstable |
| [RNIVRRAAVS](https://webs.iiitd.edu.in/raghava/cellppd/pepsearch1.php?seq=RNIVRRAAVS&thval=0.0) | 0.27951 | Non-Toxin | NON-ALLERGEN | NON-ALLERGEN | 2 min | 1 hours | unstable |
| [VLVMKRKRDS](https://webs.iiitd.edu.in/raghava/cellppd/pepsearch1.php?seq=VLVMKRKRDS&thval=0.0) | -0.46926 | Non-Toxin | NON-ALLERGEN | NON-ALLERGEN | >10 hours | 100 hours | unstable |
| [LLRHFQKDAK](https://webs.iiitd.edu.in/raghava/cellppd/pepsearch1.php?seq=LLRHFQKDAK&thval=0.0) | -0.11087 | Non-Toxin | ALLERGEN | ALLERGEN | 2 min | 5.5 hours | stable |
| [LLPFAAAPPK](https://webs.iiitd.edu.in/raghava/cellppd/pepsearch1.php?seq=LLPFAAAPPK&thval=0.0) | 0.21139 | Non-Toxin | NON-ALLERGEN | NON-ALLERGEN | 2 min | 5.5 hours | unstable |
| [MERIKELRNL](https://webs.iiitd.edu.in/raghava/cellppd/pepsearch1.php?seq=MERIKELRNL&thval=0.0) | 0.06768 | Non-Toxin | ALLERGEN | NON-ALLERGEN | >10 hours | 30 hours | stable |
| [RRRVDINPGH](https://webs.iiitd.edu.in/raghava/cellppd/pepsearch1.php?seq=RRRVDINPGH&thval=0.0) | 0.21389 | Non-Toxin | NON-ALLERGEN | ALLERGEN | 2 min | 1 hours | unstable |
| [ATKRLTVLGK](https://webs.iiitd.edu.in/raghava/cellppd/pepsearch1.php?seq=ATKRLTVLGK&thval=0.0) | 0.05838 | Non-Toxin | NON-ALLERGEN | NON-ALLERGEN | >10 hours | 4.4 hours | stable |
| **M2** |  |  |  |  |  |  |  |
| [KCIYRRFKYG](https://webs.iiitd.edu.in/raghava/cellppd/pepsearch1.php?seq=KCIYRRFKYG&thval=0.0) | 0.06632 | Non-Toxin | NON-ALLERGEN | NON-ALLERGEN | 3 min | 1.3 hours | stable |
| [RLFFKCIYRR](https://webs.iiitd.edu.in/raghava/cellppd/pepsearch1.php?seq=RLFFKCIYRR&thval=0.0) | 0.0457 | Non-Toxin | ALLERGEN | ALLERGEN | 2 min | 1 hours | stable |
| [IYRRFKYGLK](https://webs.iiitd.edu.in/raghava/cellppd/pepsearch1.php?seq=IYRRFKYGLK&thval=0.0) | -0.00848 | Non-Toxin | ALLERGEN | ALLERGEN | >10 hours | 20 hours | stable |
| [YRRFKYGLKG](https://webs.iiitd.edu.in/raghava/cellppd/pepsearch1.php?seq=YRRFKYGLKG&thval=0.0) | -0.18246 | Non-Toxin | NON-ALLERGEN | NON-ALLERGEN | 2 min | 2.8 hours | stable |
| [FFKCIYRRFK](https://webs.iiitd.edu.in/raghava/cellppd/pepsearch1.php?seq=FFKCIYRRFK&thval=0.0) | 0.16255 | Non-Toxin | ALLERGEN | ALLERGEN | 2 min | 1.1 hours | stable |
| [FKCIYRRFKY](https://webs.iiitd.edu.in/raghava/cellppd/pepsearch1.php?seq=FKCIYRRFKY&thval=0.0) | 0.18474 | Non-Toxin | ALLERGEN | NON-ALLERGEN | 2 min | 1.1 hours | stable |
| [CIYRRFKYGL](https://webs.iiitd.edu.in/raghava/cellppd/pepsearch1.php?seq=CIYRRFKYGL&thval=0.0) | 0.02896 | Non-Toxin | NON-ALLERGEN | NON-ALLERGEN | >10 hours | 1.2 hours | stable |
| [WILDRLFFKC](https://webs.iiitd.edu.in/raghava/cellppd/pepsearch1.php?seq=WILDRLFFKC&thval=0.0) | 0.14132 | Non-Toxin | ALLERGEN | NON-ALLERGEN | 2 min | 2.8 hours | stable |
| **Epitope** | **Immunogenicity**  **(IEDB)** | **Toxicity** | **Allergenicity (AllerTop)** | **Allergenicity (AllergenFP)** | **Half-life in E.coli** | **Half-life in**  **mammalian** | **Instability index** |
| [LWILDRLFFK](https://webs.iiitd.edu.in/raghava/cellppd/pepsearch1.php?seq=LWILDRLFFK&thval=0.0) | 0.2608 | Non-Toxin | ALLERGEN | ALLERGEN | 2 min | 5.5 hours | stable |
| **NA** |  |  |  |  |  |  |  |
| [IKSWRKKILR](https://webs.iiitd.edu.in/raghava/cellppd/pepsearch1.php?seq=IKSWRKKILR&thval=0.0) | -0.08757 | Non-Toxin | NON-ALLERGEN | NON-ALLERGEN | >10 hours | 20 hours | stable |
| [ELIRGRPKEK](https://webs.iiitd.edu.in/raghava/cellppd/pepsearch1.php?seq=ELIRGRPKEK&thval=0.0) | 0.04474 | Non-Toxin | NON-ALLERGEN | NON-ALLERGEN | >10 hours | 1 hours | stable |
| [YKIFKIEKGK](https://webs.iiitd.edu.in/raghava/cellppd/pepsearch1.php?seq=YKIFKIEKGK&thval=0.0) | 0.01265 | Non-Toxin | NON-ALLERGEN | ALLERGEN | 2 min | 2.8 hours | stable |
| [ETIKSWRKKI](https://webs.iiitd.edu.in/raghava/cellppd/pepsearch1.php?seq=ETIKSWRKKI&thval=0.0) | -0.37848 | Non-Toxin | NON-ALLERGEN | NON-ALLERGEN | >10 hours | 1 hours | stable |
| [TIKSWRKKIL](https://webs.iiitd.edu.in/raghava/cellppd/pepsearch1.php?seq=TIKSWRKKIL&thval=0.0) | -0.27761 | Non-Toxin | NON-ALLERGEN | NON-ALLERGEN | >10 hours | 7.2 hours | stable |
| [KIFKIEKGKV](https://webs.iiitd.edu.in/raghava/cellppd/pepsearch1.php?seq=KIFKIEKGKV&thval=0.0) | -0.2523 | Non-Toxin | NON-ALLERGEN | ALLERGEN | 3 min | 1.3 hours | stable |
| [LIRGRPKEKT](https://webs.iiitd.edu.in/raghava/cellppd/pepsearch1.php?seq=LIRGRPKEKT&thval=0.0) | -0.154 | Non-Toxin | NON-ALLERGEN | NON-ALLERGEN | 2 min | 5.5 hours | stable |
| [TETIKSWRKK](https://webs.iiitd.edu.in/raghava/cellppd/pepsearch1.php?seq=TETIKSWRKK&thval=0.0) | -0.09839 | Non-Toxin | NON-ALLERGEN | ALLERGEN | >10 hours | 7.2 hours | stable |
| [KIEKGKVTKS](https://webs.iiitd.edu.in/raghava/cellppd/pepsearch1.php?seq=KIEKGKVTKS&thval=0.0) | -0.41588 | Non-Toxin | NON-ALLERGEN | NON-ALLERGEN | 3 min | 1.3 hours | stable |
| [GVWIGRTKSH](https://webs.iiitd.edu.in/raghava/cellppd/pepsearch1.php?seq=GVWIGRTKSH&thval=0.0) | 0.0471 | Non-Toxin | ALLERGEN | NON-ALLERGEN | >10 hours | 30 hours | stable |
| [FWVELIRGRP](https://webs.iiitd.edu.in/raghava/cellppd/pepsearch1.php?seq=FWVELIRGRP&thval=0.0) | 0.34051 | Non-Toxin | ALLERGEN | NON-ALLERGEN | 2 min | 1.1 hours | stable |
| [PIRGWAIYSK](https://webs.iiitd.edu.in/raghava/cellppd/pepsearch1.php?seq=PIRGWAIYSK&thval=0.0) | 0.3302 | Non-Toxin | NON-ALLERGEN | NON-ALLERGEN | ? | >20 hours | stable |
| **PB1-F2** |  |  |  |  |  |  |  |
| [KTRVLKRWRL](https://webs.iiitd.edu.in/raghava/cellppd/pepsearch1.php?seq=KTRVLKRWRL&thval=0.0) | 0.10344 | Non-Toxin | NON-ALLERGEN | NON-ALLERGEN | 3 min | 1.3 hours | unstable |
| [LKRWRLFSKH](https://webs.iiitd.edu.in/raghava/cellppd/pepsearch1.php?seq=LKRWRLFSKH&thval=0.0) | 0.12387 | Non-Toxin | ALLERGEN | ALLERGEN | 2 min | 5.5 hours | unstable |
| [VLKRWRLFSK](https://webs.iiitd.edu.in/raghava/cellppd/pepsearch1.php?seq=VLKRWRLFSK&thval=0.0) | 0.23988 | Non-Toxin | NON-ALLERGEN | ALLERGEN | >10 hours | 100 hours | unstable |
| [LKTRVLKRWR](https://webs.iiitd.edu.in/raghava/cellppd/pepsearch1.php?seq=LKTRVLKRWR&thval=0.0) | 0.06418 | Non-Toxin | NON-ALLERGEN | ALLERGEN | 2 min | 5.5 hours | unstable |
| [FLKTRVLKRW](https://webs.iiitd.edu.in/raghava/cellppd/pepsearch1.php?seq=FLKTRVLKRW&thval=0.0) | -0.10254 | Non-Toxin | NON-ALLERGEN | ALLERGEN | 2 min | 1.1 hours | unstable |
| [RVLKRWRLFS](https://webs.iiitd.edu.in/raghava/cellppd/pepsearch1.php?seq=RVLKRWRLFS&thval=0.0) | 0.15326 | Non-Toxin | NON-ALLERGEN | ALLERGEN | 2 min | 1 hours | unstable |
| [KRWRLFSKHE](https://webs.iiitd.edu.in/raghava/cellppd/pepsearch1.php?seq=KRWRLFSKHE&thval=0.0) | -0.09165 | Non-Toxin | ALLERGEN | NON-ALLERGEN | 3 min | 1.3 hours | unstable |
| [WKQWLSLRNP](https://webs.iiitd.edu.in/raghava/cellppd/pepsearch1.php?seq=WKQWLSLRNP&thval=0.0) | 0.04285 | Non-Toxin | ALLERGEN | NON-ALLERGEN | 2 min | 2.8 hours | unstable |
| [TRVLKRWRLF](https://webs.iiitd.edu.in/raghava/cellppd/pepsearch1.php?seq=TRVLKRWRLF&thval=0.0) | 0.08835 | Non-Toxin | NON-ALLERGEN | ALLERGEN | >10 hours | 7.2 hours | unstable |
| [KQWLSLRNPI](https://webs.iiitd.edu.in/raghava/cellppd/pepsearch1.php?seq=KQWLSLRNPI&thval=0.0) | -0.07438 | Non-Toxin | ALLERGEN | NON-ALLERGEN | 3 min | 1.3 hours | unstable |
| [RNSTRLMGHC](https://webs.iiitd.edu.in/raghava/cellppd/pepsearch1.php?seq=RNSTRLMGHC&thval=0.0) | -0.09284 | Non-Toxin | NON-ALLERGEN | NON-ALLERGEN | 2 min | 1 hours | stable |
| [GQQTPKLEHR](https://webs.iiitd.edu.in/raghava/cellppd/pepsearch1.php?seq=GQQTPKLEHR&thval=0.0) | -0.12638 | Non-Toxin | NON-ALLERGEN | NON-ALLERGEN | >10 hours | 30 hours | stable |
| [KQIVYWKQWL](https://webs.iiitd.edu.in/raghava/cellppd/pepsearch1.php?seq=KQIVYWKQWL&thval=0.0) | 0.1255 | Non-Toxin | NON-ALLERGEN | ALLERGEN | 3 min | 1.3 hours | unstable |
| [HRNSTRLMGH](https://webs.iiitd.edu.in/raghava/cellppd/pepsearch1.php?seq=HRNSTRLMGH&thval=0.0) | -0.21921 | Non-Toxin | NON-ALLERGEN | NON-ALLERGEN | >10 hours | 3.5 hours | stable |
| [VYWKQWLSLR](https://webs.iiitd.edu.in/raghava/cellppd/pepsearch1.php?seq=VYWKQWLSLR&thval=0.0) | -0.19874 | Non-Toxin | ALLERGEN | NON-ALLERGEN | >10 hours | 100 hours | unstable |
| **Epitope** | **Immunogenicity**  **(IEDB)** | **Toxicity** | **Allergenicity (AllerTop)** | **Allergenicity (AllergenFP)** | **Half-life in E.coli** | **Half-life in**  **mammalian** | **Instability index** |
| **PA** |  |  |  |  |  |  |  |
| [KIPKTKNMKK](https://webs.iiitd.edu.in/raghava/cellppd/pepsearch1.php?seq=KIPKTKNMKK&thval=0.0) | -0.67309 | Non-Toxin | NON-ALLERGEN | ALLERGEN | 3 min | 1.3 hours | stable |
| [RIKTRLFTIR](https://webs.iiitd.edu.in/raghava/cellppd/pepsearch1.php?seq=RIKTRLFTIR&thval=0.0) | 0.22938 | Non-Toxin | NON-ALLERGEN | NON-ALLERGEN | 2 min | 1 hours | stable |
| [KTTPRPLRLP](https://webs.iiitd.edu.in/raghava/cellppd/pepsearch1.php?seq=KTTPRPLRLP&thval=0.0) | 0.0678 | Non-Toxin | NON-ALLERGEN | ALLERGEN | 3 min | 1.3 hours | unstable |
| [ESRARIKTRL](https://webs.iiitd.edu.in/raghava/cellppd/pepsearch1.php?seq=ESRARIKTRL&thval=0.0) | 0.09617 | Non-Toxin | NON-ALLERGEN | ALLERGEN | >10 hours | 1 hours | stable |
| [RARIKTRLFT](https://webs.iiitd.edu.in/raghava/cellppd/pepsearch1.php?seq=RARIKTRLFT&thval=0.0) | 0.08628 | Non-Toxin | NON-ALLERGEN | ALLERGEN | 2 min | 1 hours | stable |
| [FLKTTPRPLR](https://webs.iiitd.edu.in/raghava/cellppd/pepsearch1.php?seq=FLKTTPRPLR&thval=0.0) | 0.02894 | Non-Toxin | NON-ALLERGEN | NON-ALLERGEN | 2 min | 1.1 hours | stable |
| [FIIKGRSHLR](https://webs.iiitd.edu.in/raghava/cellppd/pepsearch1.php?seq=FIIKGRSHLR&thval=0.0) | -0.22531 | Non-Toxin | NON-ALLERGEN | NON-ALLERGEN | 2 min | 1.1 hours | unstable |
| [LKTTPRPLRL](https://webs.iiitd.edu.in/raghava/cellppd/pepsearch1.php?seq=LKTTPRPLRL&thval=0.0) | 0.1017 | Non-Toxin | NON-ALLERGEN | NON-ALLERGEN | 2 min | 5.5 hours | stable |
| [PRPLRLPNGP](https://webs.iiitd.edu.in/raghava/cellppd/pepsearch1.php?seq=PRPLRLPNGP&thval=0.0) | 0.02874 | Non-Toxin | ALLERGEN | ALLERGEN | ? | >20 hours | unstable |
| [KNMKKTSQLK](https://webs.iiitd.edu.in/raghava/cellppd/pepsearch1.php?seq=KNMKKTSQLK&thval=0.0) | -0.70617 | Non-Toxin | NON-ALLERGEN | NON-ALLERGEN | 3 min | 1.3 hours | stable |
| [EKIPKTKNMK](https://webs.iiitd.edu.in/raghava/cellppd/pepsearch1.php?seq=EKIPKTKNMK&thval=0.0) | -0.45122 | Non-Toxin | NON-ALLERGEN | NON-ALLERGEN | >10 hours | 1 hours | stable |
| [GKVCRTLLAK](https://webs.iiitd.edu.in/raghava/cellppd/pepsearch1.php?seq=GKVCRTLLAK&thval=0.0) | 0.05041 | Non-Toxin | ALLERGEN | NON-ALLERGEN | >10 hours | 30 hours | stable |
| [SRARIKTRLF](https://webs.iiitd.edu.in/raghava/cellppd/pepsearch1.php?seq=SRARIKTRLF&thval=0.0) | 0.05812 | Non-Toxin | NON-ALLERGEN | ALLERGEN | >10 hours | 1.9 hours | stable |
| [KKTSQLKWAL](https://webs.iiitd.edu.in/raghava/cellppd/pepsearch1.php?seq=KKTSQLKWAL&thval=0.0) | -0.27067 | Non-Toxin | NON-ALLERGEN | ALLERGEN | 3 min | 1.3 hours | stable |
| [KGRSHLRNDT](https://webs.iiitd.edu.in/raghava/cellppd/pepsearch1.php?seq=KGRSHLRNDT&thval=0.0) | -0.07275 | Non-Toxin | ALLERGEN | NON-ALLERGEN | 3 min | 1.3 hours | unstable |
| [TTPRPLRLPN](https://webs.iiitd.edu.in/raghava/cellppd/pepsearch1.php?seq=TTPRPLRLPN&thval=0.0) | 0.05976 | Non-Toxin | NON-ALLERGEN | ALLERGEN | >10 hours | 7.2 hours | unstable |
| [ARIKTRLFTI](https://webs.iiitd.edu.in/raghava/cellppd/pepsearch1.php?seq=ARIKTRLFTI&thval=0.0) | 0.02544 | Non-Toxin | NON-ALLERGEN | NON-ALLERGEN | >10 hours | 4.4 hours | stable |
| [KCMRTFFGWK](https://webs.iiitd.edu.in/raghava/cellppd/pepsearch1.php?seq=KCMRTFFGWK&thval=0.0) | 0.4151 | Non-Toxin | NON-ALLERGEN | ALLERGEN | 3 min | 1.3 hours | unstable |
| [EESRARIKTR](https://webs.iiitd.edu.in/raghava/cellppd/pepsearch1.php?seq=EESRARIKTR&thval=0.0) | 0.05284 | Non-Toxin | NON-ALLERGEN | NON-ALLERGEN | >10 hours | 1 hours | unstable |
| [PFLKTTPRPL](https://webs.iiitd.edu.in/raghava/cellppd/pepsearch1.php?seq=PFLKTTPRPL&thval=0.0) | -0.11824 | Non-Toxin | ALLERGEN | NON-ALLERGEN | ? | >20 hours | stable |
| [NGTSKIKMKW](https://webs.iiitd.edu.in/raghava/cellppd/pepsearch1.php?seq=NGTSKIKMKW&thval=0.0) | -0.71147 | Non-Toxin | ALLERGEN | NON-ALLERGEN | >10 hours | 1.4 hours | stable |
| [TPRPLRLPNG](https://webs.iiitd.edu.in/raghava/cellppd/pepsearch1.php?seq=TPRPLRLPNG&thval=0.0) | 0.02166 | Non-Toxin | ALLERGEN | NON-ALLERGEN | >10 hours | 7.2 hours | unstable |
| [KTKNMKKTSQ](https://webs.iiitd.edu.in/raghava/cellppd/pepsearch1.php?seq=KTKNMKKTSQ&thval=0.0) | -0.72441 | Non-Toxin | NON-ALLERGEN | NON-ALLERGEN | 3 min | 1.3 hours | stable |
| [HIYYLEKANK](https://webs.iiitd.edu.in/raghava/cellppd/pepsearch1.php?seq=HIYYLEKANK&thval=0.0) | -0.09198 | Non-Toxin | NON-ALLERGEN | NON-ALLERGEN | >10 hours | 3.5 hours | unstable |
| [KMKWGMEMRR](https://webs.iiitd.edu.in/raghava/cellppd/pepsearch1.php?seq=KMKWGMEMRR&thval=0.0) | -0.00882 | Non-Toxin | ALLERGEN | ALLERGEN | 3 min | 1.3 hours | unstable |
| [KVCRTLLAKS](https://webs.iiitd.edu.in/raghava/cellppd/pepsearch1.php?seq=KVCRTLLAKS&thval=0.0) | -0.04184 | Non-Toxin | ALLERGEN | ALLERGEN | 3 min | 1.3 hours | stable |
| [RFIEIGVTRR](https://webs.iiitd.edu.in/raghava/cellppd/pepsearch1.php?seq=RFIEIGVTRR&thval=0.0) | 0.40841 | Non-Toxin | ALLERGEN | NON-ALLERGEN | 2 min | 1 hours | unstable |
| [TKEGRRKTNL](https://webs.iiitd.edu.in/raghava/cellppd/pepsearch1.php?seq=TKEGRRKTNL&thval=0.0) | -0.00662 | Non-Toxin | ALLERGEN | NON-ALLERGEN | >10 hours | 7.2 hours | unstable |
| [PLRLPNGPPC](https://webs.iiitd.edu.in/raghava/cellppd/pepsearch1.php?seq=PLRLPNGPPC&thval=0.0) | 0.0046 | Non-Toxin | ALLERGEN | NON-ALLERGEN | ? | >20 hours | unstable |
| [PKTKNMKKTS](https://webs.iiitd.edu.in/raghava/cellppd/pepsearch1.php?seq=PKTKNMKKTS&thval=0.0) | -0.74402 | Non-Toxin | NON-ALLERGEN | ALLERGEN | ? | >20 hours | stable |
| **Epitope** | **Immunogenicity**  **(IEDB)** | **Toxicity** | **Allergenicity (AllerTop)** | **Allergenicity (AllergenFP)** | **Half-life in E.coli** | **Half-life in**  **mammalian** | **Instability index** |
| [HCRATEYIMK](https://webs.iiitd.edu.in/raghava/cellppd/pepsearch1.php?seq=HCRATEYIMK&thval=0.0) | 0.19771 | Non-Toxin | ALLERGEN | ALLERGEN | >10 hours | 3.5 hours | stable |
| [KEGRRKTNLY](https://webs.iiitd.edu.in/raghava/cellppd/pepsearch1.php?seq=KEGRRKTNLY&thval=0.0) | -0.07192 | Non-Toxin | NON-ALLERGEN | ALLERGEN | 3 min | 1.3 hours | unstable |
| **M1** |  |  |  |  |  |  |  |
| [KAVKLYRKLK](https://webs.iiitd.edu.in/raghava/cellppd/pepsearch1.php?seq=KAVKLYRKLK&thval=0.0) | -0.35776 | Non-Toxin | NON-ALLERGEN | NON-ALLERGEN | 3 min | 1.3 hours | stable |
| [RGLQRRRFVQ](https://webs.iiitd.edu.in/raghava/cellppd/pepsearch1.php?seq=RGLQRRRFVQ&thval=0.0) | 0.15228 | Non-Toxin | NON-ALLERGEN | NON-ALLERGEN | 2 min | 1 hours | unstable |
| [KLYRKLKREI](https://webs.iiitd.edu.in/raghava/cellppd/pepsearch1.php?seq=KLYRKLKREI&thval=0.0) | -0.27074 | Non-Toxin | NON-ALLERGEN | NON-ALLERGEN | 3 min | 1.3 hours | stable |
| [AVKLYRKLKR](https://webs.iiitd.edu.in/raghava/cellppd/pepsearch1.php?seq=AVKLYRKLKR&thval=0.0) | -0.37272 | Non-Toxin | NON-ALLERGEN | NON-ALLERGEN | >10 hours | 4.4 hours | stable |
| [LQRRRFVQNA](https://webs.iiitd.edu.in/raghava/cellppd/pepsearch1.php?seq=LQRRRFVQNA&thval=0.0) | 0.1706 | Non-Toxin | ALLERGEN | ALLERGEN | 2 min | 5.5 hours | unstable |
| [RKLKREITFH](https://webs.iiitd.edu.in/raghava/cellppd/pepsearch1.php?seq=RKLKREITFH&thval=0.0) | 0.15374 | Non-Toxin | NON-ALLERGEN | ALLERGEN | 2 min | 1 hours | unstable |
| [GLQRRRFVQN](https://webs.iiitd.edu.in/raghava/cellppd/pepsearch1.php?seq=GLQRRRFVQN&thval=0.0) | 0.19264 | Non-Toxin | NON-ALLERGEN | ALLERGEN | >10 hours | 30 hours | unstable |
| [VKLYRKLKRE](https://webs.iiitd.edu.in/raghava/cellppd/pepsearch1.php?seq=VKLYRKLKRE&thval=0.0) | -0.32912 | Non-Toxin | NON-ALLERGEN | NON-ALLERGEN | >10 hours | 100 hours | stable |
| [ERGLQRRRFV](https://webs.iiitd.edu.in/raghava/cellppd/pepsearch1.php?seq=ERGLQRRRFV&thval=0.0) | 0.09824 | Non-Toxin | NON-ALLERGEN | NON-ALLERGEN | >10 hours | 1 hours | unstable |
| [WLKTRPILSP](https://webs.iiitd.edu.in/raghava/cellppd/pepsearch1.php?seq=WLKTRPILSP&thval=0.0) | 0.02792 | Non-Toxin | NON-ALLERGEN | ALLERGEN | 2 min | 2.8 hours | unstable |
| [DKAVKLYRKL](https://webs.iiitd.edu.in/raghava/cellppd/pepsearch1.php?seq=DKAVKLYRKL&thval=0.0) | -0.25236 | Non-Toxin | NON-ALLERGEN | ALLERGEN | >10 hours | 1.1 hours | stable |
| [MDKAVKLYRK](https://webs.iiitd.edu.in/raghava/cellppd/pepsearch1.php?seq=MDKAVKLYRK&thval=0.0) | -0.18375 | Non-Toxin | NON-ALLERGEN | NON-ALLERGEN | >10 hours | 30 hours | stable |
| **PA-X** |  |  |  |  |  |  |  |
| [RASCLKCPKK](https://webs.iiitd.edu.in/raghava/cellppd/pepsearch1.php?seq=RASCLKCPKK&thval=0.0) | -0.51486 | Non-Toxin | NON-ALLERGEN | NON-ALLERGEN | 2 min | 1 hours | unstable |
| [EKRQLKKGLK](https://webs.iiitd.edu.in/raghava/cellppd/pepsearch1.php?seq=EKRQLKKGLK&thval=0.0) | -0.50144 | Non-Toxin | NON-ALLERGEN | NON-ALLERGEN | >10 hours | 1 hours | stable |
| [RIKTRLFTIR](https://webs.iiitd.edu.in/raghava/cellppd/pepsearch1.php?seq=RIKTRLFTIR&thval=0.0) | 0.22938 | Non-Toxin | NON-ALLERGEN | NON-ALLERGEN | 2 min | 1 hours | stable |
| [KRQLKKGLKS](https://webs.iiitd.edu.in/raghava/cellppd/pepsearch1.php?seq=KRQLKKGLKS&thval=0.0) | -0.57222 | Non-Toxin | NON-ALLERGEN | NON-ALLERGEN | 3 min | 1.3 hours | stable |
| [ESRARIKTRL](https://webs.iiitd.edu.in/raghava/cellppd/pepsearch1.php?seq=ESRARIKTRL&thval=0.0) | 0.09617 | Non-Toxin | NON-ALLERGEN | ALLERGEN | >10 hours | 1 hours | stable |
| [RARIKTRLFT](https://webs.iiitd.edu.in/raghava/cellppd/pepsearch1.php?seq=RARIKTRLFT&thval=0.0) | 0.08628 | Non-Toxin | NON-ALLERGEN | ALLERGEN | 2 min | 1 hours | stable |
| [RQLKKGLKSQ](https://webs.iiitd.edu.in/raghava/cellppd/pepsearch1.php?seq=RQLKKGLKSQ&thval=0.0) | -0.6867 | Non-Toxin | NON-ALLERGEN | ALLERGEN | 2 min | 1 hours | stable |
| [REEKRQLKKG](https://webs.iiitd.edu.in/raghava/cellppd/pepsearch1.php?seq=REEKRQLKKG&thval=0.0) | -0.56534 | Non-Toxin | NON-ALLERGEN | NON-ALLERGEN | 2 min | 1 hours | unstable |
| [SRARIKTRLF](https://webs.iiitd.edu.in/raghava/cellppd/pepsearch1.php?seq=SRARIKTRLF&thval=0.0) | 0.05812 | Non-Toxin | NON-ALLERGEN | ALLERGEN | >10 hours | 1.9 hours | stable |
| [PREEKRQLKK](https://webs.iiitd.edu.in/raghava/cellppd/pepsearch1.php?seq=PREEKRQLKK&thval=0.0) | -0.27075 | Non-Toxin | NON-ALLERGEN | NON-ALLERGEN | ? | >20 hours | unstable |
| [LRASCLKCPK](https://webs.iiitd.edu.in/raghava/cellppd/pepsearch1.php?seq=LRASCLKCPK&thval=0.0) | -0.47205 | Non-Toxin | NON-ALLERGEN | NON-ALLERGEN | 2 min | 5.5 hours | unstable |
| [KVSRRTSPAL](https://webs.iiitd.edu.in/raghava/cellppd/pepsearch1.php?seq=KVSRRTSPAL&thval=0.0) | -0.05565 | Non-Toxin | NON-ALLERGEN | ALLERGEN | 3 min | 1.3 hours | unstable |
| [ARIKTRLFTI](https://webs.iiitd.edu.in/raghava/cellppd/pepsearch1.php?seq=ARIKTRLFTI&thval=0.0) | 0.02544 | Non-Toxin | NON-ALLERGEN | NON-ALLERGEN | >10 hours | 4.4 hours | stable |
| [PTKVSRRTSP](https://webs.iiitd.edu.in/raghava/cellppd/pepsearch1.php?seq=PTKVSRRTSP&thval=0.0) | -0.15434 | Non-Toxin | ALLERGEN | ALLERGEN | ? | >20 hours | unstable |
| [VSRRTSPALK](https://webs.iiitd.edu.in/raghava/cellppd/pepsearch1.php?seq=VSRRTSPALK&thval=0.0) | -0.03832 | Non-Toxin | ALLERGEN | NON-ALLERGEN | >10 hours | 100 hours | unstable |
| **Epitope** | **Immunogenicity**  **(IEDB)** | **Toxicity** | **Allergenicity (AllerTop)** | **Allergenicity (AllergenFP)** | **Half-life in E.coli** | **Half-life in**  **mammalian** | **Instability index** |
| [NRTATLRASC](https://webs.iiitd.edu.in/raghava/cellppd/pepsearch1.php?seq=NRTATLRASC&thval=0.0) | 0.06405 | Non-Toxin | ALLERGEN | ALLERGEN | >10 hours | 1.4 hours | unstable |
| [EESRARIKTR](https://webs.iiitd.edu.in/raghava/cellppd/pepsearch1.php?seq=EESRARIKTR&thval=0.0) | 0.05284 | Non-Toxin | NON-ALLERGEN | NON-ALLERGEN | >10 hours | 1 hours | unstable |
| [SRRTSPALKI](https://webs.iiitd.edu.in/raghava/cellppd/pepsearch1.php?seq=SRRTSPALKI&thval=0.0) | -0.21457 | Non-Toxin | NON-ALLERGEN | NON-ALLERGEN | >10 hours | 1.9 hours | unstable |
| [SNRTATLRAS](https://webs.iiitd.edu.in/raghava/cellppd/pepsearch1.php?seq=SNRTATLRAS&thval=0.0) | 0.18786 | Non-Toxin | ALLERGEN | ALLERGEN | >10 hours | 1.9 hours | stable |
| [HIYYLEKANK](https://webs.iiitd.edu.in/raghava/cellppd/pepsearch1.php?seq=HIYYLEKANK&thval=0.0) | -0.09198 | Non-Toxin | NON-ALLERGEN | NON-ALLERGEN | >10 hours | 3.5 hours | unstable |
| [RFIEIGVTRR](https://webs.iiitd.edu.in/raghava/cellppd/pepsearch1.php?seq=RFIEIGVTRR&thval=0.0) | 0.40841 | Non-Toxin | ALLERGEN | NON-ALLERGEN | 2 min | 1 hours | unstable |
| [ASLPTKVSRR](https://webs.iiitd.edu.in/raghava/cellppd/pepsearch1.php?seq=ASLPTKVSRR&thval=0.0) | -0.25748 | Non-Toxin | NON-ALLERGEN | ALLERGEN | >10 hours | 4.4 hours | unstable |
| [TKVSRRTSPA](https://webs.iiitd.edu.in/raghava/cellppd/pepsearch1.php?seq=TKVSRRTSPA&thval=0.0) | -0.16183 | Non-Toxin | ALLERGEN | NON-ALLERGEN | >10 hours | 7.2 hours | unstable |
| [RRTSPALKIL](https://webs.iiitd.edu.in/raghava/cellppd/pepsearch1.php?seq=RRTSPALKIL&thval=0.0) | -0.24125 | Non-Toxin | ALLERGEN | ALLERGEN | 2 min | 1 hours | unstable |
| [SPREEKRQLK](https://webs.iiitd.edu.in/raghava/cellppd/pepsearch1.php?seq=SPREEKRQLK&thval=0.0) | -0.05047 | Non-Toxin | NON-ALLERGEN | NON-ALLERGEN | >10 hours | 1.9 hours | unstable |
| PB1 |  |  |  |  |  |  |  |
| [FQRKRRVRDN](https://webs.iiitd.edu.in/raghava/cellppd/pepsearch1.php?seq=FQRKRRVRDN&thval=0.0) | -0.0039 | Non-Toxin | NON-ALLERGEN | NON-ALLERGEN | 2 min | 1.1 hours | unstable |
| [THFQRKRRVR](https://webs.iiitd.edu.in/raghava/cellppd/pepsearch1.php?seq=THFQRKRRVR&thval=0.0) | -0.12164 | Non-Toxin | NON-ALLERGEN | NON-ALLERGEN | >10 hours | 7.2 hours | unstable |
| [KMITQRTIGK](https://webs.iiitd.edu.in/raghava/cellppd/pepsearch1.php?seq=KMITQRTIGK&thval=0.0) | 0.18852 | Non-Toxin | ALLERGEN | NON-ALLERGEN | 3 min | 1.3 hours | stable |
| [RRVRDNMTKK](https://webs.iiitd.edu.in/raghava/cellppd/pepsearch1.php?seq=RRVRDNMTKK&thval=0.0) | -0.17776 | Non-Toxin | NON-ALLERGEN | NON-ALLERGEN | 2 min | 1 hours | unstable |
| [GITTHFQRKR](https://webs.iiitd.edu.in/raghava/cellppd/pepsearch1.php?seq=GITTHFQRKR&thval=0.0) | 0.0058 | Non-Toxin | NON-ALLERGEN | NON-ALLERGEN | >10 hours | 30 hours | stable |
| [HFQRKRRVRD](https://webs.iiitd.edu.in/raghava/cellppd/pepsearch1.php?seq=HFQRKRRVRD&thval=0.0) | -0.03132 | Non-Toxin | NON-ALLERGEN | ALLERGEN | >10 hours | 3.5 hours | unstable |
| [KKIEKIRPLL](https://webs.iiitd.edu.in/raghava/cellppd/pepsearch1.php?seq=KKIEKIRPLL&thval=0.0) | 0.09643 | Non-Toxin | ALLERGEN | ALLERGEN | 3 min | 1.3 hours | unstable |
| [RLNKRSYLIR](https://webs.iiitd.edu.in/raghava/cellppd/pepsearch1.php?seq=RLNKRSYLIR&thval=0.0) | -0.26488 | Non-Toxin | NON-ALLERGEN | NON-ALLERGEN | 2 min | 1 hours | unstable |
| [DAERGKLKRR](https://webs.iiitd.edu.in/raghava/cellppd/pepsearch1.php?seq=DAERGKLKRR&thval=0.0) | -0.25462 | Non-Toxin | NON-ALLERGEN | ALLERGEN | >10 hours | 1.1 hours | unstable |
| [ERGKLKRRAI](https://webs.iiitd.edu.in/raghava/cellppd/pepsearch1.php?seq=ERGKLKRRAI&thval=0.0) | -0.31154 | Non-Toxin | NON-ALLERGEN | ALLERGEN | >10 hours | 1 hours | unstable |
| [KDAERGKLKR](https://webs.iiitd.edu.in/raghava/cellppd/pepsearch1.php?seq=KDAERGKLKR&thval=0.0) | -0.14151 | Non-Toxin | NON-ALLERGEN | ALLERGEN | 3 min | 1.3 hours | stable |
| [QRKRRVRDNM](https://webs.iiitd.edu.in/raghava/cellppd/pepsearch1.php?seq=QRKRRVRDNM&thval=0.0) | 0.13634 | Non-Toxin | NON-ALLERGEN | NON-ALLERGEN | 10 hours | 0.8 hours | unstable |
| [AERGKLKRRA](https://webs.iiitd.edu.in/raghava/cellppd/pepsearch1.php?seq=AERGKLKRRA&thval=0.0) | -0.29898 | Non-Toxin | NON-ALLERGEN | ALLERGEN | >10 hours | 4.4 hours | unstable |
| [KRRVRDNMTK](https://webs.iiitd.edu.in/raghava/cellppd/pepsearch1.php?seq=KRRVRDNMTK&thval=0.0) | -0.00127 | Non-Toxin | NON-ALLERGEN | ALLERGEN | 3 min | 1.3 hours | unstable |
| [TQRTIGKRKQ](https://webs.iiitd.edu.in/raghava/cellppd/pepsearch1.php?seq=TQRTIGKRKQ&thval=0.0) | -0.06686 | Non-Toxin | NON-ALLERGEN | NON-ALLERGEN | >10 hours | 7.2 hours | unstable |
| [RGKLKRRAIA](https://webs.iiitd.edu.in/raghava/cellppd/pepsearch1.php?seq=RGKLKRRAIA&thval=0.0) | -0.08126 | Non-Toxin | NON-ALLERGEN | ALLERGEN | 2 min | 1 hours | unstable |
| [SWIPKRNRSI](https://webs.iiitd.edu.in/raghava/cellppd/pepsearch1.php?seq=SWIPKRNRSI&thval=0.0) | -0.18663 | Non-Toxin | ALLERGEN | ALLERGEN | >10 hours | 1.9 hours | unstable |
| [MITQRTIGKR](https://webs.iiitd.edu.in/raghava/cellppd/pepsearch1.php?seq=MITQRTIGKR&thval=0.0) | 0.01212 | Non-Toxin | NON-ALLERGEN | ALLERGEN | >10 hours | 30 hours | stable |
| [GKLKRRAIAT](https://webs.iiitd.edu.in/raghava/cellppd/pepsearch1.php?seq=GKLKRRAIAT&thval=0.0) | 0.05221 | Non-Toxin | NON-ALLERGEN | ALLERGEN | >10 hours | 30 hours | unstable |
| [KRRAIATPGM](https://webs.iiitd.edu.in/raghava/cellppd/pepsearch1.php?seq=KRRAIATPGM&thval=0.0) | 0.27085 | Non-Toxin | NON-ALLERGEN | NON-ALLERGEN | 3 min | 1.3 hours | unstable |
| **Epitope** | **Immunogenicity**  **(IEDB)** | **Toxicity** | **Allergenicity (AllerTop)** | **Allergenicity (AllergenFP)** | **Half-life in E.coli** | **Half-life in**  **mammalian** | **Instability index** |
| [KKLWEQTRSK](https://webs.iiitd.edu.in/raghava/cellppd/pepsearch1.php?seq=KKLWEQTRSK&thval=0.0) | 0.18755 | Non-Toxin | NON-ALLERGEN | NON-ALLERGEN | 3 min | 1.3 hours | unstable |
| [ITTHFQRKRR](https://webs.iiitd.edu.in/raghava/cellppd/pepsearch1.php?seq=ITTHFQRKRR&thval=0.0) | -0.05669 | Non-Toxin | NON-ALLERGEN | NON-ALLERGEN | >10 hours | 20 hours | unstable |
| [TTHFQRKRRV](https://webs.iiitd.edu.in/raghava/cellppd/pepsearch1.php?seq=TTHFQRKRRV&thval=0.0) | -0.06318 | Non-Toxin | NON-ALLERGEN | ALLERGEN | >10 hours | 7.2 hours | unstable |
| [RKRRVRDNMT](https://webs.iiitd.edu.in/raghava/cellppd/pepsearch1.php?seq=RKRRVRDNMT&thval=0.0) | 0.0723 | Non-Toxin | NON-ALLERGEN | NON-ALLERGEN | 2 min | 1 hours | unstable |
| [KSMKLRTQIP](https://webs.iiitd.edu.in/raghava/cellppd/pepsearch1.php?seq=KSMKLRTQIP&thval=0.0) | -0.21786 | Non-Toxin | ALLERGEN | ALLERGEN | 3 min | 1.3 hours | stable |
| [TRKKIEKIRP](https://webs.iiitd.edu.in/raghava/cellppd/pepsearch1.php?seq=TRKKIEKIRP&thval=0.0) | -0.12034 | Non-Toxin | NON-ALLERGEN | NON-ALLERGEN | >10 hours | 7.2 hours | unstable |
| [HSWIPKRNRS](https://webs.iiitd.edu.in/raghava/cellppd/pepsearch1.php?seq=HSWIPKRNRS&thval=0.0) | 0.05852 | Non-Toxin | NON-ALLERGEN | ALLERGEN | >10 hours | 3.5 hours | unstable |
| [WIPKRNRSIL](https://webs.iiitd.edu.in/raghava/cellppd/pepsearch1.php?seq=WIPKRNRSIL&thval=0.0) | -0.18964 | Non-Toxin | ALLERGEN | ALLERGEN | 2 min | 2.8 hours | unstable |
| [STRKKIEKIR](https://webs.iiitd.edu.in/raghava/cellppd/pepsearch1.php?seq=STRKKIEKIR&thval=0.0) | -0.29059 | Non-Toxin | NON-ALLERGEN | NON-ALLERGEN | >10 hours | 1.9 hours | stable |
| [MGITTHFQRK](https://webs.iiitd.edu.in/raghava/cellppd/pepsearch1.php?seq=MGITTHFQRK&thval=0.0) | 0.19424 | Non-Toxin | NON-ALLERGEN | NON-ALLERGEN | >10 hours | 30 hours | stable |
| [KYFNDSTRKK](https://webs.iiitd.edu.in/raghava/cellppd/pepsearch1.php?seq=KYFNDSTRKK&thval=0.0) | -0.15379 | Non-Toxin | NON-ALLERGEN | NON-ALLERGEN | 3 min | 1.3 hours | stable |
| [NDSTRKKIEK](https://webs.iiitd.edu.in/raghava/cellppd/pepsearch1.php?seq=NDSTRKKIEK&thval=0.0) | -0.20642 | Non-Toxin | NON-ALLERGEN | ALLERGEN | >10 hours | 1.4 hours | unstable |
| [KIEKIRPLLI](https://webs.iiitd.edu.in/raghava/cellppd/pepsearch1.php?seq=KIEKIRPLLI&thval=0.0) | -0.03078 | Non-Toxin | NON-ALLERGEN | NON-ALLERGEN | 3 min | 1.3 hours | unstable |
| [KLWEQTRSKA](https://webs.iiitd.edu.in/raghava/cellppd/pepsearch1.php?seq=KLWEQTRSKA&thval=0.0) | -0.11925 | Non-Toxin | NON-ALLERGEN | NON-ALLERGEN | 3 min | 1.3 hours | unstable |
| [KLKRRAIATP](https://webs.iiitd.edu.in/raghava/cellppd/pepsearch1.php?seq=KLKRRAIATP&thval=0.0) | 0.25156 | Non-Toxin | NON-ALLERGEN | NON-ALLERGEN | 3 min | 1.3 hours | unstable |
| [GINMSKKKSY](https://webs.iiitd.edu.in/raghava/cellppd/pepsearch1.php?seq=GINMSKKKSY&thval=0.0) | -1.03156 | Non-Toxin | NON-ALLERGEN | NON-ALLERGEN | >10 hours | 30 hours | unstable |
| [ITQRTIGKRK](https://webs.iiitd.edu.in/raghava/cellppd/pepsearch1.php?seq=ITQRTIGKRK&thval=0.0) | 0.06202 | Non-Toxin | NON-ALLERGEN | NON-ALLERGEN | >10 hours | 20 hours | stable |
| [MSKKKSYINR](https://webs.iiitd.edu.in/raghava/cellppd/pepsearch1.php?seq=MSKKKSYINR&thval=0.0) | -0.55304 | Non-Toxin | NON-ALLERGEN | NON-ALLERGEN | >10 hours | 30 hours | unstable |
| [RQTYDWTLNR](https://webs.iiitd.edu.in/raghava/cellppd/pepsearch1.php?seq=RQTYDWTLNR&thval=0.0) | 0.26958 | Non-Toxin | NON-ALLERGEN | ALLERGEN | 2 min | 1 hours | stable |
| [QTRRSFEIKK](https://webs.iiitd.edu.in/raghava/cellppd/pepsearch1.php?seq=QTRRSFEIKK&thval=0.0) | 0.10235 | Non-Toxin | NON-ALLERGEN | ALLERGEN | 10 hours | 0.8 hours | unstable |
| [THSWIPKRNR](https://webs.iiitd.edu.in/raghava/cellppd/pepsearch1.php?seq=THSWIPKRNR&thval=0.0) | 0.12489 | Non-Toxin | ALLERGEN | ALLERGEN | >10 hours | 7.2 hours | unstable |
| [DRFYRTCKLH](https://webs.iiitd.edu.in/raghava/cellppd/pepsearch1.php?seq=DRFYRTCKLH&thval=0.0) | -0.11675 | Non-Toxin | NON-ALLERGEN | NON-ALLERGEN | >10 hours | 1.1 hours | stable |
| [NLGQKRYTKT](https://webs.iiitd.edu.in/raghava/cellppd/pepsearch1.php?seq=NLGQKRYTKT&thval=0.0) | -0.36188 | Non-Toxin | NON-ALLERGEN | ALLERGEN | >10 hours | 1.4 hours | stable |
| [RGDTQIQTRR](https://webs.iiitd.edu.in/raghava/cellppd/pepsearch1.php?seq=RGDTQIQTRR&thval=0.0) | 0.01702 | Non-Toxin | NON-ALLERGEN | NON-ALLERGEN | 2 min | 1 hours | stable |
| [RRSFEIKKLW](https://webs.iiitd.edu.in/raghava/cellppd/pepsearch1.php?seq=RRSFEIKKLW&thval=0.0) | -0.10028 | Non-Toxin | ALLERGEN | NON-ALLERGEN | 2 min | 1 hours | unstable |
| [RARIDARIDF](https://webs.iiitd.edu.in/raghava/cellppd/pepsearch1.php?seq=RARIDARIDF&thval=0.0) | 0.38442 | Non-Toxin | NON-ALLERGEN | NON-ALLERGEN | 2 min | 1 hours | stable |
| [KRSYLIRALT](https://webs.iiitd.edu.in/raghava/cellppd/pepsearch1.php?seq=KRSYLIRALT&thval=0.0) | 0.13664 | Non-Toxin | NON-ALLERGEN | NON-ALLERGEN | 3 min | 1.3 hours | unstable |
| [KRYTKTTYWW](https://webs.iiitd.edu.in/raghava/cellppd/pepsearch1.php?seq=KRYTKTTYWW&thval=0.0) | 0.0285 | Non-Toxin | NON-ALLERGEN | NON-ALLERGEN | 3 min | 1.3 hours | stable |
| [TRRSFEIKKL](https://webs.iiitd.edu.in/raghava/cellppd/pepsearch1.php?seq=TRRSFEIKKL&thval=0.0) | -0.12089 | Non-Toxin | ALLERGEN | NON-ALLERGEN | >10 hours | 7.2 hours | unstable |
| [RKKIEKIRPL](https://webs.iiitd.edu.in/raghava/cellppd/pepsearch1.php?seq=RKKIEKIRPL&thval=0.0) | 0.1139 | Non-Toxin | NON-ALLERGEN | ALLERGEN | 2 min | 1 hours | unstable |
| [GVDRFYRTCK](https://webs.iiitd.edu.in/raghava/cellppd/pepsearch1.php?seq=GVDRFYRTCK&thval=0.0) | 0.21966 | Non-Toxin | ALLERGEN | NON-ALLERGEN | >10 hours | 30 hours | stable |
| **Epitope** | **Immunogenicity**  **(IEDB)** | **Toxicity** | **Allergenicity (Aller Top)** | **Allergenicity (AllergenFP)** | **Half-life in E.coli** | **Half-life in**  **mammalian** | **Instability index** |
| [PSSSYRRPVG](https://webs.iiitd.edu.in/raghava/cellppd/pepsearch1.php?seq=PSSSYRRPVG&thval=0.0) | -0.10989 | Non-Toxin | ALLERGEN | ALLERGEN | ? | >20 hours | unstable |
| [MVSRARIDAR](https://webs.iiitd.edu.in/raghava/cellppd/pepsearch1.php?seq=MVSRARIDAR&thval=0.0) | 0.25374 | Non-Toxin | ALLERGEN | NON-ALLERGEN | >10 hours | 30 hours | stable |
| [TQIQTRRSFE](https://webs.iiitd.edu.in/raghava/cellppd/pepsearch1.php?seq=TQIQTRRSFE&thval=0.0) | -0.00766 | Non-Toxin | ALLERGEN | NON-ALLERGEN | >10 hours | 7.2 hours | unstable |
| NEP |  |  |  |  |  |  |  |
| [FQDILLRMSK](https://webs.iiitd.edu.in/raghava/cellppd/pepsearch1.php?seq=FQDILLRMSK&thval=0.0) | -0.07662 | Non-Toxin | NON-ALLERGEN | NON-ALLERGEN | 2 min | 1.1 hours | unstable |
| [LQNRNEKWRE](https://webs.iiitd.edu.in/raghava/cellppd/pepsearch1.php?seq=LQNRNEKWRE&thval=0.0) | 0.15536 | Non-Toxin | NON-ALLERGEN | NON-ALLERGEN | 2 min | 5.5 hours | unstable |
